# Supplementary material for: Electrochemical Synthesis of Methoxy-NNO-azoxy Compounds via N=N Bond Formation Between Ammonium N-(methoxy)nitramide and Nitroso Compounds
Source: Molecules. 2025 Dec 10;30(24):4723. doi: 10.3390/molecules30244723 (PMC12736137; doi:10.3390/molecules30244723)

Supporting Information for

Electrochemical synthesis of methoxy-*NNO*-azoxy compounds via N=N bond formation between ammonium *N*-(methoxy)nitramide and nitroso compounds

Alexander S. Budnikov,[a] Andrey A. Kulikov,[a] Michael S. Klenov,*[a] Nikita E. Leonov,[a] Igor B. Krylov,*[a] Alexander O. Terent'ev,[a] and Vladimir A. Tartakovsky[a]

[a] N. D. Zelinsky Institute of Organic Chemistry, Russian Academy of Sciences, 47 Leninsky prosp., 119991 Moscow, Russian Federation

E-mail: krylovigor@yandex.ru (I.B.K.); klenov@ioc.ac.ru (M.S.K.)

Table of contents

1. General information S5

2. Experimental data S6

3. Characterization of Products S11

4. Pictures of the Equipment Used S18

5. X-ray single-crystal diffraction: Structure determination of compounds **2c**, **2d**, **2p** and **2s** S18

6. References S25

9. NMR Data S28

9.1.1 ^{1}H NMR spectrum of compound **2a** [500.13 MHz, $CDCl_3$] S28

9.1.2 ^{13}C NMR spectrum of compound **2a** [125.76 MHz, $CDCl_3$] S29

9.1.3 {^{1}H–^{13}C} HSQC spectrum of compound **2a** [500.13 MHz, $CDCl_3$] S30

9.1.4 {^{1}H–^{13}C} HMBC spectrum of compound **2a** [500.13 MHz, $CDCl_3$] S31

9.1.5 ^{14}N NMR spectrum of compound **2a** [36.14 MHz, $CDCl_3$] S32

9.2.1 ^{1}H NMR spectrum of compound **2b** [500.13 MHz, $CDCl_3$] S33

9.2.2 ^{13}C NMR spectrum of compound **2b** [125.76 MHz, $CDCl_3$] S34

9.2.3 {^{1}H–^{13}C} HSQC spectrum of compound **2b** [500.13 MHz, $CDCl_3$] S35

9.2.4 {^{1}H–^{13}C} HMBC spectrum of compound **2b** [500.13 MHz, $CDCl_3$] S36

9.2.5 ^{14}N NMR spectrum of compound **2b** [36.14 MHz, $CDCl_3$] S37

9.3.1 ^{1}H NMR spectrum of compound **2c** [500.13 MHz, $CDCl_3$] S38

9.3.2 ^{13}C NMR spectrum of compound **2c** [125.76 MHz, $CDCl_3$] S39

9.3.3 {^{1}H–^{13}C} HSQC spectrum of compound **2c** [500.13 MHz, $CDCl_3$] S40

9.3.4 {^{1}H–^{13}C} HMBC spectrum of compound **2c** [500.13 MHz, $CDCl_3$] S41

9.3.5 ^{14}N NMR spectrum of compound **2c** [43.37 MHz, $CDCl_3$] S42

9.4.1 ^{1}H NMR spectrum of compound **2d** [500.13 MHz, $CDCl_3$] S43

9.4.2 ^{13}C NMR spectrum of compound **2d** [125.76 MHz, $CDCl_3$] S44

9.4.3 {^{1}H–^{13}C} HSQC spectrum of compound **2d** [500.13 MHz, $CDCl_3$] S45

9.4.4 {^{1}H–^{13}C} HMBC spectrum of compound **2d** [500.13 MHz, $CDCl_3$] S46

9.4.5 ^{14}N NMR spectrum of compound **2d** [36.14 MHz, $CDCl_3$] S47

9.5.1 ^{1}H NMR spectrum of compound **2e** [500.13 MHz, $CDCl_3$] S48

9.5.2 ^{13}C NMR spectrum of compound **2e** [125.76 MHz, $CDCl_3$] S49

9.5.3 {^{1}H–^{13}C} HSQC spectrum of compound **2e** [500.13 MHz, $CDCl_3$] S50

9.5.4 {^{1}H–^{13}C} HMBC spectrum of compound **2e** [500.13 MHz, $CDCl_3$] S51

9.5.5 ^{14}N NMR spectrum of compound **2e** [36.14 MHz, $CDCl_3$] S52

9.6.1 ^{1}H NMR spectrum of compound **2f** [600.13 MHz, $CDCl_3$] S53

9.6.2 ^{13}C NMR spectrum of compound **2f** [150.90 MHz, $CDCl_3$] S54

9.6.3 {^{1}H–^{13}C} HSQC spectrum of compound **2f** [600.13 MHz, $CDCl_3$] S55

9.6.4 {^{1}H–^{13}C} HMBC spectrum of compound **2f** [600.13 MHz, $CDCl_3$] S56
9.6.5 ^{14}N NMR spectrum of compound **2f** [43.37 MHz, $CDCl_3$] S57
9.7.1 ^{1}H NMR spectrum of compound **2g** [600.13 MHz, $CDCl_3$] S58
9.7.2 ^{13}C NMR spectrum of compound **2g** [150.90 MHz, $CDCl_3$] S59
9.7.3 {^{1}H–^{13}C} HSQC spectrum of compound **2g** [600.13 MHz, $CDCl_3$]...................S60
9.7.4 {^{1}H–^{13}C} HMBC spectrum of compound **2g** [600.13 MHz, $CDCl_3$] S61
9.7.5 ^{14}N NMR spectrum of compound **2g** [43.14 MHz, $CDCl_3$] S62
9.8.1 ^{1}H NMR spectrum of compound **2h** [500.13 MHz, $CDCl_3$] S63
9.8.2 ^{13}C NMR spectrum of compound **2h** [125.76 MHz, $CDCl_3$] S64
9.8.3 {^{1}H–^{13}C} HSQC spectrum of compound **2h** [500.13 MHz, $CDCl_3$]...................S65
9.8.4 {^{1}H–^{13}C} HMBC spectrum of compound **2h** [500.13 MHz, $CDCl_3$] S66
9.8.5 ^{14}N NMR spectrum of compound **2h** [36.14 MHz, $CDCl_3$] S67
9.9.1 ^{1}H NMR spectrum of compound **2i** [500.13 MHz, $CDCl_3$]................................S68
9.9.2 ^{13}C NMR spectrum of compound **2i** [125.76 MHz, $CDCl_3$] S69
9.9.3 {^{1}H–^{13}C} HSQC spectrum of compound **2i** [500.13 MHz, $CDCl_3$]....................S70
9.9.4 {^{1}H–^{13}C} HMBC spectrum of compound **2i** [500.13 MHz, $CDCl_3$]....................S71
9.9.5 ^{14}N NMR spectrum of compound **2i** [36.14 MHz, $CDCl_3$] S72
9.10.1 ^{1}H NMR spectrum of compound **2j** [500.13 MHz, $CDCl_3$].............................S73
9.10.2 ^{13}C NMR spectrum of compound **2j** [125.76 MHz, $CDCl_3$] S74
9.10.3 {^{1}H–^{13}C} HSQC spectrum of compound **2j** [500.13 MHz, $CDCl_3$].................S75
9.10.4 {^{1}H–^{13}C} HMBC spectrum of compound **2j** [500.13 MHz, $CDCl_3$].................S76
9.10.5 ^{14}N NMR spectrum of compound **2j** [36.14 MHz, $CDCl_3$] S77
9.10.6 ^{19}F NMR spectrum of compound **2j** [470.59 MHz, $CDCl_3$] S78
9.11.1 ^{1}H NMR spectrum of compound **2k** [500.13 MHz, $CDCl_3$].............................S79
9.11.2 ^{13}C NMR spectrum of compound **2k** [125.76 MHz, $CDCl_3$] S80
9.11.3 {^{1}H–^{13}C} HSQC spectrum of compound **2k** [500.13 MHz, $CDCl_3$].................S81
9.11.4 {^{1}H–^{13}C} HMBC spectrum of compound **2k** [500.13 MHz, $CDCl_3$].................S82
9.11.5 ^{14}N NMR spectrum of compound **2k** [36.14 MHz, $CDCl_3$] S83
9.11.6 ^{19}F NMR spectrum of compound **2k** [470.59 MHz, $CDCl_3$] S84
9.12.1 ^{1}H NMR spectrum of compound **2l** [500.13 MHz, $CDCl_3$].............................S85
9.12.2 ^{13}C NMR spectrum of compound **2l** [125.76 MHz, $CDCl_3$] S86
9.12.3 {^{1}H–^{13}C} HSQC spectrum of compound **2l** [500.13 MHz, $CDCl_3$]..................S87
9.12.4 {^{1}H–^{13}C} HMBC spectrum of compound **2l** [500.13 MHz, $CDCl_3$]..................S88
9.12.5 ^{14}N NMR spectrum of compound **2l** [36.14 MHz, $CDCl_3$] S89
9.13.1 ^{1}H NMR spectrum of compound **2m** [600.13 MHz, $CDCl_3$] S90
9.13.2 ^{13}C NMR spectrum of compound **2m** [150.90 MHz, $CDCl_3$] S91

9.13.3 {^{1}H–^{13}C} HSQC spectrum of compound **2m** [600.13 MHz, $CDCl_3$]................S92
9.13.4 {^{1}H–^{13}C} HMBC spectrum of compound **2m** [600.13 MHz, $CDCl_3$] S93
9.13.5 ^{14}N NMR spectrum of compound **2m** [43.37 MHz, $CDCl_3$]............................S94
9.14.1 ^{1}H NMR spectrum of compound **2n** [600.13 MHz, $CDCl_3$] S95
9.14.2 ^{13}C NMR spectrum of compound **2n** [150.90 MHz, $CDCl_3$] S96
9.14.3 {^{1}H–^{13}C} HSQC spectrum of compound **2n** [600.13 MHz, $CDCl_3$].................S97
9.14.4 {^{1}H–^{13}C} HMBC spectrum of compound **2n** [600.13 MHz, $CDCl_3$] S98
9.14.5 ^{14}N NMR spectrum of compound **2n** [43.37 MHz, $CDCl_3$].............................S99
9.15.1 ^{1}H NMR spectrum of compound **2o** [500.13 MHz, $CDCl_3$] S100
9.15.2 ^{13}C NMR spectrum of compound **2o** [125.76 MHz, $CDCl_3$] S101
9.15.3 {^{1}H–^{13}C} HSQC spectrum of compound **2o** [500.13 MHz, $CDCl_3$]...............S102
9.15.4 {^{1}H–^{13}C} HMBC spectrum of compound **2o** [500.13 MHz, $CDCl_3$] S103
9.15.5 ^{14}N NMR spectrum of compound **2o** [36.14 MHz, $CDCl_3$] S104
9.16.1 ^{1}H NMR spectrum of compound **2p** [500.13 MHz, [D_6]acetone]..................S105
9.16.2 ^{13}C NMR spectrum of compound **2p** [125.76 MHz, [D_6]acetone] S106
9.16.3 {^{1}H–^{13}C} HSQC spectrum of compound **2p** [500.13 MHz, [D_6]acetone]......S107
9.16.4 {^{1}H–^{13}C} HMBC spectrum of compound **2p** [500.13 MHz, [D_6]acetone]......S108
9.16.5 ^{14}N NMR spectrum of compound **2p** [36.14 MHz, [D_6]acetone] S109
9.17.1 ^{1}H NMR spectrum of compound **2q** [500.13 MHz, $CDCl_3$] S110
9.17.2 ^{13}C NMR spectrum of compound **2q** [125.76 MHz, $CDCl_3$] S111
9.17.3 {^{1}H–^{13}C} HSQC spectrum of compound **2q** [500.13 MHz, $CDCl_3$]...............S112
9.17.4 {^{1}H–^{13}C} HMBC spectrum of compound **2q** [500.13 MHz, $CDCl_3$] S113
9.17.5 ^{14}N NMR spectrum of compound **2q** [36.14 MHz, $CDCl_3$]...........................S114
9.18.1 ^{1}H NMR spectrum of compound **2r** [500.13 MHz, [D_6]acetone] S115
9.18.2 ^{13}C NMR spectrum of compound **2r** [125.76 MHz, [D_6]acetone].................S116
9.18.3 {^{1}H–^{13}C} HSQC spectrum of compound **2r** [500.13 MHz, [D_6]acetone].......S117
9.18.4 {^{1}H–^{13}C} HMBC spectrum of compound **2r** [500.13 MHz, [D_6]acetone] S118
9.18.5 ^{14}N NMR spectrum of compound **2r** [36.14 MHz, [D_6]acetone] S119
9.19.1 ^{1}H NMR spectrum of compound **2s** [600.13 MHz, [D_6]acetone]..................S120
9.19.2 ^{13}C NMR spectrum of compound **2s** [150.90 MHz, [D_6]acetone]................S121
9.19.3 {^{1}H–^{13}C} HSQC spectrum of compound **2s** [600.13 MHz, [D_6]acetone]......S122
9.19.5 ^{14}N NMR spectrum of compound **2s** [43.37 MHz, [D_6]acetone]...................S123
9.20.1 ^{1}H NMR spectrum of compound **4a** [600.13 MHz, $CDCl_3$]...........................S124
9.20.2 ^{13}C NMR spectrum of compound **4a** [150.90 MHz, $CDCl_3$] S125
9.20.3 ^{14}N NMR spectrum of compound **4a** [43.37 MHz, $CDCl_3$] S126

]\1. General information

Safety precautions

Although we have encountered no difficulties during preparation and handling of compounds described and used in this paper, they are explosive energetic materials which are sensitive to impact and friction. Mechanical actions of these energetic materials, involving scratching or scraping, must be avoided.

^{1}H, ^{13}C, ^{14}N, ^{19}F NMR spectra were recorded with Bruker DRX-500 (500.1, 125.8, 36.1, 470.59 MHz, respectively) and Bruker AV600 (600.1, 150.9, 43.4 MHz, respectively) spectrometers. *Chemical shifts* are reported in delta (*δ*) *units*, parts per million (ppm) downfield from internal TMS (^{1}H, ^{13}C) or external CH_3NO_2 (^{14}N negative values of δ_N correspond to upfield shifts). The IR spectra were recorded with a Bruker ALPHA-T spectrometer in the range 400–4000 cm^{-1} (resolution 2 cm^{-1}) as pellets with KBr or as a thin layer. High-resolution ESI mass spectra (HRMS) were recorded with a Bruker micrOTOF II instrument. Silica gel 60 Merck (15–40 µm) was used for preparative column and thin-layer chromatography. Analytical thin-layer chromatography (TLC) was carried out on Merck silica gel 60 F254 aluminum sheets. All reagents were purchased from Acros and Sigma-Aldrich. *Solvents were purified* before use, according to standard procedures. All other reagents were used without further purification. Nitrosobenzene (**1a**),[1] 1-nitro-2-nitrosobenzene (**1b**),[2] 1-nitro-3-nitrosobenzene (**1c**),[3] 1-nitro-4-nitrosobenzene (**1d**),[4] 1-methoxy-2-nitrosobenzene (**1e**),[5] 1-methoxy-3-nitrosobenzene (**1f**),[6] 1-methoxy-4-nitrosobenzene (**1g**),[7] 1,3-dichloro-2-nitrosobenzene (**1h**),[8] 1,3-dibromo-2-nitrosobenzene (**1i**),[8] 2-nitroso-1-(trifluoromethyl)benzene (**1j**),[9] 3-nitroso-1-(trifluoromethyl)benzene (**1k**),[10] 2-nitro-2-nitrosopropane (**1l**),[11] 1-nitro-1-nitrosocyclopentane (**1m**),[12] 1-nitro-1-nitrosocyclohexane (**1n**),[13] 2-nitro-2-nitroso-1,3-diphenylpropane (**1o**),[11] 2,2-dimethyl-5-nitro-5-nitroso-1,3-dioxane (**1p**),[14] 2-nitrosopyridine (**1q**),[15] 2-methyl-3-nitroso-5-nitro-2*H*-triazole (**1r**),[16] 2-methyl-5-nitroso-2*H*-tetrazole (**1s**),[17] ammonium *N*-(methoxy)nitramide (**3**)[18] and dibromoisocyanuric acid (DBI)[19] were prepared according to the reported procedures.

2. Experimental data

2.1 General Procedure for the Optimization of the Reaction Conditions for the Synthesis of 1-(Methoxy-*NNO*-azoxy)benzene (2a) from 1-Nitrosobenzene (1a) *via* oxidative coupling (Experimental details for Scheme 2)

To a stirred suspension of **1a** (59 mg, 0.50 mmol) and PhI(OAc)$_2$ (161–644 mg, 0.50–2.00 mmol) or dimbromoisocyanuric acid (DBI) (144–574 mg, 0.50–2.00 mmol) in dry solvent (2 mL, see Table S1) at 0 °C under an argon atmosphere a solution of MeONH$_2$ (24–47 mg, 0.50–1.00 mmol) in dry CH$_2$Cl$_2$ (1 mL) was added dropwise. Then the reaction mixture was vigorously stirred at 0–40 °C (see Table S1) for 2 h. In case of the PhI(OAc)$_2$ reaction mixture was concentrated under reduced pressure. When DBI was used the formed precipitate was filtered off, washed with CH$_2$Cl$_2$ (3 × 2 mL) and then combined filtrates were concentrated under reduced pressure. The yields of **2a** were determined with the use of ^{1}H NMR spectroscopy using 1,1,2,2-tetrachloroethane as an internal standard (see Table S1).

Table S1. Optimization of the oxidative coupling of 1-nitrosobenzene (**1a**) with methoxyamine.

Entry	Oxidizer	Solvent	Stoichiometric ratio, [Ox] : MeONH$_2$: 1a	Temperature, °C	Yield 2a, %
**1**	**PhI(OAc)$_2$**	**CH$_2$Cl$_2$**	**1 : 1 : 1**	**25**	**39**
2	PhI(OAc)$_2$	Et$_2$O	1 : 1 : 1	25	21
3	PhI(OAc)$_2$	MeCN	1 : 1 : 1	25	15
4	PhI(OAc)$_2$	CH$_2$Cl$_2$	2 : 1 : 1	25	13
5	PhI(OAc)$_2$	CH$_2$Cl$_2$	2 : 2 : 1	25	25
6	PhI(OAc)$_2$	CH$_2$Cl$_2$	4 : 2 : 1	25	7
8	DBI	CH$_2$Cl$_2$	1 : 1 : 1	25	18
9	DBI	CH$_2$Cl$_2$	4 : 2 : 1	25	8
10	PhI(OAc)$_2$	CH$_2$Cl$_2$	1 : 1 : 1	0	30
11	PhI(OAc)$_2$	CH$_2$Cl$_2$	1 : 1 : 1	40	23

2.2 General Procedure for the Optimization of the Reaction Conditions for the Synthesis of 1-(Methoxy-*NNO*-azoxy)benzene (2a) from 1-Nitrosobenzene (1a) and 3 without electricity (Experimental details for Table 1)

To a stirred solution of **1a** (59 mg, 0.50 mmol) in 2 mL DMSO, MeCN, MeCN/MeOH at 25 °C ammonium *N*-(methoxy)nitramide (**3**) (55 mg, 0.50 mmol) was added. Then the reaction mixture was vigorously stirred at 25–50 °C for 3h – 9 days. The precipitate was then filtered off, washed with MeCN (2 × 2 mL). The combined filtrates were concentrated under reduced pressure. The yields of **2a** were determined with the use of ^{1}H NMR spectroscopy using 1,1,2,2-tetrachloroethane as an internal standard.

2.3 General Procedure for the Screening of the Reaction Conditions in an Undivided Electrochemical Cell for the Synthesis of (Methoxy-*NNO*-azoxy)benzene (2a) from Nitrosobenzene (1a) (Experimental details for Table 2)

An undivided 10 mL electrochemical cell was equipped with a platinum plate, carbon felt, or glassy carbon anode (30 × 15 mm), and a platinum wire (d = 1 mm, l = 113 mm, n_{coils} = 9), or stainless steel (SS) cathode, connected to a DC-regulated power supply. The electrodes were fully immersed, providing a total working surface area (S) of 4.5 cm^2. A solution of nitrosobenzene **1a** (0.5 mmol, 54 mg), ammonium *N*-(methoxy)nitramide **3** (1–2 mmol, 109–218 mg), and a supporting electrolyte (0–0.5 mmol, 0–164 mg) in 10 mL of solvent (MeCN, MeCN/H_2O, DMF, MeOH, CH_2Cl_2/H_2O, or DMSO) was subjected to constant-current electrolysis at 60 mA and 23–25 °C with magnetic stirring. After passing a charge of 2 F·mol^{-1} (27 min), the electrodes were washed with CH_2Cl_2 (3 × 20 mL). The combined organic phase was washed with H_2O (20 mL) and brine (20 mL), dried over Na_2SO_4, and solvent removed in vacuo. The yields of **2a** were determined with the use of ^{1}H NMR spectroscopy using 1,1,2,2-tetrachloroethane as an internal standard.

2.4 General Procedure for the Optimization of the Reaction Conditions in a Divided Electrochemical Cell for the Synthesis of (Methoxy-*NNO*-azoxy)benzene (2a) from Nitrosobenzene (1a) and 3 (Experimental details for Table 3)

A divided *H*-type electrochemical cell (volume of each compartment ~ 15 mL, divided with Celgard® 2400 membrane) was equipped with a platinum, nickel, graphite, carbon felt, and glassy carbon plate anode (30 × 15 mm^2) and a platinum, stainless steel, nickel, glassy carbon plate cathode (30 × 15 mm^2), and connected to a DC regulated power supply. A solution of nitrosobenzene **1a** (0.5 mmol, 54 mg), ammonium *N*-(methoxy)nitramide **3** (0.5–1.5 mmol, 54–163 mg), and supporting electrolyte (1 mmol, 104–369 mg) in solvent (12 mL) was placed in the anodic compartment of a divided electrochemical cell. The cathodic compartment was filled with a solution of supporting electrolyte (1 mmol, 104–369 mg) in the same solvent (12 mL). Electrolysis was carried out under constant current (I = 10–30 mA, 1–3 F per mole **1a**) at 23–25 °C under magnetic stirring. After passing 1–3 F·mol^{-1} of electricity (reaction time 27–160 min), electrodes were washed with CH_2Cl_2 (3 × 20 mL). The combined organic phase was washed with H_2O (2x20 mL), dried over Na_2SO_4, and solvent removed in vacuo. The yields of **2a** were determined with the use of ^{1}H NMR spectroscopy using 1,1,2,2-tetrachloroethane as an internal standard. In run 22, **2a** was isolated by column chromatography on silica gel (R_f

= 0.43, petroleum ether/EtOAc, 3:1) to afford the desired product (53 mg, 70%) as a pale-yellow crystals. mp: 40–41 °C. The synthesized compound **2a** was identical (^{1}H, ^{13}C, and ^{14}N NMR, TLC) to the compound prepared according to the reported procedure.[20]

2.5 Typical Procedure for Electrochemical Synthesis of (Methoxy-*NNO*-Azoxy)compounds 2a–2s (Experimental details for Scheme 3, Method A)

Electrolysis was conducted in a divided H-cell (15 mL per compartment) equipped with a Celgard® 2400 membrane, using a glassy carbon anode (30 × 15 mm²) and stainless steel cathode (30 × 15 mm²) connected to a DC power supply. A solution of nitroso compound **1** (0.5 mmol, 54-135 mg), **3** (1 mmol, 109 mg), and *n*-Bu_4NBF_4 (1 mmol, 329 mg) in DMSO (12 mL) was placed in the anodic compartment of a divided electrochemical cell. The cathodic compartment was filled with a solution of *n*-Bu_4NBF_4 (1 mmol, 329 mg) in DMSO (12 mL). Electrolysis was carried out under constant current (I = 30 mA, 2 F per mole 1) at 23–25 °C under magnetic stirring. After passing 2 $F \cdot mol^{-1}$ of electricity (reaction time 54 min), electrodes were washed with CH_2Cl_2 (3 × 20 mL). The combined organic phase was washed with H_2O (2x20 mL), dried over Na_2SO_4, and solvent was evaporated under reduced pressure. Synthesized products **2a**–**2s** were purified by column chromatography on silica gel (petroleum ether/EtOAc, 5:1 – 1:1). In case of the compound **1s** – 5,5-azoxy-bis-2,2-methyltetrazole (**5**) (21 mg, 40%) was also isolated. This product was identical (TLC, ^{1}H and ^{13}C NMR, HRMS) to the compound prepared according to the reported procedure.[17]

2.6 Typical Procedure for Synthesis of (Methoxy-*NNO*-Azoxy)compounds 2a–2s with 3 without electricity (Experimental details for Scheme 3, Method B)

To a stirred solution of **1** (0.50 mmol) in 2 mL of MeCN/MeOH (1/1) mixture at 25 °C, **3** (55 mg, 0.50 mmol) was added. Then the reaction mixture was vigorously stirred at this temperature for the given time (see Table S2). The precipitate was then filtered off, washed with MeCN (2 × 2 mL). The combined filtrates were concentrated under reduced pressure. Products **2a**–**2s** purified by column chromatography on silica gel.

Table S2. Reaction time of nitroso compounds **1** with **3** without electricity.

Substrate	1a	1b	1c	1d	1i	1j	1k	1l	1m	1n	1o	1p	1q	1r	1s
Reaction time, days	5	4	3	4	4	3	3	2	5	7	5	2	8	2	8

2.7 Typical Procedure for Synthesis of (Methoxy-*NNO*-Azoxy)compounds 2a–2s via oxidative coupling (Experimental details for Scheme 3, Method C)

To a stirred suspension of **1** (0.50 mmol) and PhI(OAc)$_2$ (161 mg, 0.50 mmol) in dry CH_2Cl_2 (2 mL) at 0 °C under an argon atmosphere a solution of $MeONH_2$ (24 mg, 0.50 mmol) in dry CH_2Cl_2 (1 mL) was added dropwise. Then the reaction mixture was vigorously stirred at 25 °C for 2 h. After reaction completion mixture was concentrated under reduced pressure. Products **2a**–**2s** were isolated by column chromatography on silica gel.

2.8 Reaction under Controlled Potential Electrolysis (Experimental details for Scheme 4)

Controlled-potential electrolysis was performed in an undivided 20 mL electrochemical cell using a glassy carbon anode (30 × 15 mm², S = 4.5 cm²), a stainless steel cathode (30 × 15 mm²), and an $Ag/AgNO_3$ reference electrode. A solution of nitrosobenzene **1a** (0.5 mmol, 54 mg) and **3** (1.0 mmol, 109 mg) in DMSO (12 mL) was electrolyzed at 370 mV (*vs.* $Ag/AgNO_3$) at 23–25 °C. Upon passing of 2.0 $F{\cdot}mol^{-1}$ of electricity, the electrodes were washed with CH_2Cl_2 (3 × 20 mL). The combined organic extract was washed with water (2 × 20 mL), dried (Na_2SO_4), and concentrated in vacuo. The yield of **2a** was determined according to ^1H NMR spectroscopy using 1,1,2,2-tetrachloroethane as an internal standard.

2.9 Cyclic Voltammetry Studies

Cyclic voltammetry (CV) was implemented on PS-30 computer-assisted potentiostat-galvanostat manufactured by «SmartStat», the scan rate was 100 $mV{\cdot}s^{-1}$. Cyclic voltammetry (CV) experiments were conducted in a 10 mL water-jacketed, five-necked conical glass cell using a standard three-electrode configuration. A typical measurement utilized a 5 mL solution under thermostatic control at 21.0 ± 0.5 °C. The working electrode was a glassy carbon disk (*d* = 3 mm), the counter electrode was a platinum wire, and the reference electrode was $Ag/AgNO_3$ (0.1 M in 0.1 M n-Bu_4NBF_4/MeCN), connected to the solution via a porous glass diaphragm. All solutions were deaerated by argon purging prior to measurement, and the experiments were performed under an argon atmosphere. The working electrode was polished before recording each CV-curve.

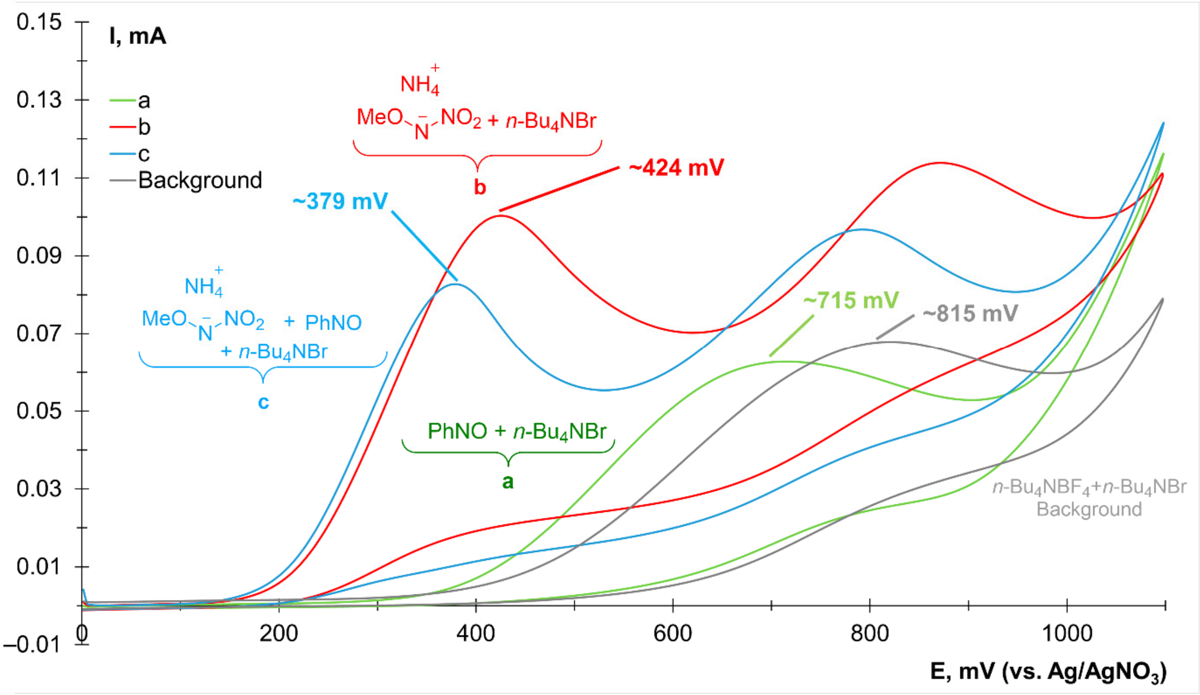

Figure S1. CV-curves of 0.01 M solutions of a) **1a** with n-Bu_4NBr (green), b) **3** with n-Bu_4NBr (red), c) **1a** and **3** with n-Bu_4NBr (blue), and n-Bu_4NBr in 0.1 M n-Bu_4NBF_4 solution (Background, grey) in DMSO on a working glassy-carbon electrode (d = 3 mm) under a scan rate of 0.1 $V{\cdot}s^{-1}$ at 298K.

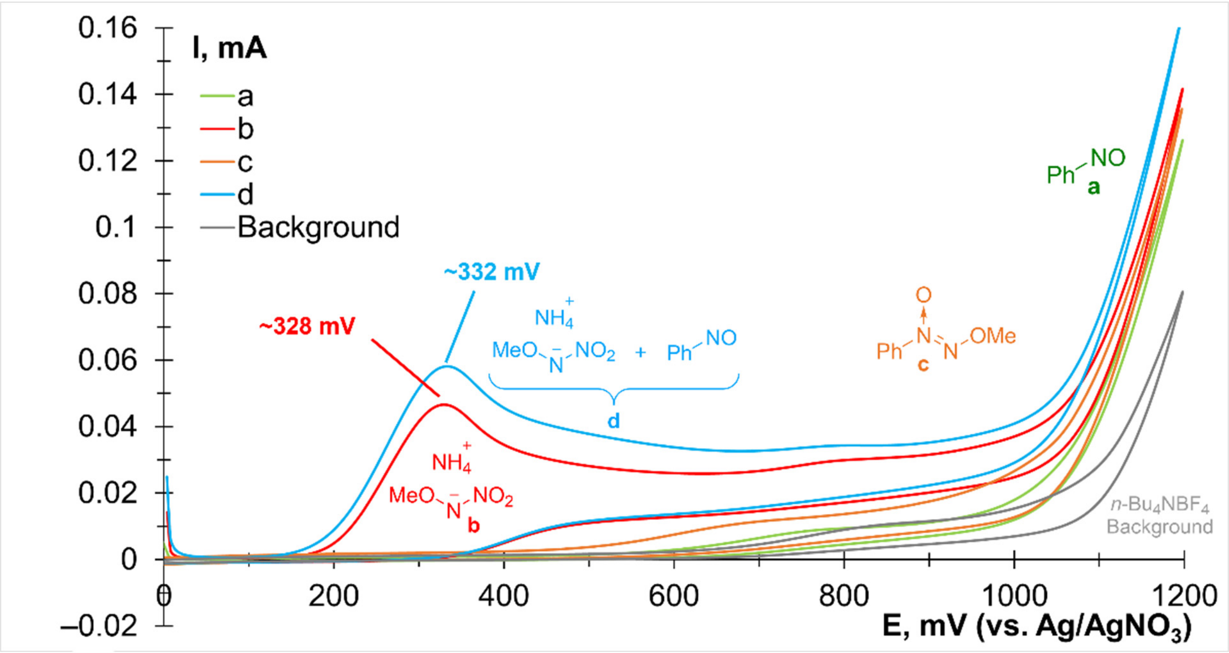

Figure S2. CV-curves of 0.01 M solutions of a) **1a** (green), b) **3** (red), c) **2a** (yellow) and d) **1a** and **3** (blue) in 0.1 M n-Bu_4NBF_4 solution in DMSO on a working glassy-carbon electrode (d = 3 mm) under a scan rate of 0.1 $V{\cdot}s^{-1}$ at 298K.

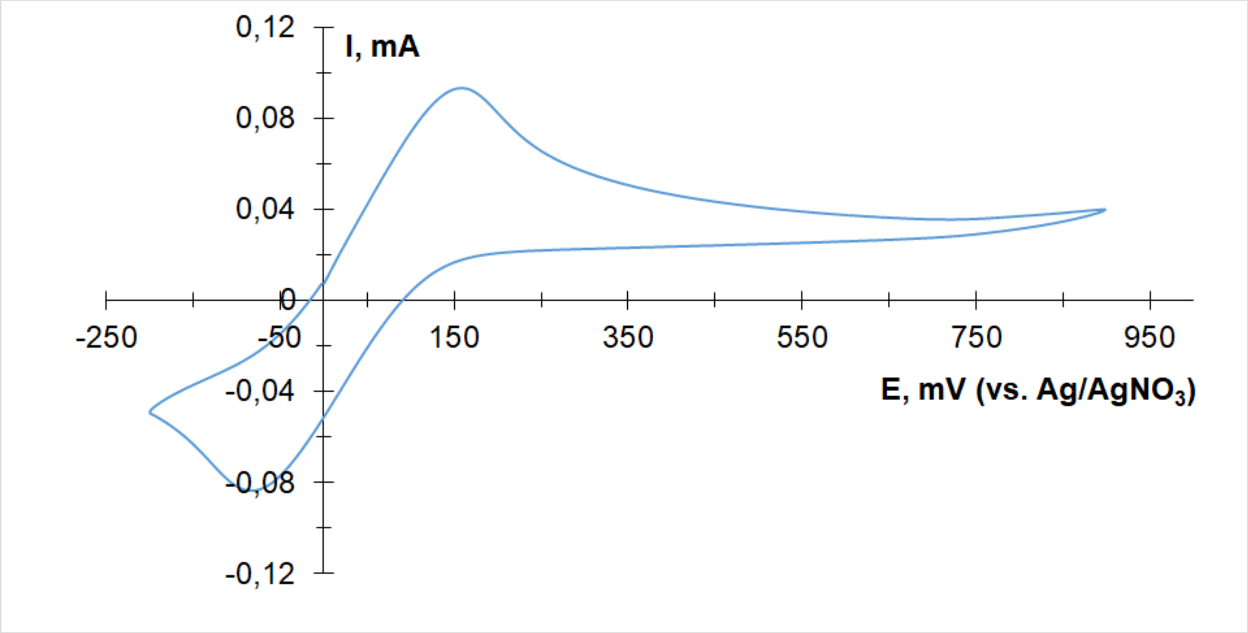

Figure S3. CV-curve of 0.01 M solution of ferrocene in 0.1 M *n*-Bu_4NBF_4 solution in DMSO on a working glassy-carbon electrode (*d* = 3 mm) under a scan rate of 0.1 V·s^{-1} at 298 K.

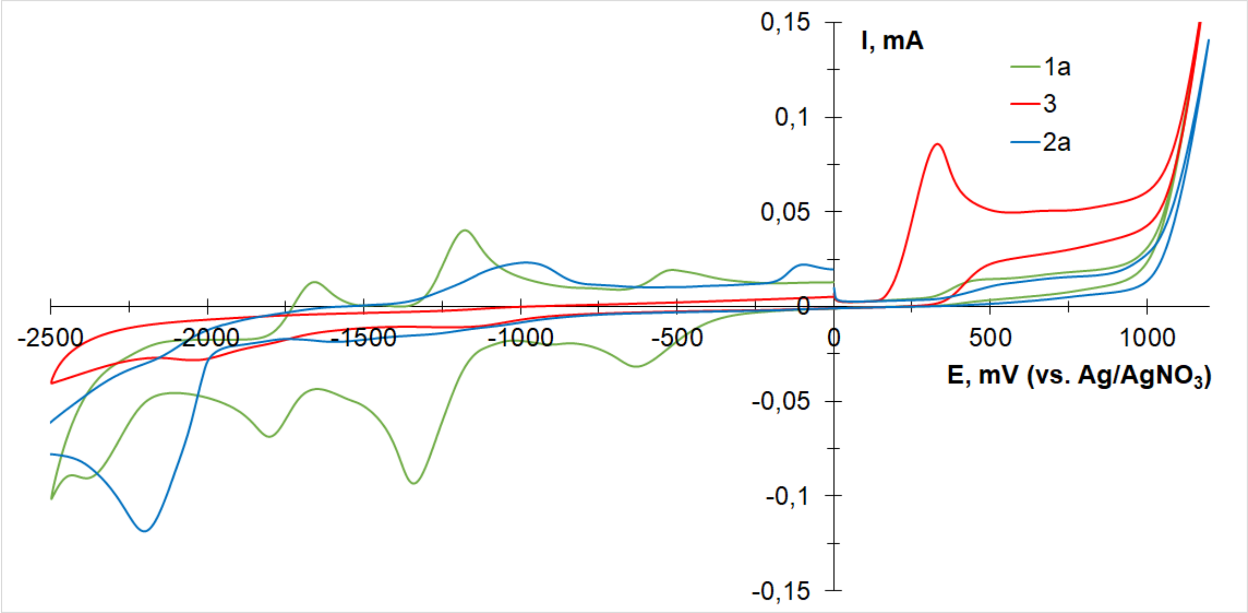

Figure S4. CV-curves of 0.01 M solutions of a) **1a** (green), b) **3** (red), c) **2a** (blue) in 0.1 M *n*-Bu_4NBF_4 solution in DMSO on a working glassy-carbon electrode (d = 3 mm) under a scan rate of 0.1 V·s^{-1} at 298K.

3. Characterization of Products

1-(Methoxy-*NNO*-azoxy)benzene (2a): Pale yellow crystals, m.p. 40–41 °C (lit. m.p. 39.5–40.5 °C).[21] R_f (petroleum ether/ethyl acetate, 3:1) = 0.43. ^{1}H NMR (500.13 MHz, $CDCl_3$)δ: 4.24 (s, 3H, OMe), 7.48, 7.50 (m, 2H, H(3,5), 7.52, 7.53 (m, 1H, H(4)), 7.99 (d, 2H, H(2,6), $^3J_{HH}$ = 7.7 Hz) ppm. ^{13}C NMR (125.76 MHz, $CDCl_3$)δ: 61.4 (s, OMe), 120.6 (s, C(2,6)), 128.5 (s, C(3,5)), 130.8 (s, C(4)), 142.5 (br. s, C(1)) ppm. The ^{1}H–^{13}C HSQC and HMBC experiments were used to assign the signals. ^{14}N NMR (36.14 MHz, $CDCl_3$)δ: –68

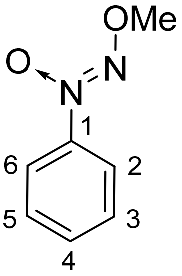

(N(O)=N–OMe, $\Delta\nu_{½}$ = 90 Hz) ppm. IR (KBr): ν = 2946 (m), 1479 (s), 1432 (s), 1122 (m), 1046 (s), 1018 (m) cm^{-1}. HRMS (ESI): m/z calcd for $C_7H_8N_2O_2$ $[M+H]^+$ 153.0659; found 153.0659. Elemental analysis calcd (%) for $C_7H_8N_2O_2$: C 55.26, H 5.30, N 18.41; found: C 55.30, H 5.32, N 18.35.

1-(Methoxy-*NNO*-azoxy)-2-nitrobenzene (2b): Yellow crystals, m.p. 59–63 °C (lit. m.p. 66.5–67.3 °C).[21] R_f (petroleum ether/ethyl acetate, 3:1) = 0.20. ^{1}H NMR (500.13 MHz, $CDCl_3$)*δ*: 4.22 (s, 3H, OMe), 7.73–8.04 (m, 4H, H(3,4,5,6)) ppm. ^{13}C NMR (125.76 MHz, $CDCl_3$)*δ*: 62.2 (s, OMe), 125.3, 126.2 (C(3,6)), 131.8, 133.9 (C(4,5)), 136.7 (br. s, C(1)), 143.1 (br. s, C(2)) ppm. The ^{1}H–^{13}C HSQC and HMBC experiments were used to assign the signals. ^{14}N NMR (36.14 MHz, $CDCl_3$)*δ*: –16 (NO_2, $\Delta\nu_{½}$ = 90 Hz), –75 (N(O)=N–OMe, $\Delta\nu_{½}$ = 135 Hz) ppm. IR (KBr): ν = 3104 (w), 2950 (m), 1606 (m), 1536 (s), 1468 (s), 1442 (s), 1352 (s), 1316 (m), 1143 (s), 1085 (s), 1043 (s) cm^{-1}. HRMS (ESI): m/z calcd for $C_7H_7N_3O_4$ $[M+H]^+$ 198.0509; found 198.0503. Elemental analysis calcd (%) for $C_7H_7N_3O_4$: C 42.65, H 3.58, N 21.31; found: C 42.68, H 3.61, N 21.22.

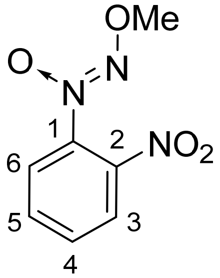

1-(Methoxy-*NNO*-azoxy)-3-nitrobenzene (2c): Yellowish crystals, m.p. 133–134 °C (lit. m.p. 133–134 °C).[21] R_f (petroleum ether/ethyl acetate, 3:1) = 0.27. ^{1}H NMR (500.13 MHz, $CDCl_3$)*δ*: 4.32 (s, 3H, OMe), 7.76 (t, 1H, H(5), $^3J_{HH}$ = 8.2 Hz), 8.40–8.43 (m, 2H, H(4,6)), 8.88 (s, 1H, H(2) ppm. ^{13}C NMR (125.76 MHz, $CDCl_3$)*δ*: 62.5 (s, OMe), 116.6 (s, C(2)), 125.9, 126.8 (C(4,6)), 130.4 (s, C(5)), 143.6 (br. s, C(1)), 148.3 (br. s, C(3)) ppm. The ^{1}H–^{13}C HSQC and HMBC experiments were used to assign the signals. ^{14}N NMR (36.14 MHz, $CDCl_3$)*δ*: –15 (NO_2, $\Delta\nu_{½}$ = 170 Hz), –73 (*N*(O)=N–OMe, $\Delta\nu_{½}$ = 135 Hz) ppm. IR (KBr): ν = 3084 (w), 1525 (s), 1478 (m), 1350 (s), 1068 (s), 1040 (s) cm^{-1}. HRMS (ESI): m/z calcd for $C_7H_7N_3O_4$ $[M+H]^+$ 198.0509; found 198.0503. Elemental analysis calcd (%) for $C_7H_7N_3O_4$: C 42.65, H 3.58, N 21.31; found: C 42.66, H 3.63, N 21.25.

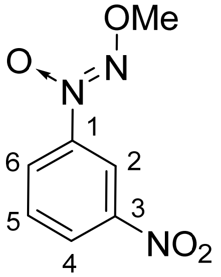

1-(Methoxy-*NNO*-azoxy)-4-nitrobenzene (2d): Yellowish crystals, m.p. 150–152 °C (lit. m.p. 152–153 °C).[21] R_f (petroleum ether/ethyl acetate, 3:1) = 0.27. ^{1}H NMR (500.13 MHz, $CDCl_3$)*δ*: 4.31 (s, 3H, OMe), 8.24 (d, 2H, H(2,6), $^3J_{HH}$ = 8.9 Hz), 8.37 (d, 2H, H(3,5), $^3J_{HH}$ = 8.9 Hz) ppm. ^{13}C NMR (125.76 MHz, $CDCl_3$)*δ*: 62.5 (s, OMe), 122.3 (s, C(2,6)), 124.6 (s, C(3,5)), 146.7 (br. s, C(1)), 149.2 (br. s, C(4)) ppm. The ^{1}H–^{13}C HSQC and HMBC experiments were used to assign the signals. ^{14}N NMR (36.14 MHz, $CDCl_3$)*δ*: –15 (NO_2,

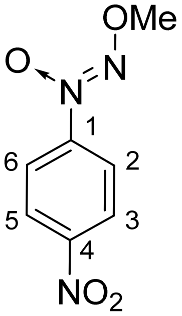

$\Delta\nu_{1/2}$ = 230 Hz), –50 ($\underline{\text{N}}$(O)=N–OMe, $\Delta\nu_{1/2}$ = 110 Hz) ppm. IR (KBr): ν = 3123 (w), 2951 (w), 1525 (s), 1460 (m), 1348 (s), 1323 (m), 1045 (s) cm^{-1}. HRMS (ESI): m/z calcd for $C_7H_7N_3O_4$ $[M+H]^+$ 198.0509; found 198.0508. Elemental analysis calcd (%) for $C_7H_7N_3O_4$: C 42.65, H 3.58, N 21.31; found: C 42.64, H 3.59, N 21.37.

1-Methoxy-2-(methoxy-*NNO*-azoxy)benzene (2e): Yellow oil. R_f (petroleum ether/ethyl acetate, 3:1) = 0.37. ^{1}H NMR (500.13 MHz, $CDCl_3$)δ: 3.86 (s, 3H, C–OMe), 4.23 (s, 3H, N–OMe), 7.48 (d, 1H, H(3), $^3J_{HH}$ = 8.3 Hz), 7.35 (t, 1H, H(4), $^3J_{HH}$ = 8.2 Hz), 7.52–7.56 (m, 2H, H(5,6)) ppm. ^{13}C NMR (125.76 MHz, $CDCl_3$)δ: 55.7 (s, C–OMe), 61.9 (s, N–OMe), 106.5, 113.2 (C(5,6)), 117.6 (s, C(3)), 129.7 (s, C(4)), 144.1 (br. s, C(2)), 160.0 (s, C(1)) ppm. The ^{1}H–^{13}C HSQC and HMBC experiments were used to assign the signals. ^{14}N NMR (36.14 MHz, $CDCl_3$)δ: –68 ($\underline{\text{N}}$(O)=N–NO_2, $\Delta\nu_{1/2}$ = 135 Hz) ppm. IR (KBr): ν = 2947 (s), 2837 (w), 1614 (s), 1588 (m), 1496 (s), 1461 (s), 1324 (m), 1285 (s), 1240 (s), 1108 (s), 1035 (s) cm^{-1}. HRMS (ESI): m/z calcd for $C_8H_{10}N_2O_3$ $[M+H]^+$ 183.0764; found 183.0765. Elemental analysis calcd (%) for $C_8H_{10}N_2O_3$: C 52.74, H 5.53, N 15.38; found: C 52.77, H 5.55, N 15.34.

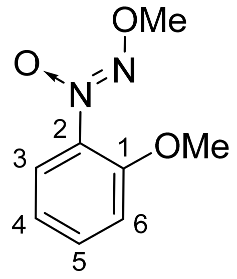

1-Methoxy-3-(methoxy-*NNO*-azoxy)benzene (2f): Yellow oil. R_f (petroleum ether/ethyl acetate, 3:1) = 0.37. ^{1}H NMR (600.13 MHz, $CDCl_3$)δ: 3.83 (s, 3H, OMe), 4.20 (s, 3H, N–OMe), 7.02 (dd, 1H, H(6), $^3J_{HH}$ = 8.3 Hz, $^4J_{HH}$ = 2.0 Hz), 7.32 (t, 1H, H(5), J_{HH} = 8.2 Hz), 7.49 (t, 1H, H(2), $^4J_{HH}$ = 2.2 Hz), 7.52 (dd, 1H, H(4), $^3J_{HH}$ = 8.1 Hz, $^4J_{HH}$ = 2.2 Hz) ppm. ^{13}C NMR (150.90 MHz, $CDCl_3$)δ: 56.4 (s, C–OMe), 62.6 (s, N–OMe), 107.2 (s, C(2)), 114.0 (s, C(4)), 118.3 (s, C(6)), 130.4 (s, C(5)), 144.8 (br. s, C(3)), 160.7 (s, C(1)) ppm. The ^{1}H–^{13}C HSQC and HMBC experiments were used to assign the signals. ^{14}N NMR (43.37 MHz, $CDCl_3$)δ: 1 (N(O)=$\underline{\text{N}}$–OMe, $\Delta\nu_{1/2}$ = > 1000 Hz), –69 ($\underline{\text{N}}$(O)=N–OMe, $\Delta\nu_{1/2}$ = 125 Hz) ppm. IR (KBr): ν = 2946 (m), 2837 (w), 1614 (s), 1496 (m), 1462 (m), 1285 (m), 1241 (w), 1108 (m), 1051 (s) cm^{-1}. HRMS (ESI): m/z calcd for $C_8H_{10}N_2O_3$ $[M+H]^+$ 183.0764; found 183.0767. Elemental analysis calcd (%) for $C_8H_{10}N_2O_3$: C 52.74, H 5.53, N 15.38; found: C 52.79, H 5.58, N 15.28.

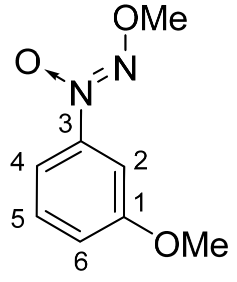

1-Methoxy-4-(methoxy-*NNO*-azoxy)benzene (2g): Beige crystals, m.p. 76–77 °C (lit. m.p. 62–63 °C).[22] R_f (petroleum ether/ethyl acetate, 3:1) = 0.31. ^{1}H NMR (600.13 MHz, $CDCl_3$)δ: 3.85 (s, 3H, C–OMe), 4.20 (s, 3H, N–OMe), 6.92 (d, 2H, H(3,5) $^3J_{HH}$ = 9.0 Hz), 7.91 (d, 2H, H(2,6), $^3J_{HH}$ = 9.0 Hz) ppm. ^{13}C NMR (150.90 MHz, $CDCl_3$)δ: 56.3 (s, C–OMe), 62.4 (N–OMe), 114.6 (s, C(3,5)), 123.3 (s, C(2,6)), 137.0 (br. s, C(4)), 162.4 (s, C(1)) ppm. The ^{1}H–^{13}C HSQC and HMBC experiments were used to assign the signals. ^{14}N NMR (43.37 MHz, $CDCl_3$)δ: –6 (N(O)=<u>N</u>–OMe, $\Delta\nu_{½}$ = > 1000 Hz), –69 (<u>N</u>(O)=N–OMe, $\Delta\nu_{½}$ = 130 Hz) ppm. IR (KBr): ν = 2945 (w), 1599 (m), 1505 (m), 1459 (m), 1256 (m), 1050 (s), 1018 (m) cm^{-1}. HRMS (ESI): m/z calcd for $C_8H_{10}N_2O_3$ [M+H]$^+$ 183.0764; found 183.0768. Elemental analysis calcd (%) for $C_8H_{10}N_2O_3$: C 52.74, H 5.53, N 15.38; found: C 52.73, H 5.54, N 15.34.

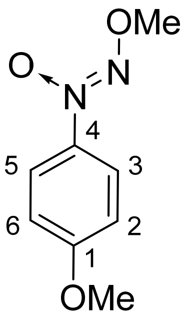

2,6-Dichloro-1-(methoxy-*NNO*-azoxy)benzene (2h): White crystals, m.p. 78–79 °C. R_f (petroleum ether/ethyl acetate, 5:1) = 0.20. ^{1}H NMR (500.13 MHz, $CDCl_3$)δ: 4.25 (s, 3H, OMe), 7.38–7.46 (m, 3H, H(3,4,5)) ppm. ^{13}C NMR (125.76 MHz, $CDCl_3$)δ: 62.2 (s, OMe), 128.9 (C(3,5)), 131.2 (C(2,6)), 131.7 (s, C(4)), 139.7 (br. s, C(1)) ppm. The ^{1}H–^{13}C HSQC and HMBC experiments were used to assign the signals. ^{14}N NMR (36.14 MHz, $CDCl_3$)δ: –76 (<u>N</u>(O)=N–OMe, $\Delta\nu_{½}$ = 200 Hz) ppm. IR (KBr): ν = 2917 (w), 2890 (w), 1625 (s), 1576 (m), 1556 (m), 1492 (s), 1448 (m), 1386 (w), 1372 (w), 1329 (m), 1301 (s), 1277 (s), 1206 (m), 1168 (m), 1043 (m) cm^{-1}. HRMS (ESI): m/z calcd for $C_7H_6Cl_2N_2O_2$ [M+H]$^+$ 220.9879; found 220.9877. Elemental analysis calcd (%) for $C_7H_6Cl_2N_2O_2$: C 38.04, H 2.74, N 12.67; found: C 38.05, H 2.76, N 12.62.

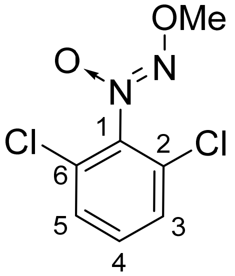

2,6-Dibromo-1-(methoxy-*NNO*-azoxy)benzene (2i): Pale-yellow crystals, m.p. 88–89 °C. R_f (petroleum ether/ethyl acetate, 5:1) = 0.17. ^{1}H NMR (500.13 MHz, $CDCl_3$)δ: 4.26 (s, 3H, OMe), 7.26 (t, 1H, H(4), $^3J_{HH}$ = 8.1 Hz), 7.64 (d, 2H, H(3,5), $^3J_{HH}$ = 8.1 Hz) ppm. ^{13}C NMR (125.76 MHz, $CDCl_3$)δ: 62.2 (s, OMe), 115.7 (s, C(2,6)), 132.3 (C(4)), 132.6 (s, C(3,5)), 142.6 (br. s, C(1)) ppm. The ^{1}H–^{13}C HSQC and HMBC experiments were used to assign the signals. ^{14}N NMR (36.14 MHz, $CDCl_3$)δ: –72 (*N*(O)=N–OMe, $\Delta\nu_{½}$ = 300 Hz) ppm. IR (KBr): ν = 2917 (w), 2889 (w), 2849 (w), 1567 (m), 1535 (s), 1490 (s), 1441 (m), 1288 (s), 1201 (m), 1068 (s), 1040 (s) cm^{-1}. HRMS (ESI): m/z calcd for $C_7H_6Br_2N_2O_2$ [M+H]$^+$ 310.8848; found 310.8847. Elemental analysis calcd (%) for $C_7H_6Br_2N_2O_2$: C 27.13, H, 1.95, N 9.04; found: C 27.18, H 1.97, N 8.95.

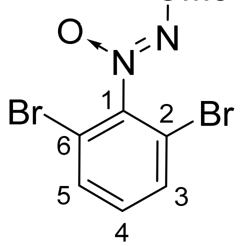

1-(Methoxy-*NNO*-azoxy)-2-(trifluoromethyl)benzene (2j): Yellowish oil. R_f (petroleum ether/ethyl acetate, 5:1) = 0.17. ^1H NMR (500.13 MHz, $CDCl_3$)δ: 4.22 (s, 3H, OMe), 7.65–7.73 (m, 3H, H(4,5,6)), 7.80 (d, 1H, H(3), $^3J_{HH}$ = 7.7 Hz) ppm. ^{13}C NMR (125.76 MHz, $CDCl_3$)δ: 62.0 (s, OMe), 126.1 (s, C(6)), 127.6 (q, C(3), $^3J_{CF}$ = 4.7 Hz), 131.0 (s, C(4)), 133.0 (C(5)), 141.0 (br. s, C(1)) ppm. The ^1H–^{13}C HSQC and HMBC experiments were used to assign the signals. ^{14}N NMR (36.14 MHz, $CDCl_3$)δ: –70 (<u>N</u>(O)=N–OMe, $\Delta\nu_{½}$ = 150 Hz) ppm. ^{19}F NMR (470.59 MHz, $CDCl_3$): –60.4 (CF_3) ppm. IR (KBr): ν = 2953 (w), 1730 (w), 1460 (m), 1332 (s), 1272 (m), 1170 (m), 1125 (s), 1048 (s) cm^{-1}. HRMS (ESI): m/z calcd for $C_8H_7F_3N_2O_2$ $[M+H]^+$ 221.0532; found 221.0531. Elemental analysis calcd (%) for $C_8H_7F_3N_2O_2$: C 43.65, H, 3.21, N 12.72; found: C 43.70, H 3.25, N 12.65.

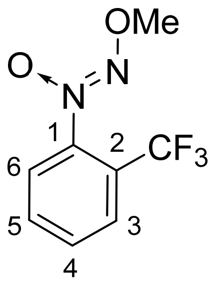

1-(Methoxy-*NNO*-azoxy)-3-(trifluoromethyl)benzene (2k): Pale-yellow oil. R_f (petroleum ether/ethyl acetate, 5:1) = 0.25. ^1H NMR (500.13 MHz, $CDCl_3$)δ: 4.28 (s, 3H, OMe), 7.65 (t, 1H, H(5), $^3J_{HH}$ = 8.0 Hz), 7.80 (d, 1H, H(4), $^3J_{HH}$ = 7.8 Hz), 8.23 (d, 1H, H(6), $^3J_{HH}$ = 8.3 Hz), 8.30 (s, 1H, H(2)) ppm. ^{13}C NMR (125.76 MHz, $CDCl_3$)δ: 62.3 (s, OMe), 118.5 (q, C(2), $^3J_{CF}$ = 3.9 Hz), 124.4 (C(6)), 128.0 (q, C(4), $^3J_{CF}$ = 3.6 Hz), 129.9 (s, C(5)), 131.8 (q, C(3), $^2J_{CF}$ = 33.6 Hz), 143.3 (br. s, C(1)) ppm. The ^1H–^{13}C HSQC and HMBC experiments were used to assign the signals. ^{14}N NMR (36.14 MHz, $CDCl_3$)δ: –71 (*N*(O)=N–OMe, $\Delta\nu_{½}$ = 105 Hz) ppm. ^{19}F NMR (470.59 MHz, $CDCl_3$): –63.3 (CF_3) ppm. IR (KBr): ν = 2951 (w), 1727 (w), 1465 (m), 1332 (s), 1274 (m), 1174 (m), 1128 (s), 1049 (s) cm^{-1}. HRMS (ESI): m/z calcd for $C_8H_7F_3N_2O_2$ $[M+H]^+$ 221.0532; found 221.0530. Elemental analysis calcd (%) for $C_8H_7F_3N_2O_2$: C 43.65, H, 3.21, N 12.72; found: C 43.68, H 3.22, N 12.68.

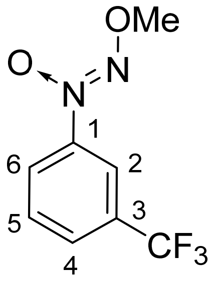

2-(Methoxy-*NNO*-azoxy)-2-nitropropane (2l): Light yellow crystals, m.p. 49–50 °C (lit. m.p. 49.5–50.5 °C).[23] R_f (petroleum ether/ethyl acetate, 3:1) = 0.30. ^1H NMR (500.13 MHz, $CDCl_3$)δ: 2.15 (s, 6H, Me), 4.19 (s, 3H, OMe) ppm. ^{13}C NMR (125.76 MHz, $CDCl_3$)δ: 24.2 (s, Me), 62.3 (s, OMe), 108.0 (br. s, C(2)) ppm. The ^1H–^{13}C HSQC and HMBC experiments were used to assign the signals. ^{14}N NMR (36.14 MHz, $CDCl_3$)δ: 0 (NO_2, $\Delta\nu_{½}$ = 75 Hz), –66 (N(O)=N–OMe, $\Delta\nu_{½}$ = 90 Hz) ppm. IR (KBr): ν = 1638 (s), 1581 (s), 1515 (m), 1384 (w), 1275 (m), 1104 (m), 1056 (w), 843 (m), 813 (s) cm^{-1}. HRMS (ESI): m/z calcd for $C_4H_9N_3O_4$ $[M+H]^+$

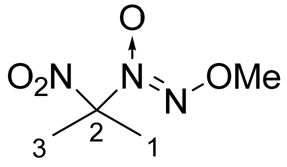

164.0666; found 164.0669. Elemental analysis calcd (%) for $C_4H_9N_3O_4$: C 29.45, H 5.56, N 25.76; found: C 29.47, H 5.57, N 25.74.

1-(Methoxy-*NNO*-azoxy)-1-nitrocyclopentane (2m): Yellow oil. R_f (petroleum ether/ethyl acetate, 3:1) = 0.20. ^1H NMR (600.13 MHz, $CDCl_3$)δ: 1.90–2.02 (m, 4H, H(2,5)), 2.73–2.89 (m, 4H, H(3,4)), 4.19 (s, 3H, OMe) ppm. ^{13}C NMR (150.90 MHz, $CDCl_3$)δ: 23.9 (s, C(2, 5)), 36.3 (s, C(3,4)), 62.3 (s, OMe), 117.3 (br. s, C(1)) ppm. The ^1H–^{13}C HSQC and HMBC experiments were used to assign the signals. ^{14}N NMR (43.37 MHz, $CDCl_3$)δ: 0 (NO_2, $\Delta\nu_{½}$ = 75 Hz), –68 (<u>N</u>(O)=N–OMe, $\Delta\nu_{½}$ = 100 Hz) ppm. IR (KBr): ν = 2955 (m), 2882 (w), 1736 (m), 1566 (s), 1477 (m), 1357 (m), 1045 (s) cm^{-1}. HRMS (ESI): m/z calcd for $C_6H_{11}N_3O_4$ $[M+H]^+$ 190.0822; found 190.0820. Elemental analysis calcd (%) for $C_6H_{11}N_3O_4$: C 38.10, H 5.86, N 22.21; found: C 38.13, H 5.88, N 22.15.

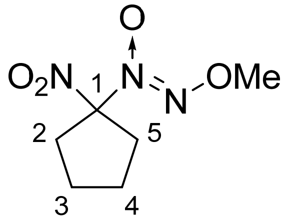

1-(Methoxy-*NNO*-azoxy)-1-nitrocyclohexane (2n): Yellow crystals, m.p. 139–141 °C (lit. m.p. 141–142 °C).[23] R_f (petroleum ether/ethyl acetate, 3:1) = 0.45. ^1H NMR (600.13 MHz, $CDCl_3$)δ: 1.52, 1.54 (m, 1H, H(4)), 1.61, 1.62 (m, 3H, H(3,4,5)), 1.77 (m, 2H, H(3,5)), 2.48–2.72 (m, 4H, H(2,6)), 4.19 (s, 3H, OMe) ppm. ^{13}C NMR (150.90 MHz, $CDCl_3$)δ: 22.4 (s, C(3,5)), 23.8 (s, C(4)), 32.4 (s, C(2,6)), 62.3 (s, OMe), 111.0 (br. s, C(1)) ppm. The ^1H–^{13}C HSQC experiment was used to assign the signals. ^{14}N NMR (43.37 MHz, $CDCl_3$)δ: 0 (NO_2, $\Delta\nu_{½}$ = 100 Hz), –70 (<u>N</u>(O)=N–OMe, $\Delta\nu_{½}$ = 125 Hz) ppm. IR (KBr): ν = 2955 (m), 2877 (m), 2864 (m), 1564 (s), 1482 (m), 1453 (m), 1372 (m), 1333 (m), 1248 (m), 1059 (s), 1033 (m) cm^{-1}. HRMS (ESI): m/z calcd for $C_7H_{13}N_3O_4$ $[M+H]^+$ 204.0979; found 204.0976. Elemental analysis calcd (%) for $C_7H_{13}N_3O_4$: C 41.38, H 6.45, N 20.68; found: C 41.41, H 6.47, N 20.62.

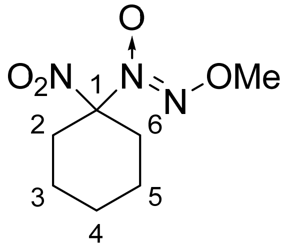

2-(Methoxy-*NNO*-azoxy)-2-nitro-1,3-diphenylpropane (2o): pale yellow crystals, m.p. 106–108 °C. R_f (petroleum ether/ethyl acetate, 5:1) = 0.40. ^1H NMR (500.13 MHz, $CDCl_3$)δ: 3.67 (s, 4H, CH_2), 4.03 (s, 3H, OMe), 7.16–7.38 (m, 10H, Ph) ppm. ^{13}C NMR (125.76 MHz, $CDCl_3$)δ: 39.4 (s, CH_2), 62.4 (s, OMe), 114.1 (br. s, C(2)), 126.7–136.2 (Ph) ppm. The ^1H–^{13}C HSQC and HMBC experiments were used to assign the signals. ^{14}N NMR (36.14 MHz, $CDCl_3$)δ: –5 (NO_2, $\Delta\nu_{½}$ = 280 Hz), –70 (<u>N</u>(O)=N–OMe, $\Delta\nu_{½}$ = 25 Hz) ppm. IR (KBr): ν = 3252 (w), 3028 (w), 1567 (m), 1495 (m), 1452 (m), 969 (m), 700 (s) cm^{-1}. HRMS (ESI): m/z calcd for $C_{16}H_{17}N_3O_4$ $[M+Na]^+$ 338.1111; found 338.1113. Elemental

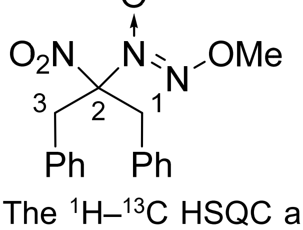

analysis calcd (%) for $C_{16}H_{17}N_3O_4$: C 60.94, H 5.43, N 13.33; found: C 60.99, H 5.48, N 13.23.

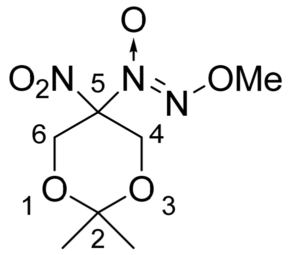

5-(Methoxy-*NNO*-azoxy)-2,2-dimethyl-5-nitro-1,3-dioxane (2p): Light yellow crystals, m.p. 58–60 °C. R_f (petroleum ether/ethyl acetate, 3:1) = 0.30. ^{1}H NMR (500.13 MHz, [D$_6$]acetone)δ: 1.41, 1.49 (s, 6H, Me), 4.15 (s, 3H, OMe), 4.74 (dd, 4H, H(4,6), $^2J_{HH}$ = 13.3 Hz, $^3J_{HH}$ = 3.9 Hz) ppm. ^{13}C NMR (125.76 MHz, [D$_6$]acetone)δ: 21.4, 25.3 (s, Me), 62.1 (s, C(4,6)), 62.8 (s, OMe), 100.9 (s, C(2)), 102.4 (br. s, C(5)) ppm. The ^{1}H–^{13}C HSQC and HMBC experiments were used to assign the signals. ^{14}N NMR (36.14 MHz, [D$_6$]acetone)δ: 8 (N(O)=<u>N</u>–OMe, $\Delta\nu_{½}$ = > 1000 Hz), –9 (NO_2, $\Delta\nu_{½}$ = 65 Hz), –75 ((<u>N</u>(O)=N–OMe, $\Delta\nu_{½}$ = 80 Hz) ppm. IR (KBr): ν = 2998 (w), 2952 (w), 1575 (s), 1477 (m), 1381 (m), 1202 (m), 1106 (m), 1052 (m) cm^{-1}. HRMS (ESI): m/z calcd for $C_7H_{13}N_3O_6$ [M+H]$^+$ 236.0877; found 236.0877. Elemental analysis calcd (%) for $C_7H_{13}N_3O_6$: C 35.75, H 5.57, N 17.87; found: C 35.79, H 5.60, N 17.81.

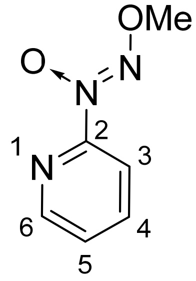

2-(Methoxy-*NNO*-azoxy)pyridine (2q): Pale yellow oil that freezes in the cold. R_f (petroleum ether/ethyl acetate, 1:1) = 0.25. ^{1}H NMR (500.13 MHz, $CDCl_3$)δ: 4.33 (s, 3H, OMe), 7.57 (t, 1H, H(5), $^3J_{HH}$ = 5.8 Hz), 8.00 (t, 1H, H(4), $^3J_{HH}$ = 7.8 Hz), 8.09 (d, 1H, H(3), $^3J_{HH}$ = 8.1 Hz), 8.60 (d, 1H, H(6), $^3J_{HH}$ = 3.5 Hz) ppm. ^{13}C NMR (125.76 MHz, $CDCl_3$)δ: 61.8 (s, OMe), 116.0 (s, C(5)), 126.2 (s, C(3)), 138.9 (s, C(4)), 147.9 (s, C(6)), 153.2 (br. s, C(2)) ppm. The ^{1}H–^{13}C HSQC and HMBC experiments were used to assign the signals. ^{14}N NMR (36.14 MHz, $CDCl_3$)δ: –67 (<u>N</u>(O)=N–OMe, $\Delta\nu_{½}$ = 115 Hz) ppm. IR (KBr): ν = 2948 (w), 1595 (m), 1476 (s), 1442 (s), 1153 (m), 1039 (s) cm^{-1}. HRMS (ESI): m/z calcd for $C_6H_7N_3O_2$ [M+H]$^+$ 154.0611; found 154.0613. Elemental analysis calcd (%) for $C_6H_7N_3O_2$: C 47.06, H 4.61, N 27.44; found: C 47.09, H 4.59, N 27.38.

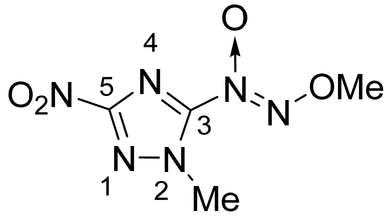

3-(Methoxy-*NNO*-azoxy)-2-methyl-5-nitro-2*H*-1,2,4-triazole (2r): Reddish solid, m.p. 102–104 °C. R_f (petroleum ether/ethyl acetate, 3:1) = 0.25. ^{1}H NMR (500.13 MHz, [D$_6$]acetone)δ: 4.31 (s, 3H, NMe or OMe), 4.32 (s, 3H, NMe or OMe) ppm. ^{13}C NMR (125.76 MHz, [D$_6$]acetone)δ: 40.4 (s, NMe), 63.5 (s, OMe), 148.6 (br. s, C(3)), 159.2 (br. s, C(5)) ppm. The ^{1}H–^{13}C HSQC and HMBC experiments were used to assign the signals. ^{14}N NMR (36.14 MHz, [D$_6$]acetone)δ: –29 (NO_2, $\Delta\nu_{½}$ = 50 Hz), –85 (<u>N</u>(O)=N–OMe, $\Delta\nu_{½}$ = 75 Hz) ppm. IR (KBr): ν = 2957 (w), 1569 (s), 1424 (s),

1317 (s), 1061 (s) cm^{-1}. HRMS (ESI): m/z calcd for $C_4H_6N_6O_4$ $[M+Na]^+$ 225.0343; found 225.0349. Elemental analysis calcd (%) for $C_4H_6N_6O_4$: C 23.77, H 2.99, N 41.58; found: C 23.81, H 3.05, N 41.49.

5-(Methoxy-*NNO*-azoxy)-2-methyl-2*H*-tetrazole (2s): White crystals, m.p. 110–111 °C.

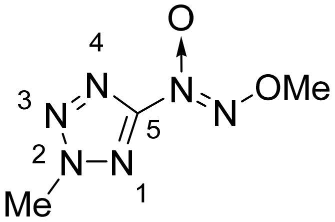

R_f (petroleum ether/ethyl acetate, 1:1) = 0.50. ^{1}H NMR (600.13 MHz, [D$_6$]acetone)δ: 4.26 (s, 3H, OMe), 4.56 (s, 3H, NMe) ppm. ^{13}C NMR (150.90 MHz, [D$_6$]acetone)δ: 41.3 (s, NMe), 62.9 (s, OMe), 163.7 (br. s, C(5)) ppm. The ^{1}H–^{13}C HSQC experiment was used to assign the signals. ^{14}N NMR (43.37 MHz, [D$_6$]acetone)δ: 5 (N(3) or N(O)=<u>N</u>–OMe, $\Delta\nu_{½}$ = 850 Hz), –61 (N(4), $\Delta\nu_{½}$ = 500 Hz), –84 (<u>N</u>(O)=N–OMe, $\Delta\nu_{½}$ = 50 Hz), –101 (N(2), $\Delta\nu_{½}$ = 135 Hz) ppm. IR (KBr): ν = 2961 (w), 1503 (m), 1472 (m), 1388 (s), 1249 (m), 1199 (m), 1082 (s), 1052 (s), 1018 (s) cm^{-1}. HRMS (ESI): m/z calcd for $C_3H_6N_6O_2$ $[M+H]^+$ 159.0625; found 159.0624. Elemental analysis calcd (%) for $C_3H_6N_6O_2$: C 22.79, H 3.82, N 53.15; found: C 22.80, H 3.81, N 53.11.

4. Pictures of the Equipment Used

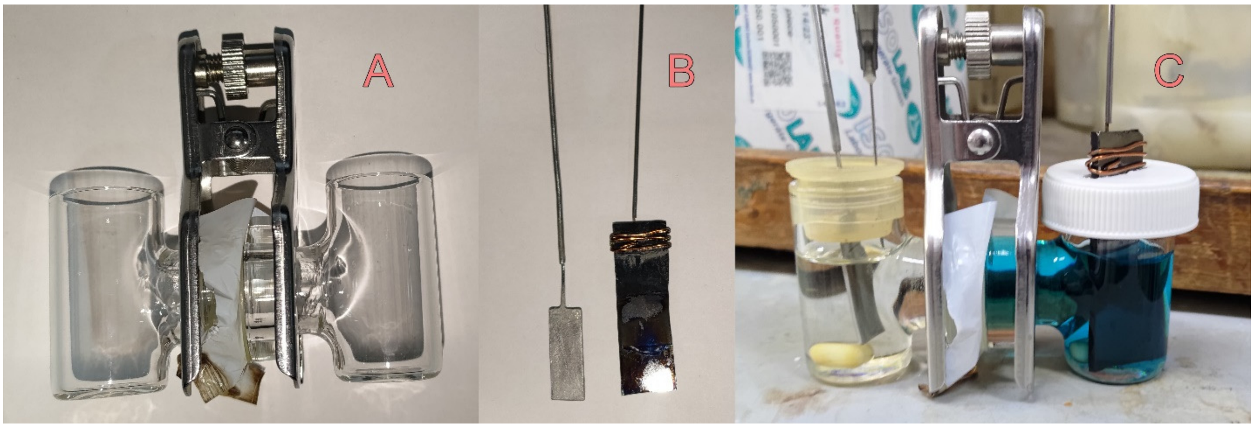

Figure S5. (A) Divided electrochemical cell with Celgard® 2400 membrane. (B) Electrodes used in the reaction – stainless steel (left) and glassy carbon (right). (C) Assembled electrochemical cell.

5. X-ray single-crystal diffraction: Structure determination of compounds 2c, 2d, 2p and 2s

X-ray diffraction data were collected at 100K on a four-circle Rigaku Synergy S diffractometer equipped with a HyPix6000HE area-detector (kappa geometry, shutterless ω-scan technique), using monochromatized Cu K_α-radiation. The intensity data were integrated and corrected for absorption and decay by the CrysAlisPro program.[24] The

structure was solved by direct methods using SHELXT[24] and refined on F^2 using SHELXL-2018[26] in the OLEX2 program.[27] All non-hydrogen atoms were refined with individual anisotropic displacement parameters. All hydrogen atoms were placed in ideal calculated positions and refined as riding atoms with relative isotropic displacement parameters. A rotating group model was applied for methyl groups.

Table S3. Crystal data and structure refinement for **2c**.

Empirical formula	$C_7H_7N_3O_4$	
Formula weight	197.16	
Temperature	100.0 K	
Wavelength	1.54184 Å	
Crystal system	Triclinic	
Space group	P-1	
Unit cell dimensions	a = 6.7389(2) Å	α = 83.090(2)°
	b = 10.6987(2) Å	β = 74.600(2)°
	c = 12.2035(3) Å	γ = 77.281(2)°
Volume	825.65(4) $Å^3$	
Z	4	
Density (calculated)	1.586 g/cm^3	
Absorption coefficient	1.148 mm^{-1}	
F(000)	408	
Crystal size	0.015 x 0.014 x 0.012 mm^3	
Theta range for data collection	3.765 to 80.045°	
Index ranges	-8<=h<=8, -13<=k<=13, -15<=l<=14	
Reflections collected	21948	
Independent reflections	3588 [R(int) = 0.0302]	
Completeness to theta = 67.684°	99.9 %	
Absorption correction	Semi-empirical from equivalents	
Max. and min. transmission	1.00000 and 0.96715	
Refinement method	Full-matrix least-squares on F^2	

Data / restraints / parameters	3588 / 0 / 256	
Goodness-of-fit on F^2	1.109	
Final R indices [I>2sigma(I)]	R1 = 0.0335, wR2 = 0.0940	
R indices (all data)	R1 = 0.0352, wR2 = 0.0956	
Extinction coefficient	0.0016(4)	
Largest diff. peak and hole	0.320 and -0.184 e.Å^{-3}	

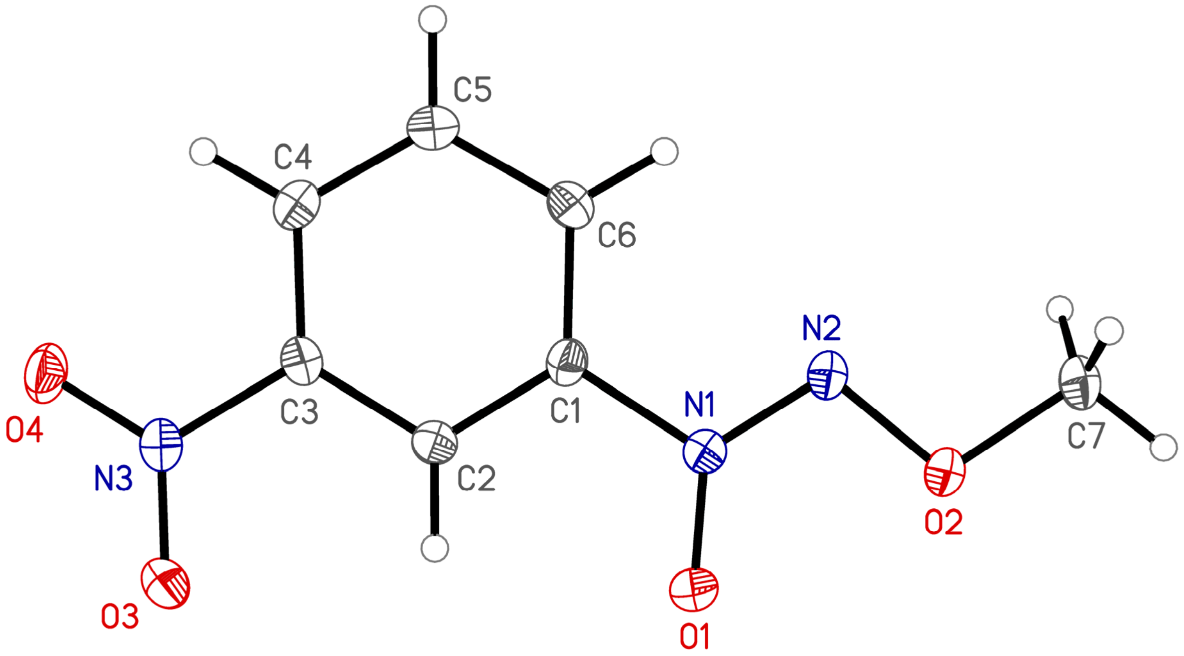

Figure S6. Crystal structure of compound **2c**, showing the atomic numbering and 50% probability displacement ellipsoids

Table S4. Crystal data and structure refinement for **2d**.

Empirical formula	$C_7H_7N_3O_4$	
Formula weight	197.16	
Temperature	100.15 K	
Wavelength	1.54184 Å	
Crystal system	Monoclinic	
Space group	P 2_1/c	
Unit cell dimensions	a = 3.77533(5) Å	α = 90°
	b = 22.0876(3) Å	β = 91.9671(11)°
	c = 10.21955(12) Å	γ = 90°

Volume	851.685(19) $Å^3$	
Z	4	
Density (calculated)	1.538 g/cm^3	
Absorption coefficient	1.113 mm^{-1}	
F(000)	408	
Crystal size	0.35 x 0.08 x 0.06 mm^3	
Theta range for data collection	4.003 to 79.935°	
Index ranges	-4<=h<=4, -28<=k<=27, -12<=l<=13	
Reflections collected	9545	
Independent reflections	1842 [R(int) = 0.0251]	
Completeness to theta = 67.684°	99.8 %	
Absorption correction	Gaussian	
Max. and min. transmission	1.000 and 0.480	
Refinement method	Full-matrix least-squares on F^2	
Data / restraints / parameters	1842 / 0 / 128	
Goodness-of-fit on F^2	1.088	
Final R indices [I>2sigma(I)]	R1 = 0.0353, wR2 = 0.0974	
R indices (all data)	R1 = 0.0370, wR2 = 0.0989	
Extinction coefficient	0.0016(4)	
Largest diff. peak and hole	0.266 and -0.248 $e.Å^{-3}$	

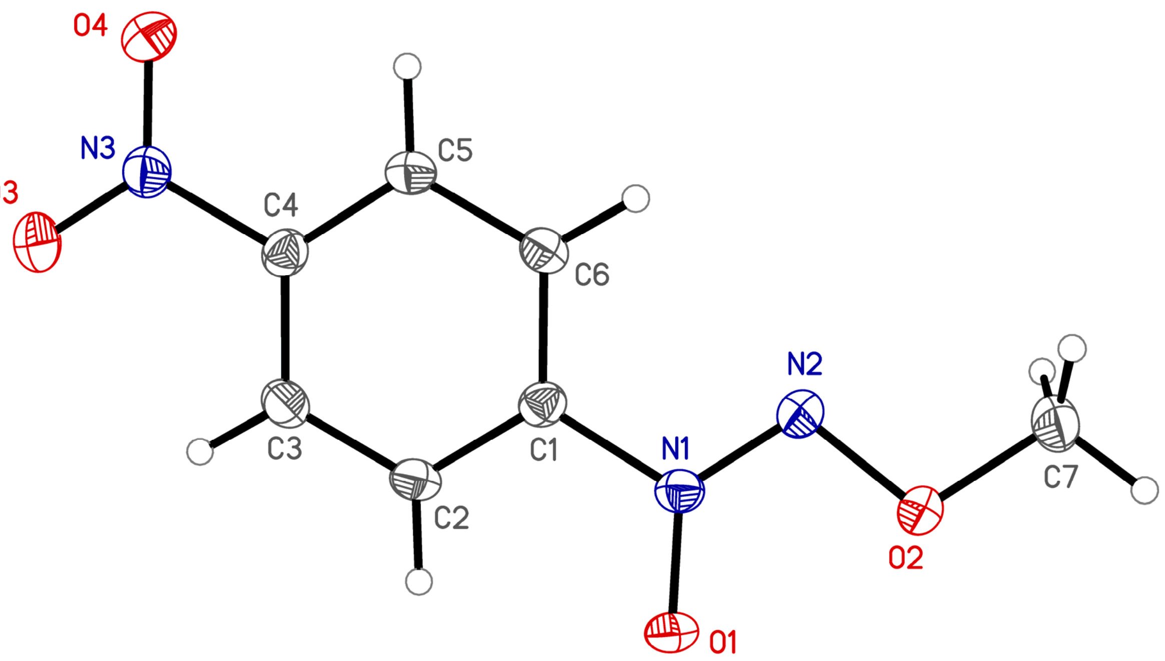

Figure S7. Crystal structure of compound **2d**, showing the atomic numbering and 50% probability displacement ellipsoids

Table S5. Crystal data and structure refinement for **2p**.

Empirical formula	$C_7H_{13}N_3O_6$	
Formula weight	235.20	
Temperature	100.15 K	
Wavelength	1.54184 Å	
Crystal system	Orthorhombic	
Space group	Pbca	
Unit cell dimensions	a = 10.88190(10) Å	α = 90°
	b = 8.34380(10) Å	β = 90°
	c = 23.7635(2) Å	γ = 90°
Volume	2157.64(4) Å^3	
Z	8	
Density (calculated)	1.448 g/cm^3	
Absorption coefficient	1.105 mm^{-1}	
F(000)	992	
Crystal size	0.53 x 0.27 x 0.12 mm^3	

Theta range for data collection	3.720 to 80.761°	
Index ranges	-10<=h<=13, -10<=k<=10, -30<=l<=30	
Reflections collected	15575	
Independent reflections	2363 [R(int) = 0.0320]	
Completeness to theta = 67.684°	100.0 %	
Absorption correction	Gaussian	
Max. and min. transmission	1.00000 and 0.552	
Refinement method	Full-matrix least-squares on F^2	
Data / restraints / parameters	2363 / 0 / 148	
Goodness-of-fit on F^2	1.082	
Final R indices [I>2sigma(I)]	R1 = 0.0350, wR2 = 0.0906	
R indices (all data)	R1 = 0.0368, wR2 = 0.0919	
Extinction coefficient	n/a	
Largest diff. peak and hole	0.294 and -0.240 e.Å^{-3}	

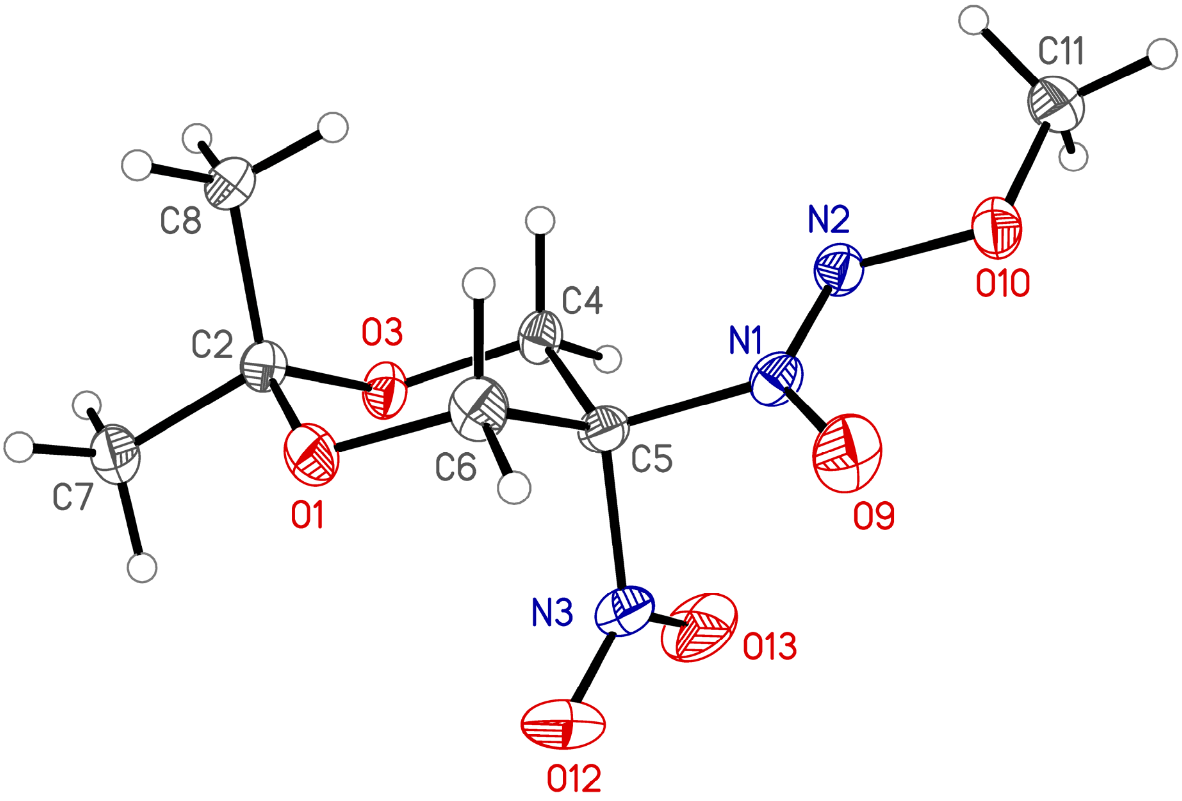

Figure S8. Crystal structure of compound **2p**, showing the atomic numbering and 50% probability displacement ellipsoids

Table S6. Crystal data and structure refinement for **2s**.

Empirical formula	$C_3H_6N_6O_2$	
Formula weight	158.14	
Temperature	100.01(10) K	
Wavelength	1.54184 Å	
Crystal system	Orthorhombic	
Space group	Pnma	
Unit cell dimensions	a = 13.2055(2) Å	α = 90°
	b = 6.21300(10) Å	β = 90°
	c = 8.27330(10) Å	γ = 90°
Volume	678.789(17) $Å^3$	
Z	4	
Density (calculated)	1.547 g/cm^3	
Absorption coefficient	1.131 mm^{-1}	
F(000)	328	
Crystal size	0.37 x 0.15 x 0.12 mm^3	
Theta range for data collection	6.313 to 79.708°	
Index ranges	-16<=h<=15, -7<=k<=6, -10<=l<=10	
Reflections collected	5092	
Independent reflections	802 [R(int) = 0.0274]	
Completeness to theta = 67.684°	100.0 %	
Absorption correction	Gaussian	
Max. and min. transmission	1.000 and 0.520	
Refinement method	Full-matrix least-squares on F^2	
Data / restraints / parameters	802 / 0 / 70	
Goodness-of-fit on F^2	1.091	
Final R indices [I>2sigma(I)]	R1 = 0.0333, wR2 = 0.0870	

R indices (all data)	R1 = 0.0348, wR2 = 0.0884	
Extinction coefficient	0.0050(6)	
Largest diff. peak and hole	0.282 and -0.225 e.Å^{-3}	

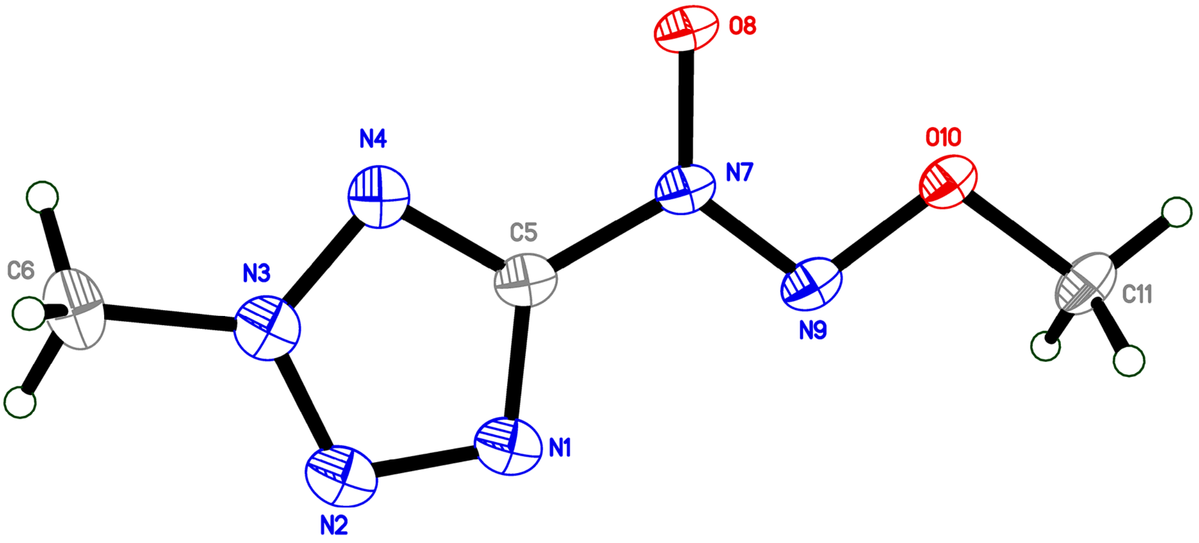

Figure S9. Crystal structure of compound **2s**, showing the atomic numbering and 50% probability displacement ellipsoids

6. References

[1] Coleman, G. H., McCloskey, C. M., Stuart, F. A. Nitrosobenzene. *Organic Syntheses*. **1945**, *25*, 80–83. DOI: 10.15227/orgsyn.025.0080.

[2] Roscales, S., Csaky, A. G., Synthesis of Di(hetero)arylamines from Nitrosoarenes and Boronic Acids: A General, Mild, and Transition-Metal-Free Coupling. *Org. Lett.* **2018**, *20* (6), 1667–1671. DOI: 10.1021/acs.orglett.8b00473.

[3] Dudek, M., Pakladek, Z., Deiana, M., Matcztszyn, K. Molecular design and structural characterization of photoresponsive azobenzene-based polyamide units. *Dyes Pigm.* **2020**, *180*, 108501. DOI: 10.1016/j.dyepig.2020.108501.

[4] Halasz, I., Biljan, I., Novak, P., Mestrovic, E., Plavec, J., Mali, G., Smrecki, V., Vancik, H., Cross-dimerization of nitrosobenzenes in solution and in solid state. *J. Mol. Struct.* **2009**, *918* (1), 19–25. DOI: 10.1016/j.molstruc.2008.07.035.

[5] Teders, M., Pogodaev, A. A.; Bojanov, G., Huck, W. T. S. Reversible Photoswitchable Inhibitors Generate Ultrasensitivity in Out-of-Equilibrium Enzymatic Reactions. *J. Am. Chem. Soc*. **2021**, 143 (15), p. 5709–5716. DOI:10.1021/jacs.0c12956.

[6] Yanagisawa, A., Lin, Y., Takeishi, A., Yoshida, K. Enantioselective Nitroso Aldol Reaction Catalyzed by a Chiral Phosphine–Silver Complex. *Eur. J. Org. Chem.,* **2016**, 2016 (32), 5355–5359. DOI: 10.1002/ejoc.201601143.

[7] Hu, W., Zheng, Q., Sun, S., Cheng, J. Catalyzed bilateral cyclization of aldehydes with nitrosos toward unsymmetrical acridines proceeding with C–H functionalization enabled by a transient directing group. *ChemComm.* **2017**, *53* (46), 6263–6266. DOI: 10.1039/c7cc03006a.

[8] Holmes, R. R., Bayer, R. P. A simple method for the direct oxidation of aromatic amines to nitroso compounds. *J. Am. Chem. Soc.* **1960**, *82*, 3454–3456. doi: 10.1021/ja01498a054.

[9] Tibiletti, F., Simonetti, M., Nicholas, K. M., Palmisano, G., Parravicini, M., Imbesi, F., Tollari, S., Penoni, A. One-pot synthesis of meridianins and meridianin analogues via indolization of nitrosoarenes. *Tetrahedron*. **2010**, *66*, 1280–1288. doi: 10.1016/j.tet.2009.12.020.

[10] Fernández, I. F., Hecquet, L., Fessner, W. Transketolase Catalyzed Synthesis of N-Aryl Hydroxamic Acids. *Adv. Synth. Catal.* **2021**, *364*, 612–621. doi: 10.1002/adsc.202101100.

[11] Charlton, W.; Earl, J. C.; Kenner, J.; Luciano, A. A. The nitration of oximes. *J. Chem. Soc.* **1932**, 30–41. DOI: 10.1039/JR9320000030.

[12] Rehse, K.; Herpel, M. New NO donors with antithrombotic and vasodilating activities, part 19: pseudonitroles and their dimeric azodioxides. *Arch. Pharm. Pharm Med. Chem.* **1998**, *331*, 79–84. DOI: 10.1002/(SICI)1521-4184(199802)331:2%3C79::AID-ARDP79%3E3.0.CO;2-9.

[13] Nametkin, S. S. About cyclohexylpseudonitrol. *Zhurnal Russkago Fiziko-Khimicheskago Obshchestva*. **1910**, *42*, 585–586.

[14] Luk'yanov, O. A.; Salamonov, Yu. B.; Bass, A. G.; Strelenko, Yu. A. N′-(α-acetoximinoalkyl)diazene-N-oxides and some of their transformations. *Russ. Chem. Bull.* **1991**, *40*, 93–98. DOI: 10.1007/BF00959638.

[15] Taylor, E. C.; Tseng, C.-P.; Rampal, J. B. Conversion of a Primary Amino Group into a Nitroso Group. Synthesis of Nitroso-Substituted Heterocycles. *J. Org. Chem.* **1982**, *47*, 552–555. DOI: 10.1021/jo00342a035.

[16] Bagal, L. I.; Pevzner, M. S.; Egorov, A. P.; Samarenko, V. Ya. Heterocyclic Nitro Compounds VI. Reaction of 1-methyl-3,5-dinitro-1,2,4-triazole with hydrazines. *Chem. Heterocycl. Compd.* **1970**, *6*, 928–931. DOI: 10.1007/BF00471694.

[17] Scherschel, N. F.; Zeller, M.; Piercey, D. G. Energetic Azoxy-Coupled Tetrazoles. *J. Heterocycl. Chem.* **2024**, *61*, 1704–1709. DOI: 10.1002/jhet.4881.

[18] Kulikov, A. A.; Leonov, N. E.; Klenov, M. S.; Smirnov, G. A.; Strelenko, Yu. A.; Fedyanin, I. V.; Kon'kova, T. S.; Matyushin, Yu. N.; Pivkina, A. N.; Tartakovsky V. A. First comprehensive study of energetic (methoxy-*NNO*-azoxy)furazans: Novel synthetic route, characterization, and property analysis. *Energ. Mater. Front.* **2025**. *6*, 370–382. DOI: 10.1016/j.enmf.2024.08.007.

[19] Demko, Z. P.; Bartsch, M.; Sharpless, K. B. Primary amides. A general nitrogen source for catalytic asymmetric aminohydroxylation of olefins. *Org. Lett.* **2000**, *2,* 2221–2223. DOI: 10.1021/ol000098m.

[20] Hou, Y.; Xie, W.; Janczuk, A.J.; Wang, P.G. O-Alkylation of Cupferron: Aiming at the Design and Synthesis of Controlled Nitric Oxide Releasing Agents. *J. Org. Chem.* **2000**, *65*, 4333–4337, doi:10.1021/jo000157+.

[21] George, M. V.; Kierstead, R. W.; Wright, GF. The stable alkylation products of organo-nitrosohydroxylamines. *Can. J. Chem.* **1959**, 37, 679–699. DOI: 10.1139/v59-094

[22] Huang, Zh.; Zhang, Y.; Fang, L.; Zhang, Zh.; Lai, Y.; Ding, Y.; Cao, F.; Zhang, J.; Peng, S. Nanometre-sized titanium dioxide-catalyzed reactions of nitric oxide with aliphatic cyclic and aromatic amines. *Chem. Commun.* **2009**, *13*, 1763–1765. DOI: 10.1039/b820535c.

[23] Luk'yanov, O. A.; Smirnov, G. A.; Vasil'ev, A. M. Synthesis of *N'*-methoxydiazene *N*-oxides from methoxyamine and nitroso compounds. *Bull. Acad. Sci. USSR, Div. Chem. Sci.* **1990** 39, 2155–2158. DOI: 10.1007/BF00958278.

[24] CrysAlisPro. Version 1.171.41.106a. Rigaku Oxford Diffraction. **2021**.

[25] Sheldrick, G. M. SHELXT - Integrated space-group and crystal-structure determination. *Acta Cryst.* **2015**, *A71*(1), 3–8. DOI: 10.1107/S2053273314026370.

[26] Sheldrick, G. M. Crystal structure refinement with SHELXL. *Acta Cryst.* **2015**, *C71*(1), 3–8. DOI: 10.1107/S2053229614024218.

[27] Dolomanov, O. V.; Bourhis, L. J.; Gildea, R. J.; Howard, J. A. K.; Puschmann, H. OLEX2: a complete structure solution, refinement and analysis program. *J. Appl. Cryst.* **2009**, *42*, 229–341. DOI: 10.1107/S0021889808042726.

9. NMR Data

9.1.1 ^{1}H NMR spectrum of compound 2a [500.13 MHz, $CDCl_3$]

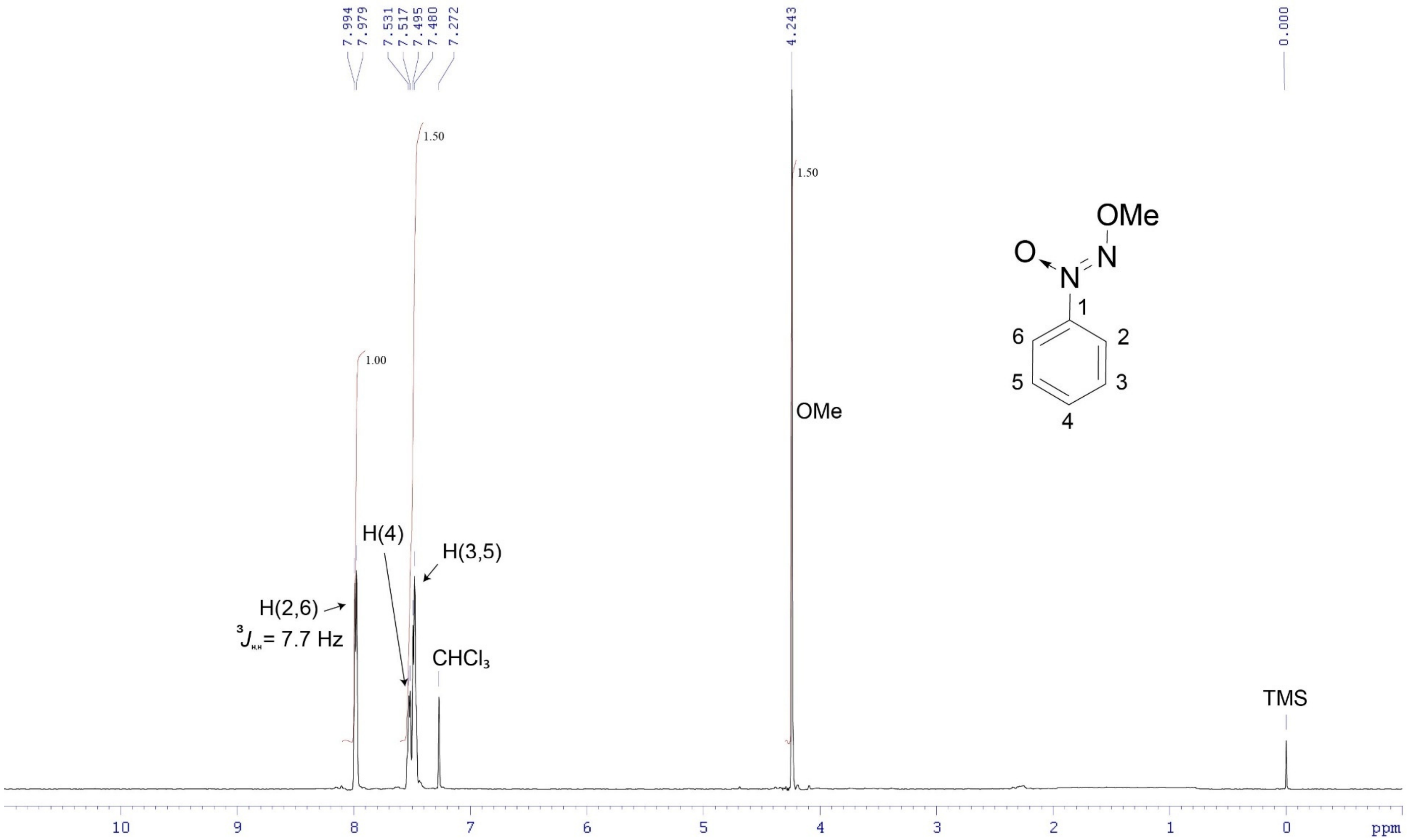

9.1.2 ^{13}C NMR spectrum of compound 2a [125.76 MHz, $CDCl_3$]

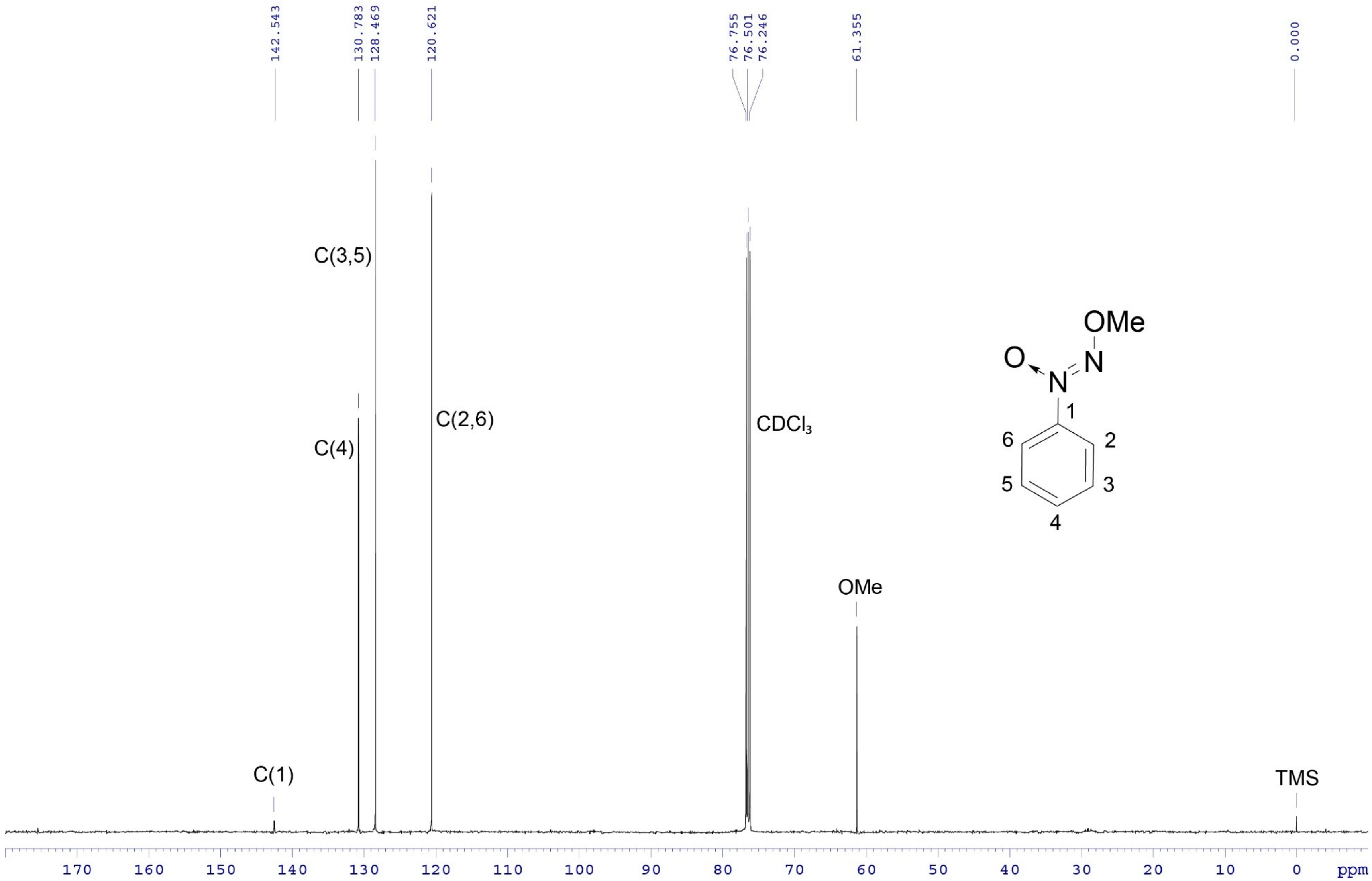

9.1.3 {^1H–^{13}C} HSQC spectrum of compound 2a [500.13 MHz, $CDCl_3$]

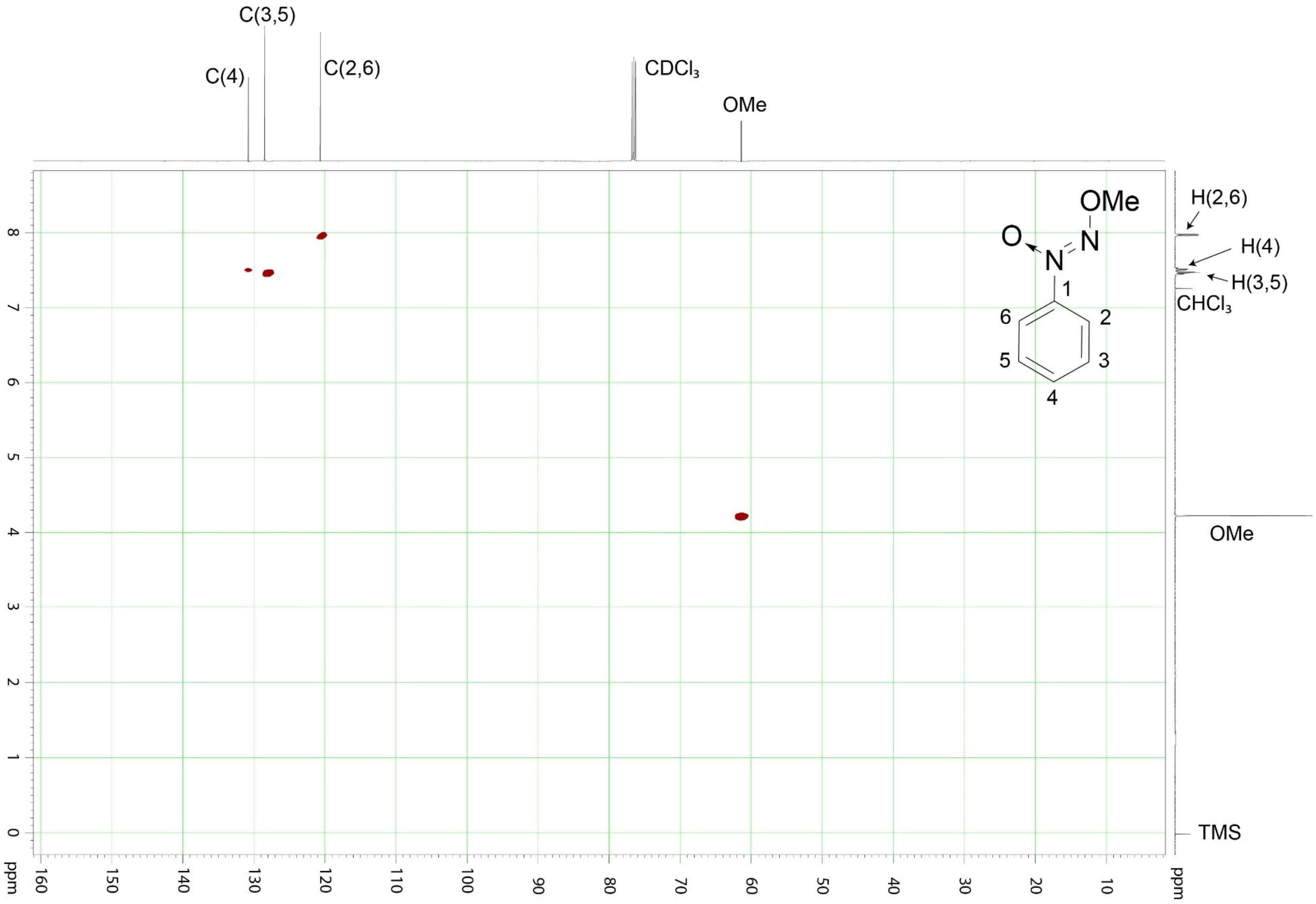

9.1.4 {^{1}H–^{13}C} HMBC spectrum of compound 2a [500.13 MHz, $CDCl_3$]

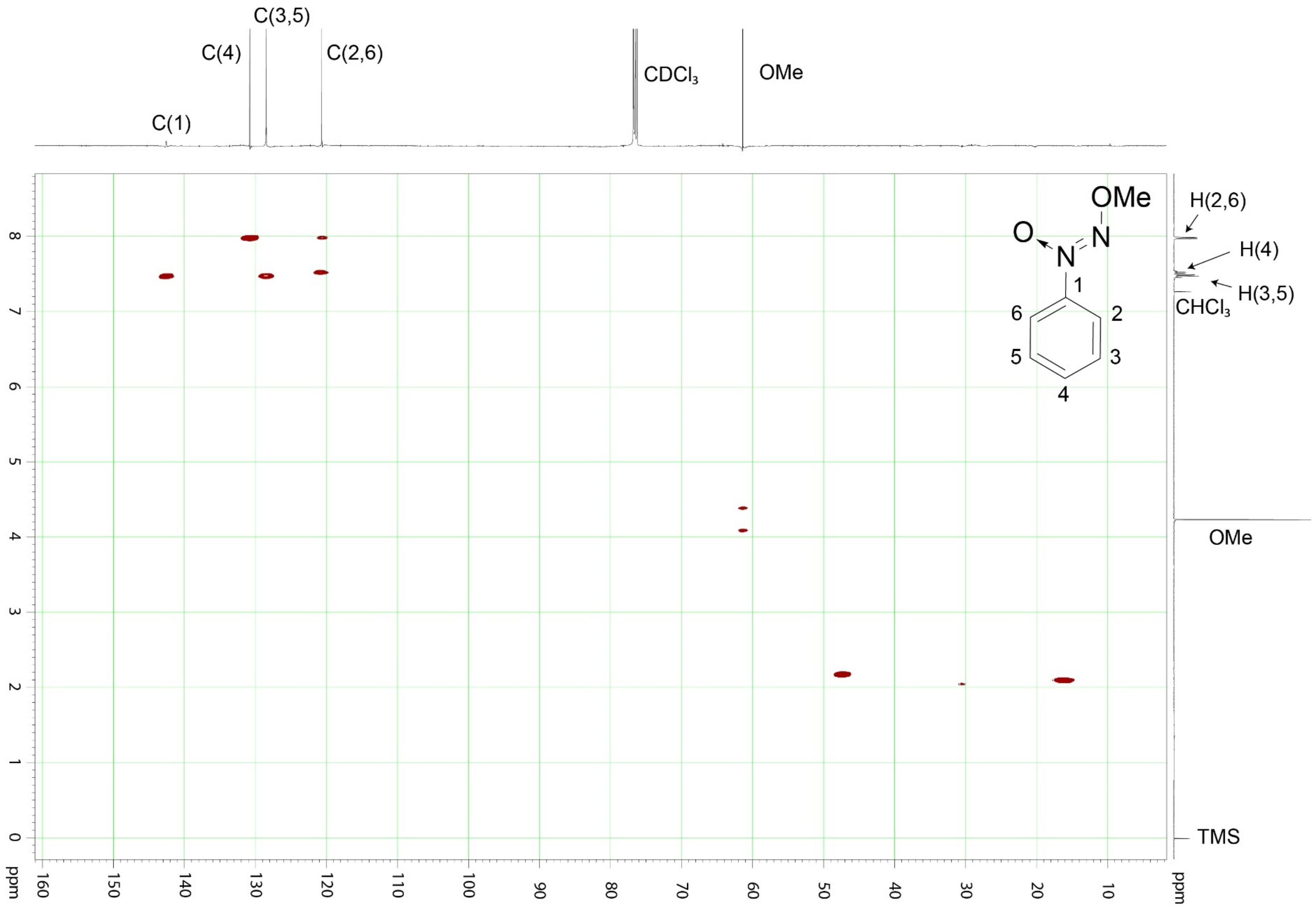

9.1.5 ^{14}N NMR spectrum of compound 2a [36.14 MHz, $CDCl_3$]

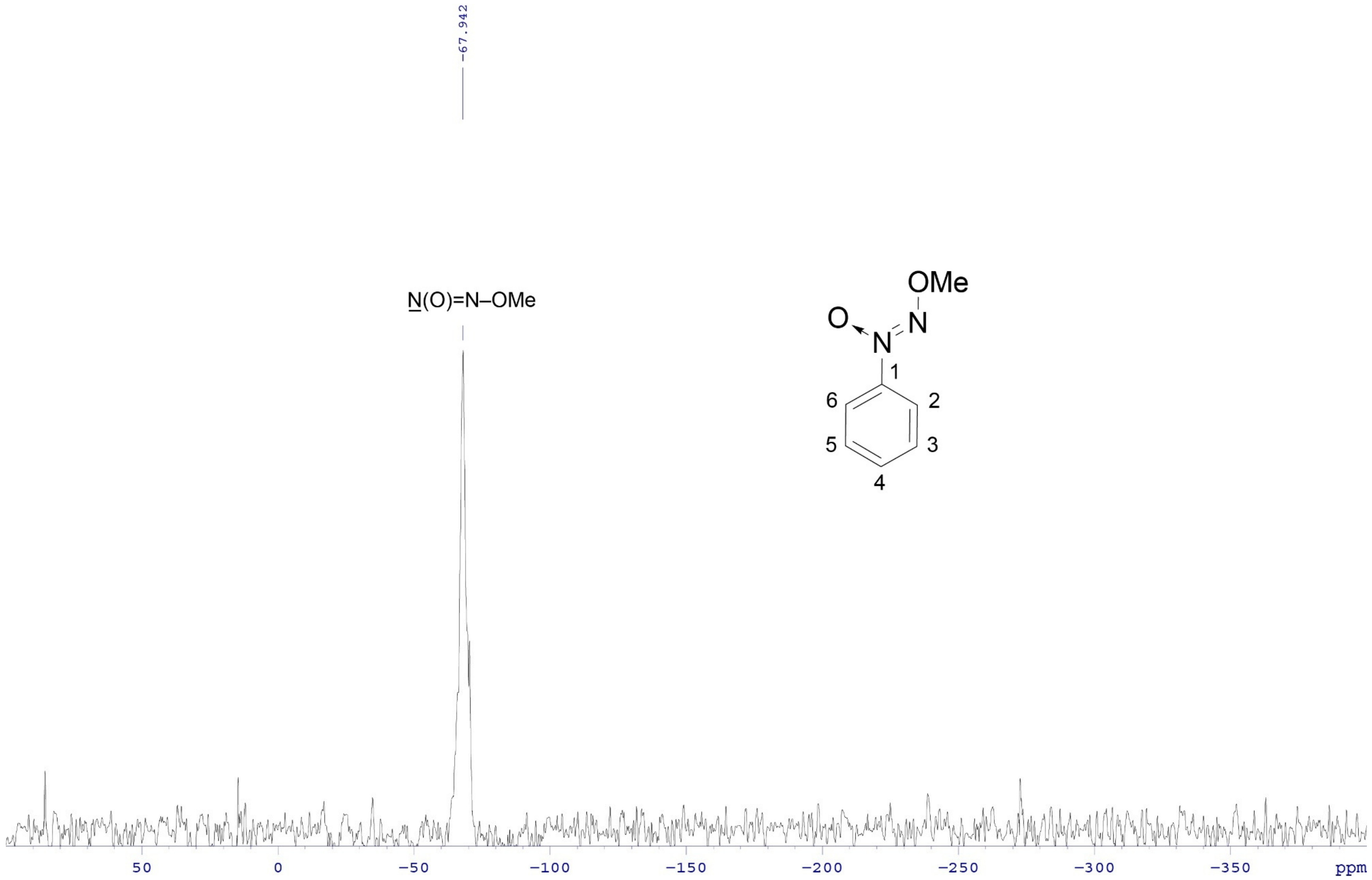

9.2.1 ^{1}H NMR spectrum of compound 2b [500.13 MHz, $CDCl_3$]

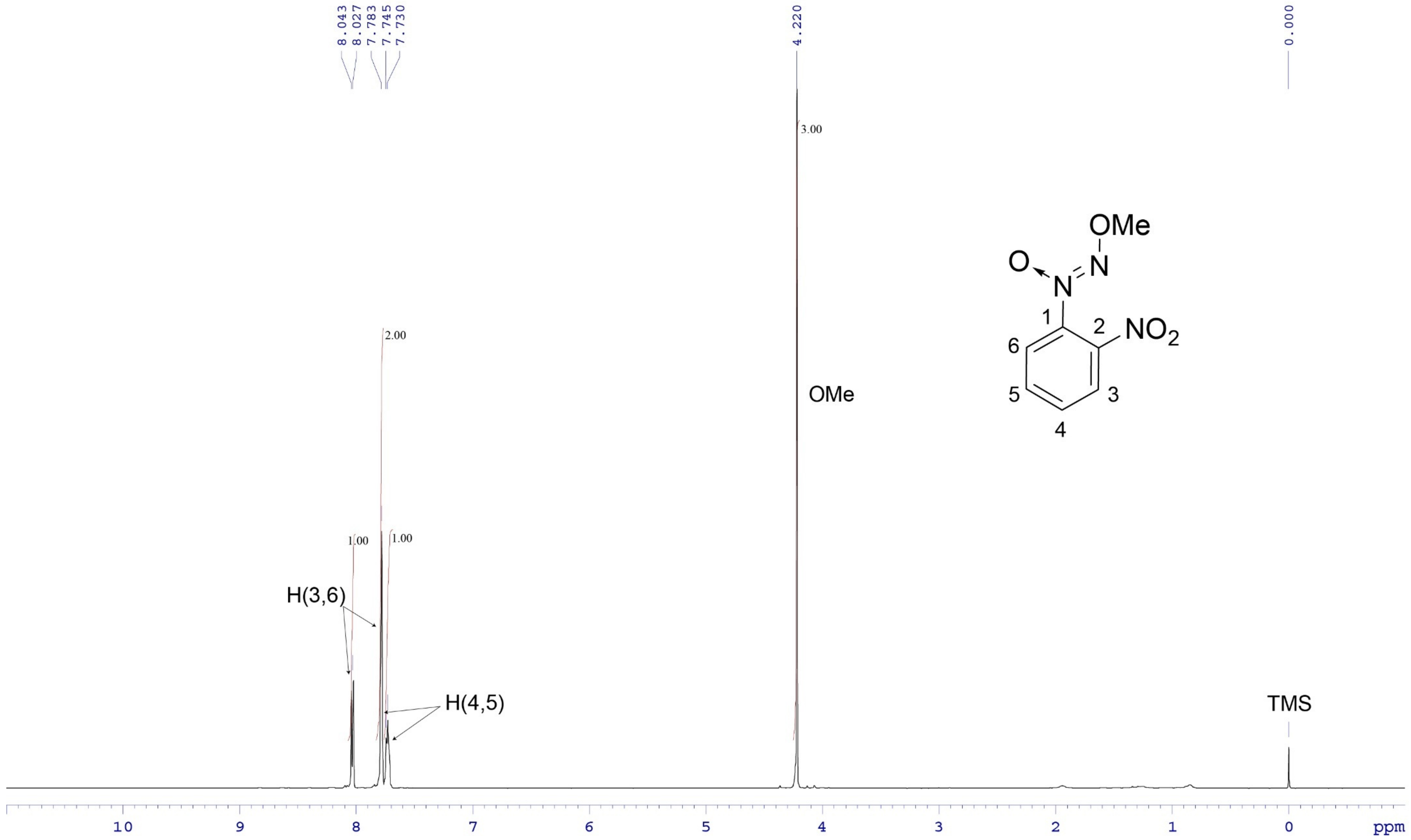

9.2.2 ^{13}C NMR spectrum of compound 2b [125.76 MHz, $CDCl_3$]

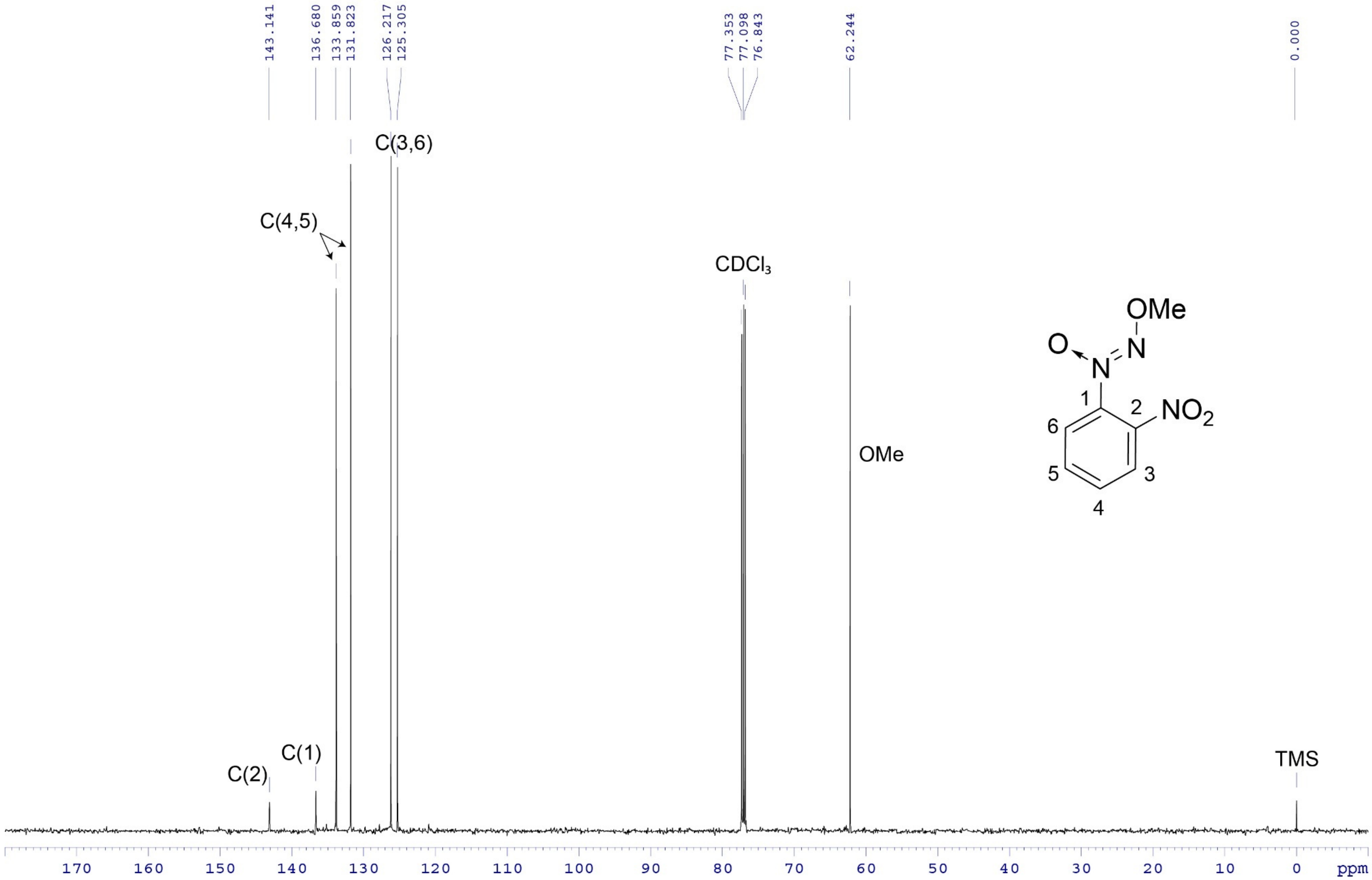

9.2.3 {^{1}H–^{13}C} HSQC spectrum of compound 2b [500.13 MHz, $CDCl_3$]

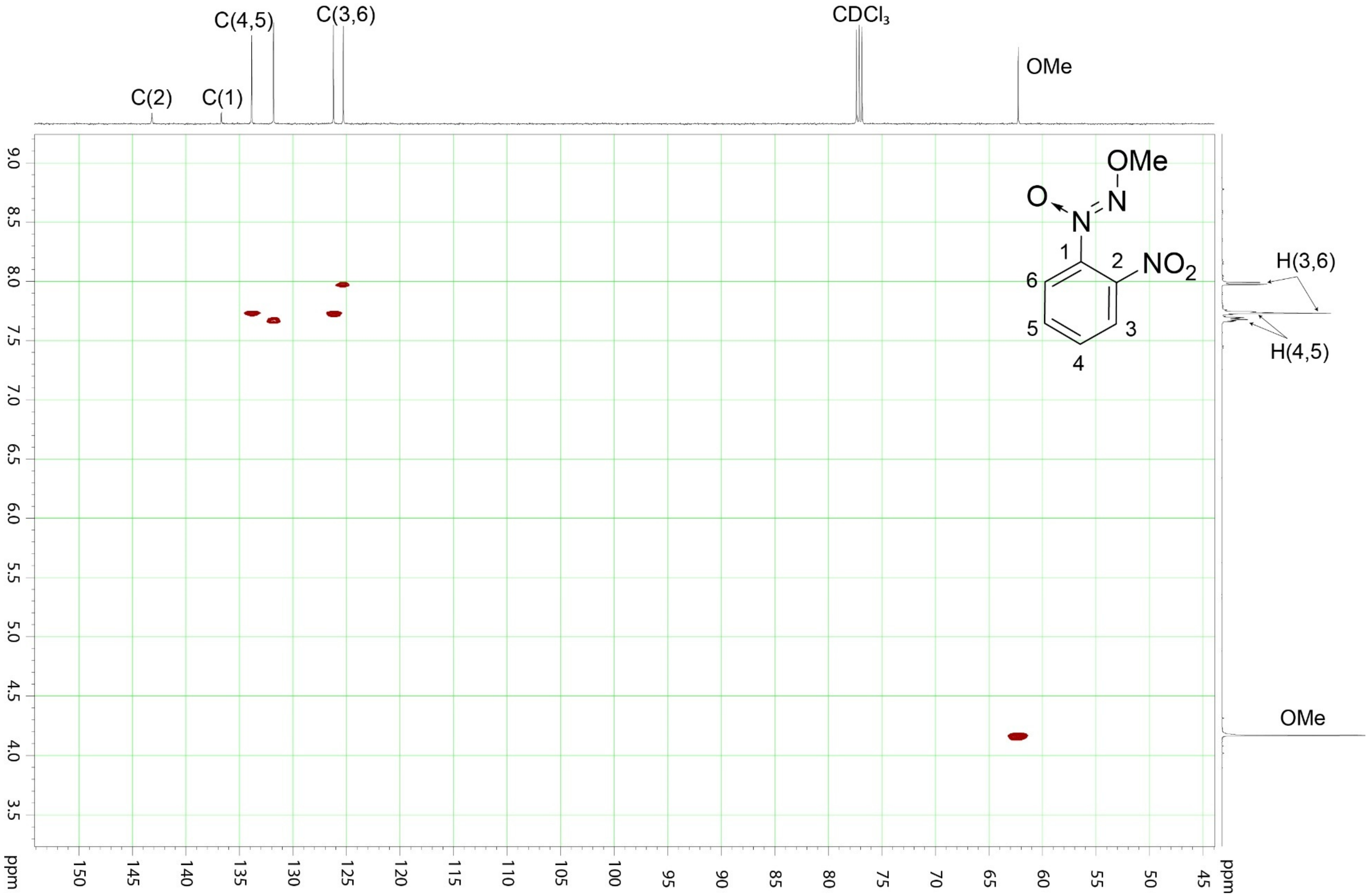

9.2.4 {^{1}H–^{13}C} HMBC spectrum of compound 2b [500.13 MHz, $CDCl_3$]

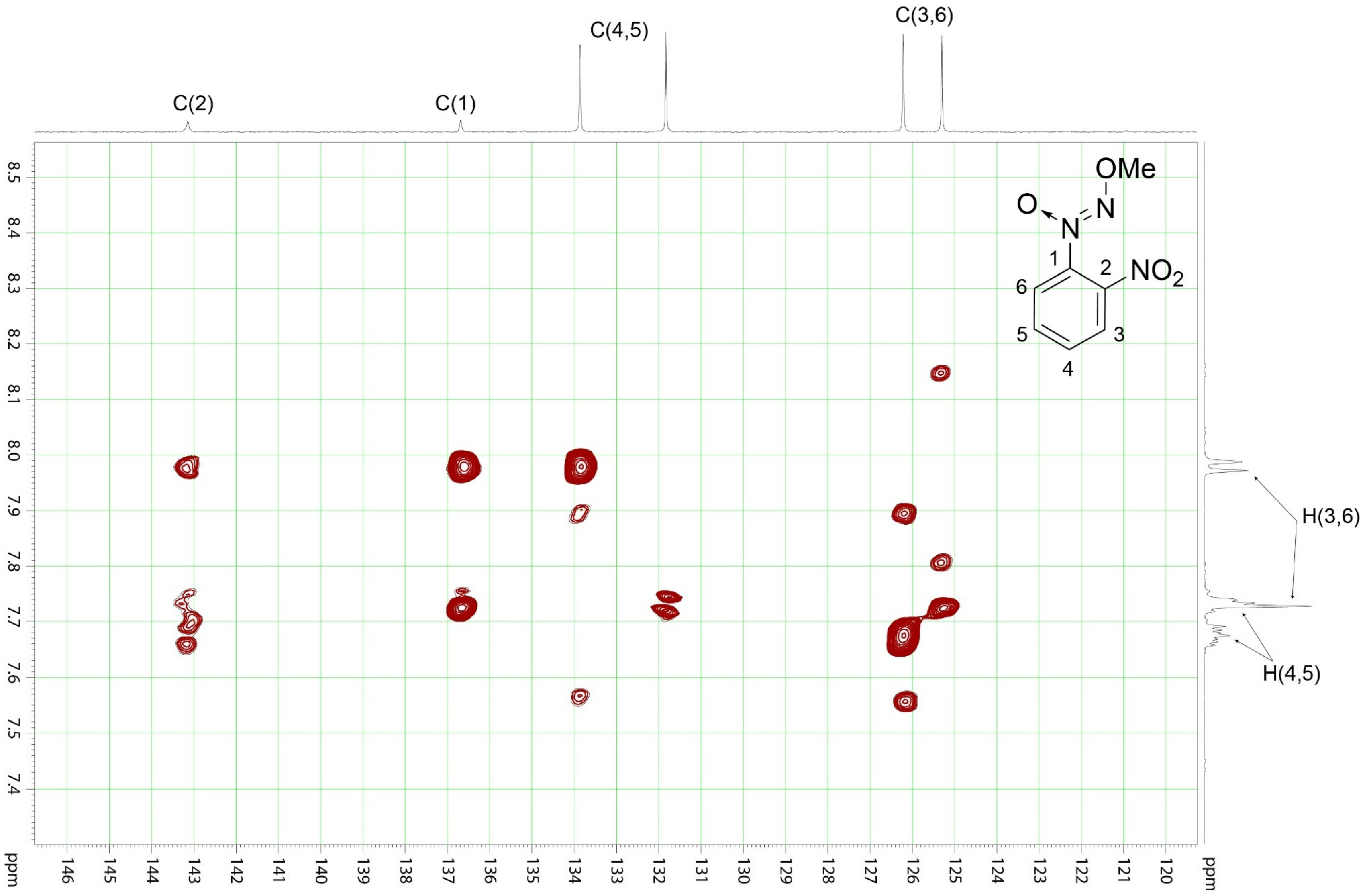

9.2.5 ^{14}N NMR spectrum of compound 2b [36.14 MHz, $CDCl_3$]

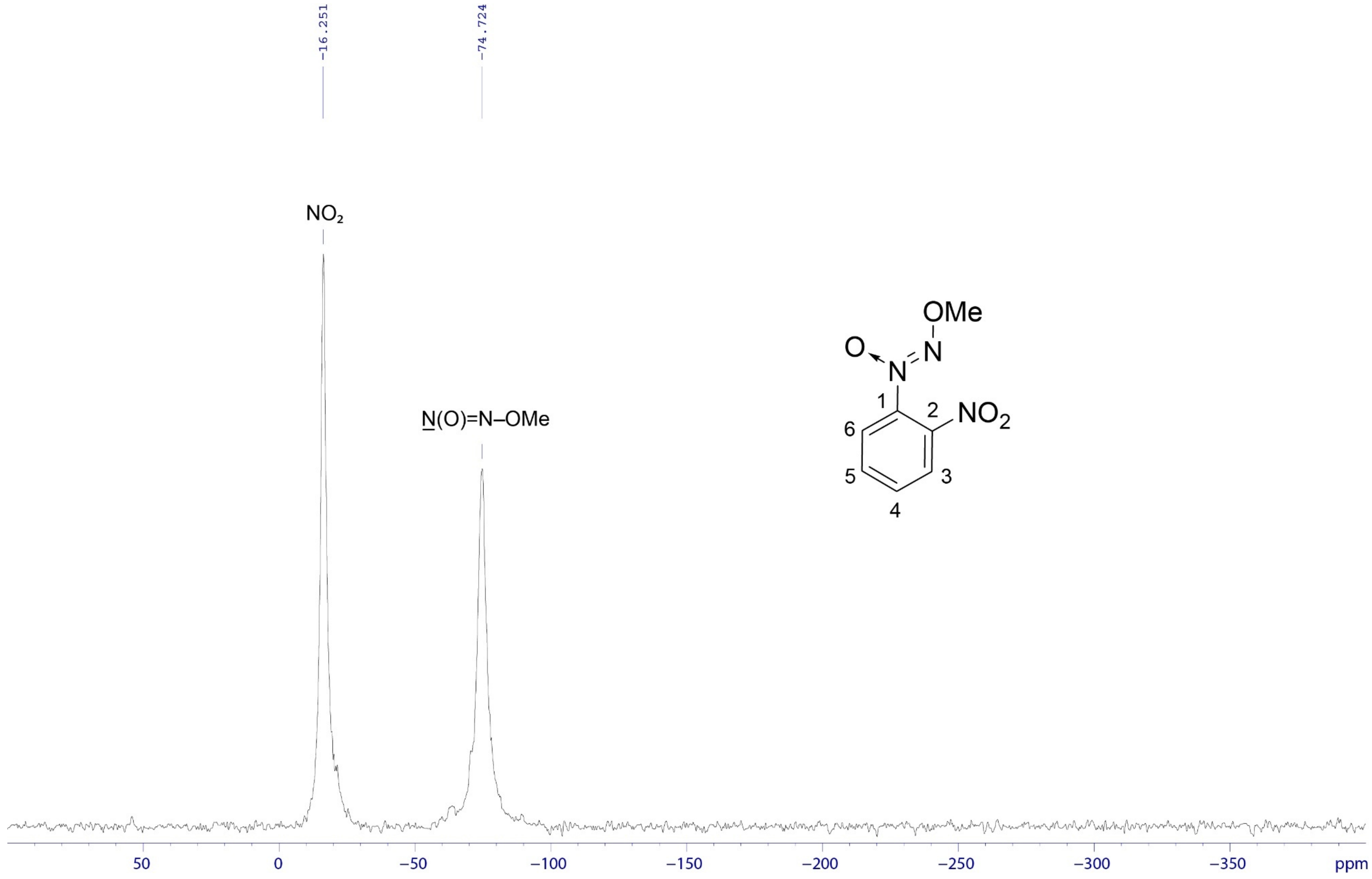

9.3.1 ^{1}H NMR spectrum of compound 2c [500.13 MHz, $CDCl_3$]

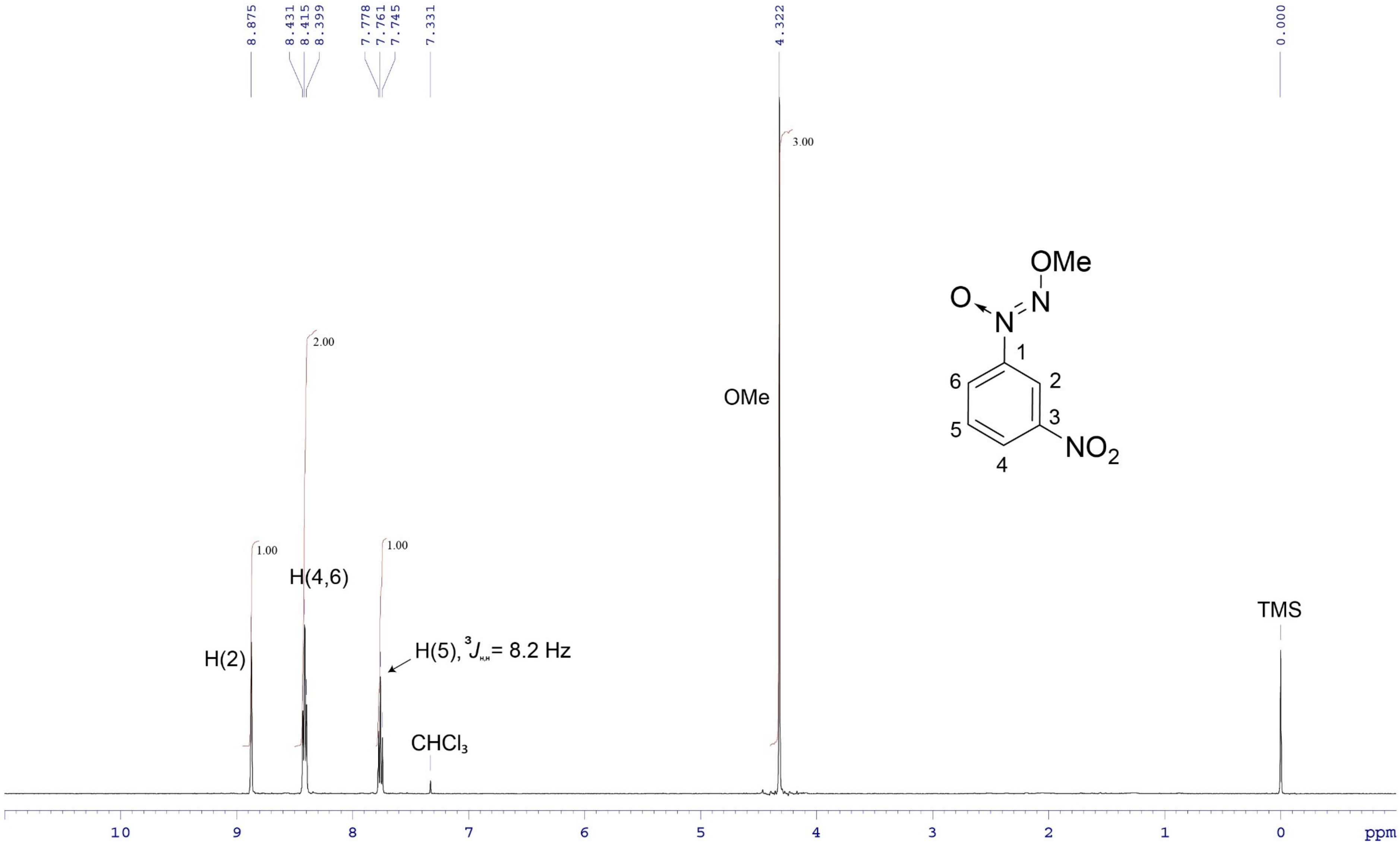

9.3.2 ^{13}C NMR spectrum of compound 2c [125.76 MHz, $CDCl_3$]

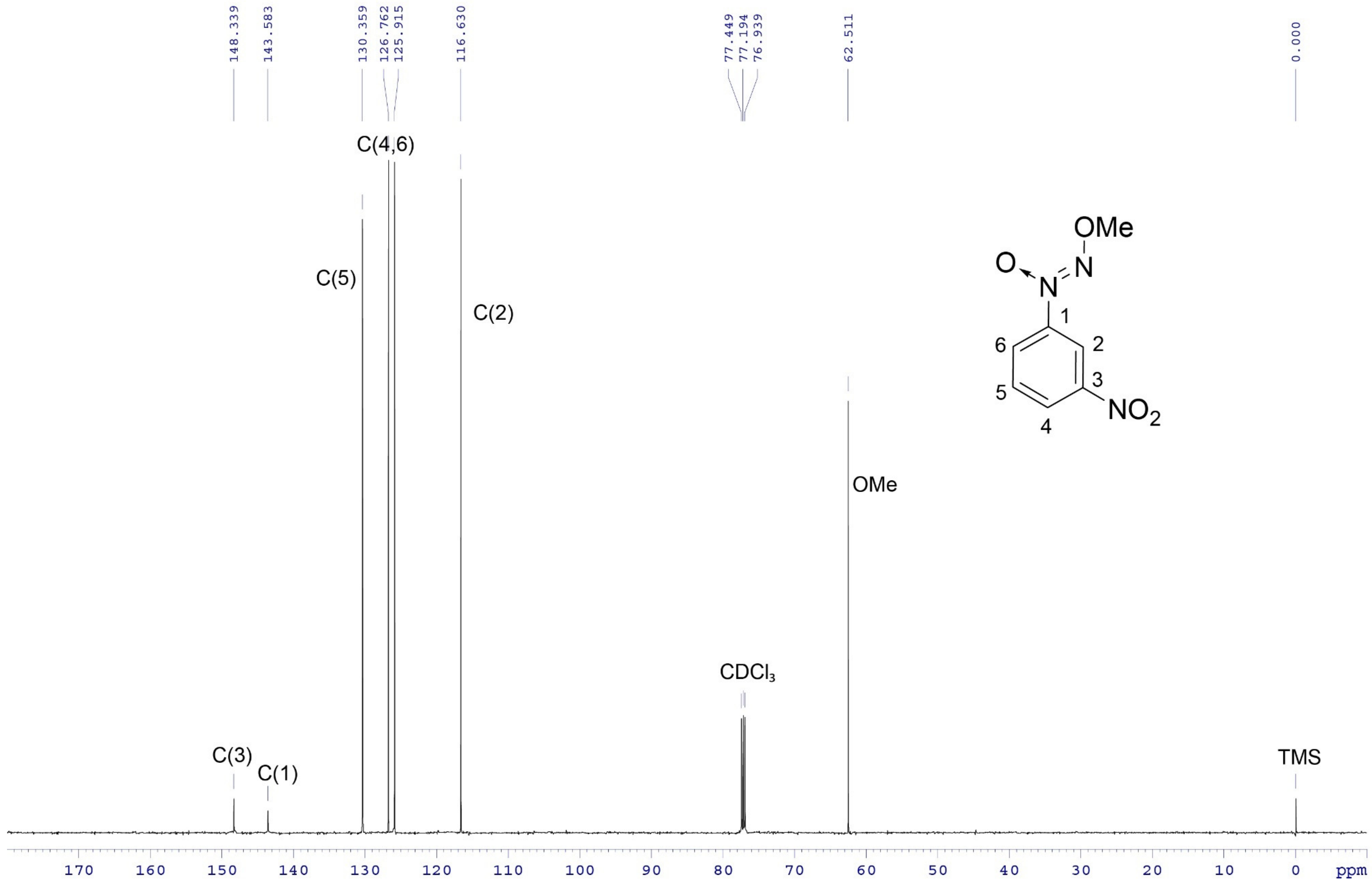

9.3.3 {^1H–^{13}C} HSQC spectrum of compound 2c [500.13 MHz, $CDCl_3$]

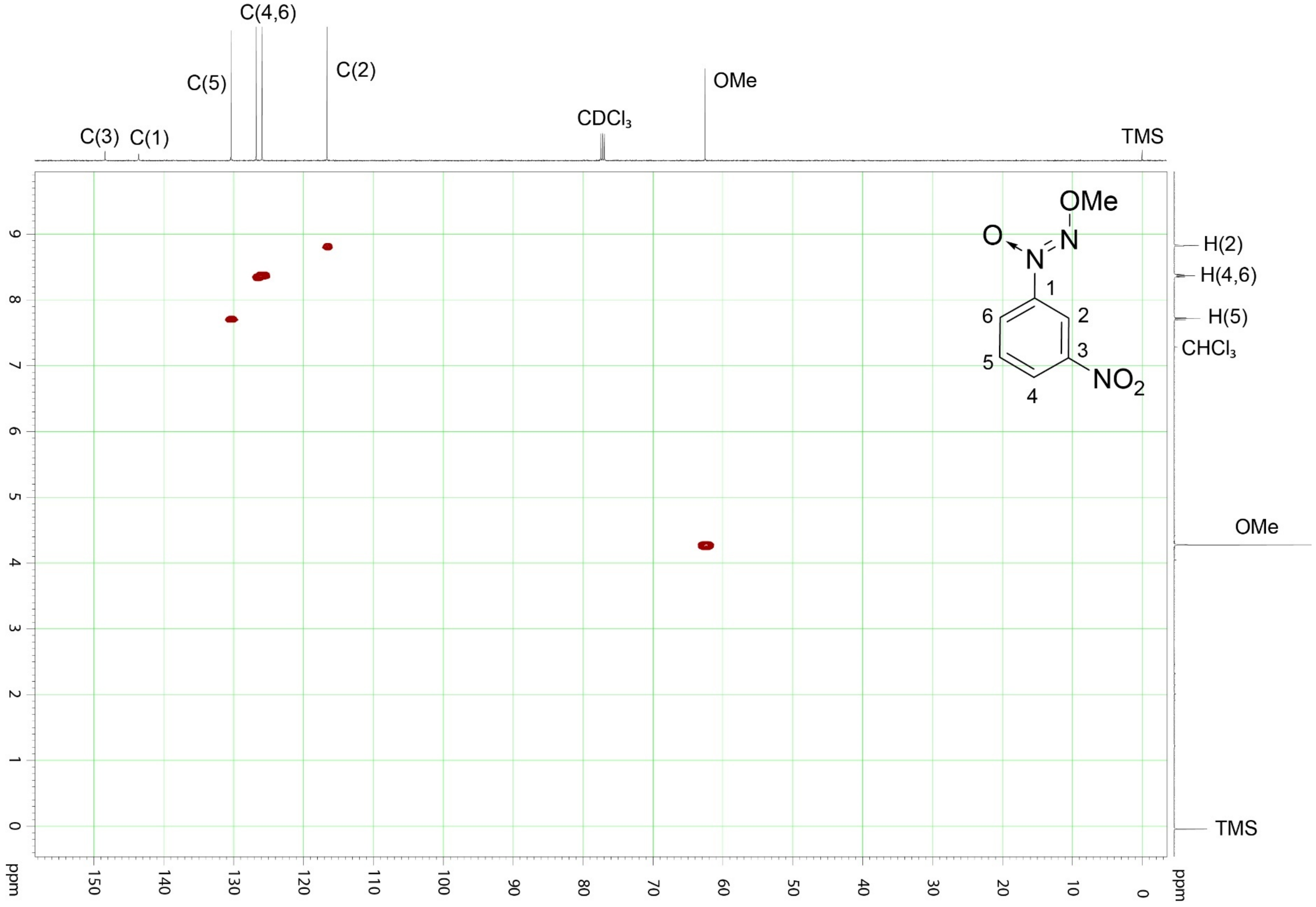

9.3.4 {^1H–^{13}C} HMBC spectrum of compound 2c [500.13 MHz, $CDCl_3$]

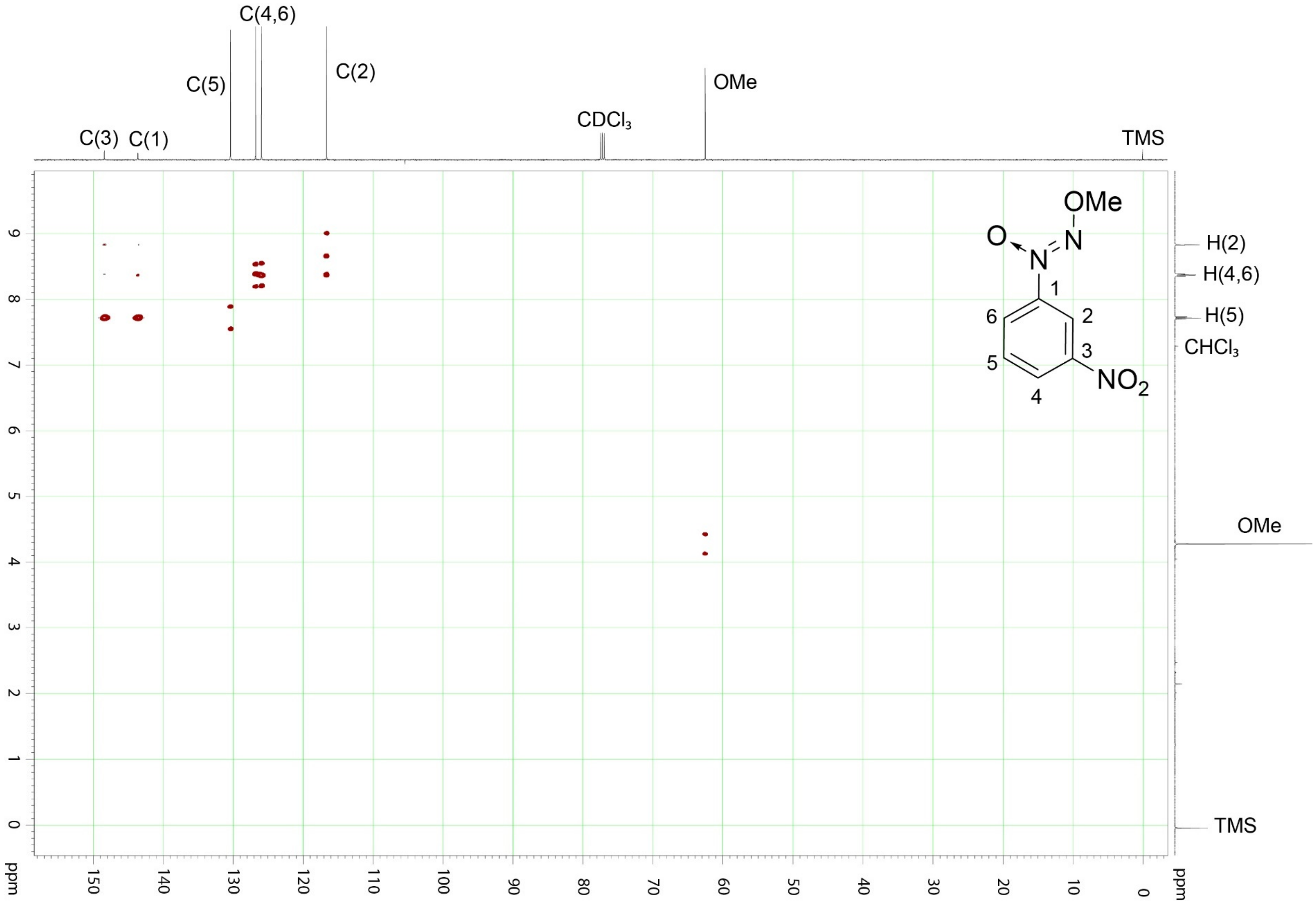

9.3.5 ^{14}N NMR spectrum of compound 2c [43.37 MHz, $CDCl_3$]

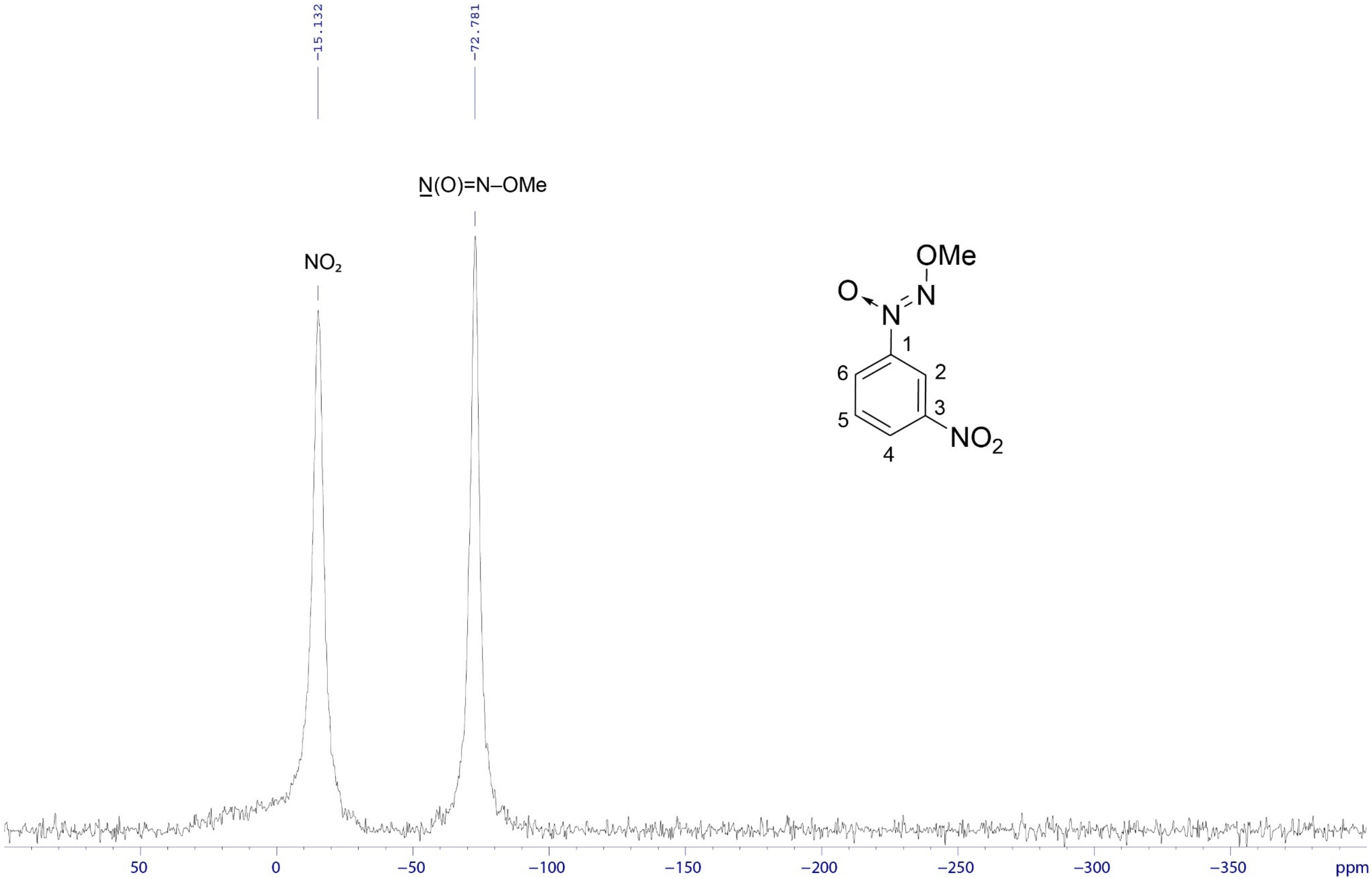

9.4.1 ^{1}H NMR spectrum of compound 2d [500.13 MHz, $CDCl_3$]

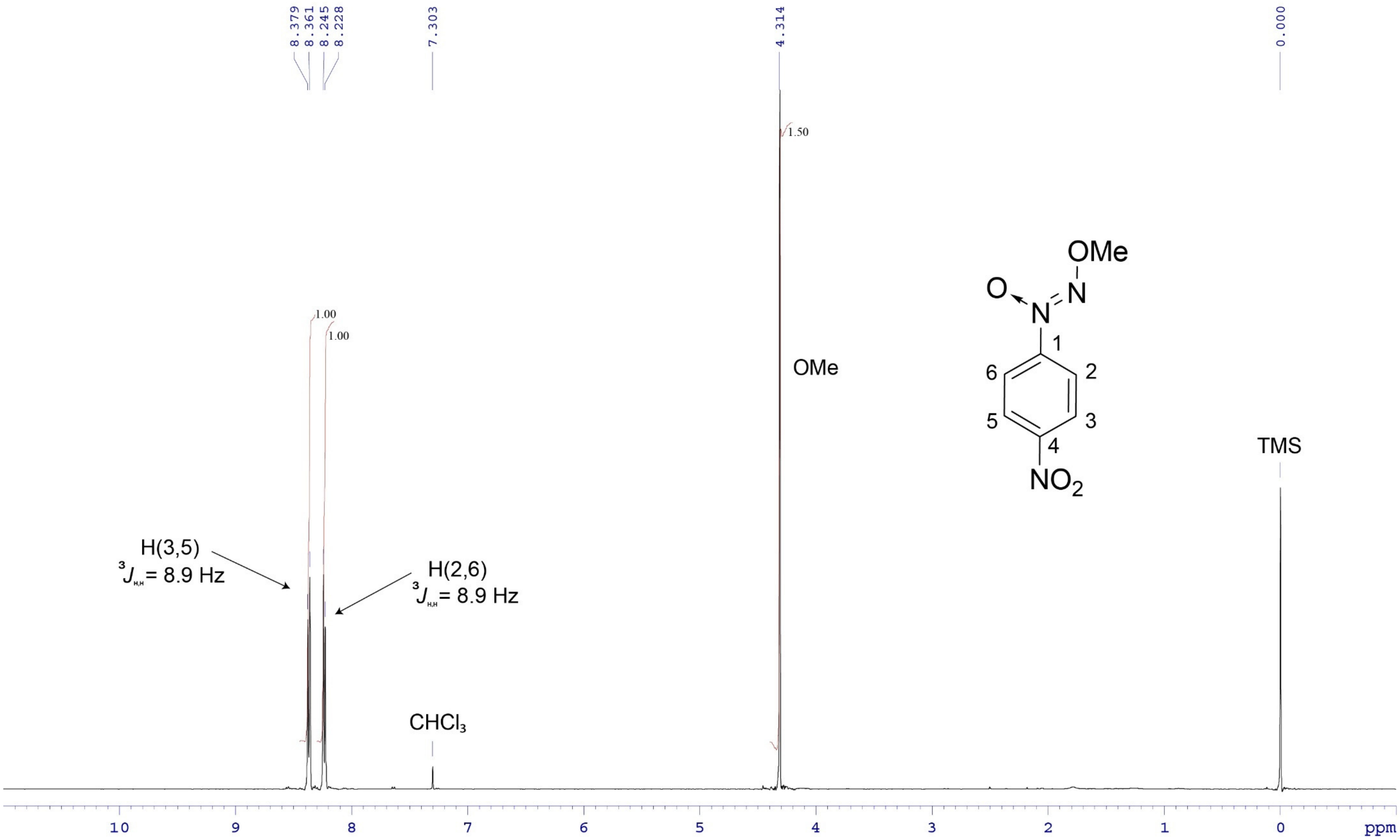

9.4.2 ^{13}C NMR spectrum of compound 2d [125.76 MHz, $CDCl_3$]

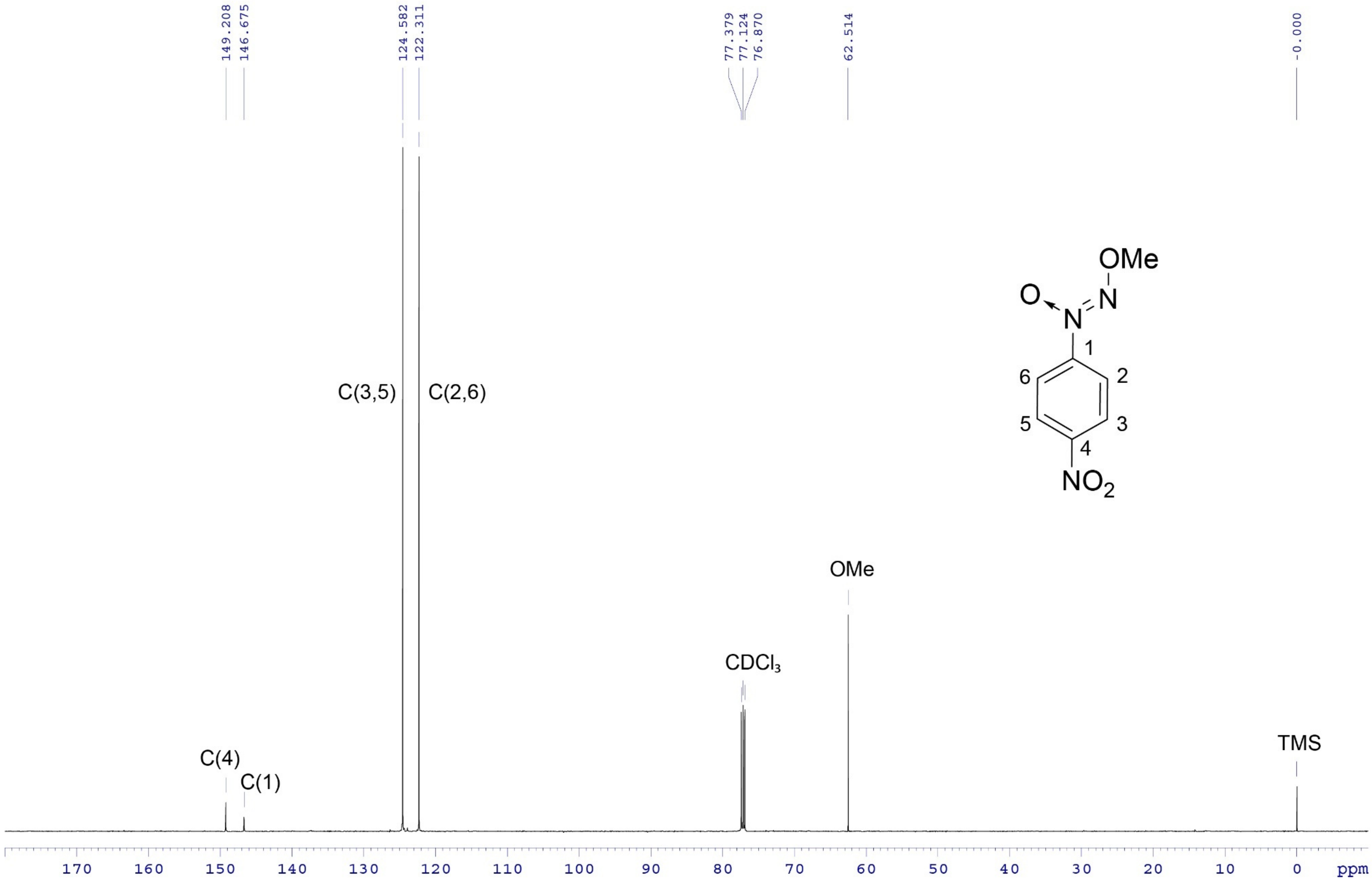

9.4.3 {^1H–^{13}C} HSQC spectrum of compound 2d [500.13 MHz, $CDCl_3$]

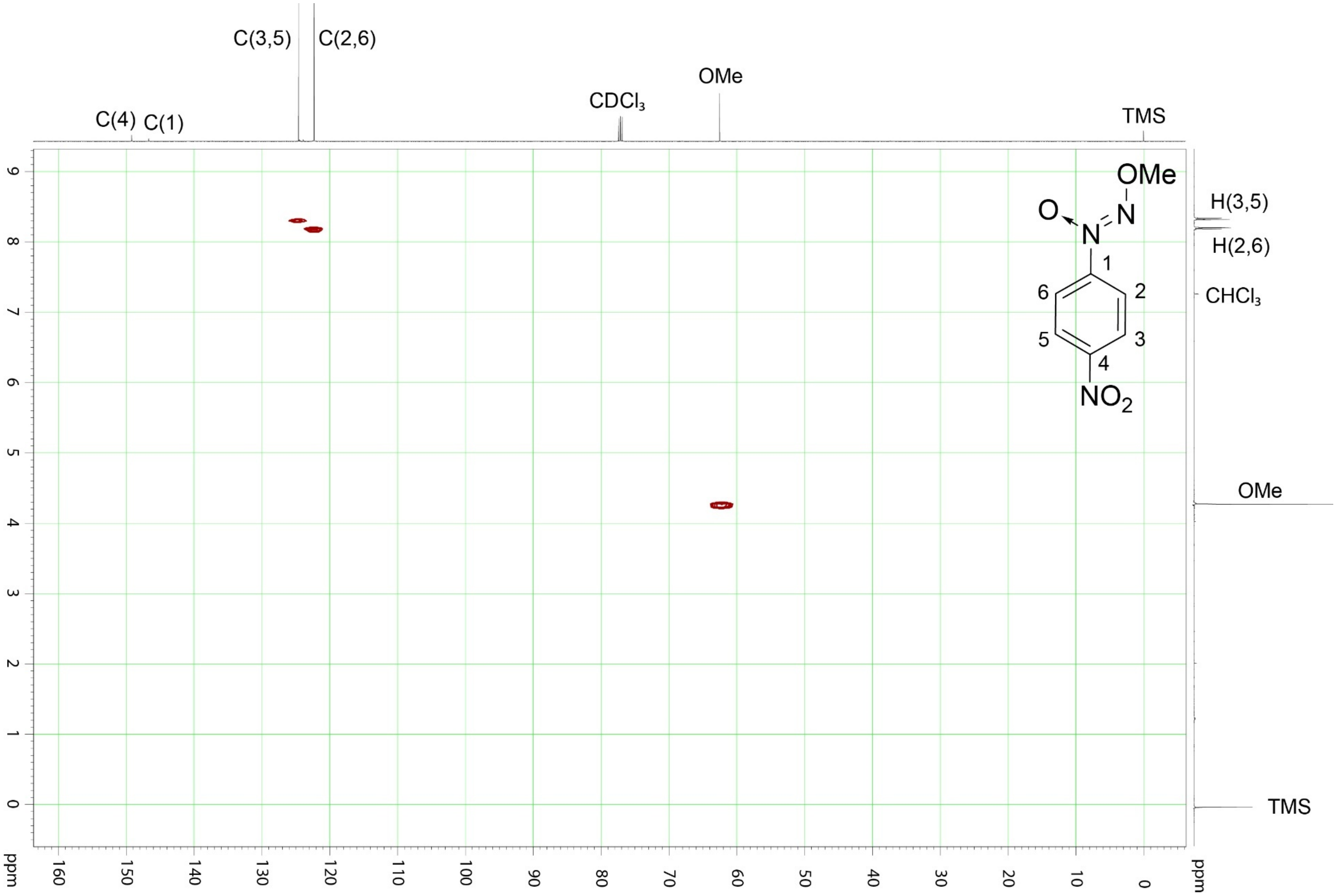

9.4.4 {^{1}H–^{13}C} HMBC spectrum of compound 2d [500.13 MHz, $CDCl_3$]

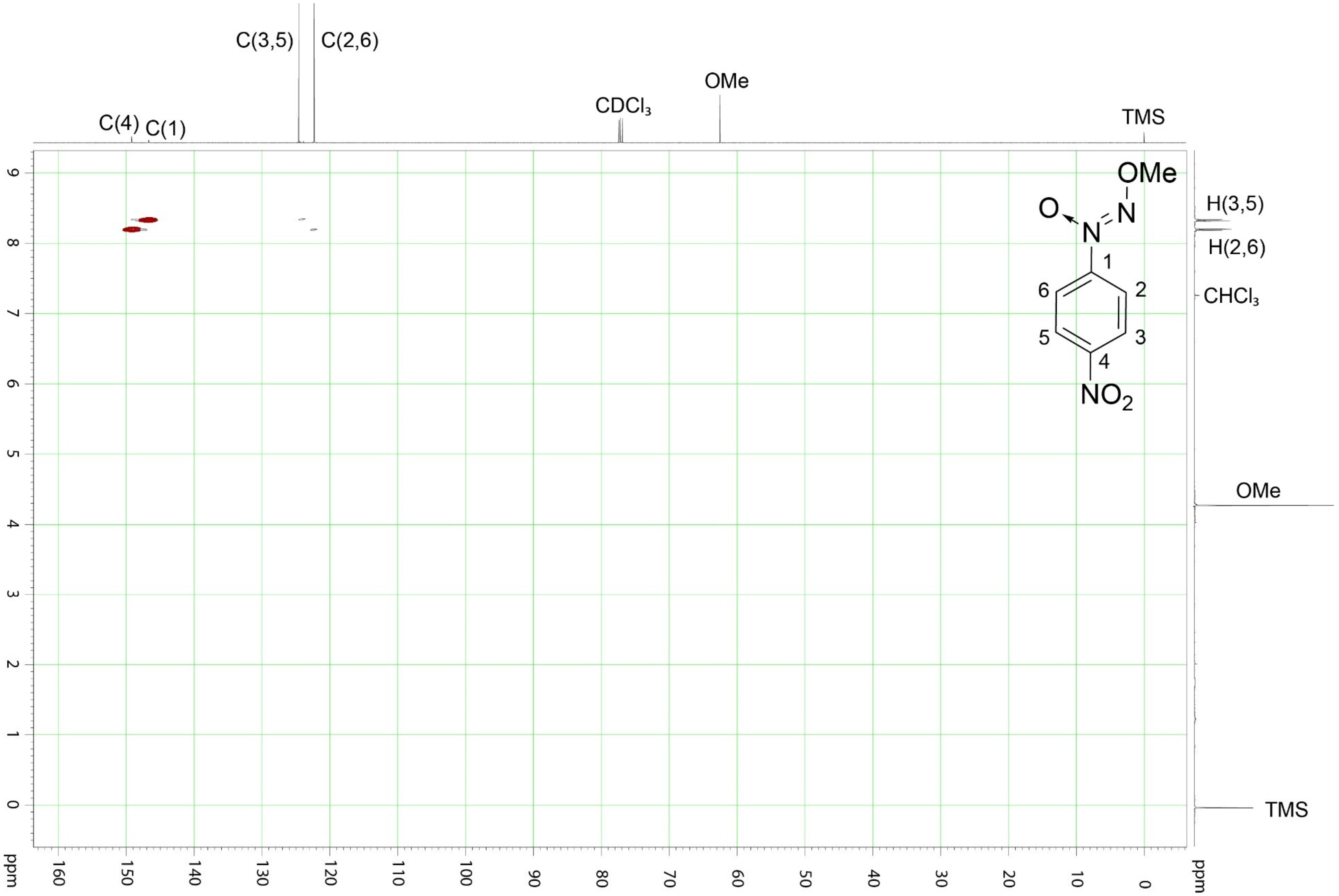

9.4.5 ^{14}N NMR spectrum of compound 2d [36.14 MHz, $CDCl_3$]

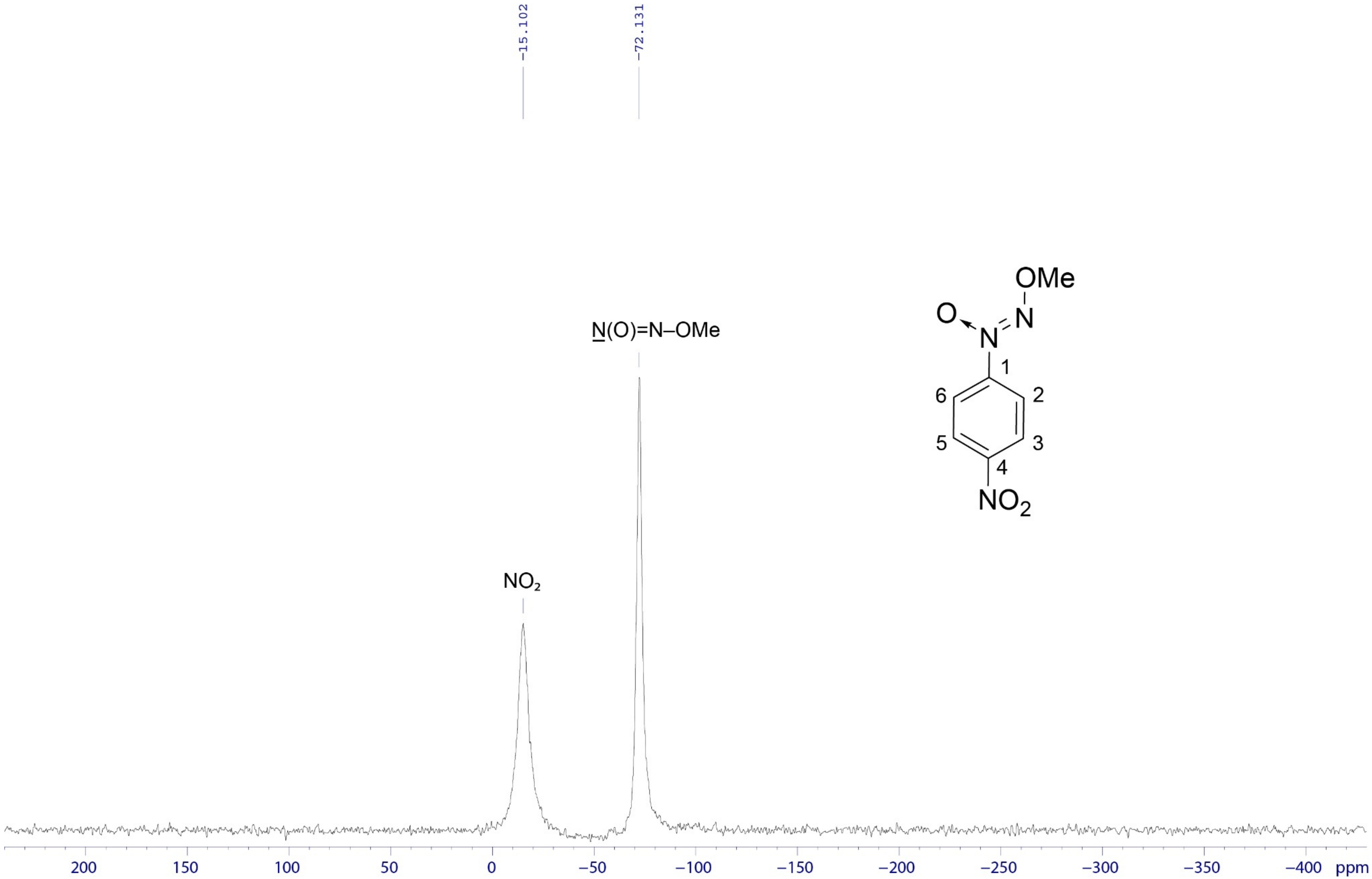

9.5.1 ^1H NMR spectrum of compound 2e [500.13 MHz, $CDCl_3$]

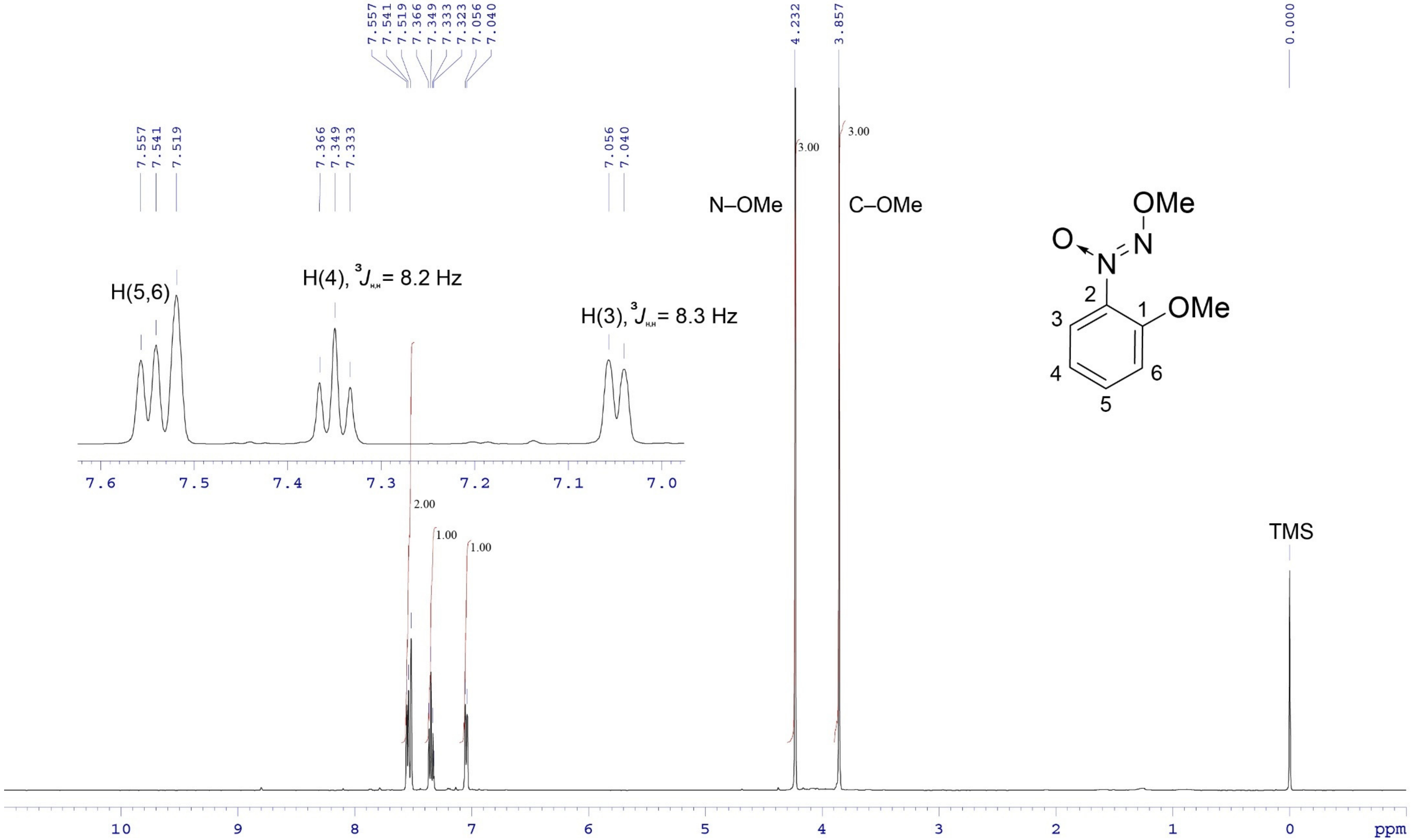

9.5.2 ^{13}C NMR spectrum of compound 2e [125.76 MHz, $CDCl_3$]

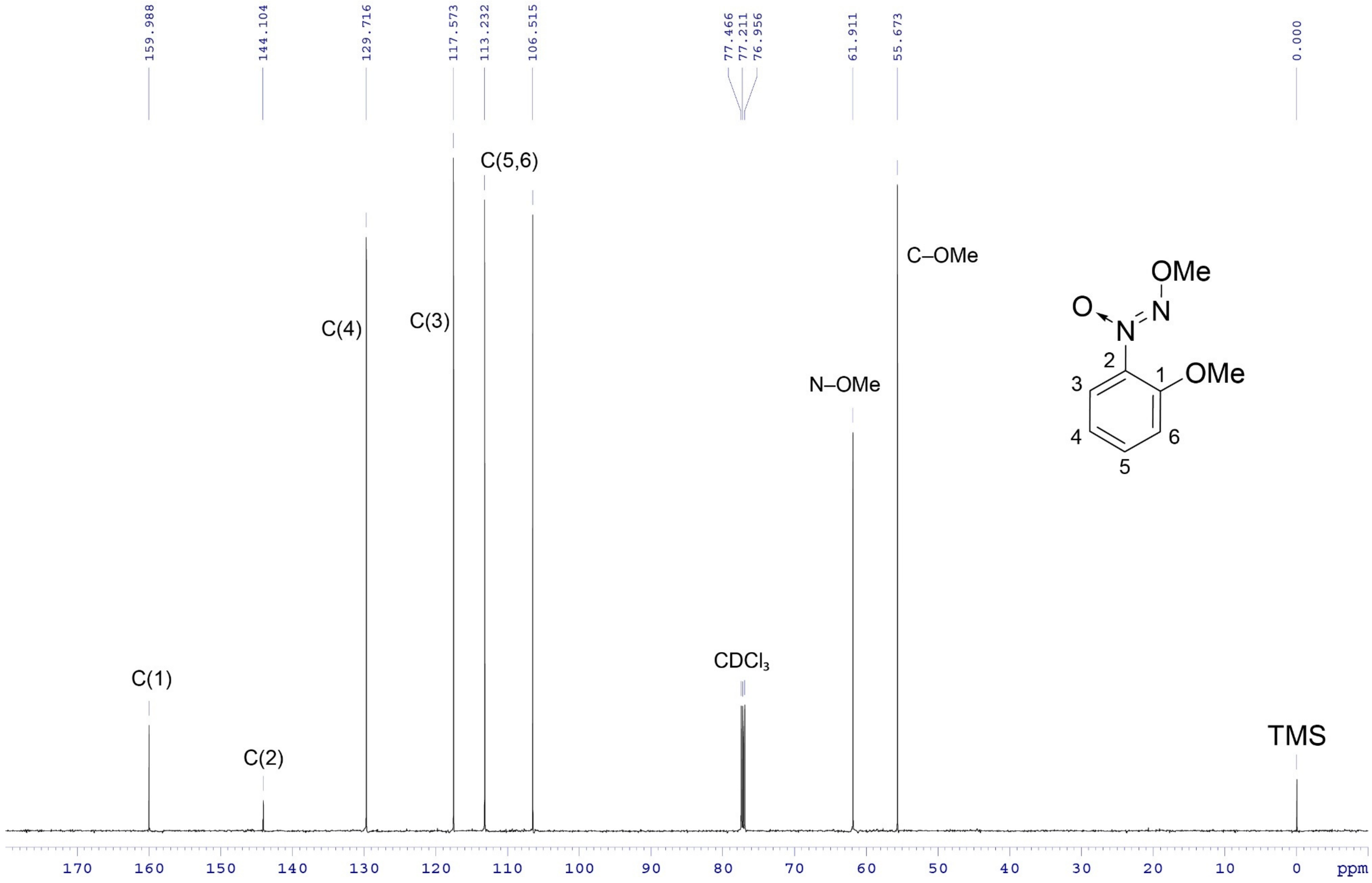

9.5.3 {^1H–^{13}C} HSQC spectrum of compound 2e [500.13 MHz, $CDCl_3$]

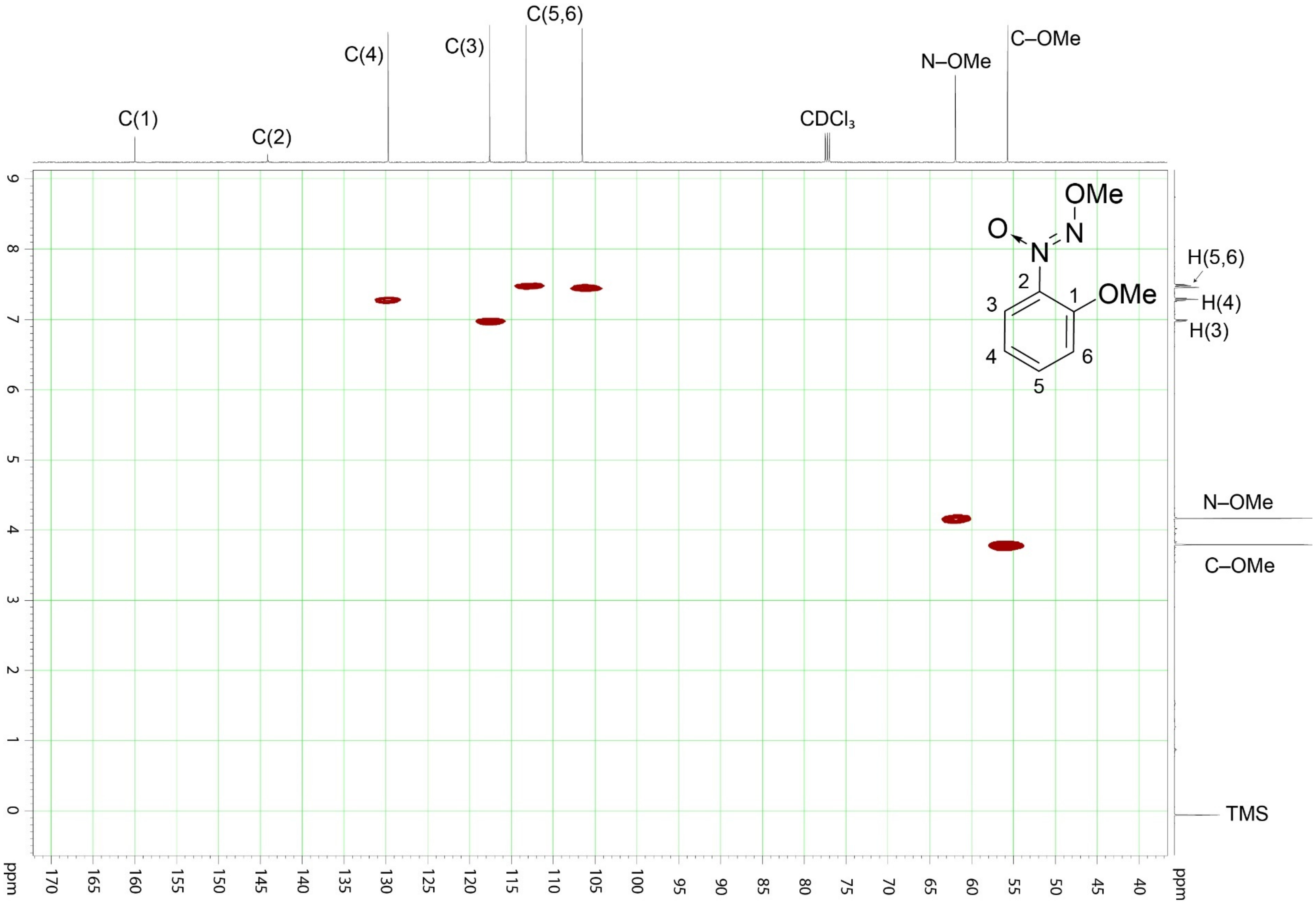

9.5.4 {^{1}H–^{13}C} HMBC spectrum of compound 2e [500.13 MHz, $CDCl_3$]

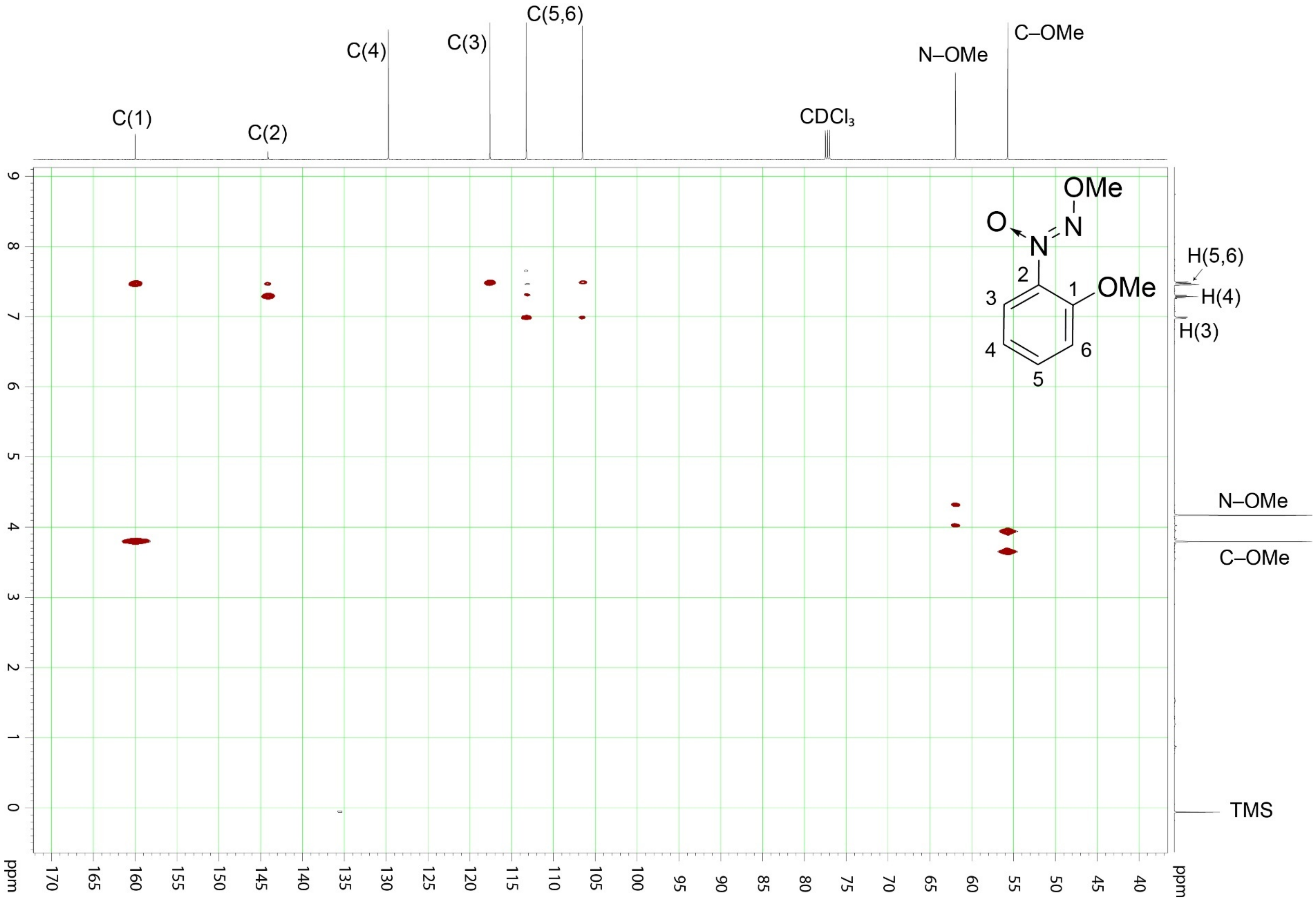

9.5.5 ^{14}N NMR spectrum of compound 2e [36.14 MHz, $CDCl_3$]

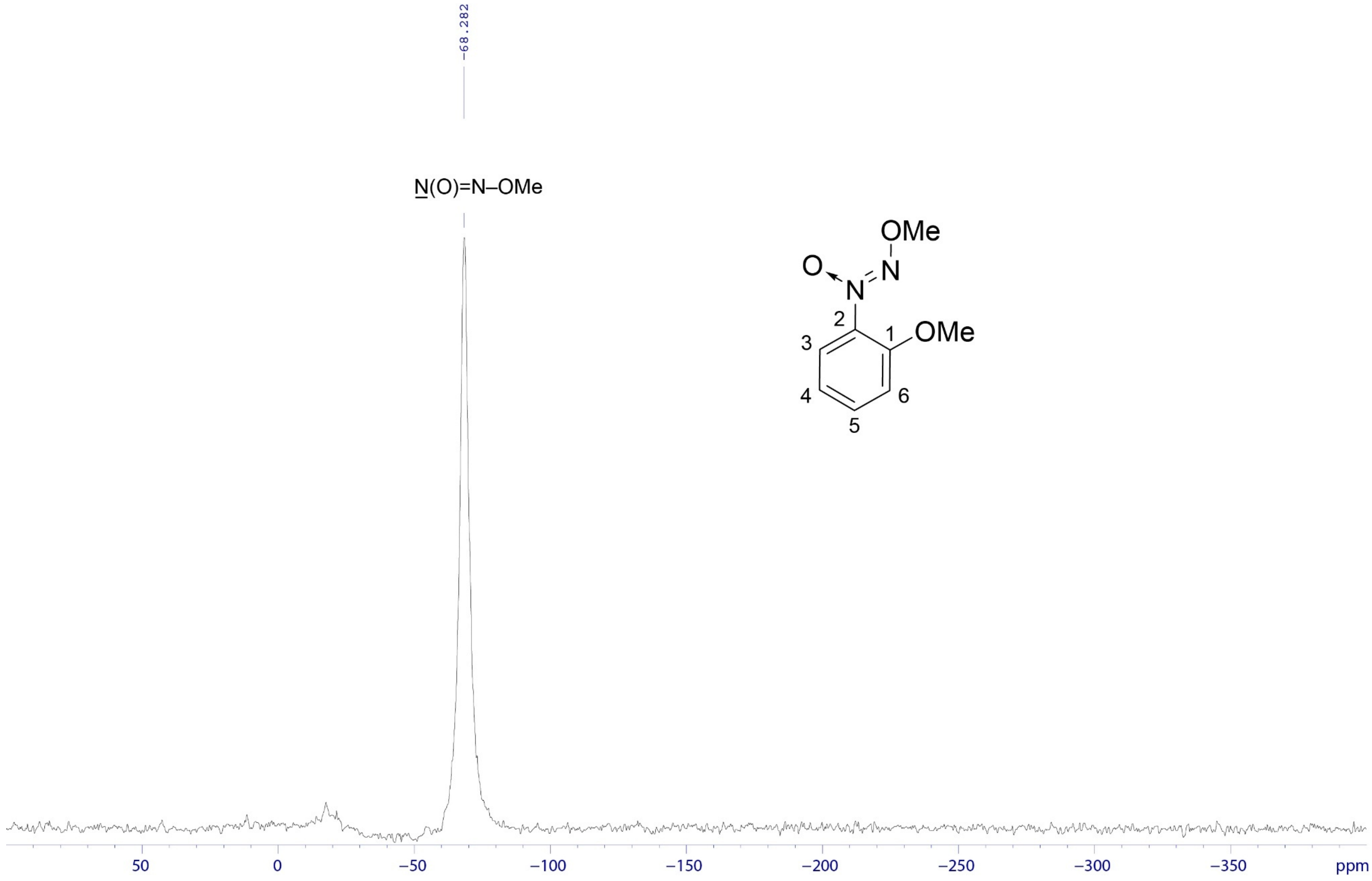

9.6.1 ^{1}H NMR spectrum of compound 2f [600.13 MHz, $CDCl_3$]

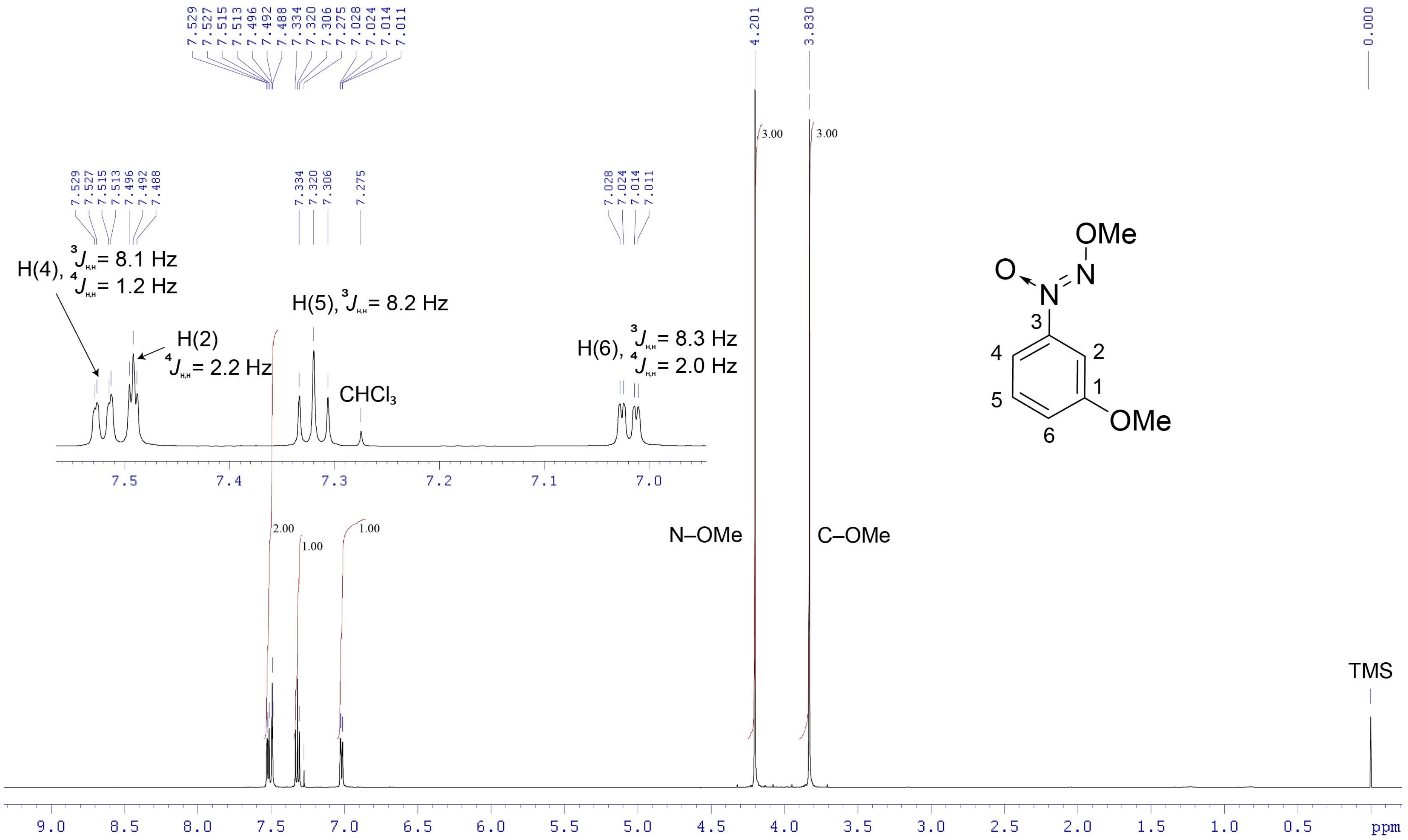

9.6.2 ^{13}C NMR spectrum of compound 2f [150.90 MHz, $CDCl_3$]

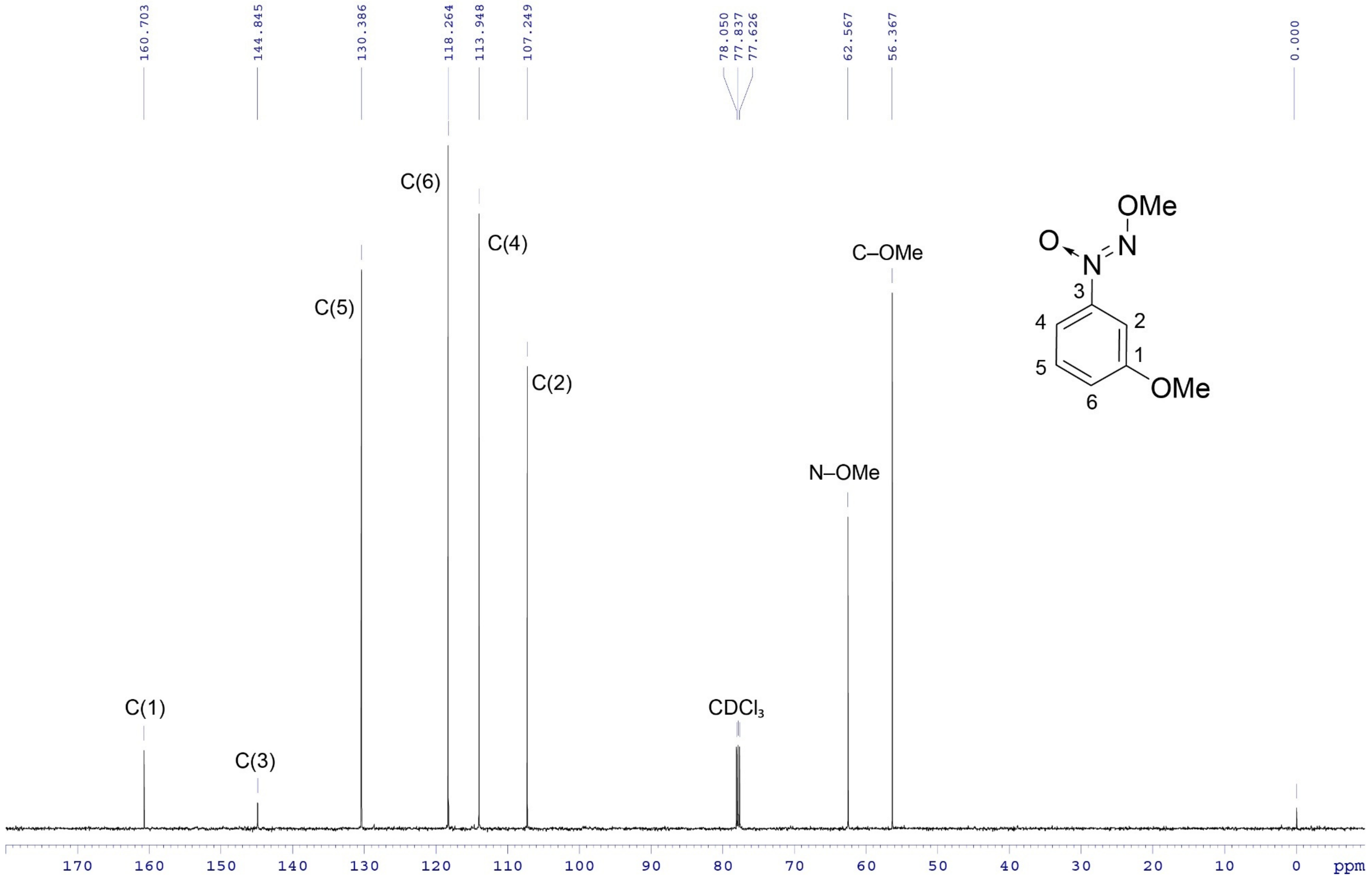

9.6.3 {^{1}H–^{13}C} HSQC spectrum of compound 2f [600.13 MHz, $CDCl_3$]

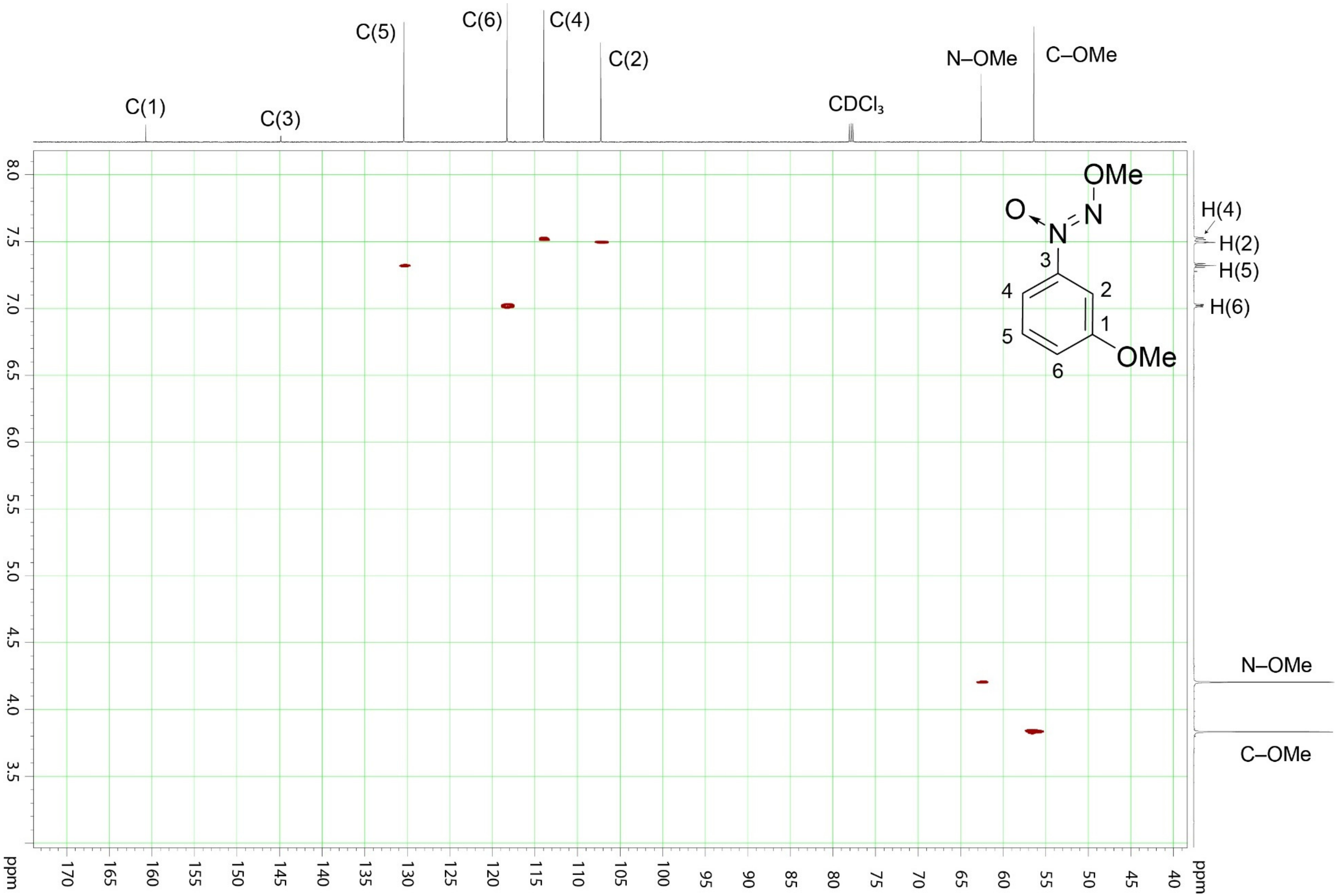

9.6.4 {$^{1}H–^{13}C$} HMBC spectrum of compound 2f [600.13 MHz, $CDCl_3$]

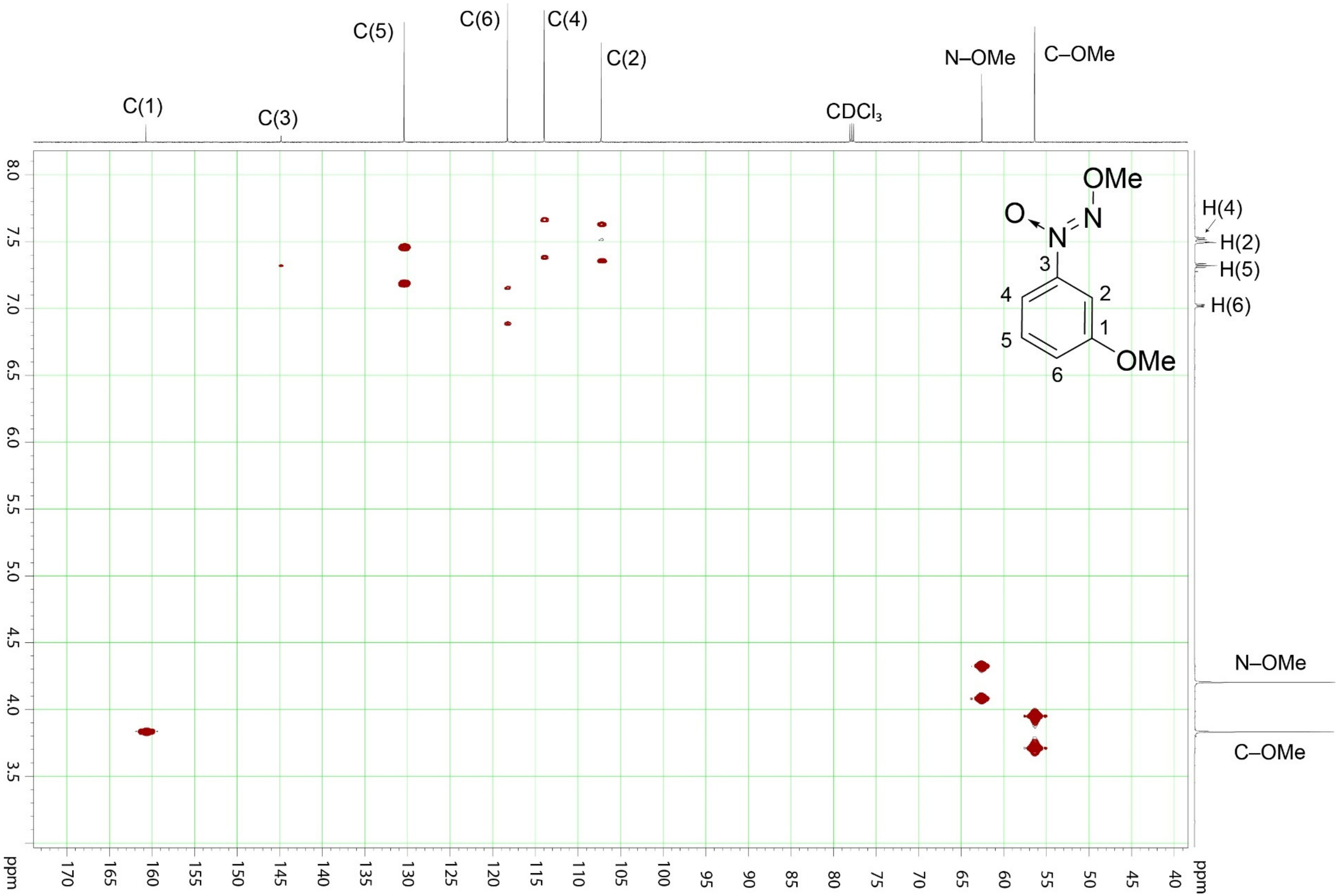

9.6.5 ^{14}N NMR spectrum of compound 2f [43.37 MHz, $CDCl_3$]

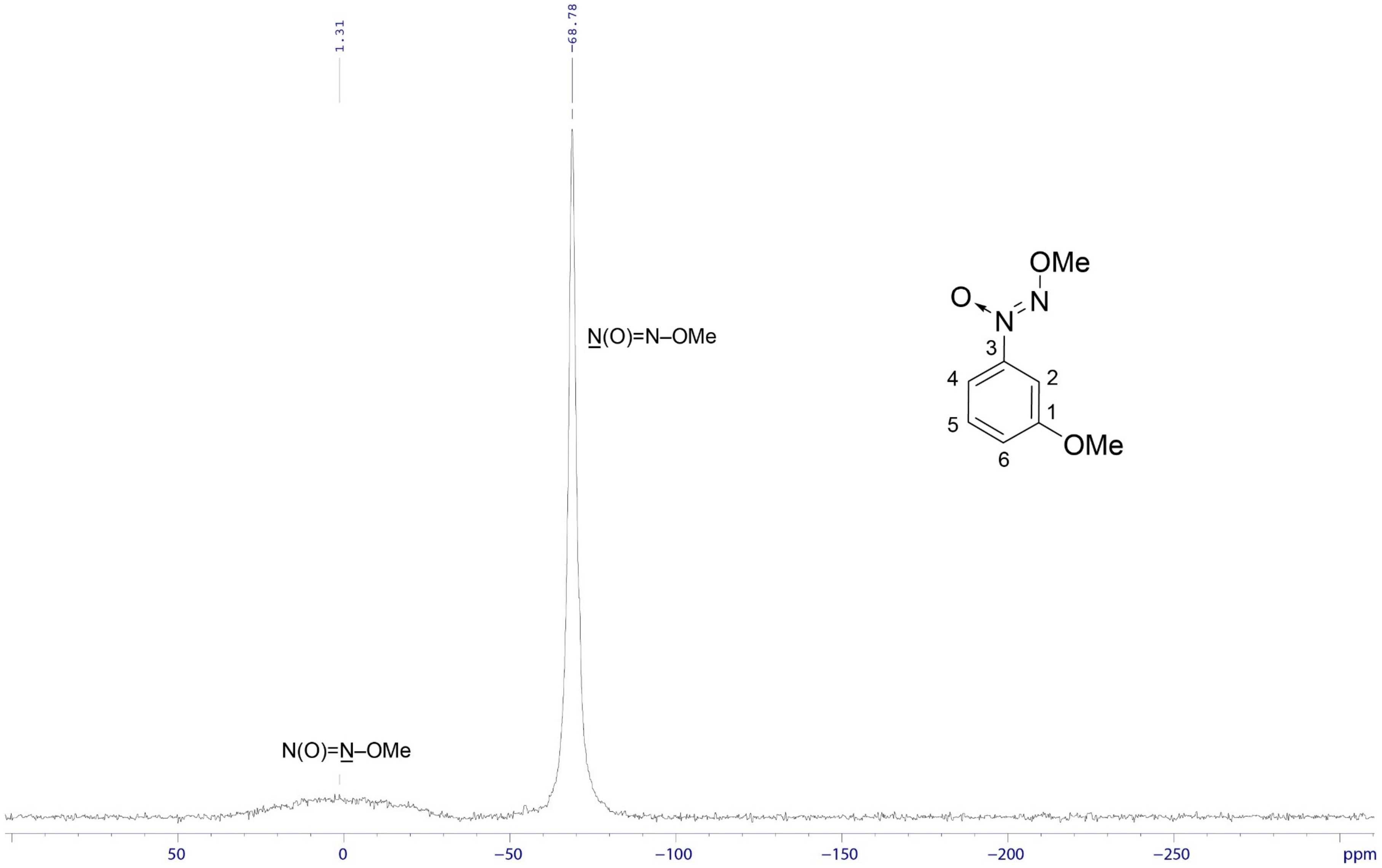

9.7.1 ^{1}H NMR spectrum of compound 2g [600.13 MHz, $CDCl_3$]

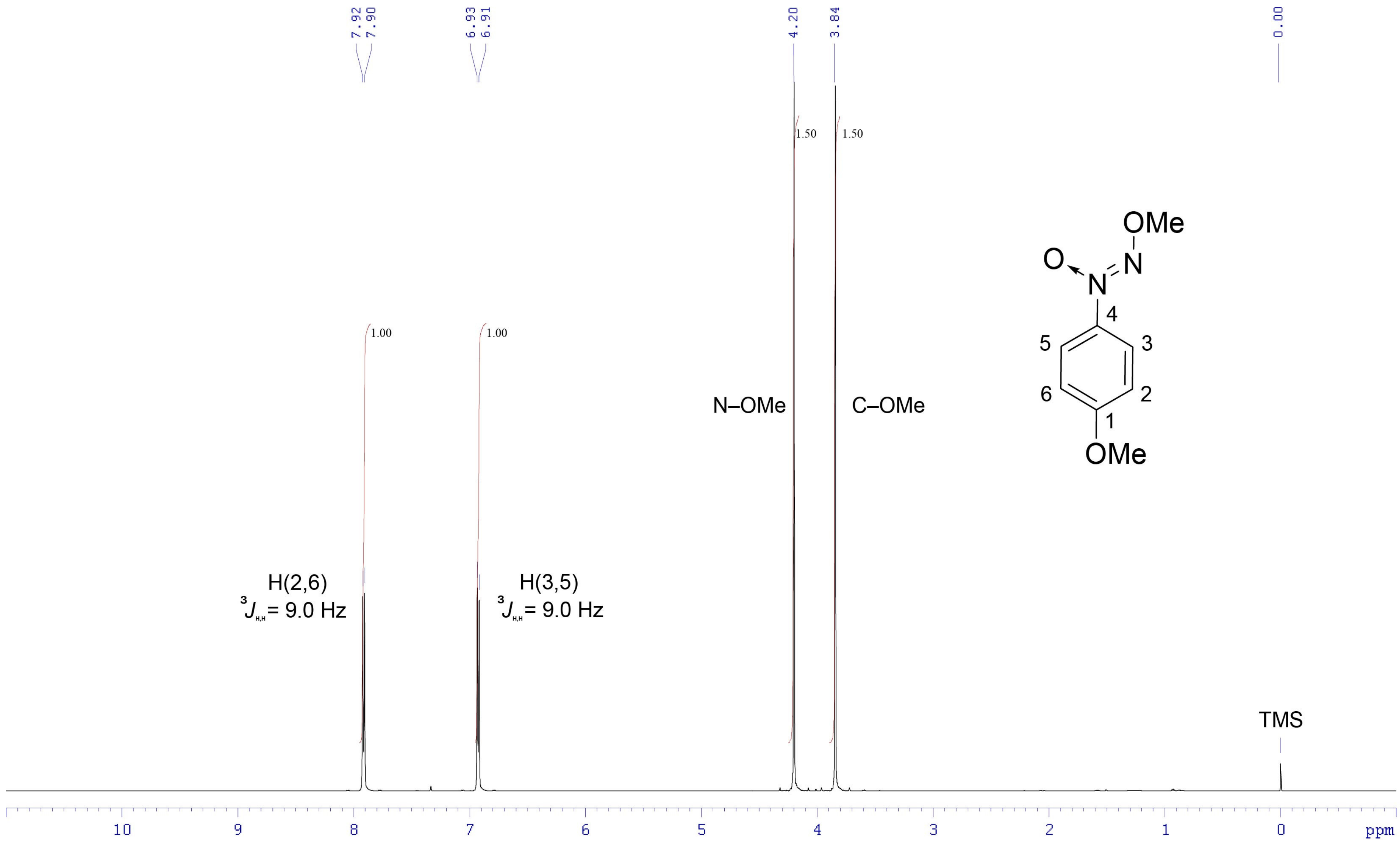

9.7.2 ^{13}C NMR spectrum of compound 2g [150.90 MHz, $CDCl_3$]

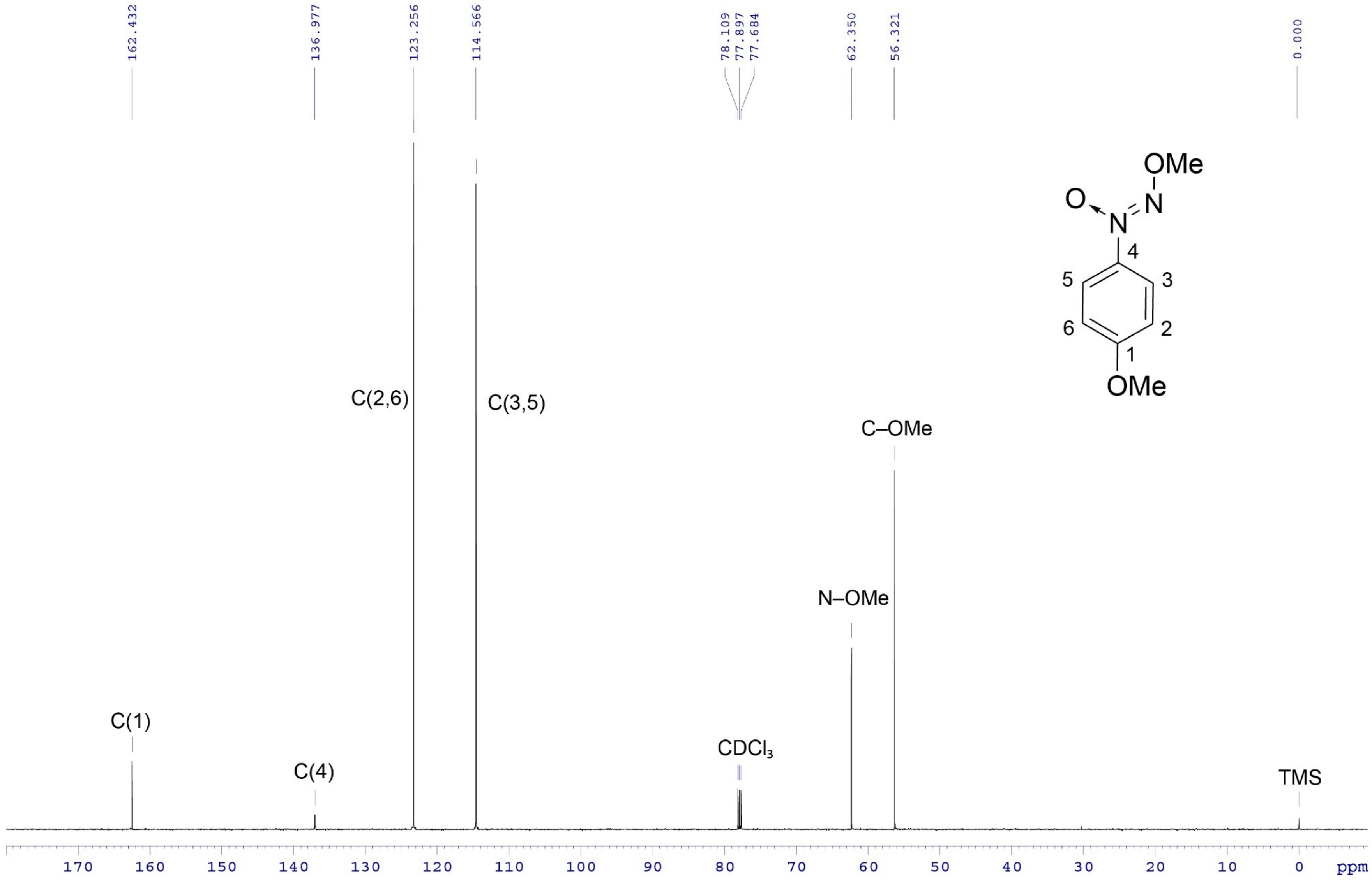

9.7.3 {^{1}H–^{13}C} HSQC spectrum of compound 2g [600.13 MHz, $CDCl_3$]

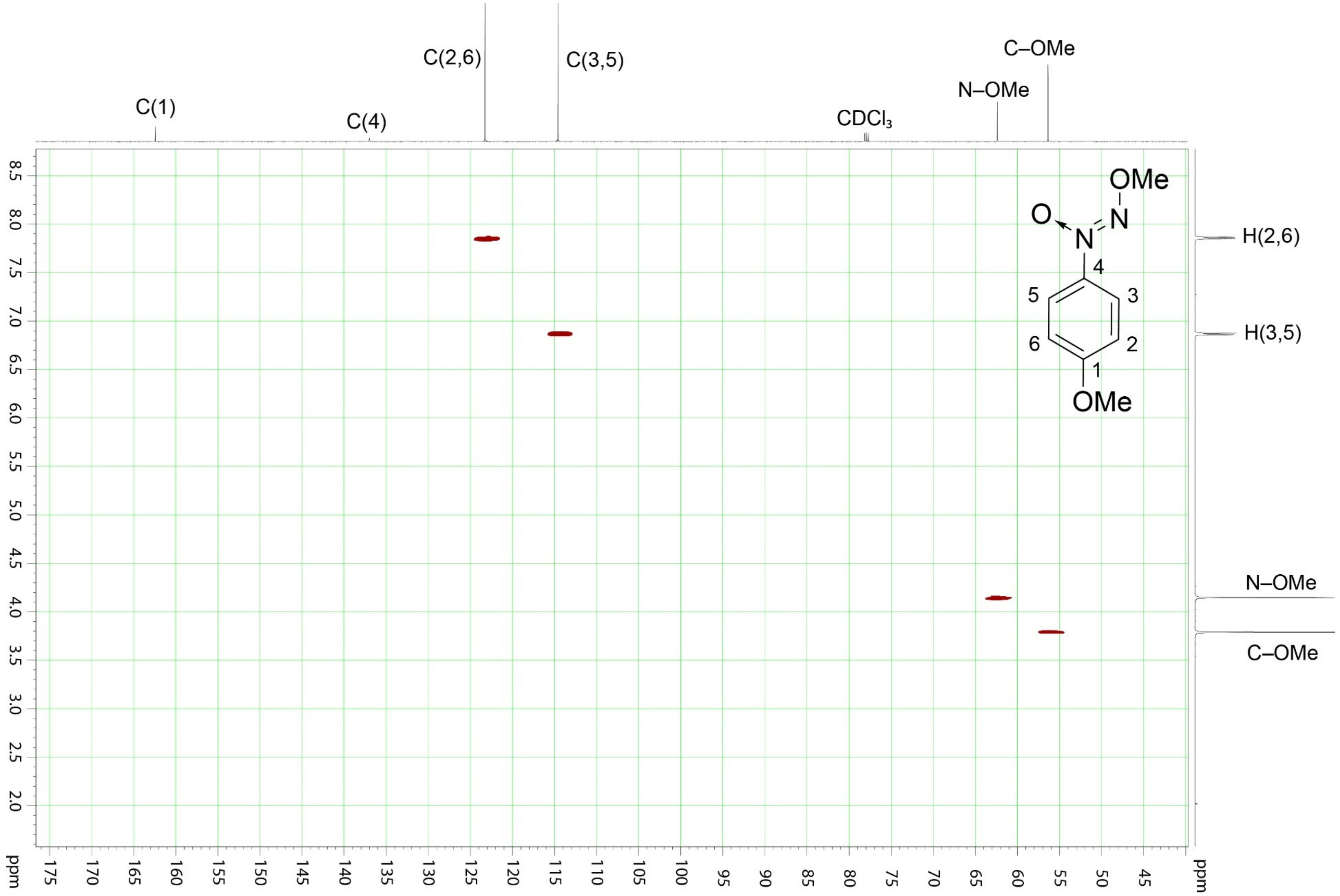

9.7.4 {^1H–^{13}C} HMBC spectrum of compound 2g [600.13 MHz, $CDCl_3$]

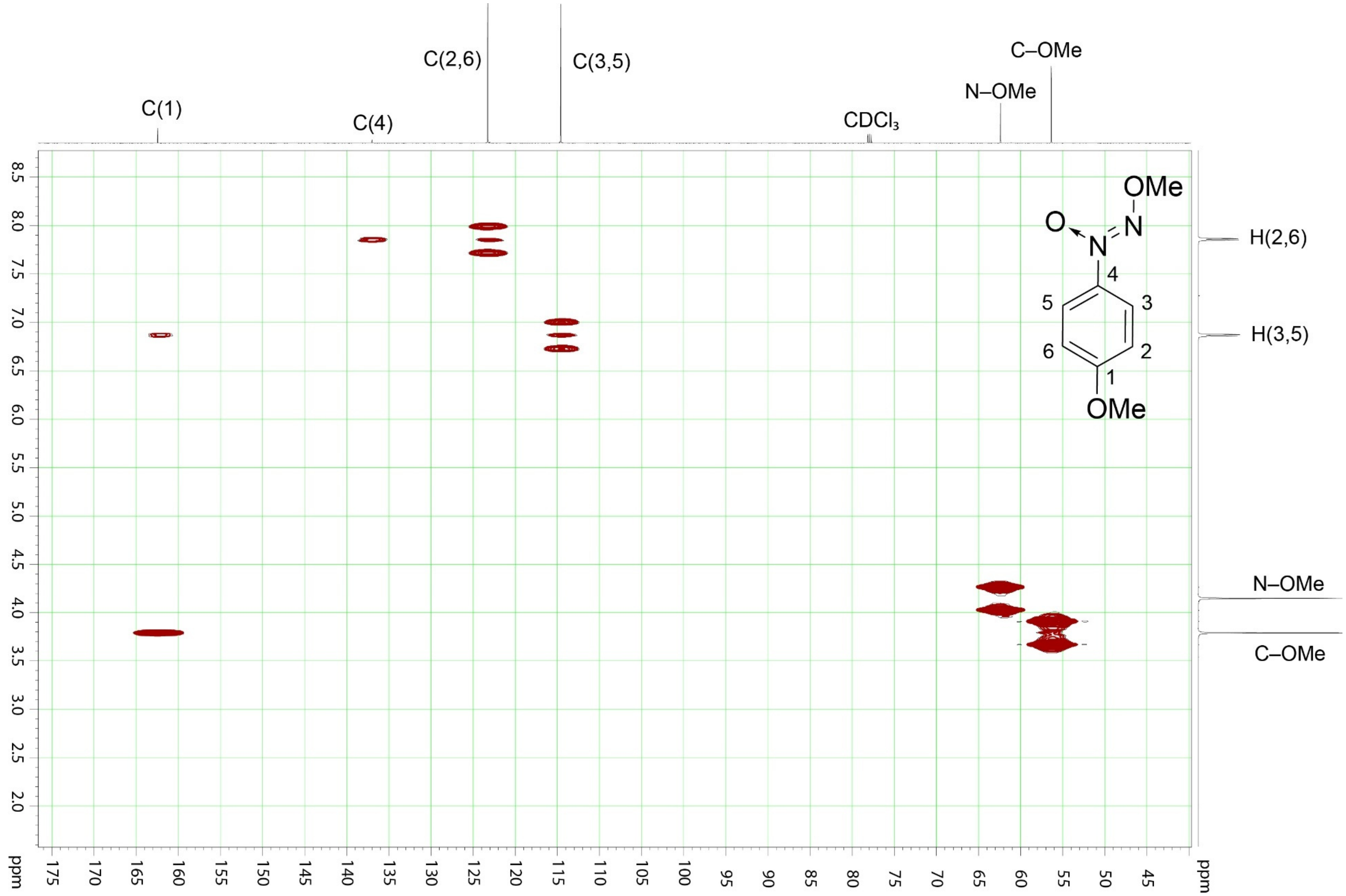

9.7.5 ^{14}N NMR spectrum of compound 2g [43.14 MHz, $CDCl_3$]

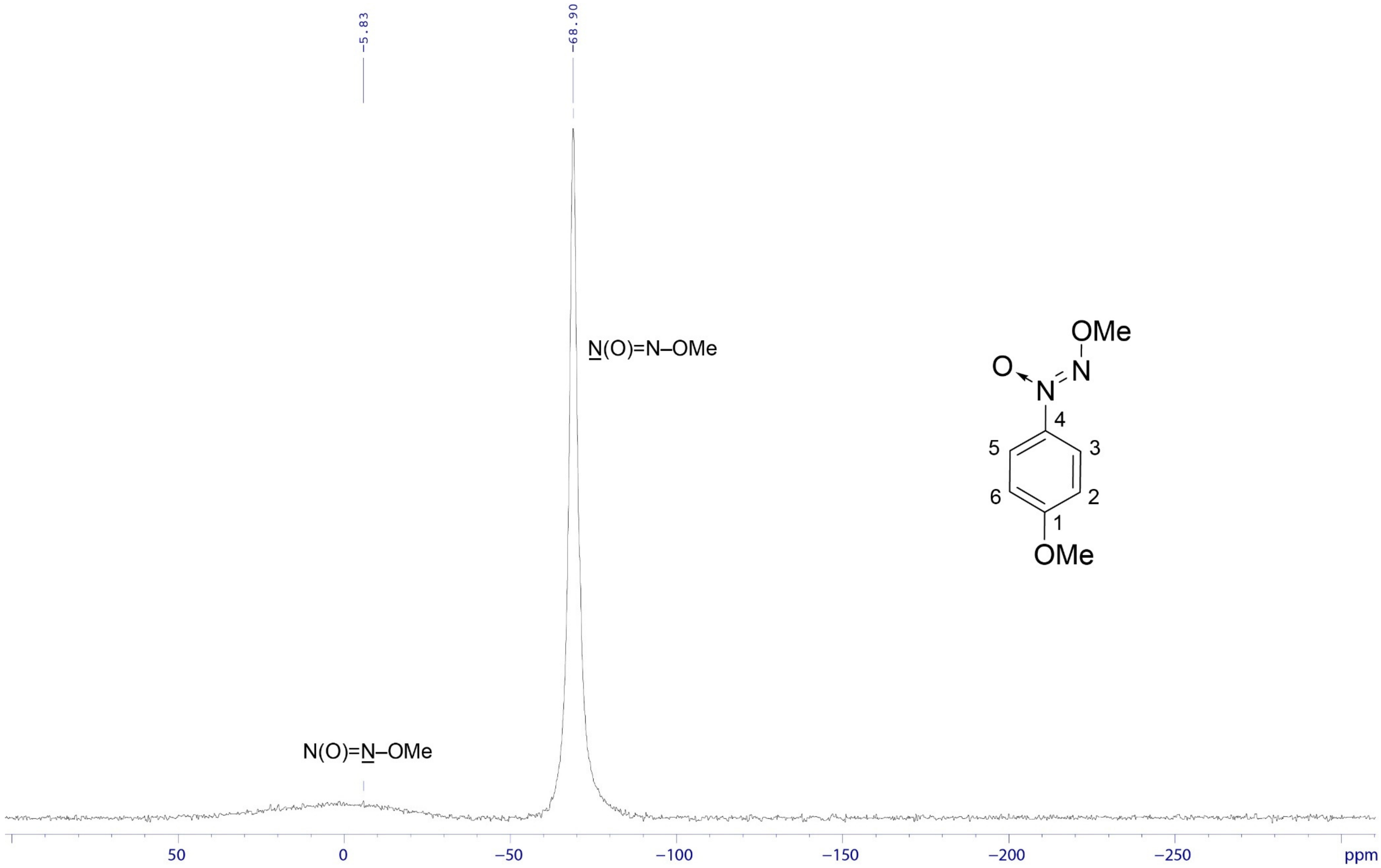

9.8.1 ^{1}H NMR spectrum of compound 2h [500.13 MHz, $CDCl_3$]

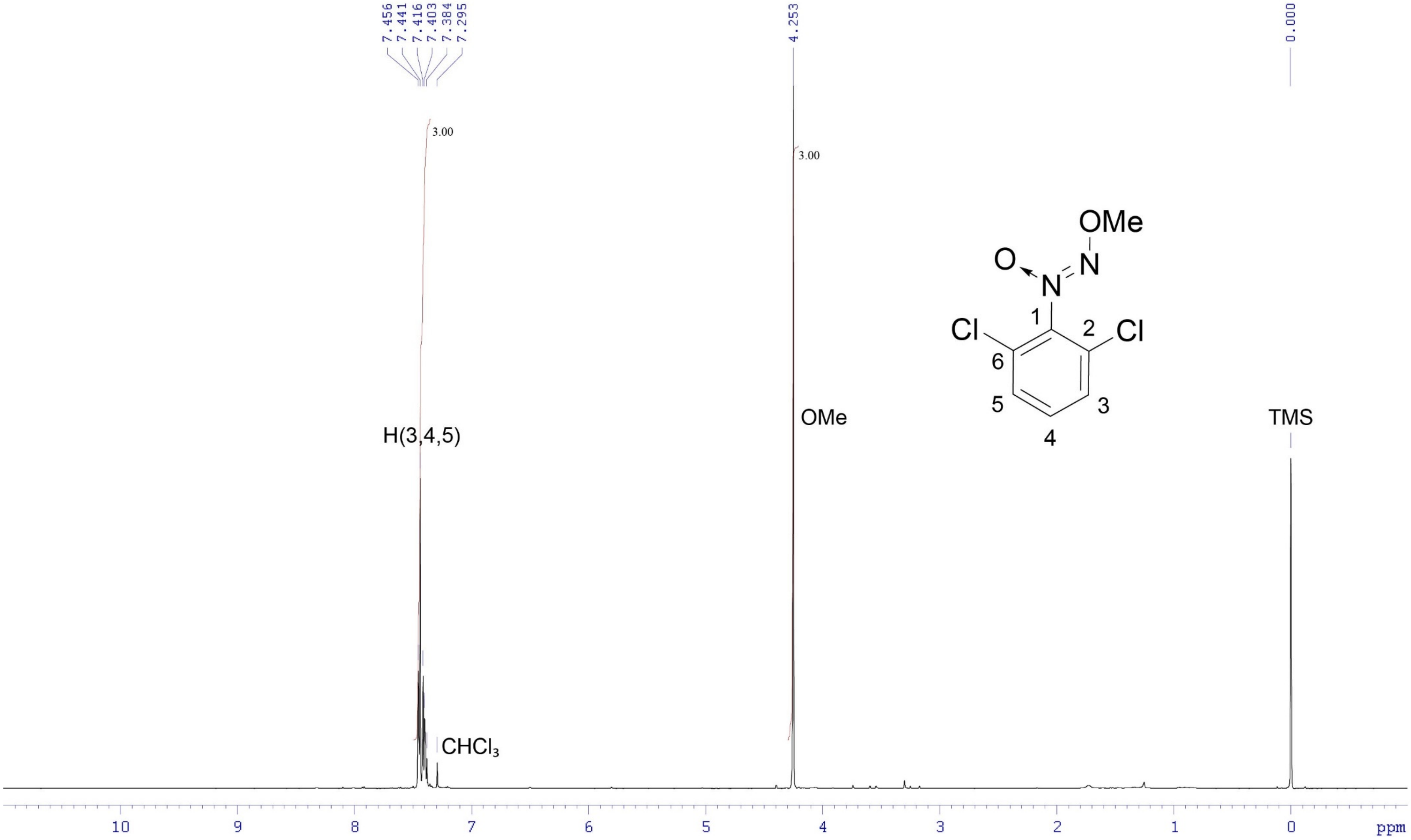

9.8.2 ^{13}C NMR spectrum of compound 2h [125.76 MHz, $CDCl_3$]

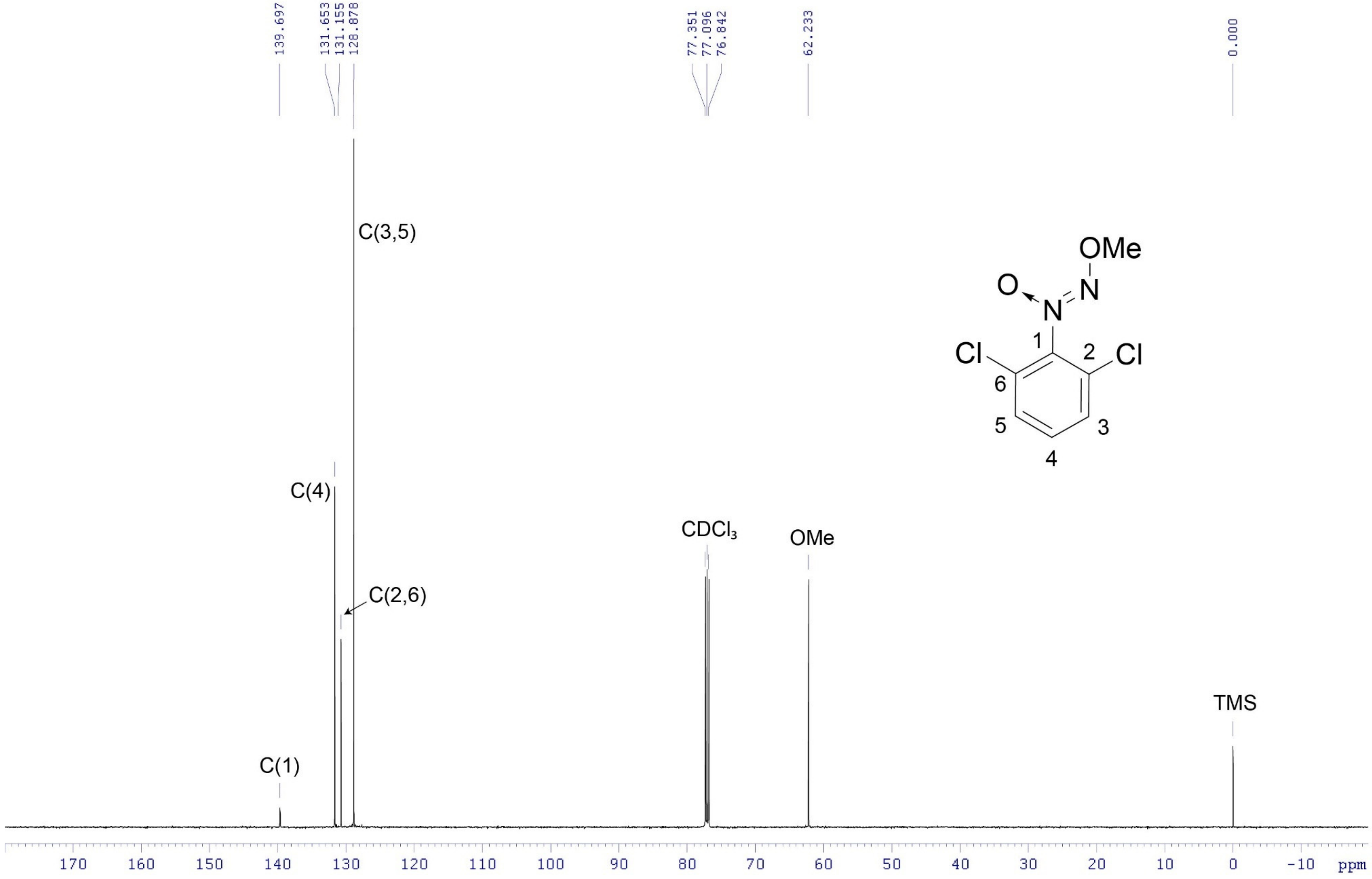

9.8.3 {^1H–^{13}C} HSQC spectrum of compound 2h [500.13 MHz, $CDCl_3$]

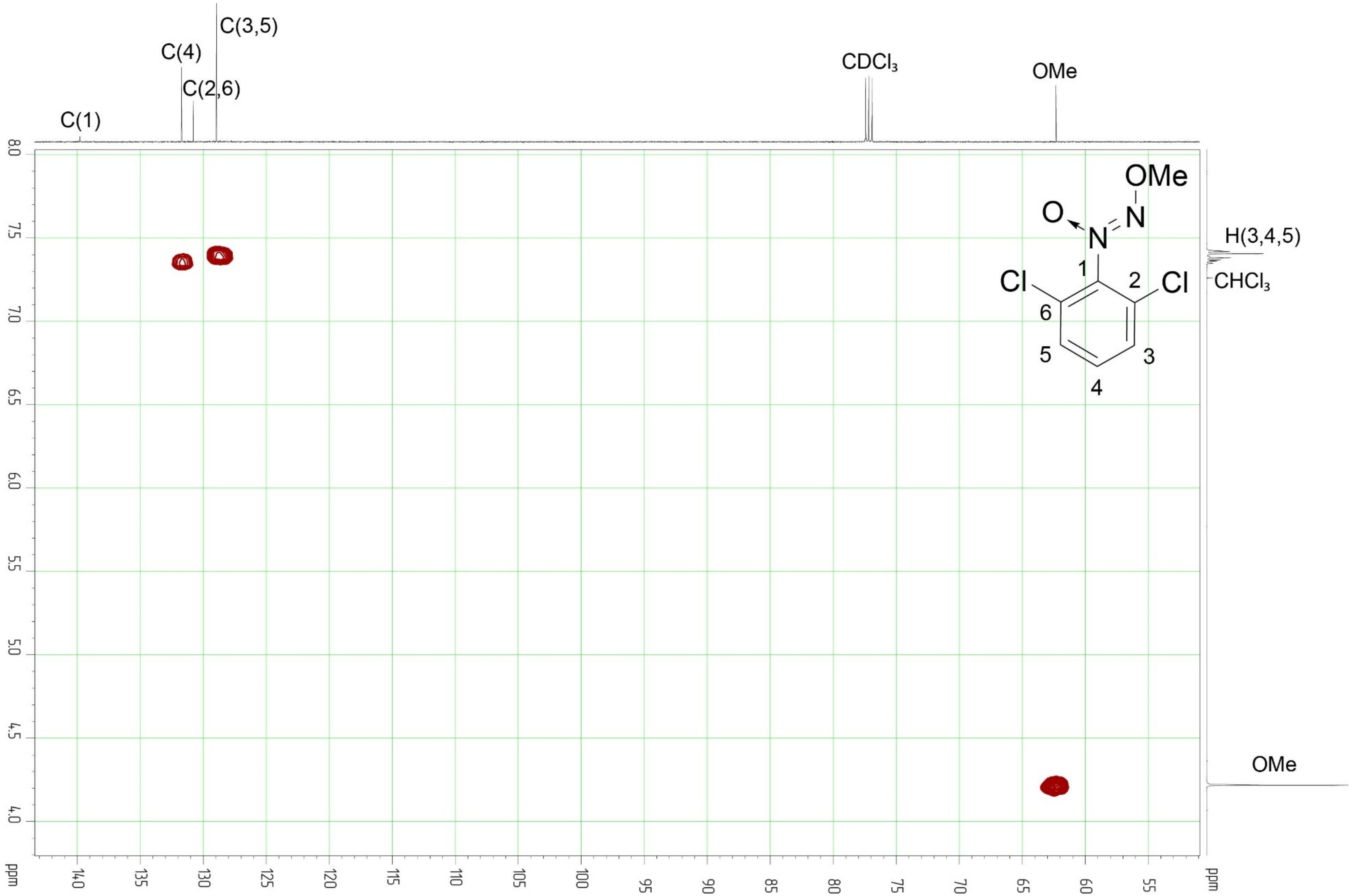

9.8.4 {^1H–^{13}C} HMBC spectrum of compound 2h [500.13 MHz, $CDCl_3$]

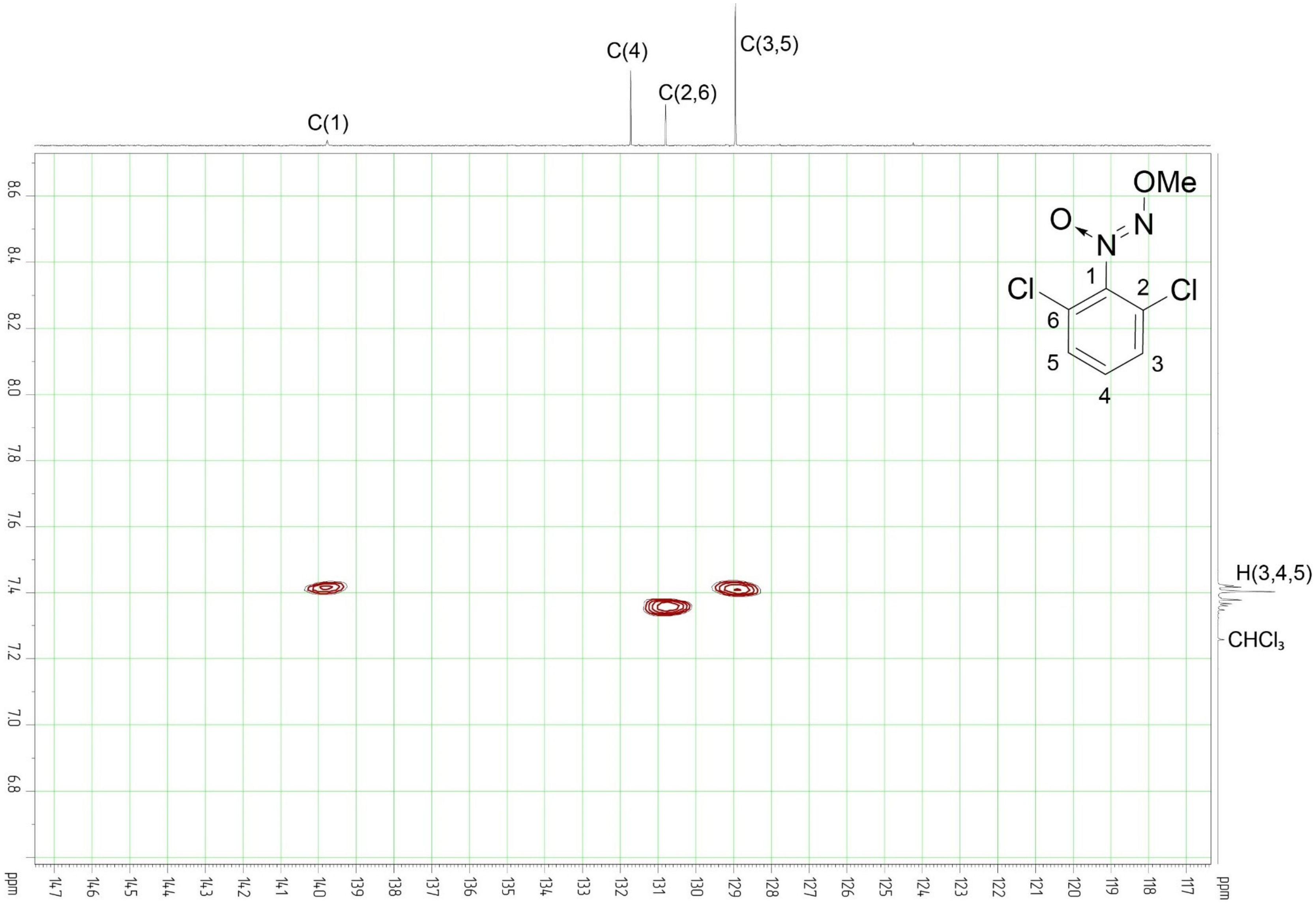

9.8.5 ^{14}N NMR spectrum of compound 2h [36.14 MHz, $CDCl_3$]

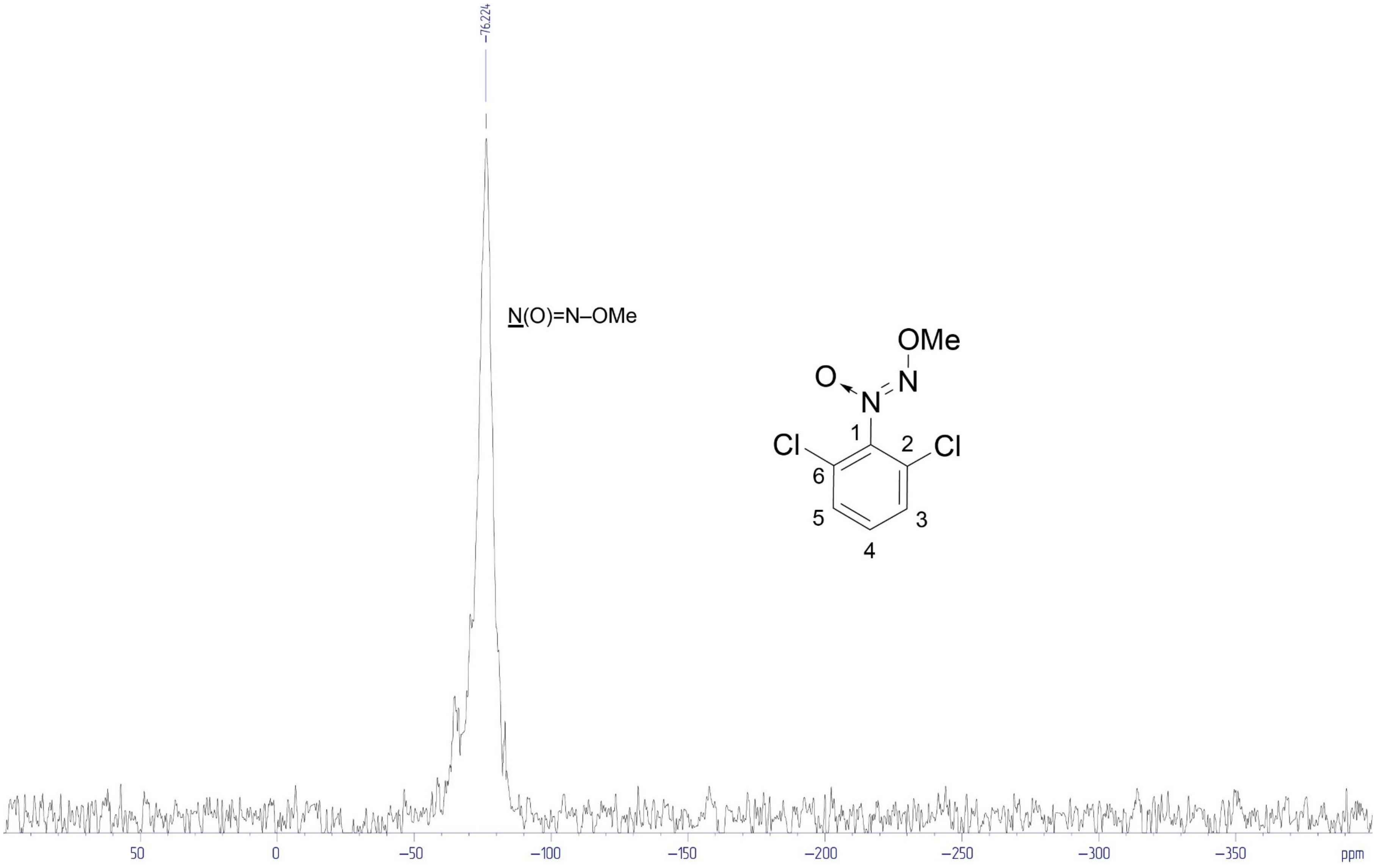

9.9.1 ^{1}H NMR spectrum of compound 2i [500.13 MHz, $CDCl_3$]

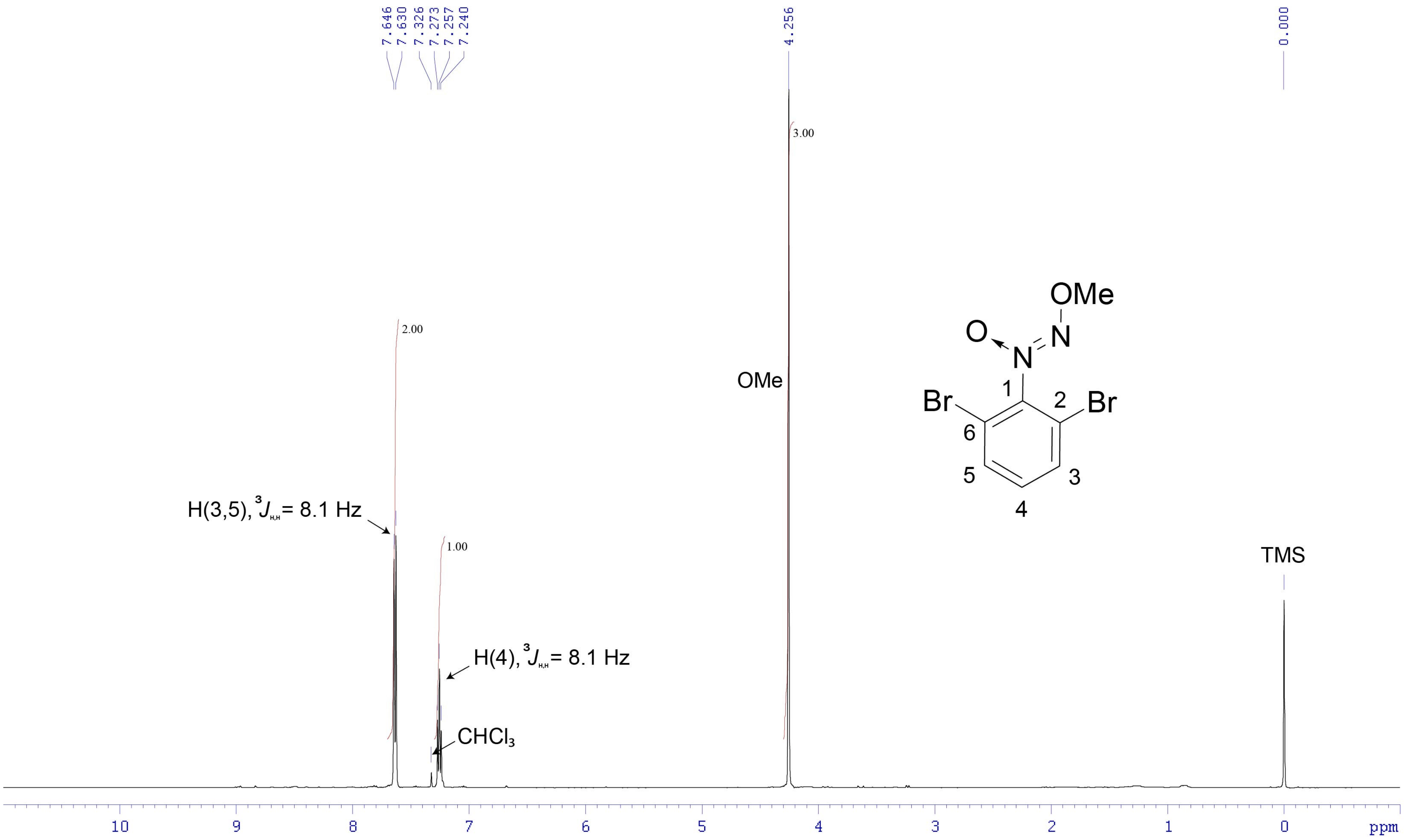

9.9.2 ^{13}C NMR spectrum of compound 2i [125.76 MHz, $CDCl_3$]

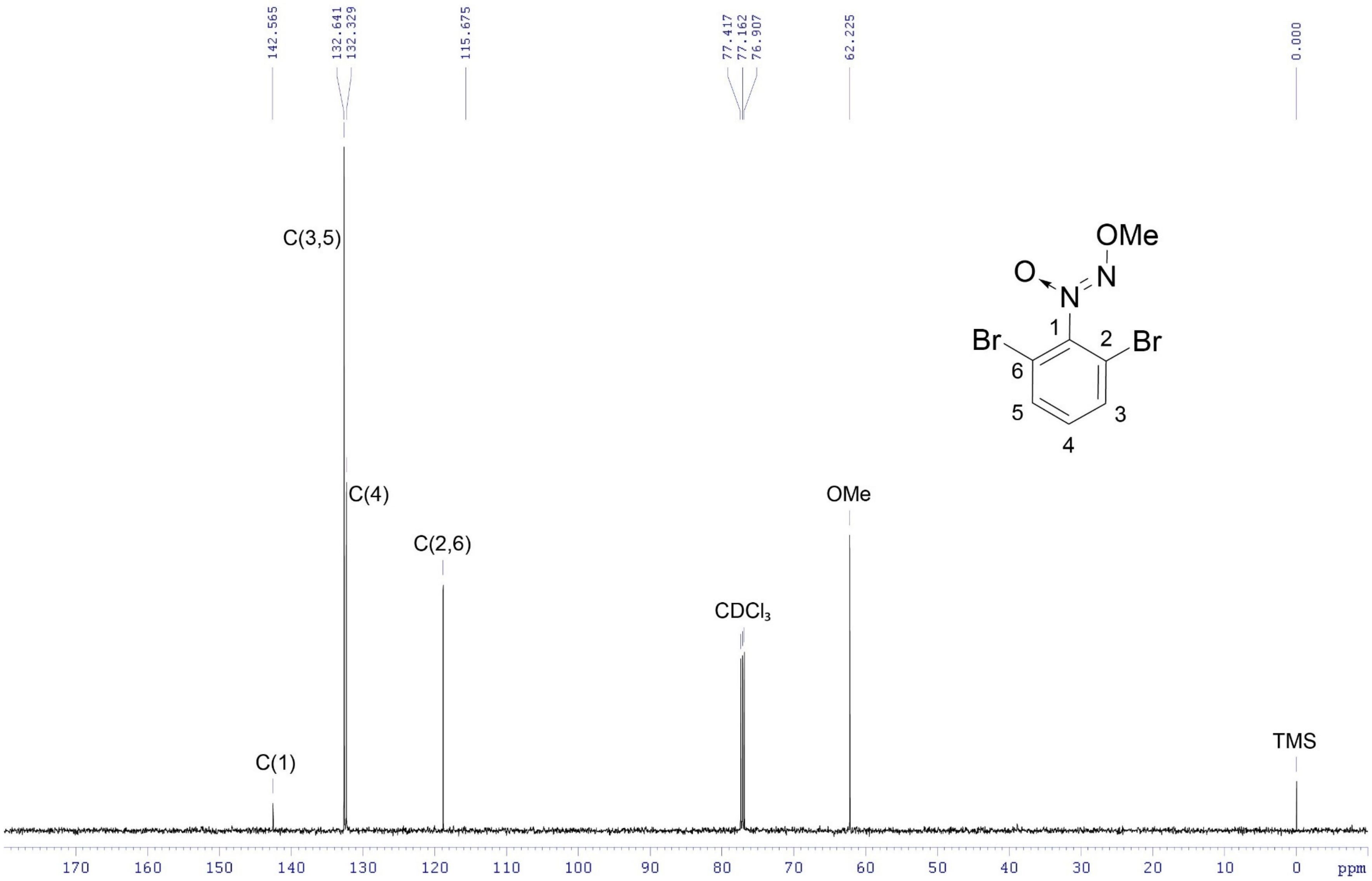

9.9.3 {^{1}H–^{13}C} HSQC spectrum of compound 2i [500.13 MHz, $CDCl_3$]

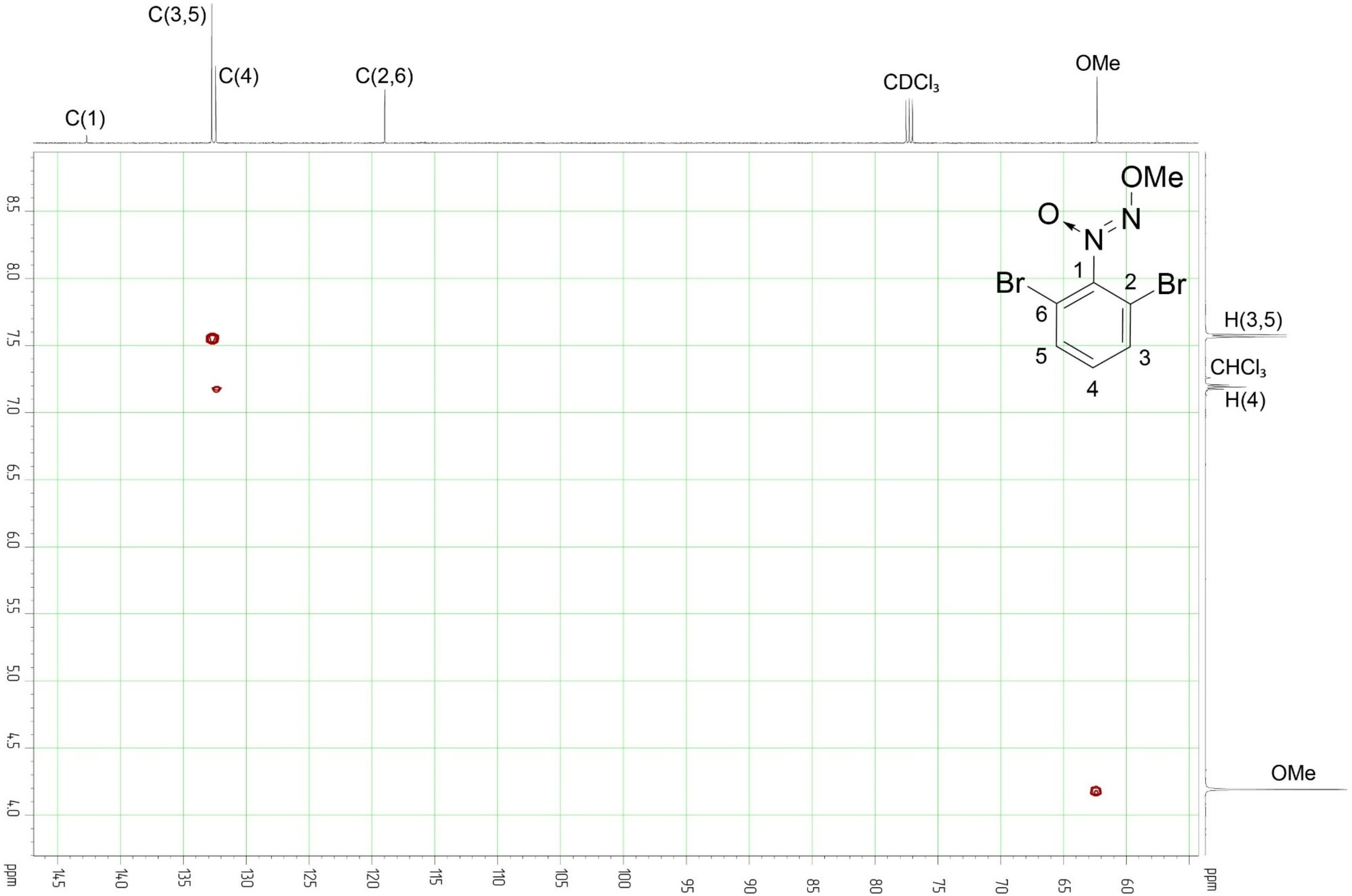

9.9.4 {^{1}H–^{13}C} HMBC spectrum of compound 2i [500.13 MHz, $CDCl_3$]

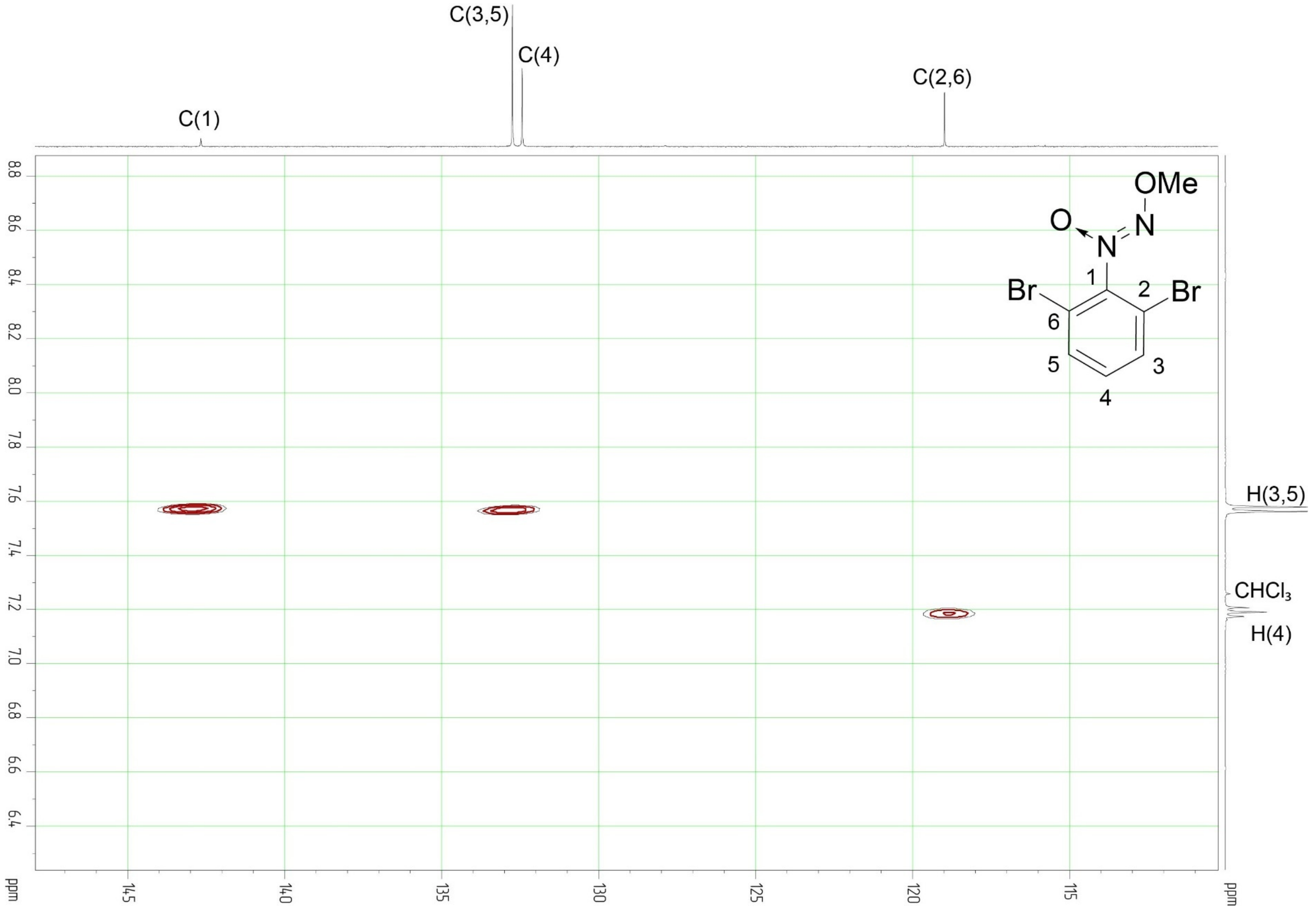

9.9.5 ^{14}N NMR spectrum of compound 2i [36.14 MHz, $CDCl_3$]

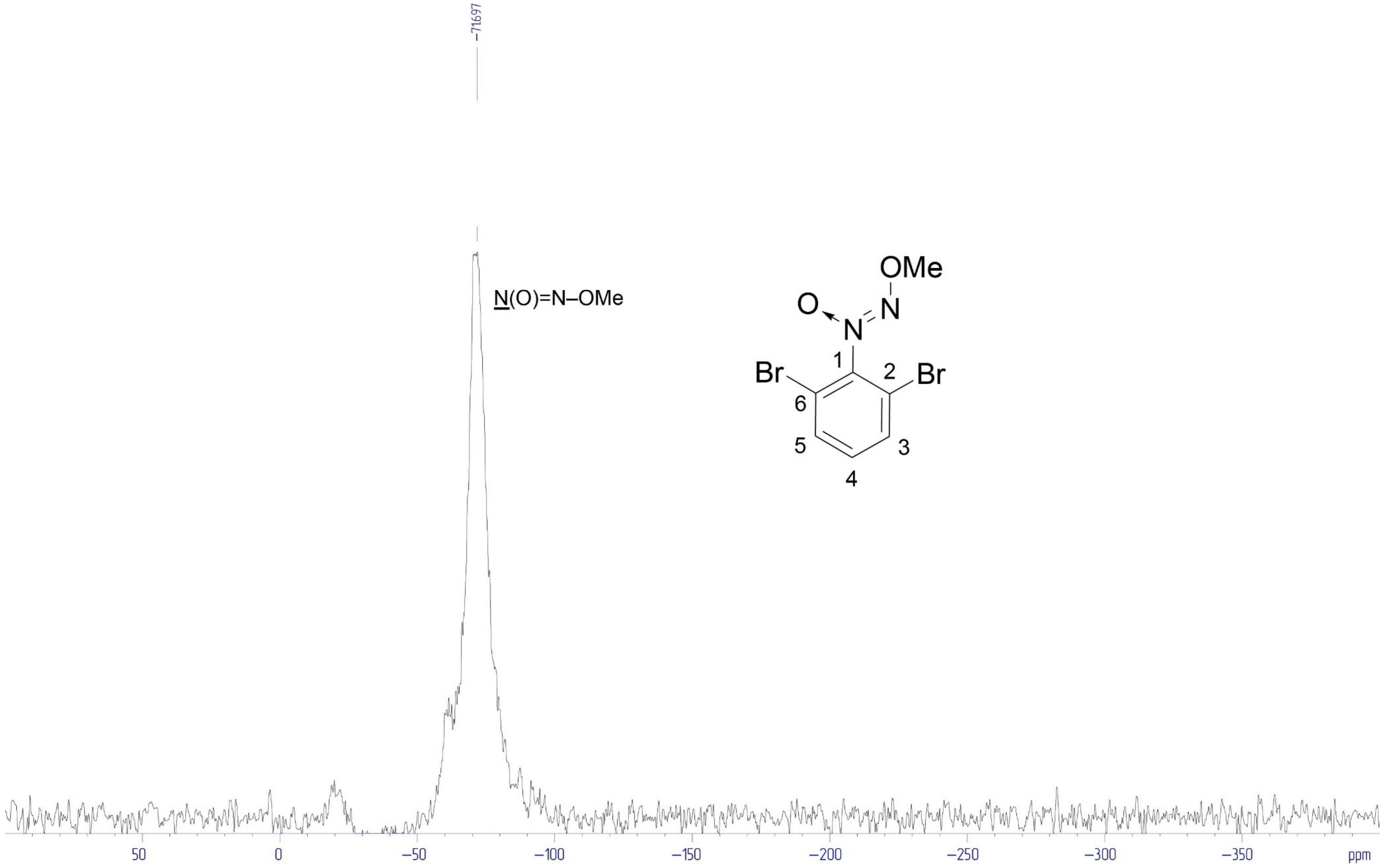

9.10.1 ^{1}H NMR spectrum of compound 2j [500.13 MHz, $CDCl_3$]

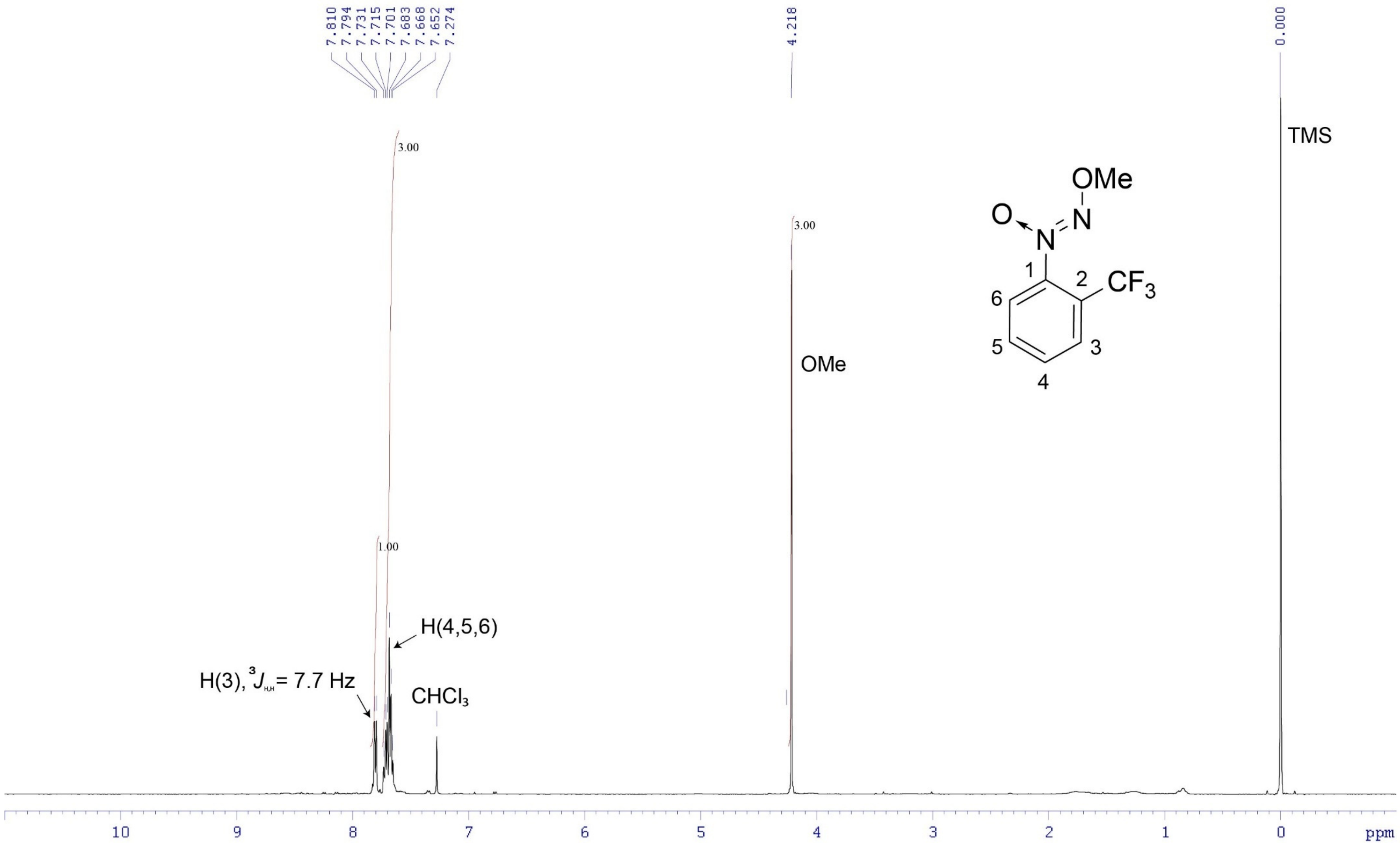

9.10.2 ^{13}C NMR spectrum of compound 2j [125.76 MHz, $CDCl_3$]

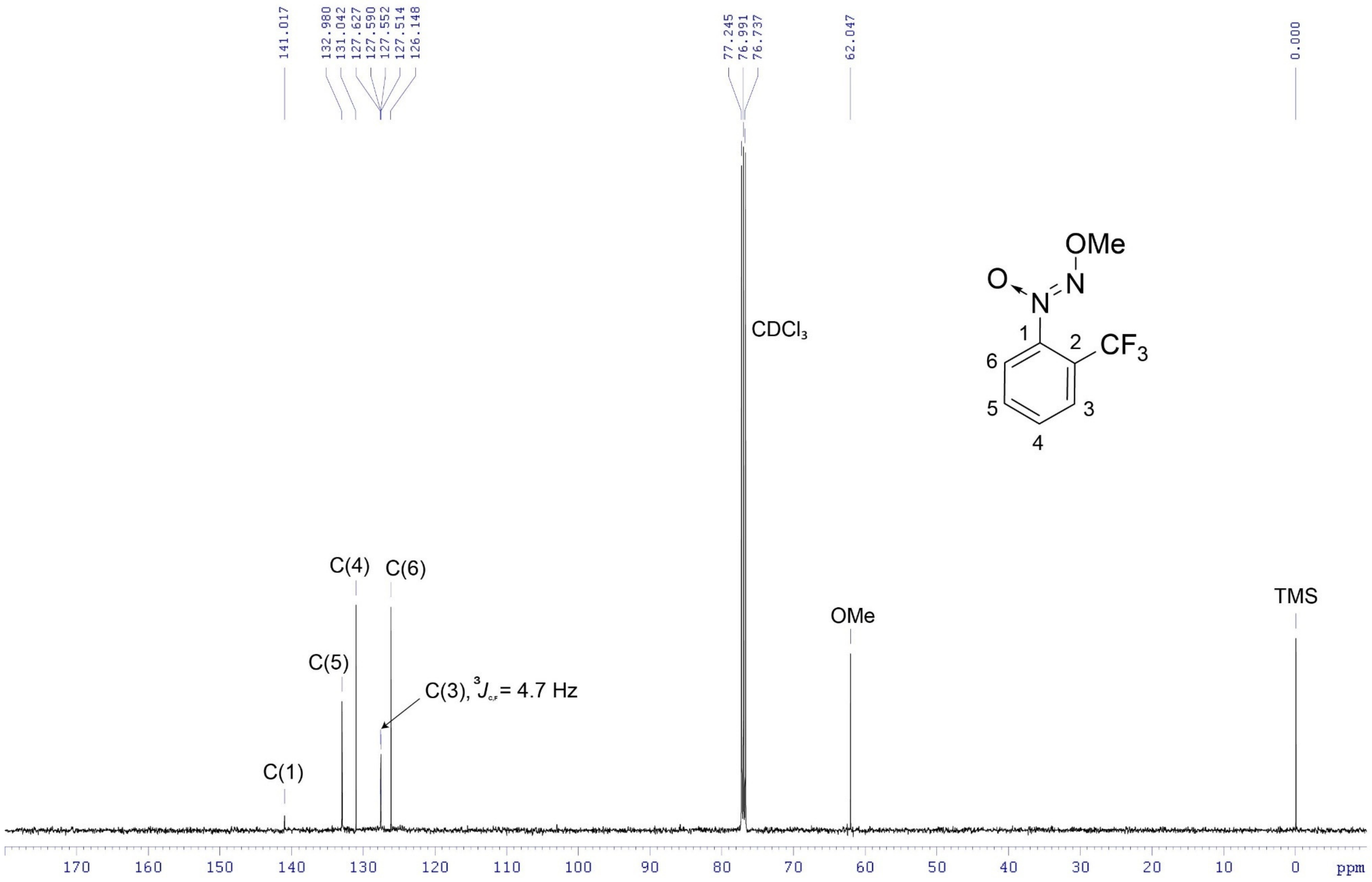

9.10.3 {^{1}H–^{13}C} HSQC spectrum of compound 2j [500.13 MHz, $CDCl_3$]

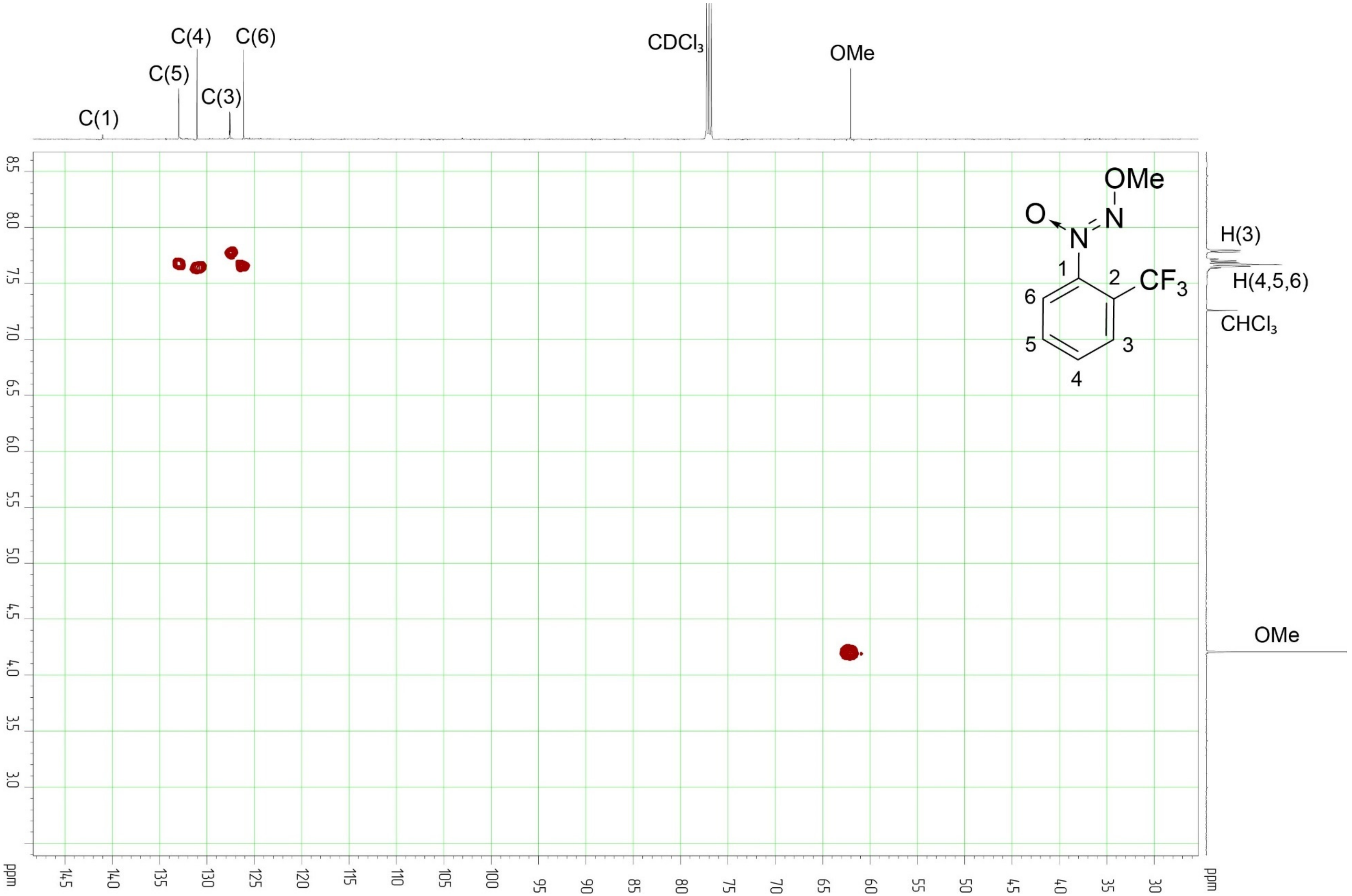

9.10.4 {^{1}H–^{13}C} HMBC spectrum of compound 2j [500.13 MHz, $CDCl_3$]

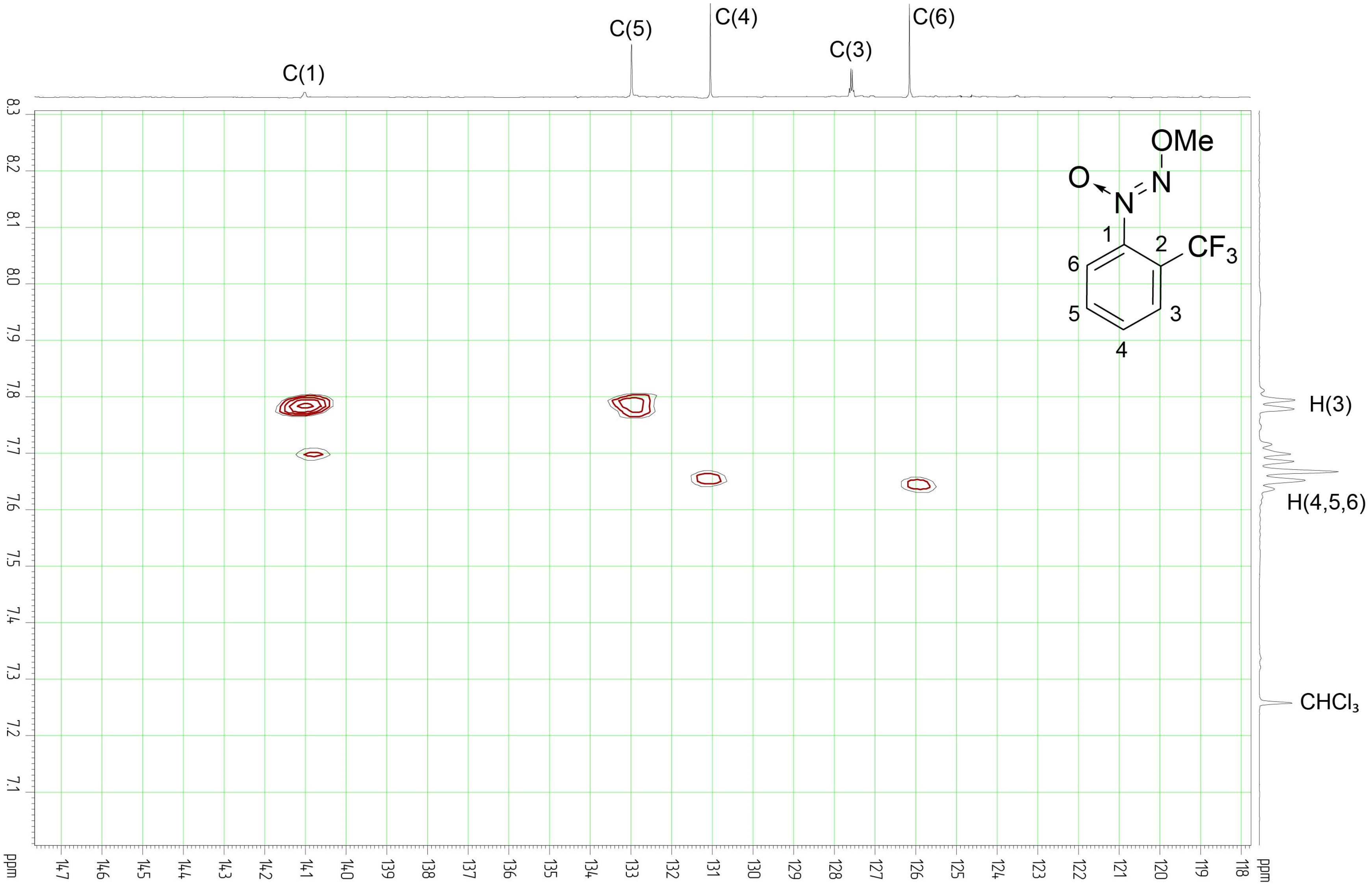

9.10.5 ^{14}N NMR spectrum of compound 2j [36.14 MHz, $CDCl_3$]

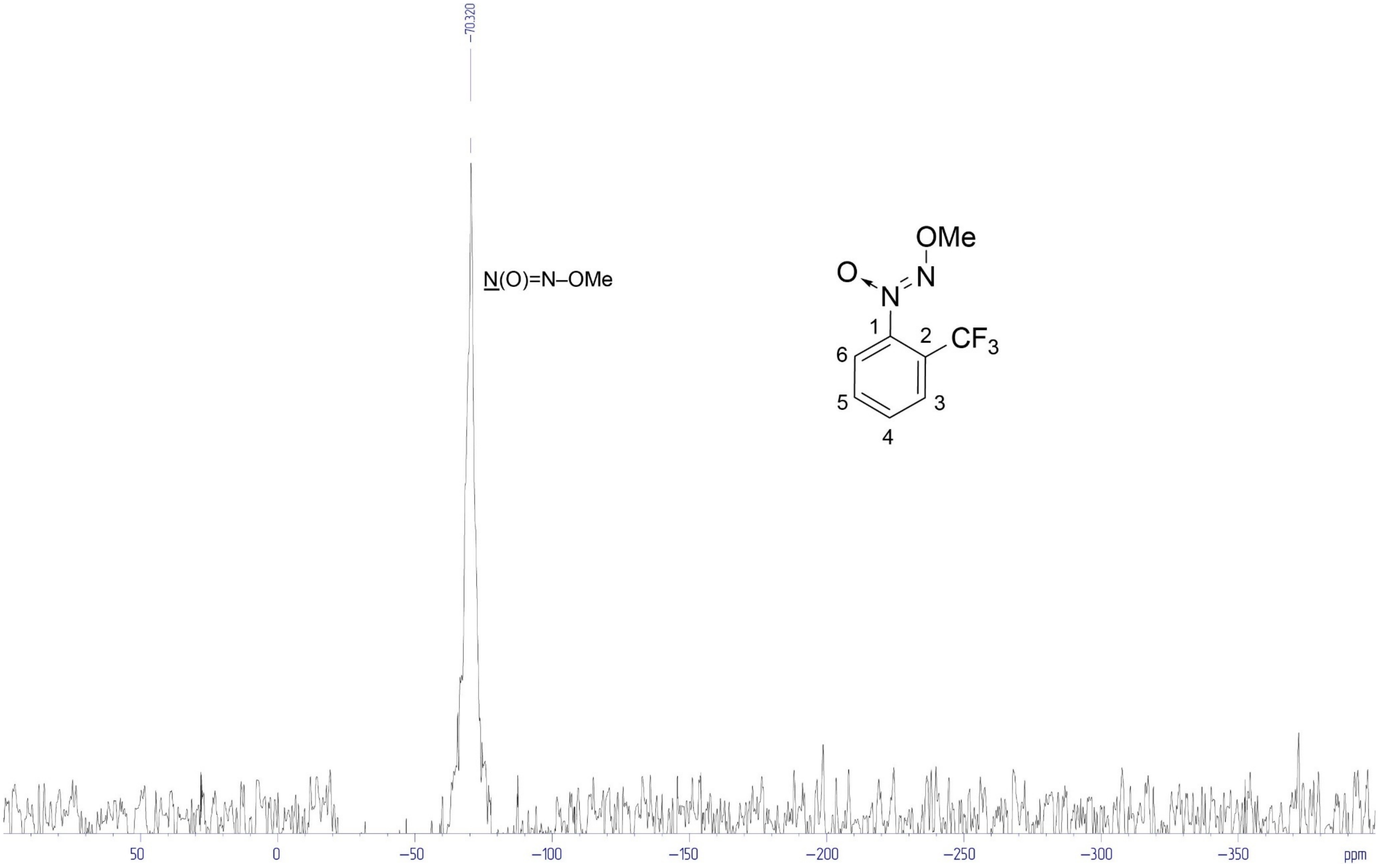

9.10.6 ^{19}F NMR spectrum of compound 2j [470.59 MHz, $CDCl_3$]

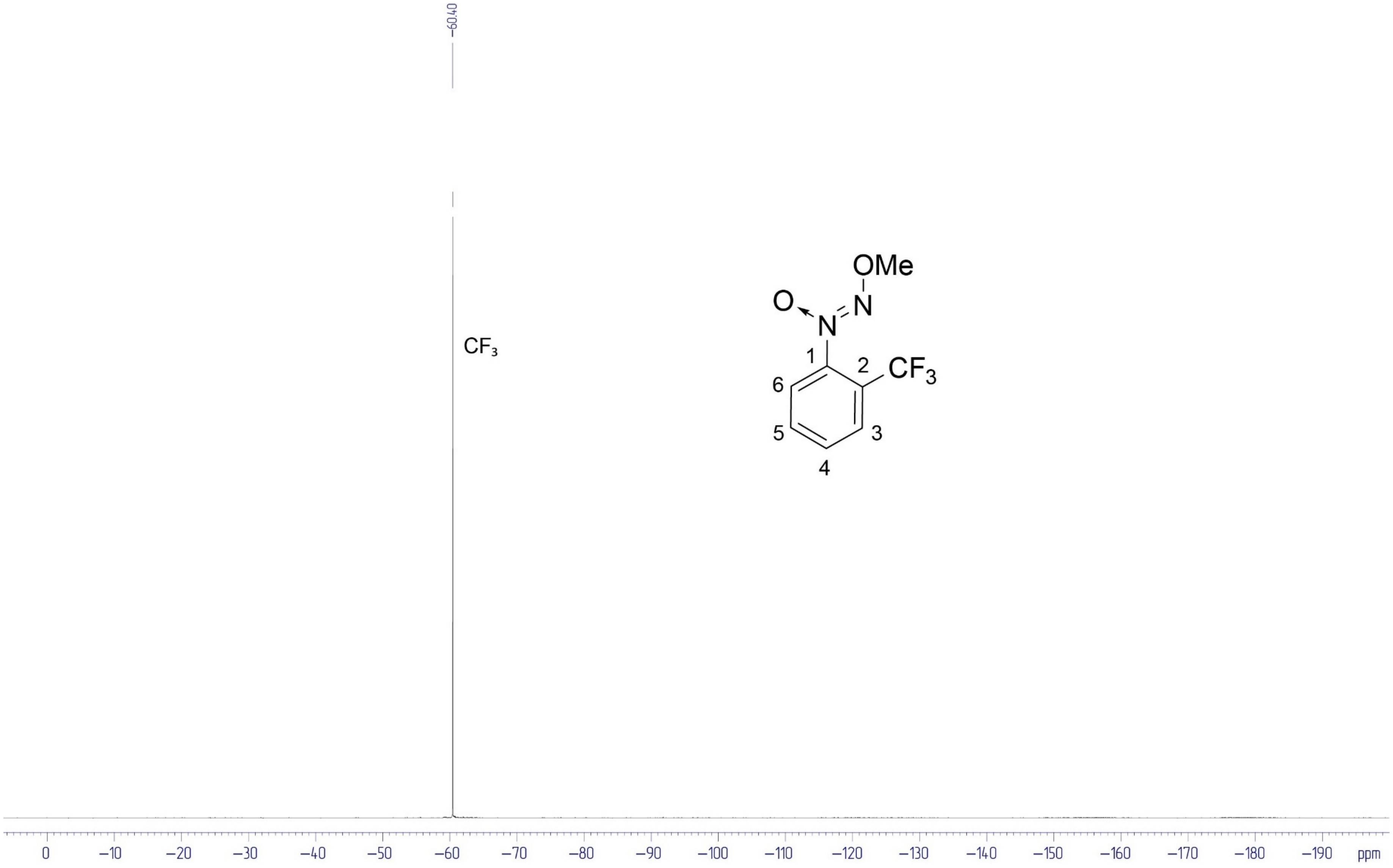

9.11.1 ^{1}H NMR spectrum of compound 2k [500.13 MHz, $CDCl_3$]

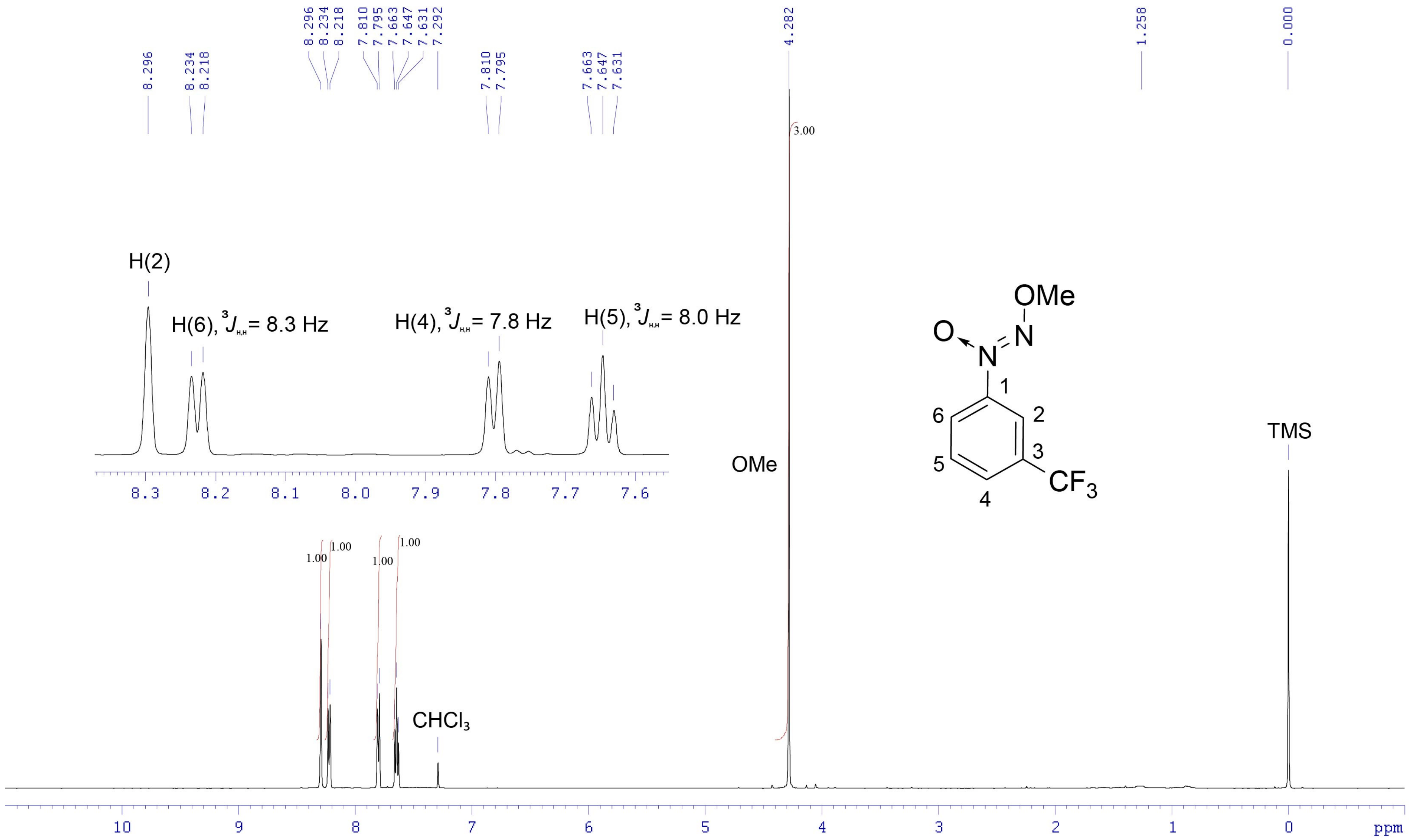

9.11.2 ^{13}C NMR spectrum of compound 2k [125.76 MHz, $CDCl_3$]

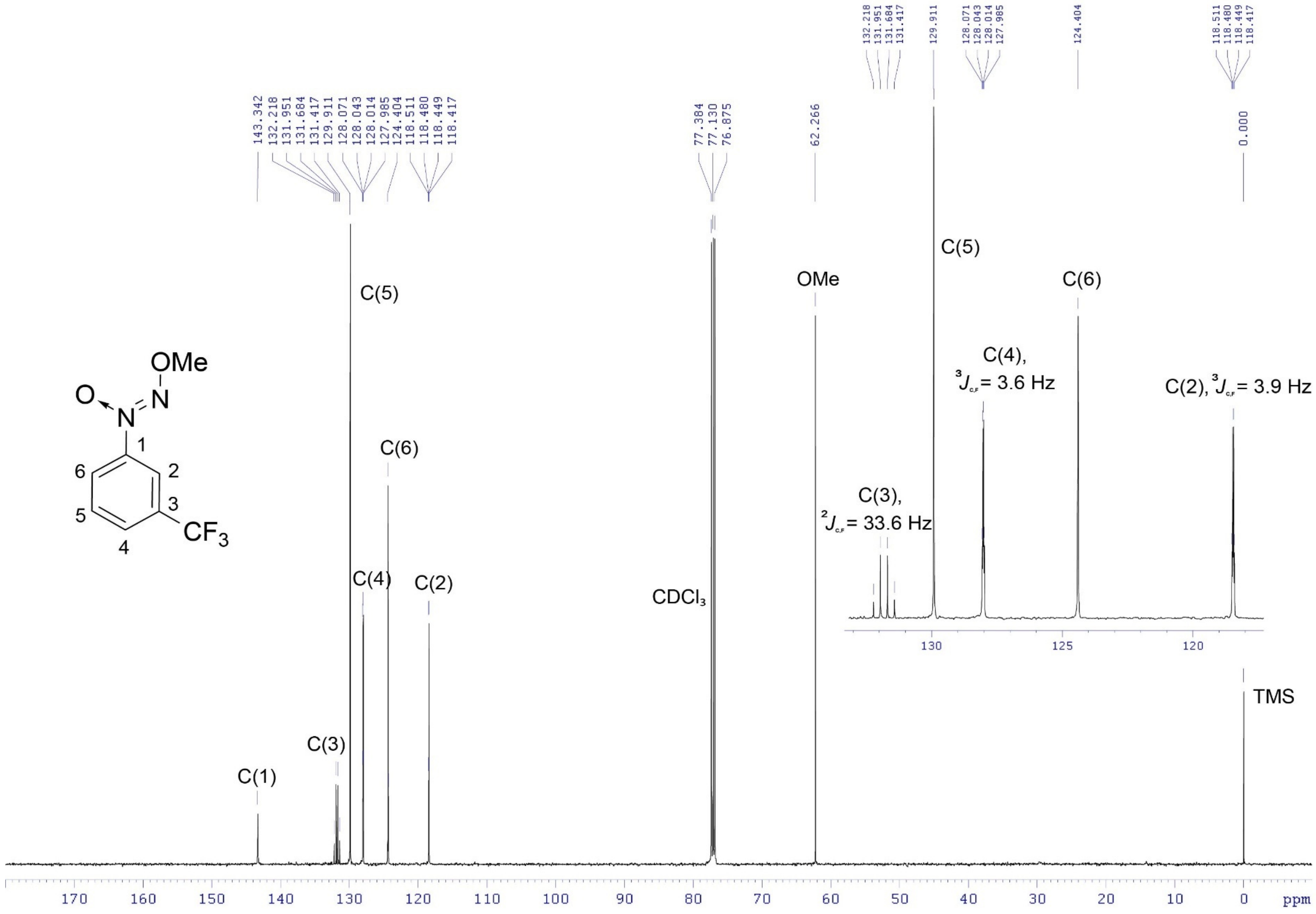

9.11.3 {^{1}H–^{13}C} HSQC spectrum of compound 2k [500.13 MHz, $CDCl_3$]

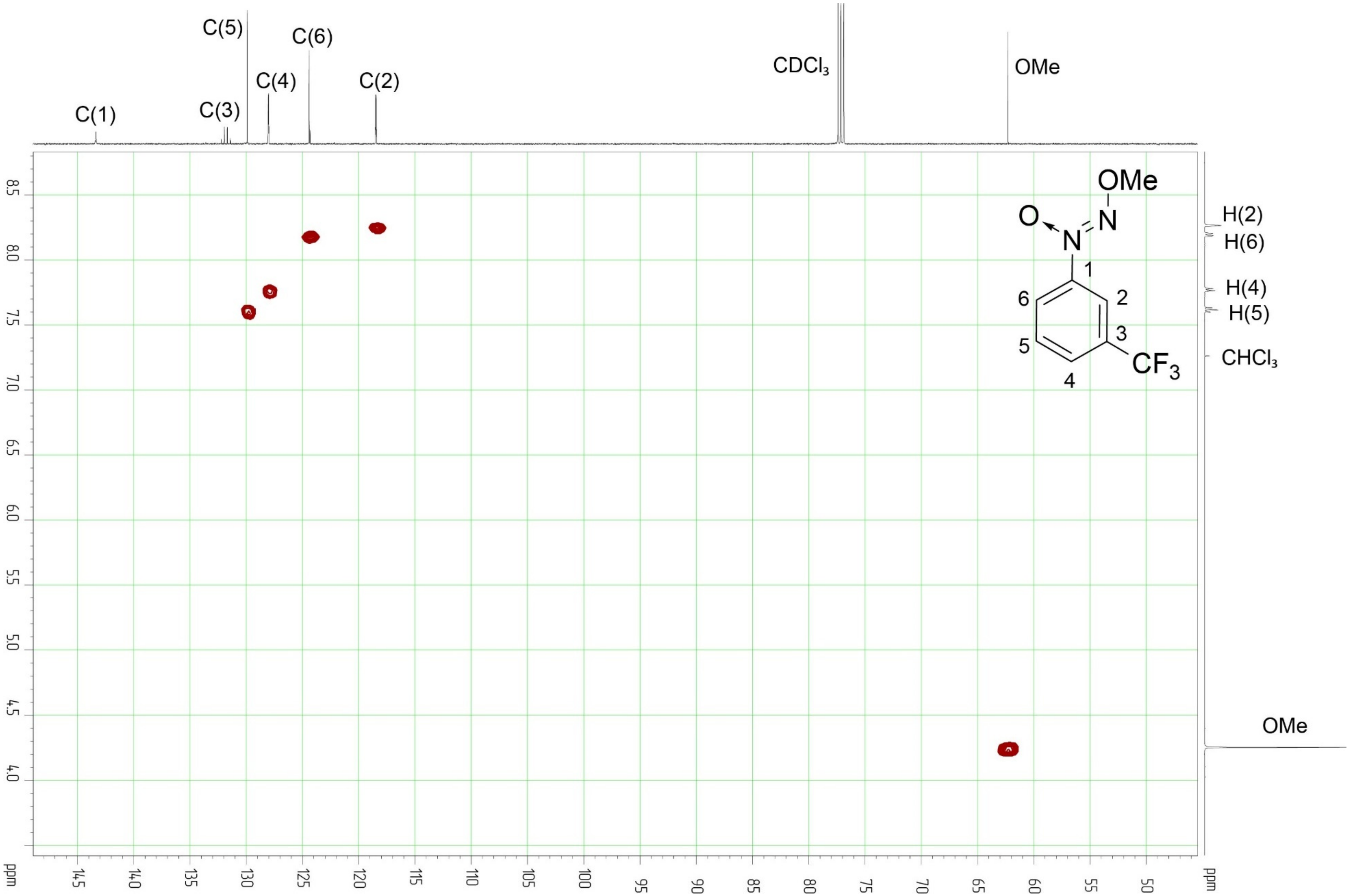

9.11.4 {^{1}H–^{13}C} HMBC spectrum of compound 2k [500.13 MHz, $CDCl_3$]

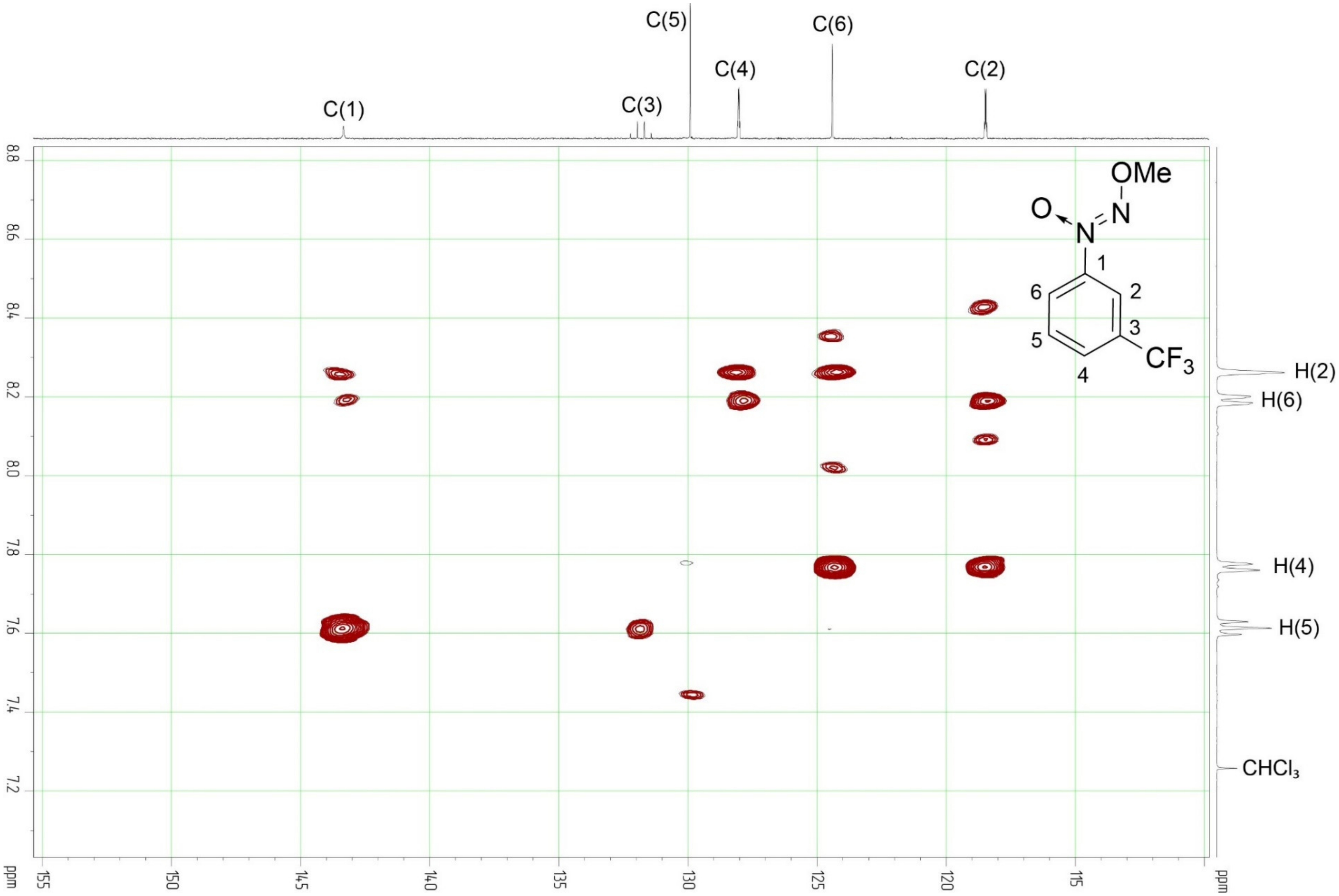

9.11.5 ^{14}N NMR spectrum of compound 2k [36.14 MHz, $CDCl_3$]

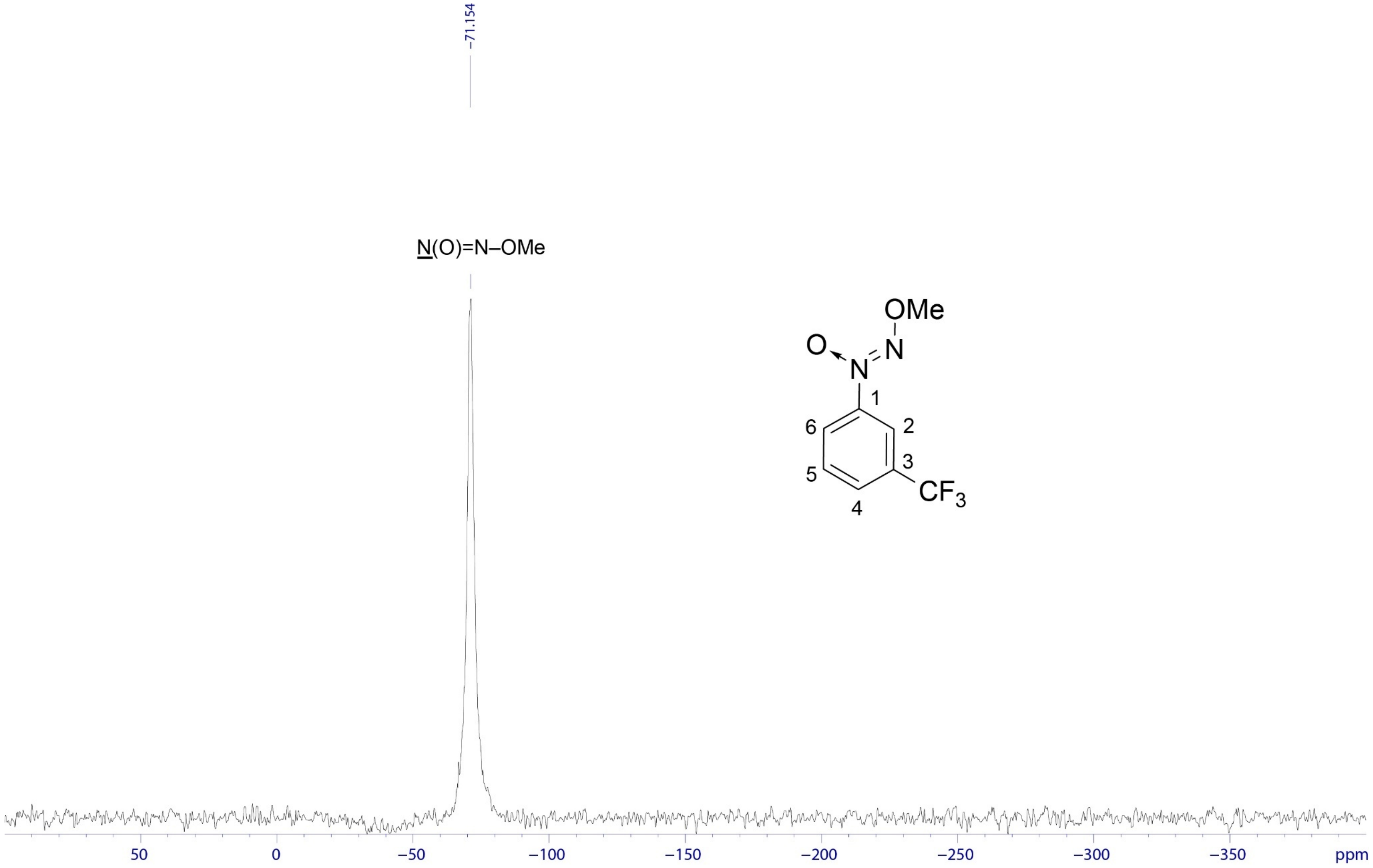

9.11.6 ^{19}F NMR spectrum of compound 2k [470.59 MHz, $CDCl_3$]

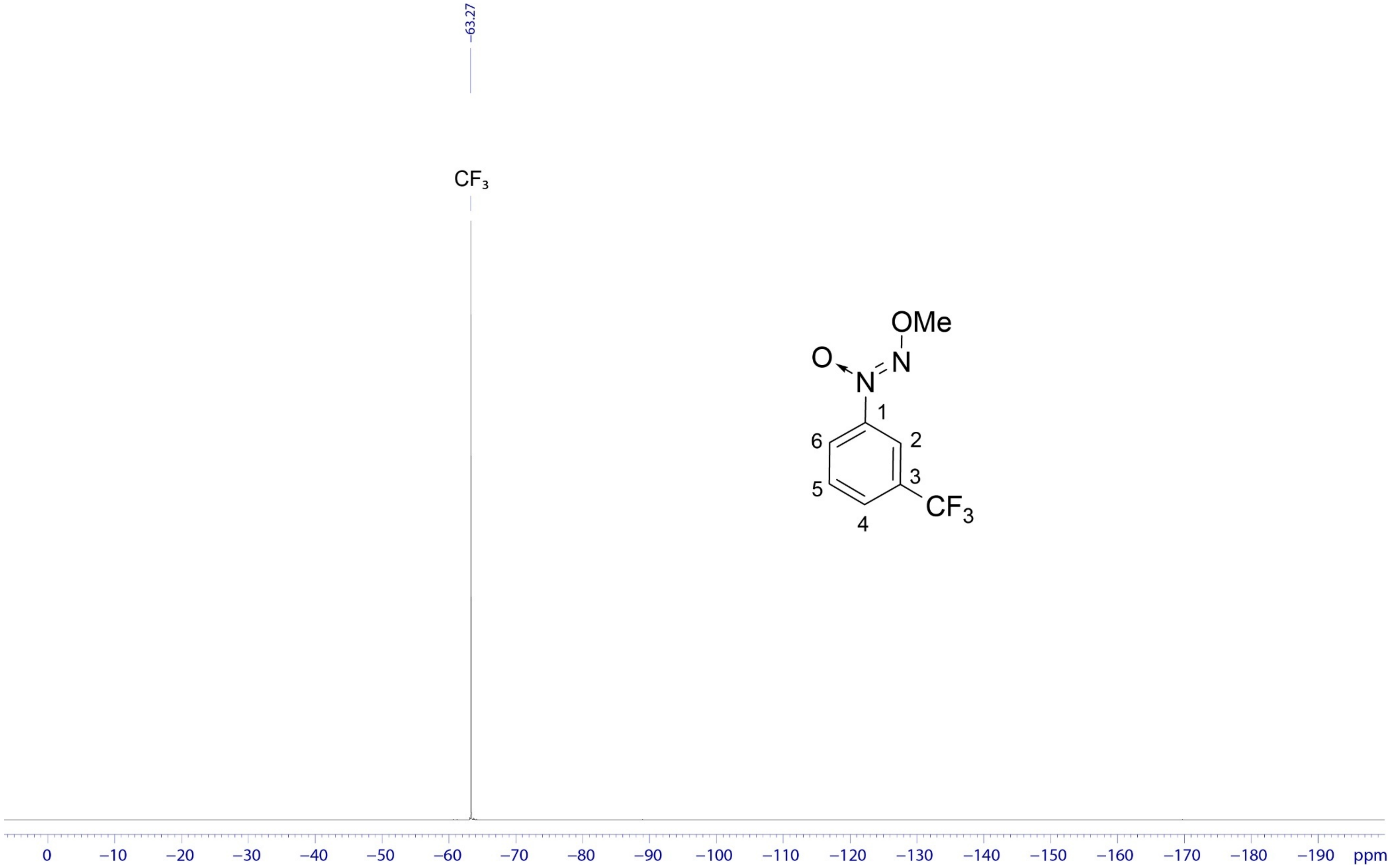

9.12.1 ^{1}H NMR spectrum of compound 2l [500.13 MHz, $CDCl_3$]

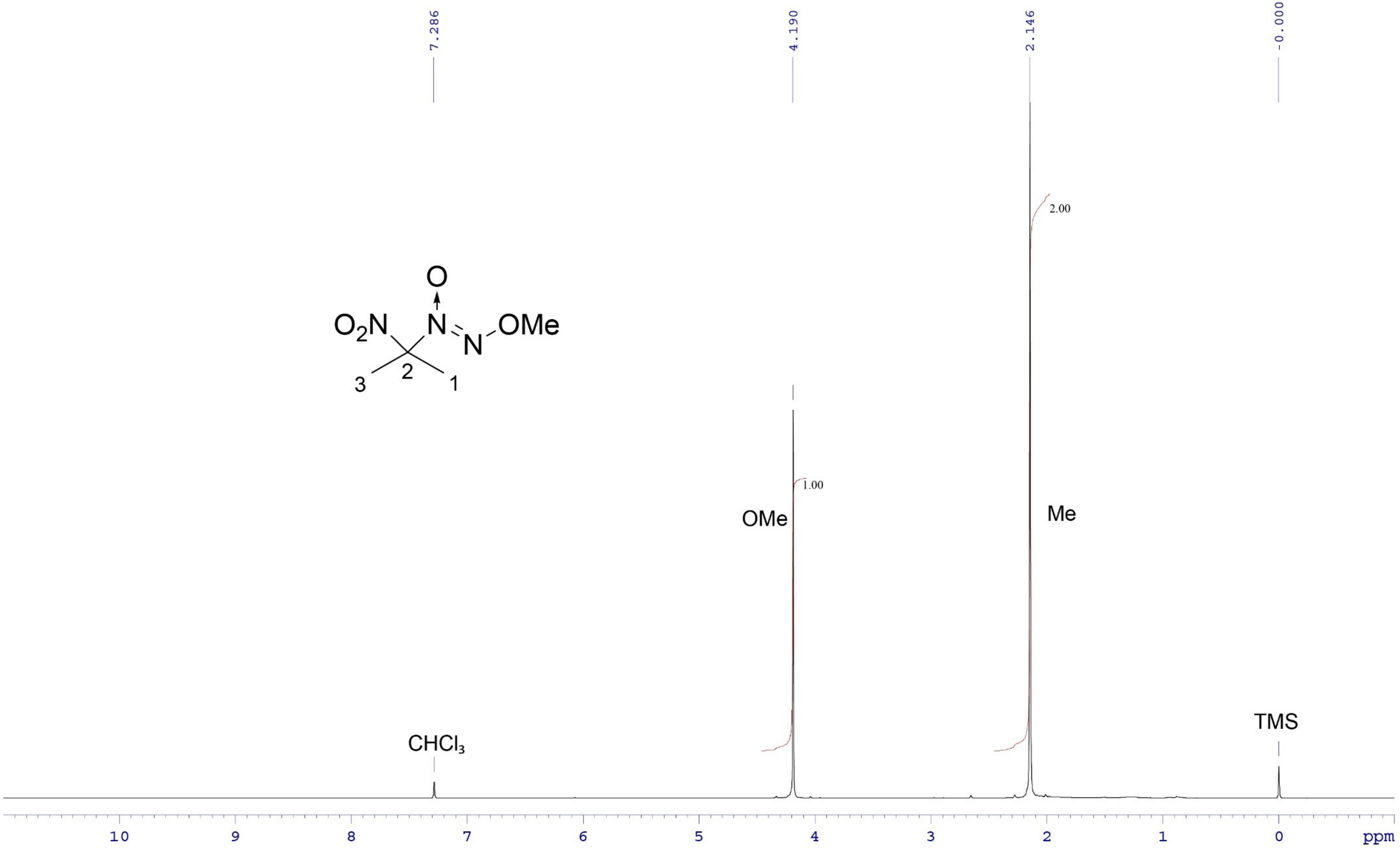

9.12.2 ^{13}C NMR spectrum of compound 2l [125.76 MHz, $CDCl_3$]

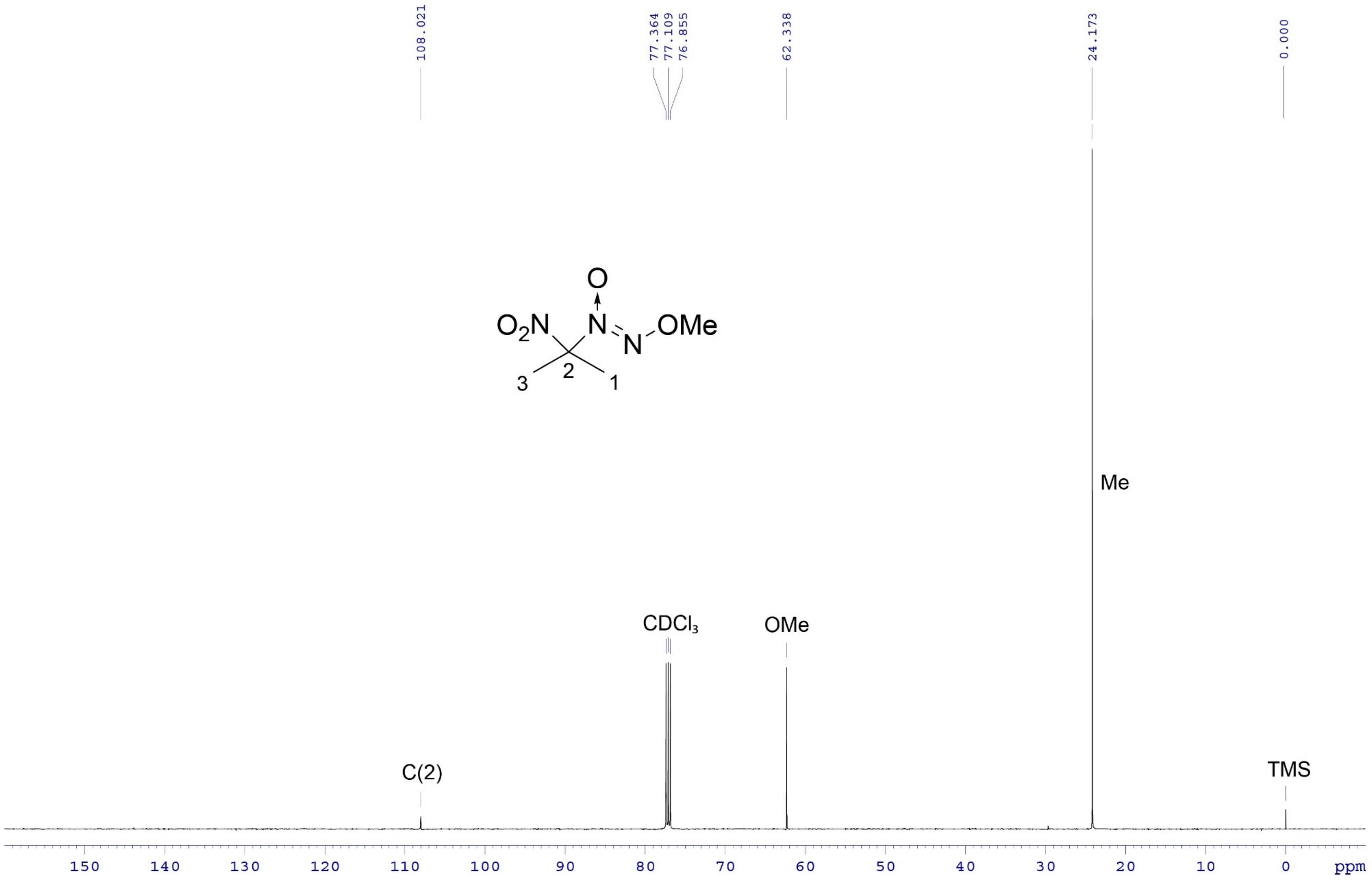

9.12.3 {^{1}H–^{13}C} HSQC spectrum of compound 2l [500.13 MHz, $CDCl_3$]

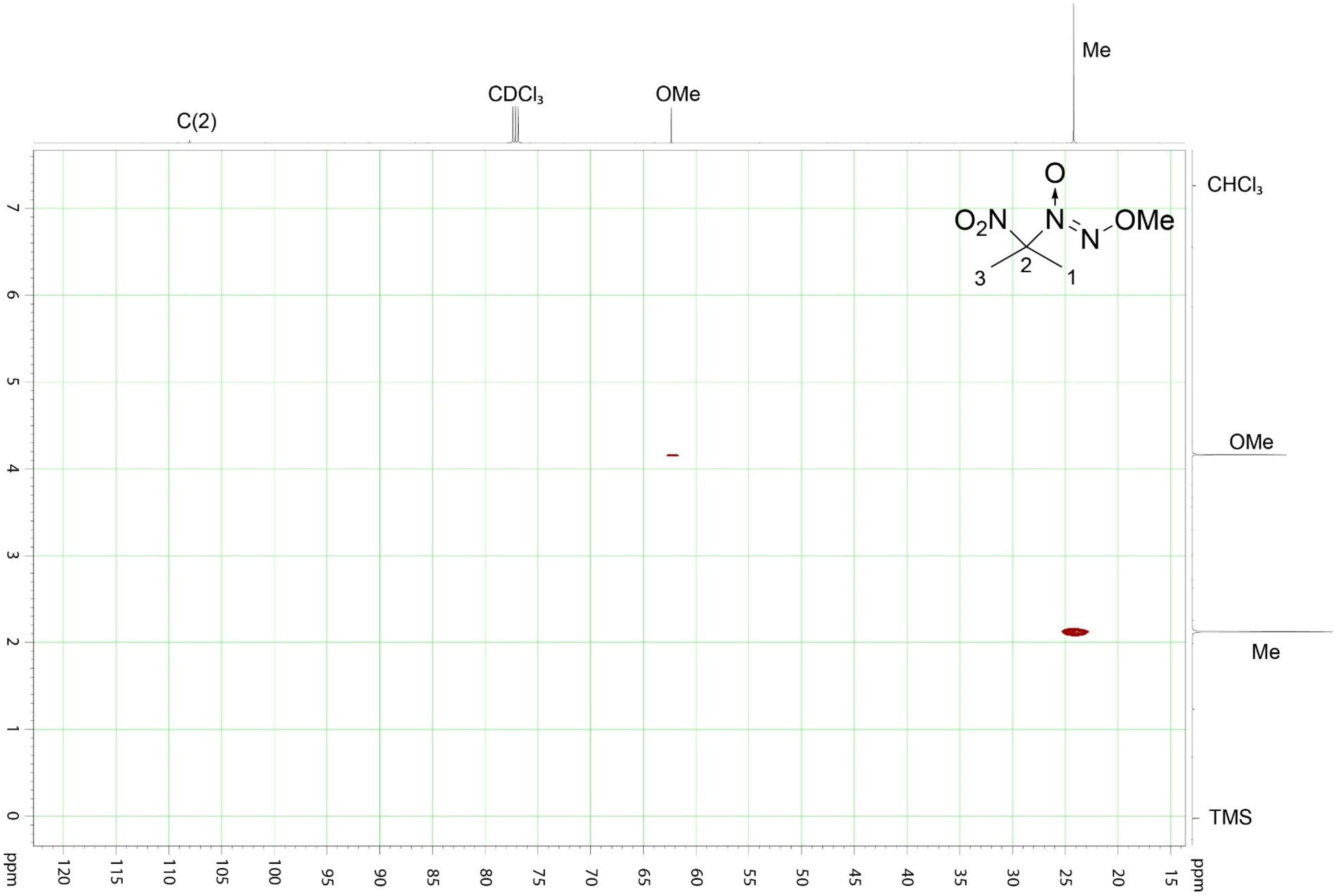

9.12.4 {^{1}H–^{13}C} HMBC spectrum of compound 2l [500.13 MHz, $CDCl_3$]

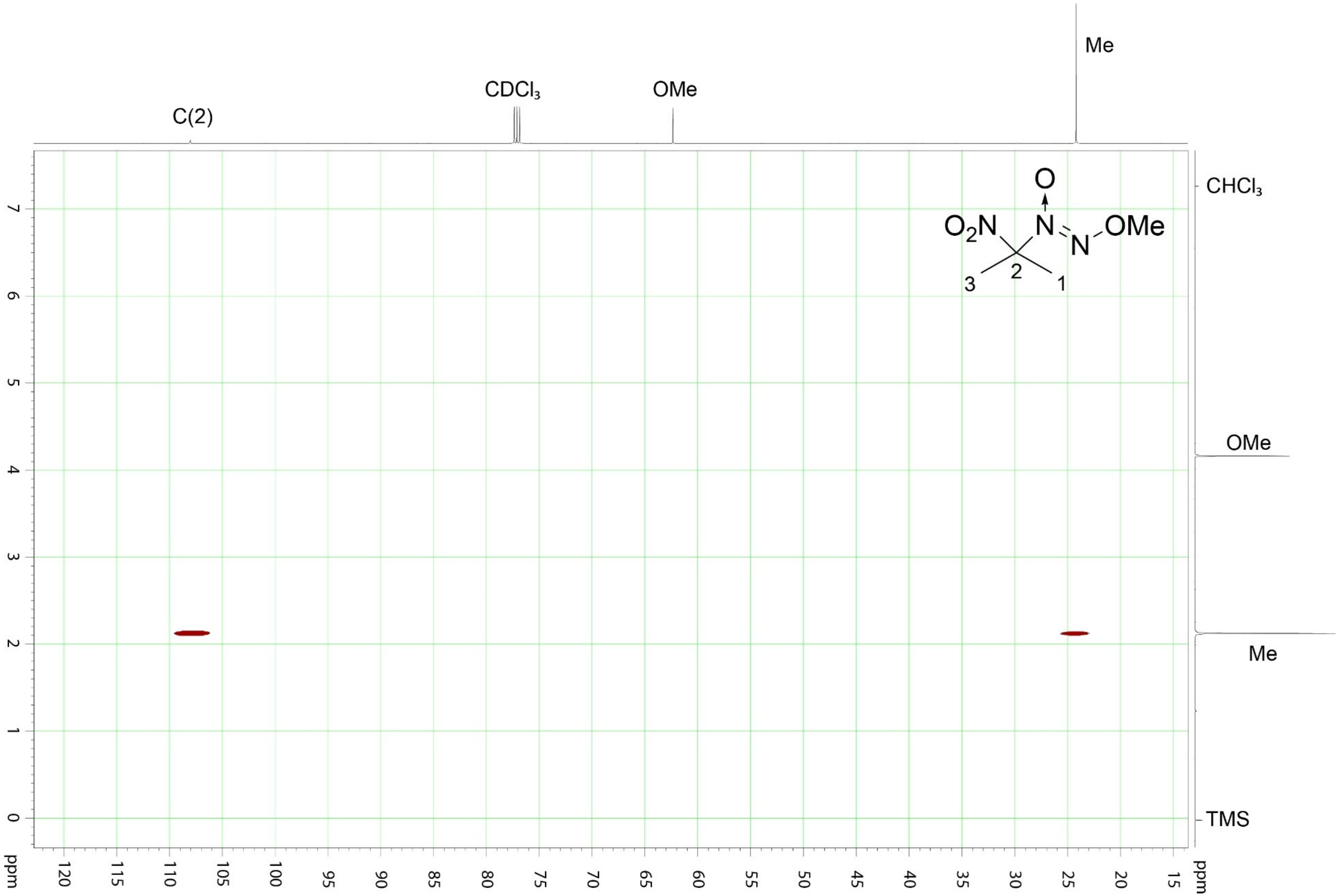

9.12.5 ^{14}N NMR spectrum of compound 2l [36.14 MHz, $CDCl_3$]

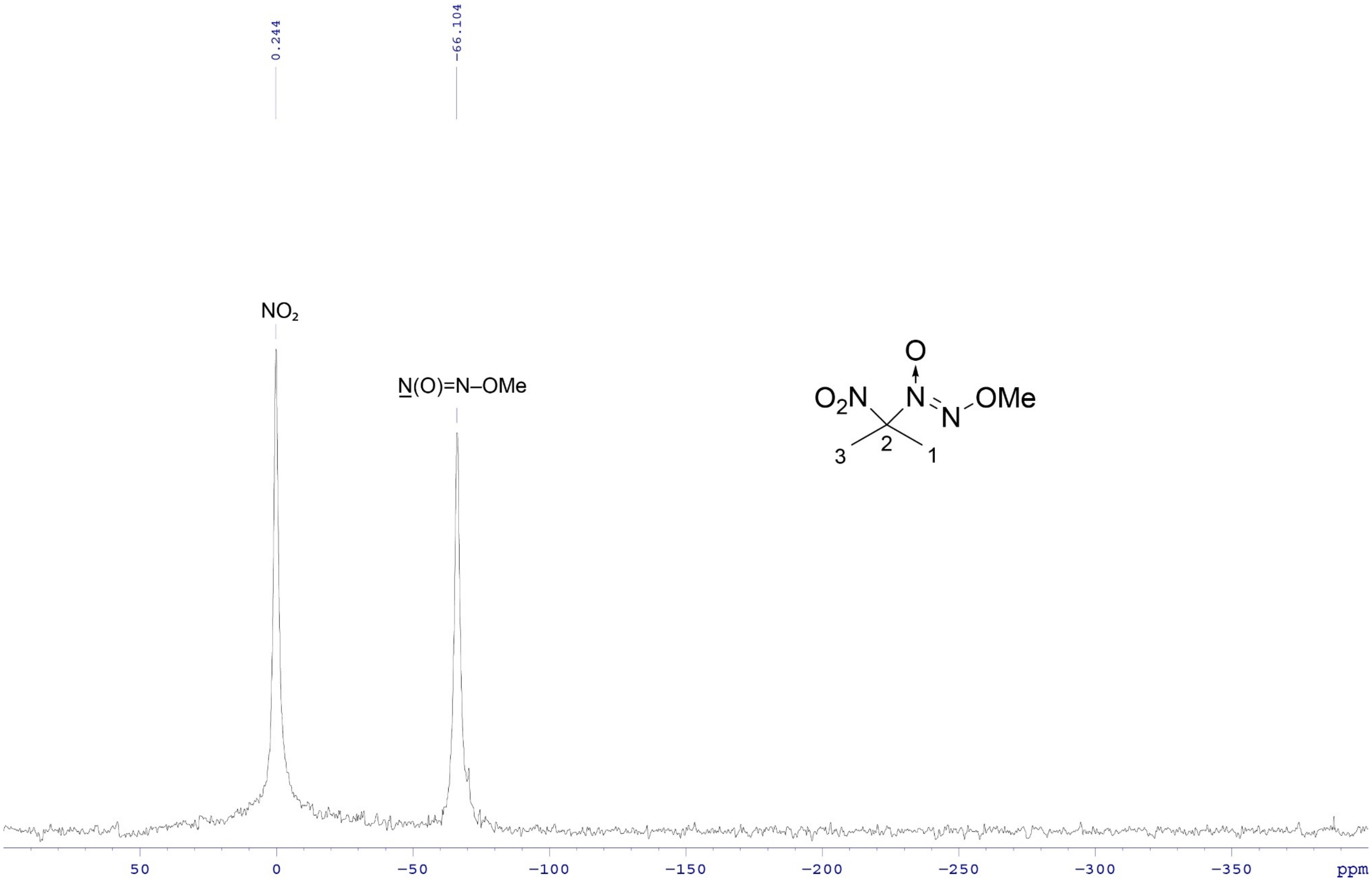

9.13.1 ^{1}H NMR spectrum of compound 2m [600.13 MHz, $CDCl_3$]

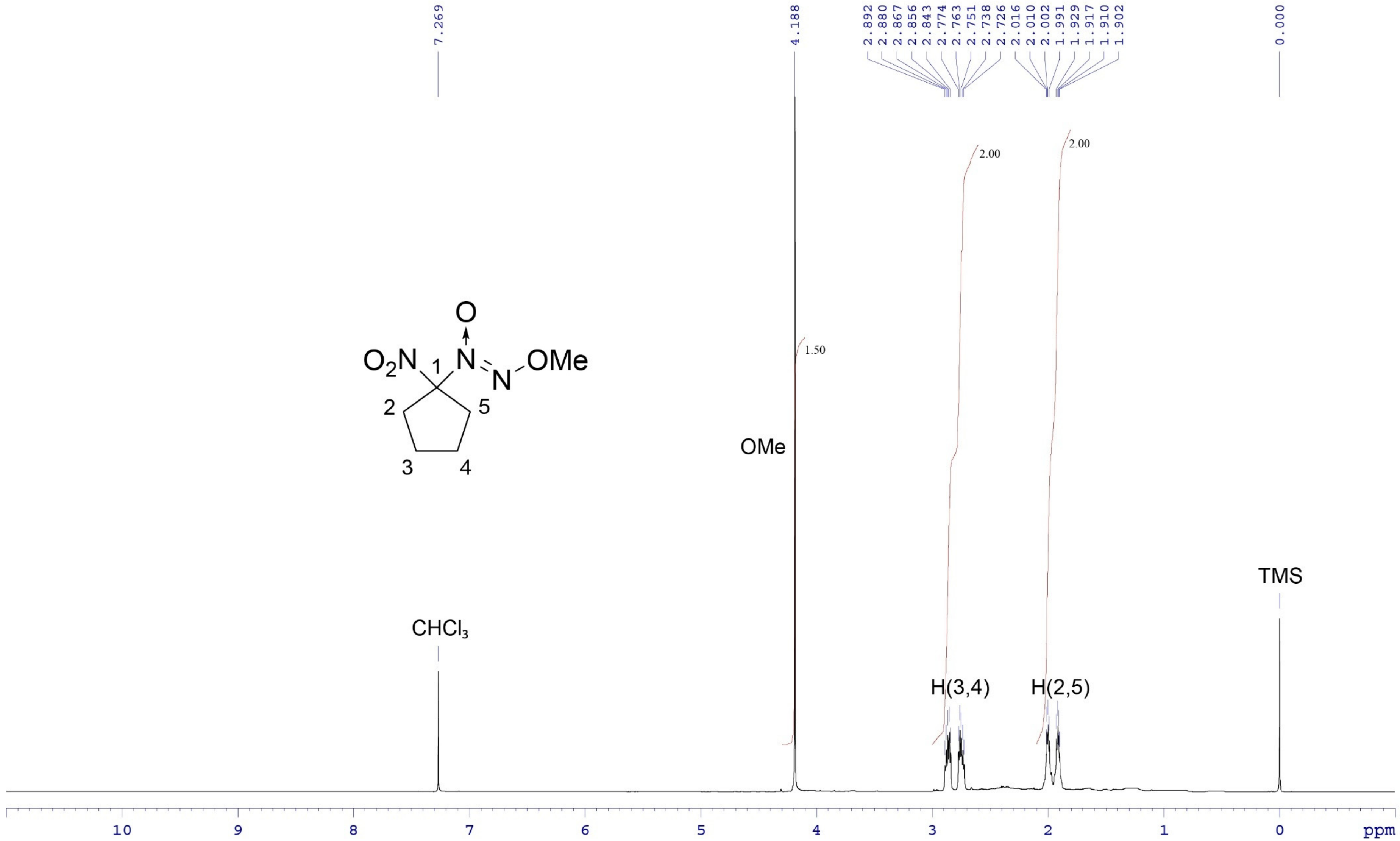

9.13.2 ^{13}C NMR spectrum of compound 2m [150.90 MHz, $CDCl_3$]

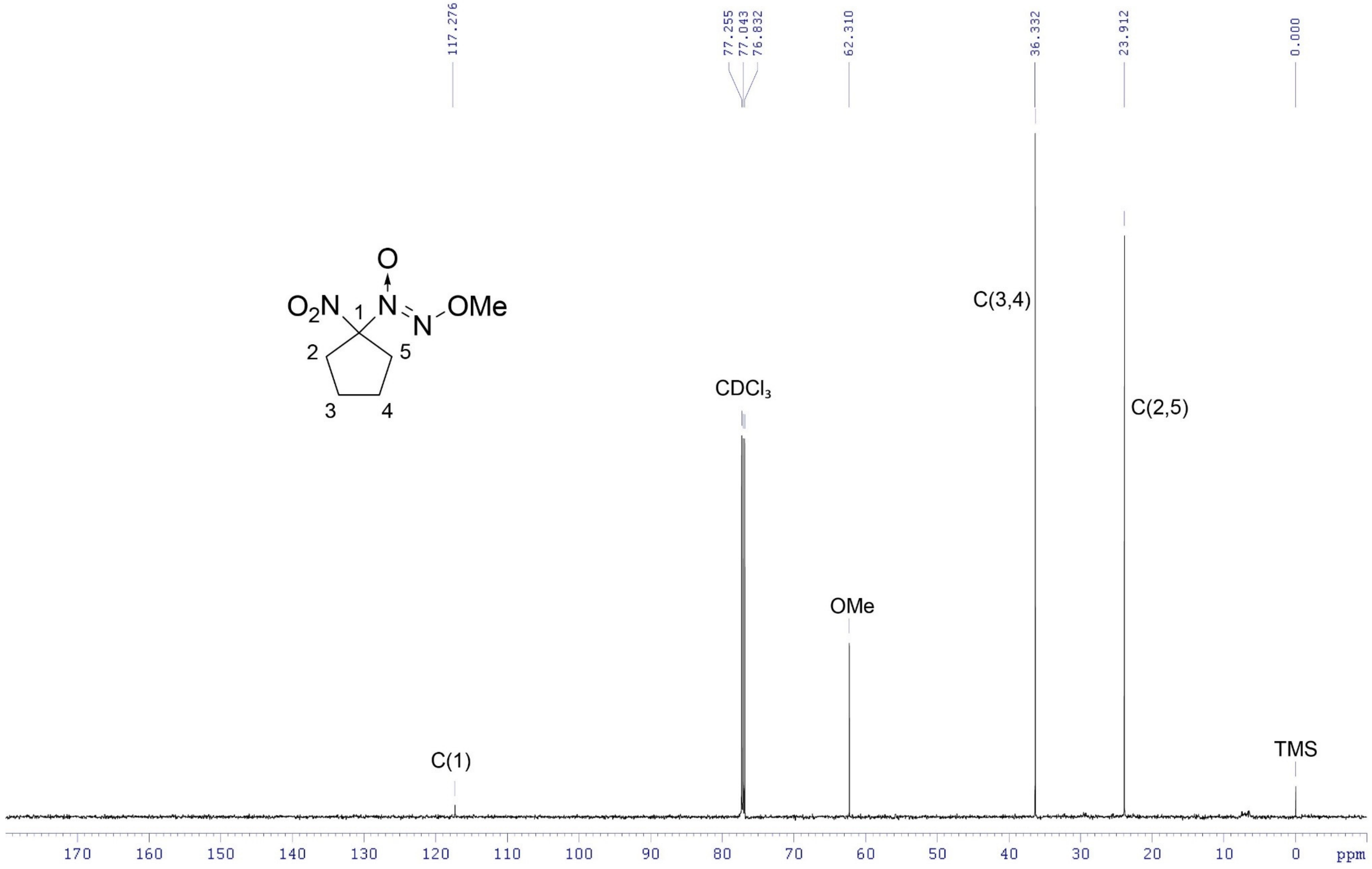

9.13.3 {^1H–^{13}C} HSQC spectrum of compound 2m [600.13 MHz, $CDCl_3$]

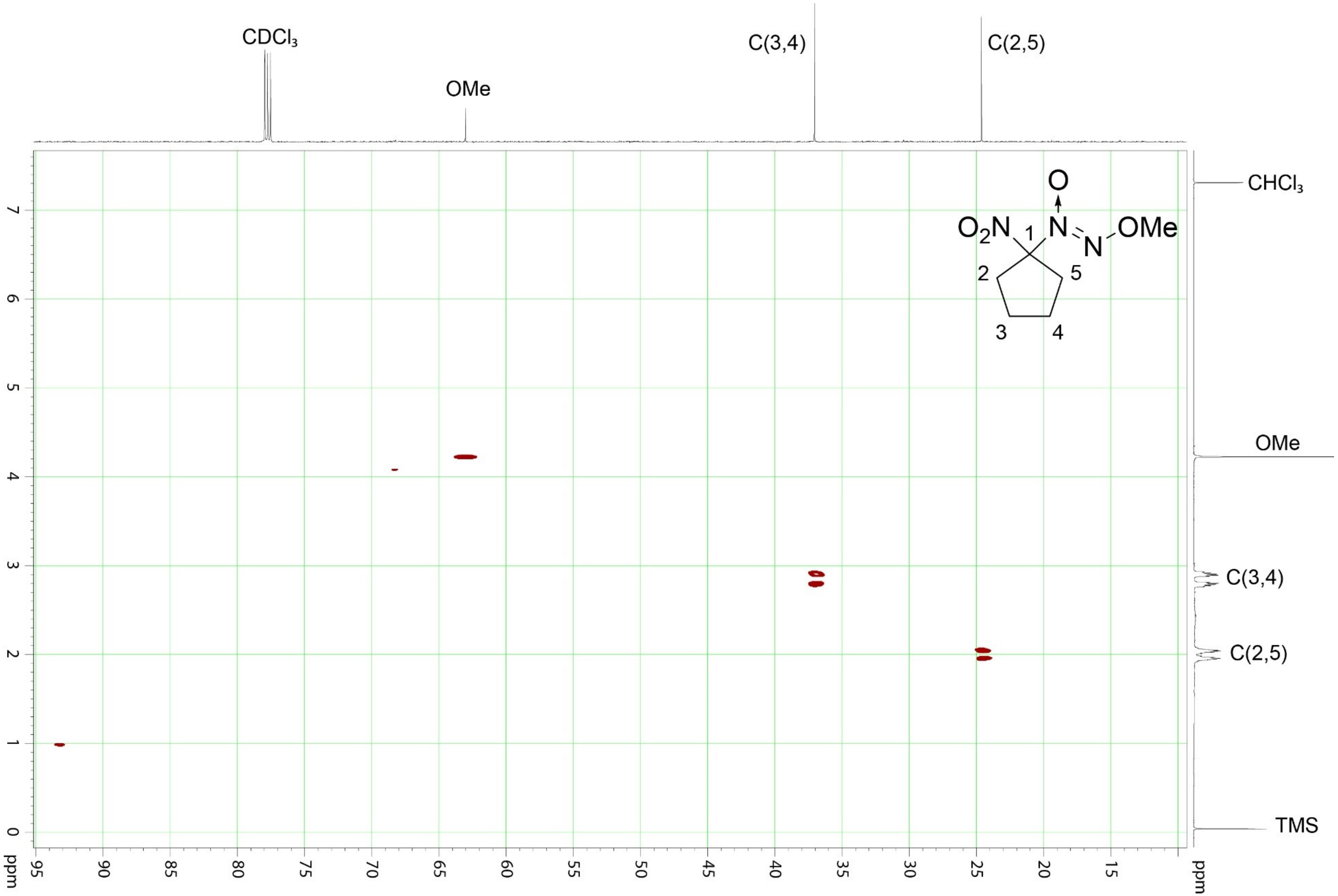

9.13.4 {^{1}H–^{13}C} HMBC spectrum of compound 2m [600.13 MHz, $CDCl_3$]

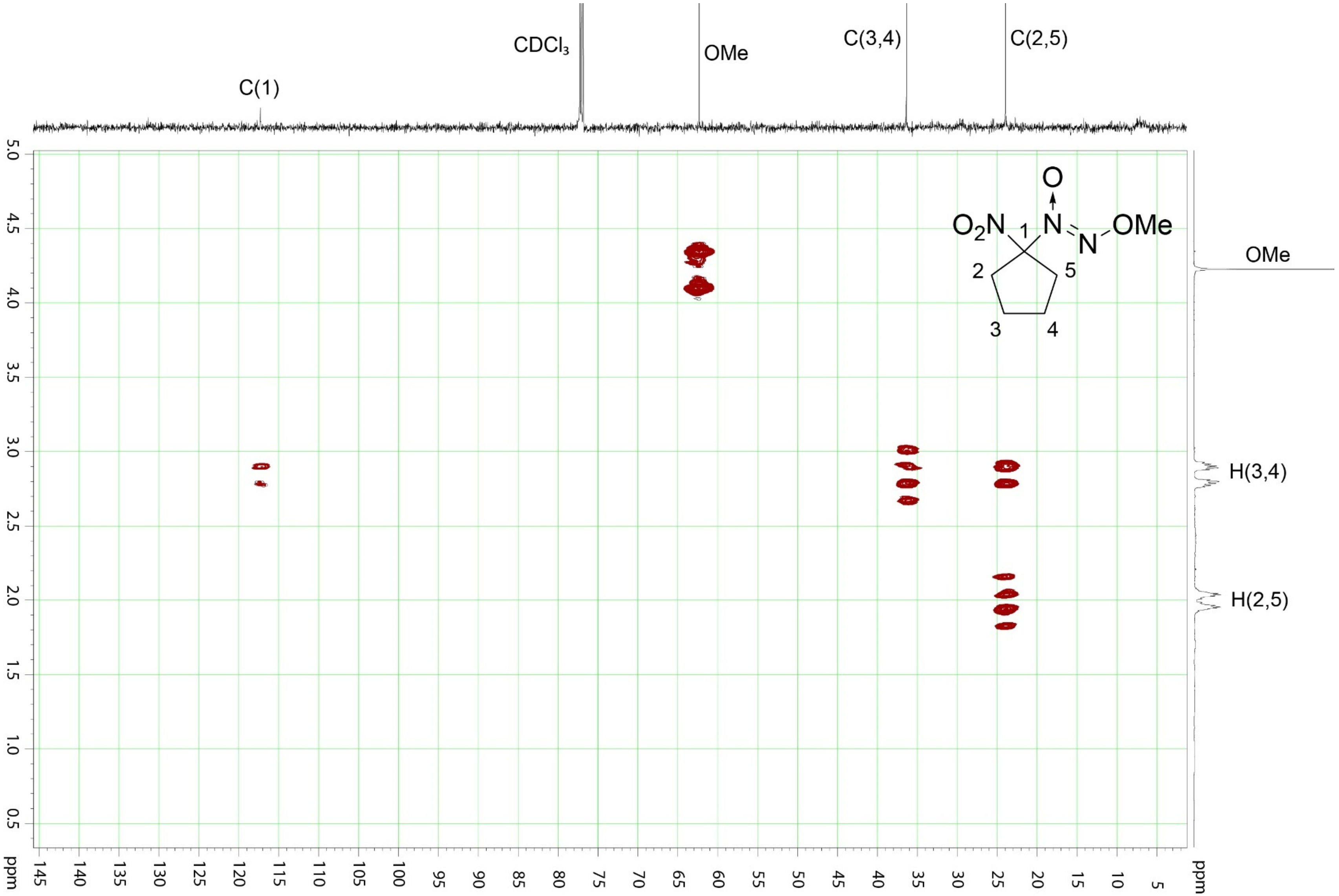

9.13.5 ^{14}N NMR spectrum of compound 2m [43.37 MHz, $CDCl_3$]

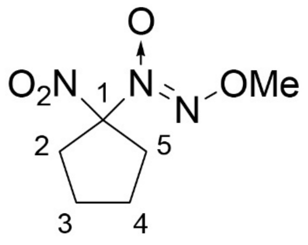

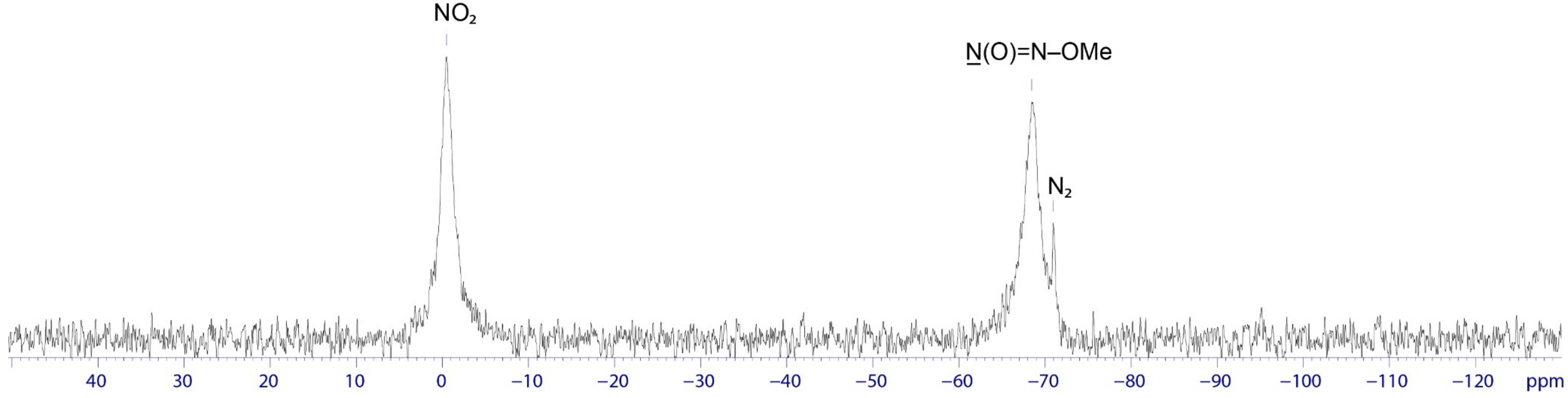

9.14.1 ^{1}H NMR spectrum of compound 2n [600.13 MHz, $CDCl_3$]

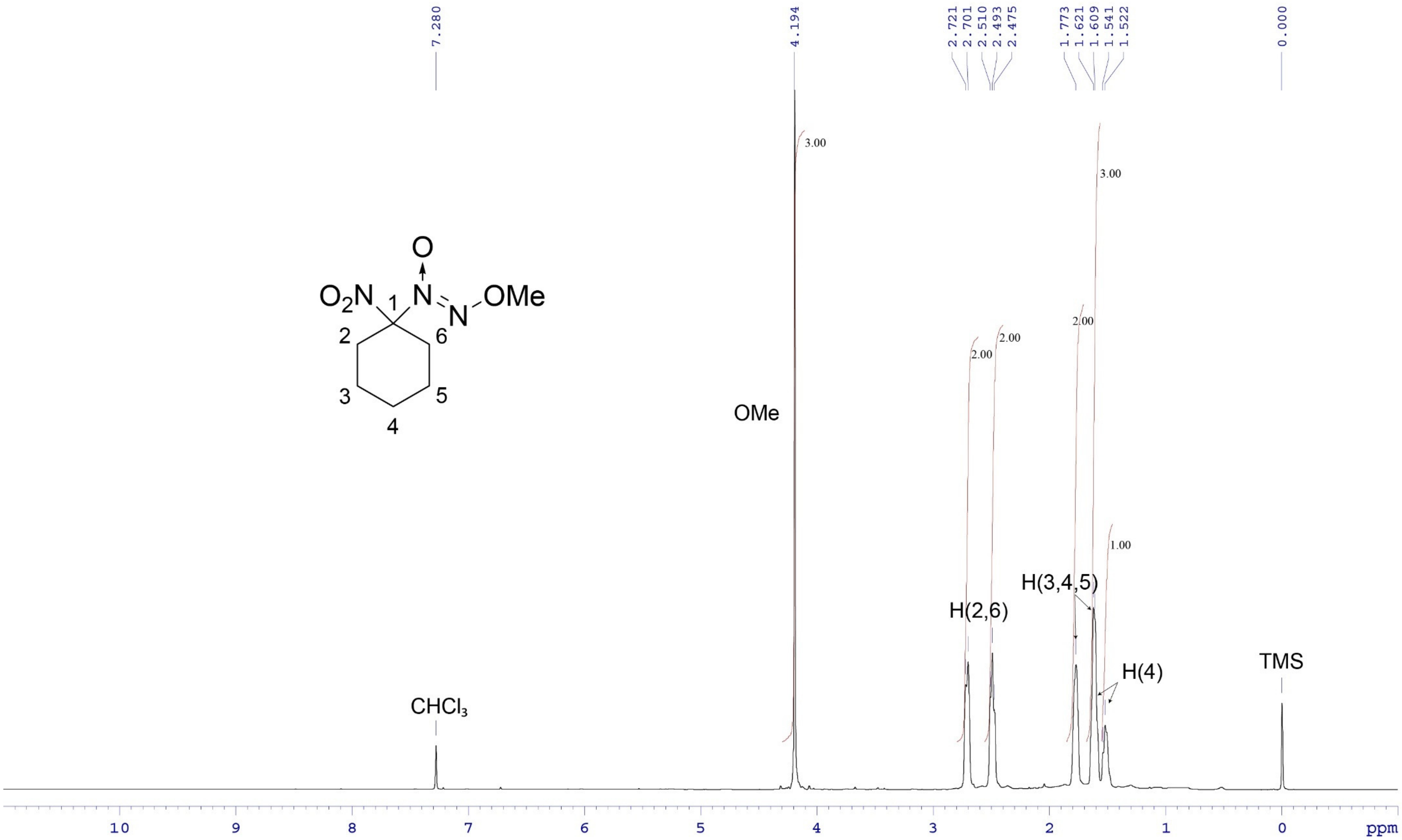

9.14.2 ^{13}C NMR spectrum of compound 2n [150.90 MHz, $CDCl_3$]

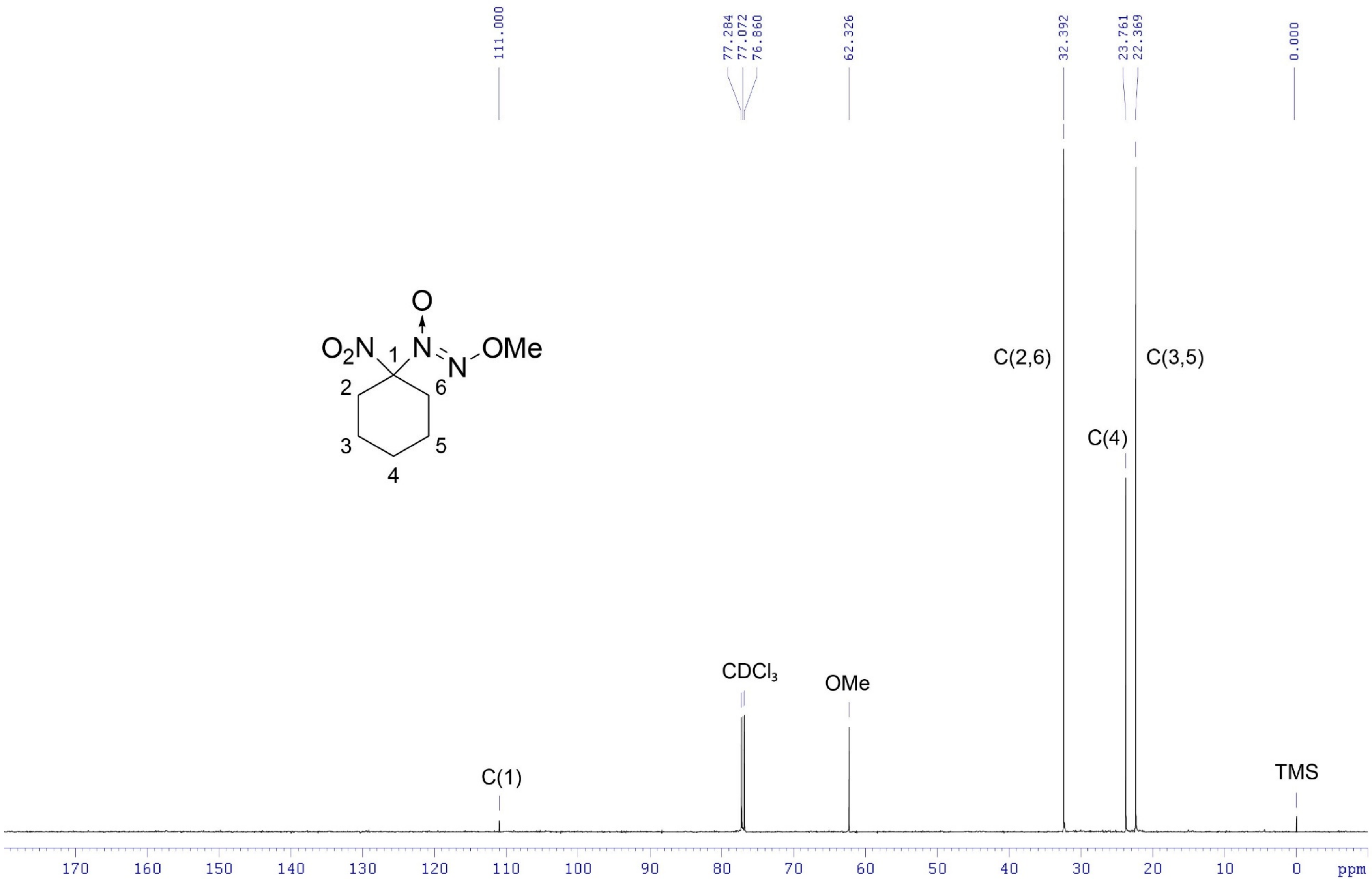

9.14.3 {^{1}H–^{13}C} HSQC spectrum of compound 2n [600.13 MHz, $CDCl_3$]

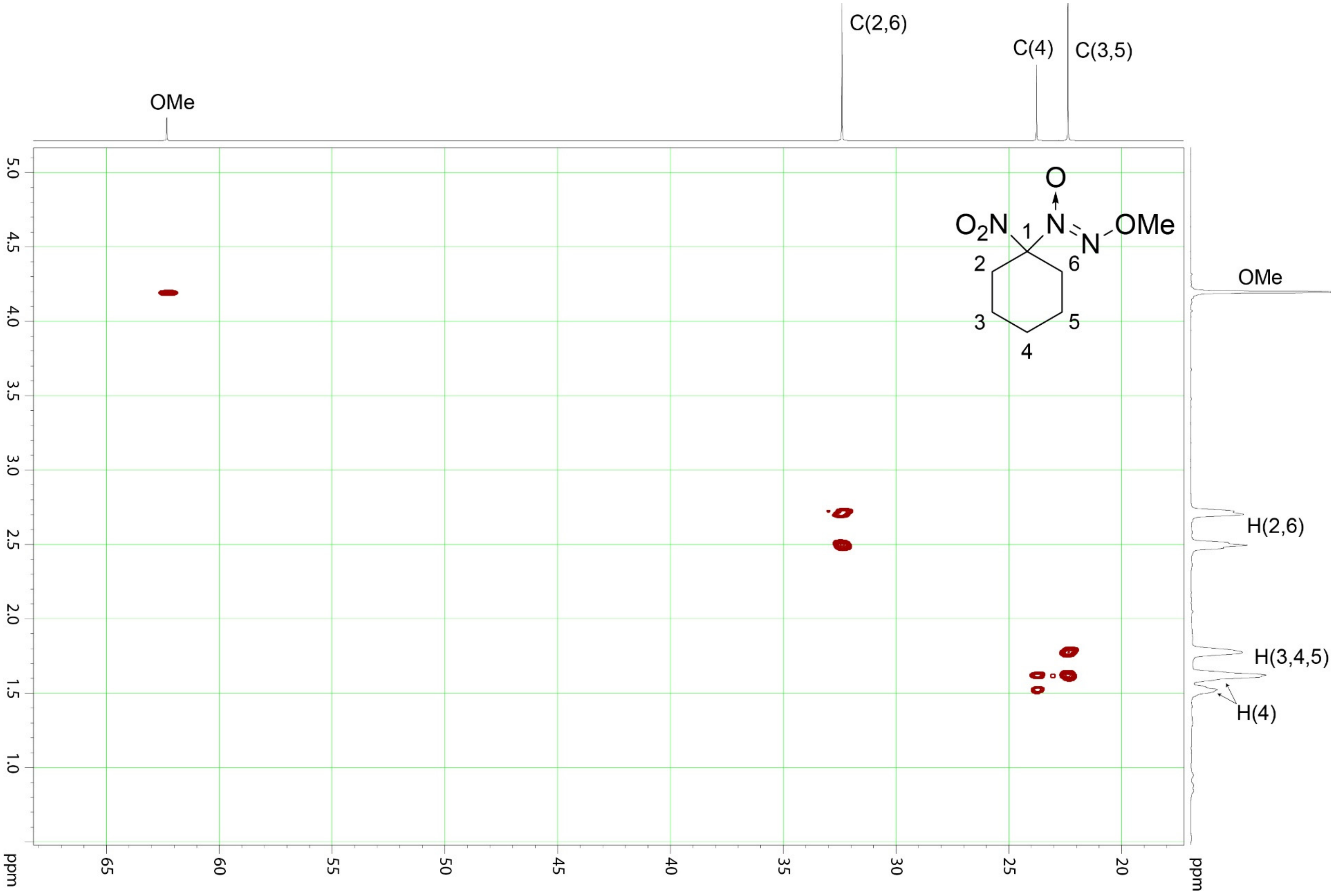

9.14.4 {^{1}H–^{13}C} HMBC spectrum of compound 2n [600.13 MHz, $CDCl_3$]

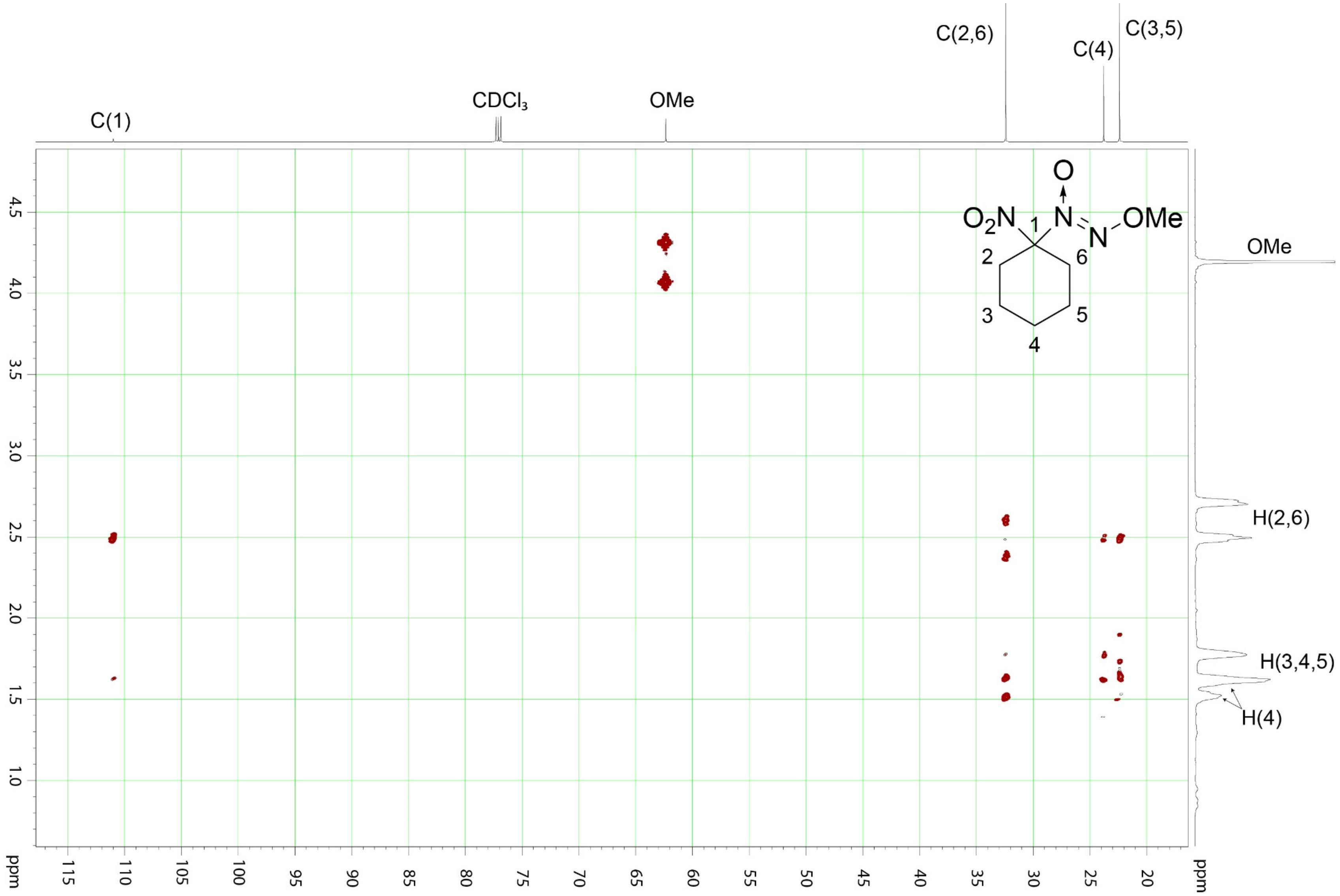

9.14.5 ^{14}N NMR spectrum of compound 2n [43.37 MHz, $CDCl_3$]

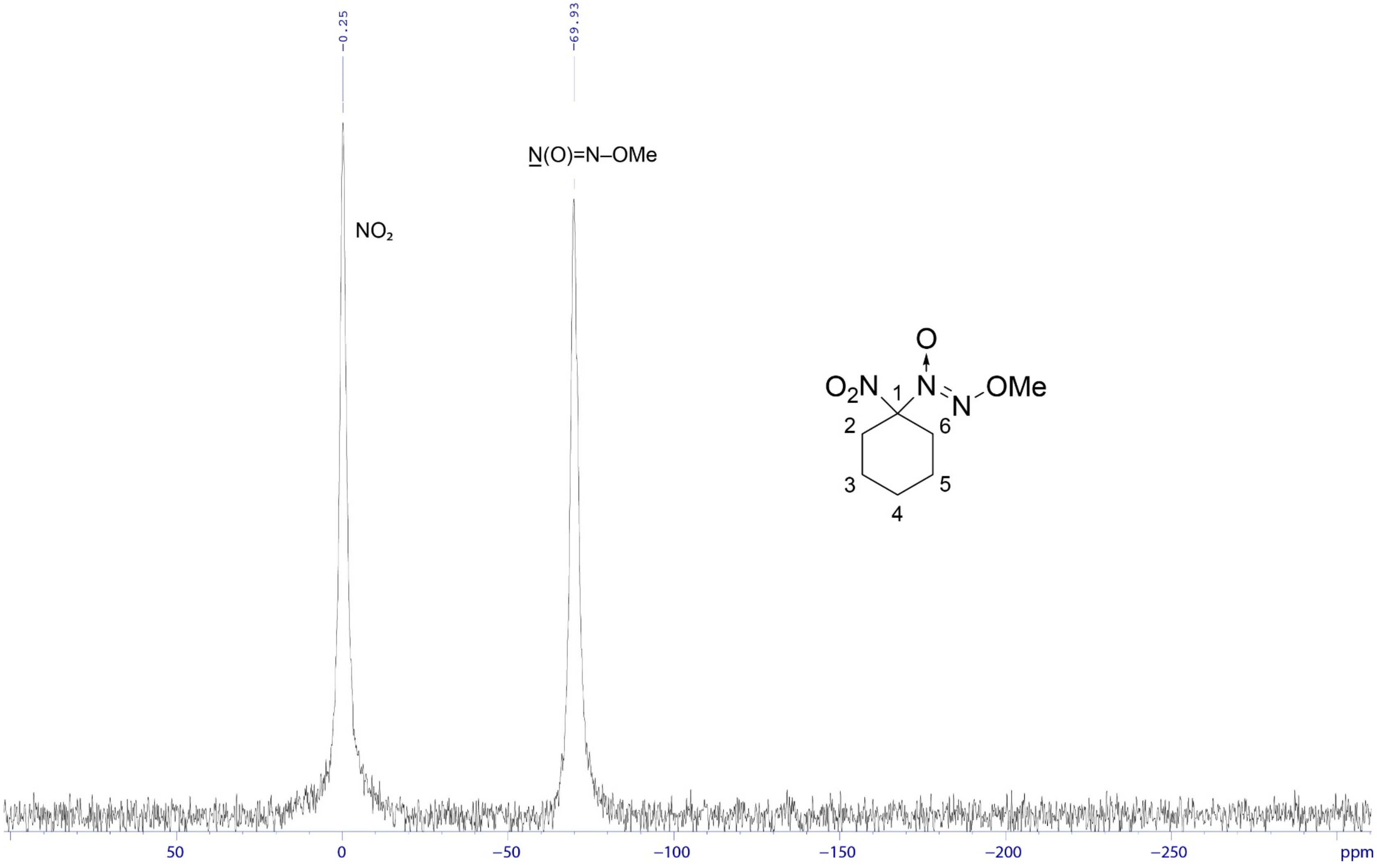

9.15.1 ^{1}H NMR spectrum of compound 2o [500.13 MHz, $CDCl_3$]

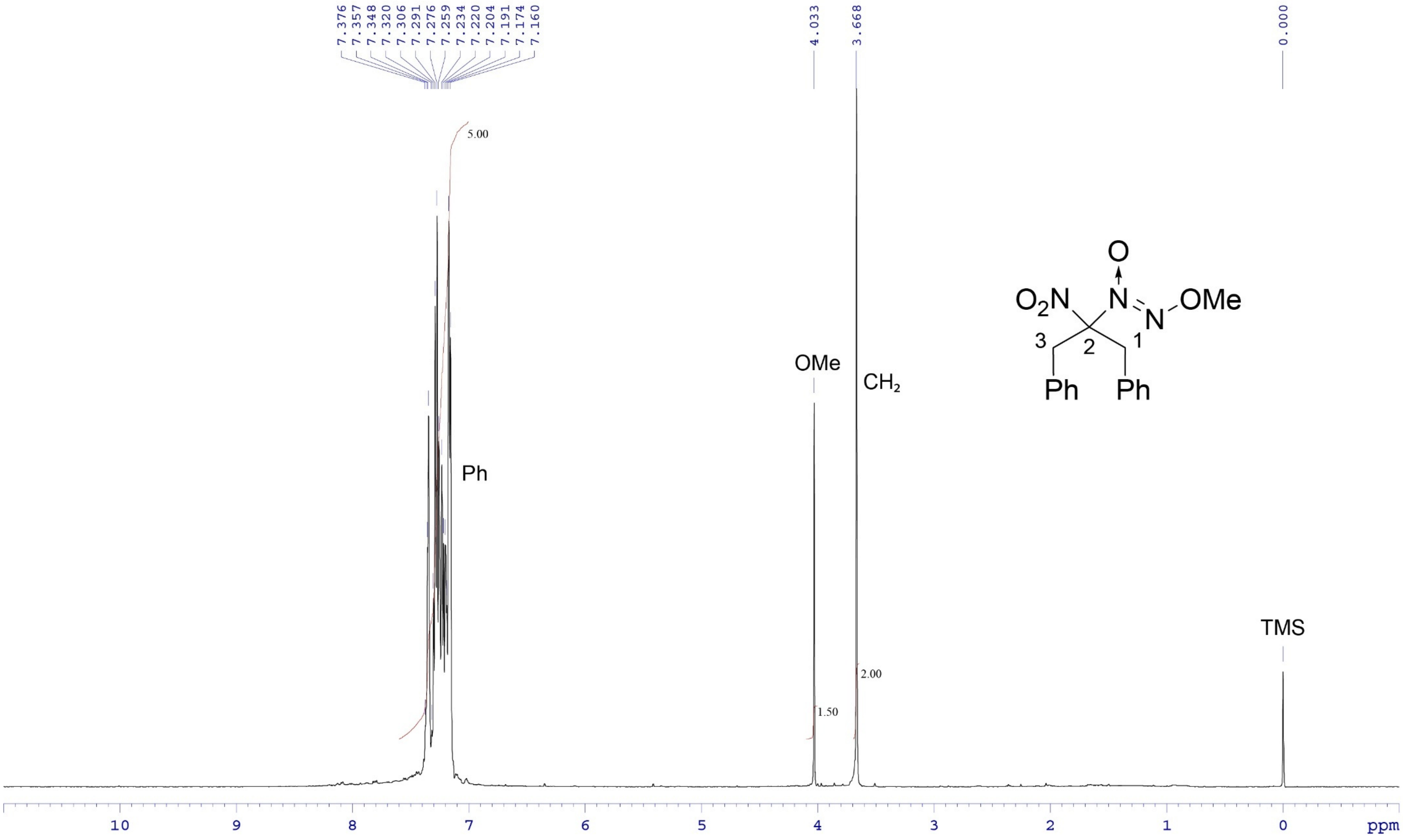

9.15.2 ^{13}C NMR spectrum of compound 2o [125.76 MHz, $CDCl_3$]

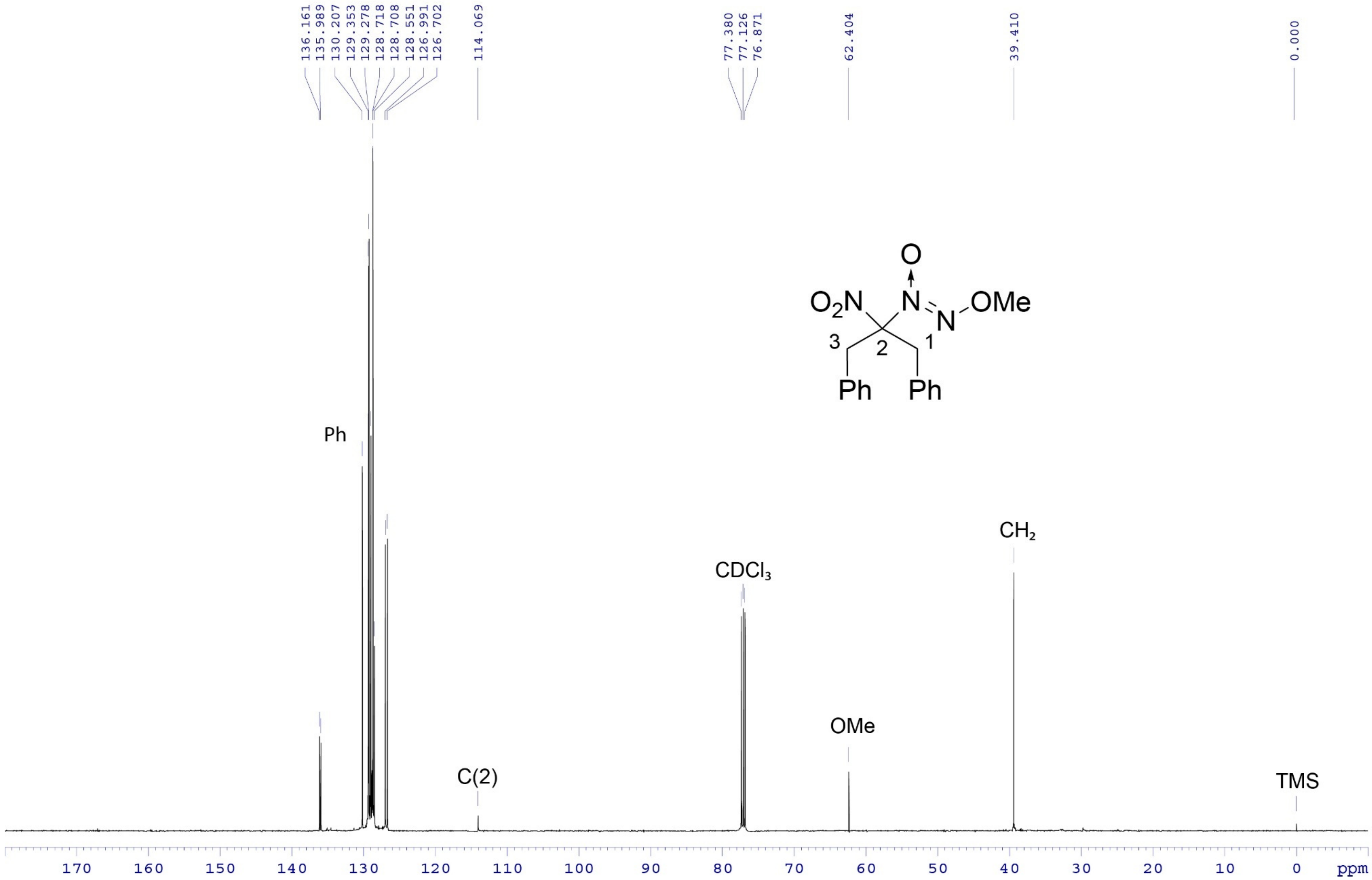

9.15.3 {^1H–^{13}C} HSQC spectrum of compound 2o [500.13 MHz, $CDCl_3$]

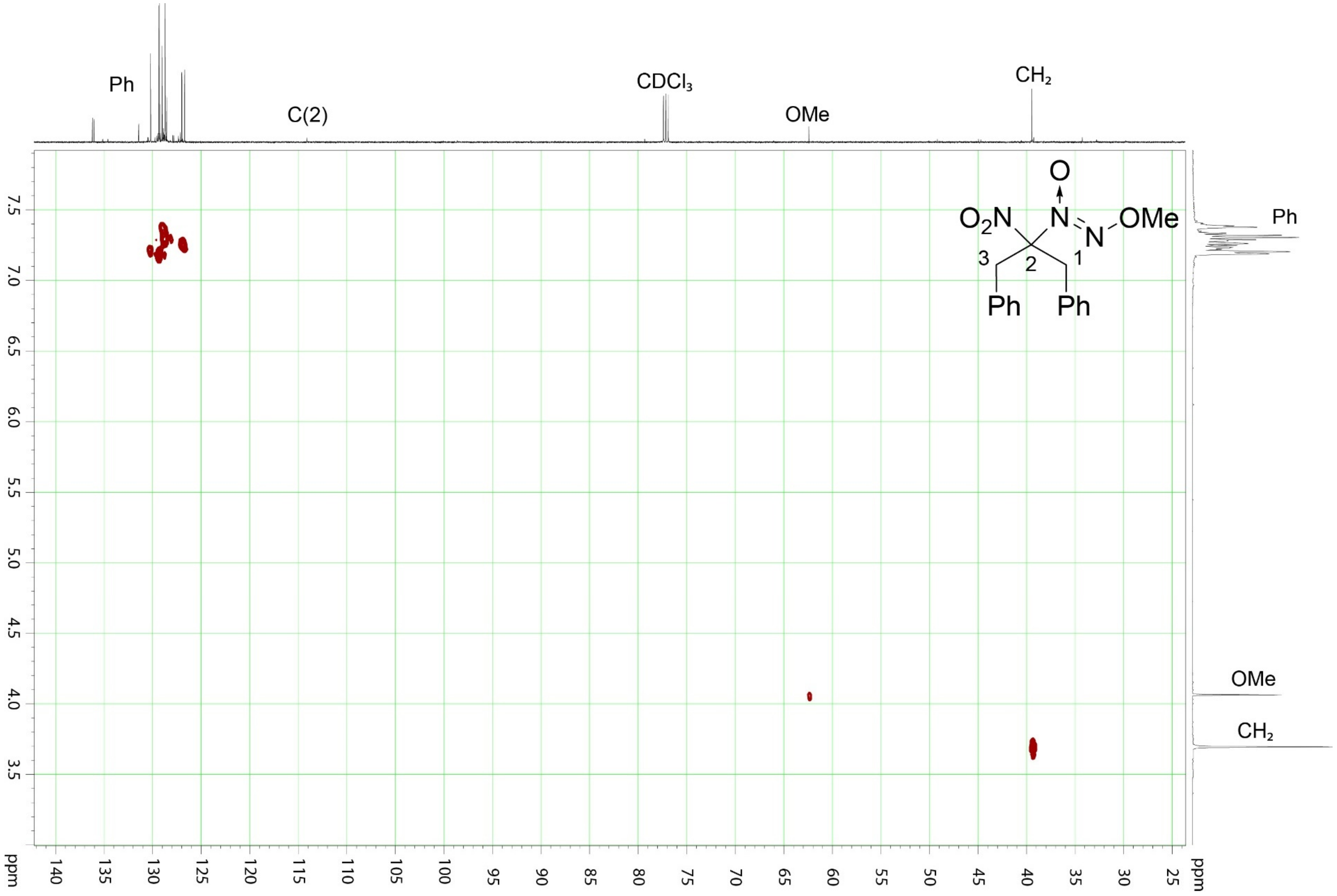

9.15.4 {^1H–^{13}C} HMBC spectrum of compound 2o [500.13 MHz, $CDCl_3$]

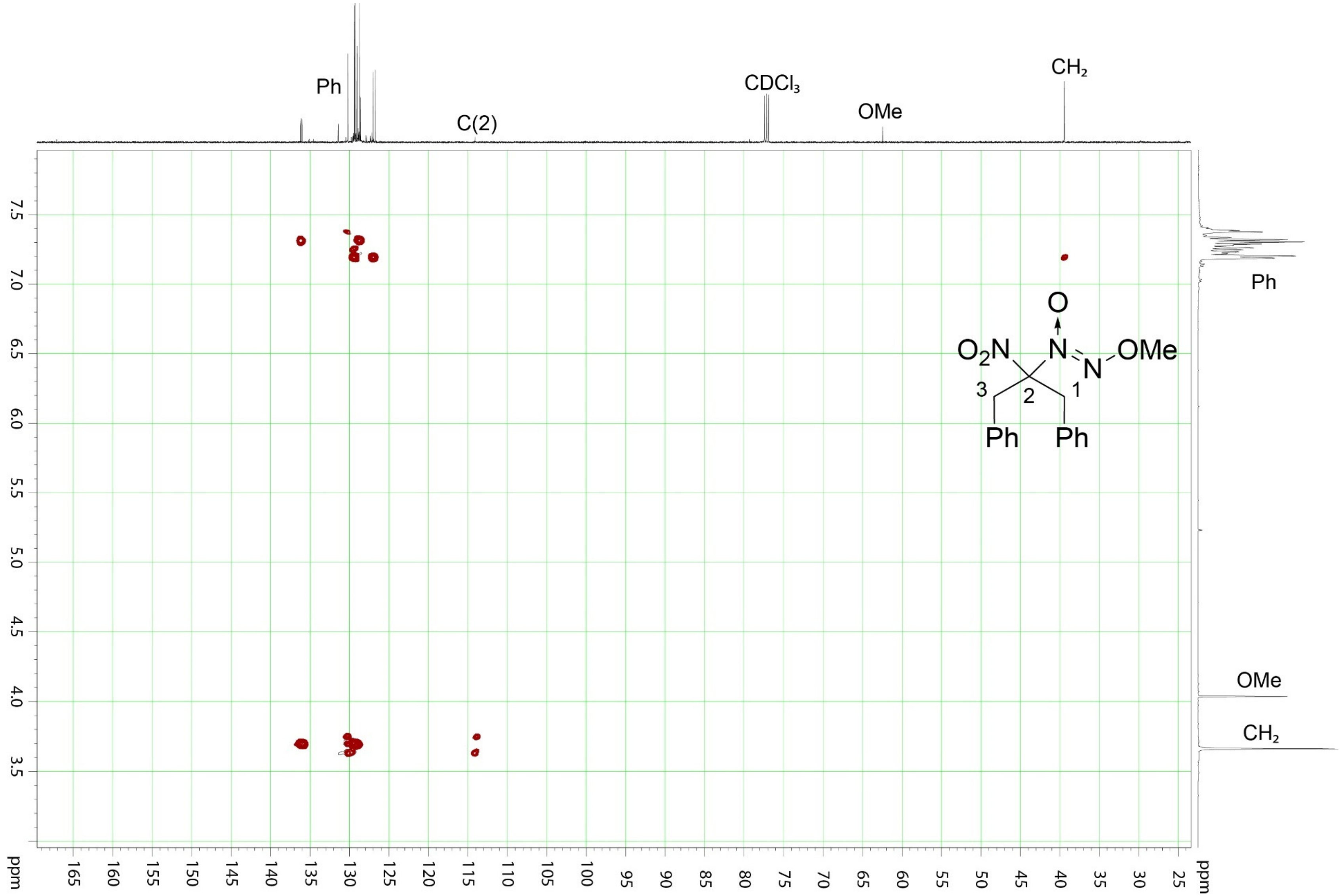

9.15.5 ^{14}N NMR spectrum of compound 2o [36.14 MHz, $CDCl_3$]

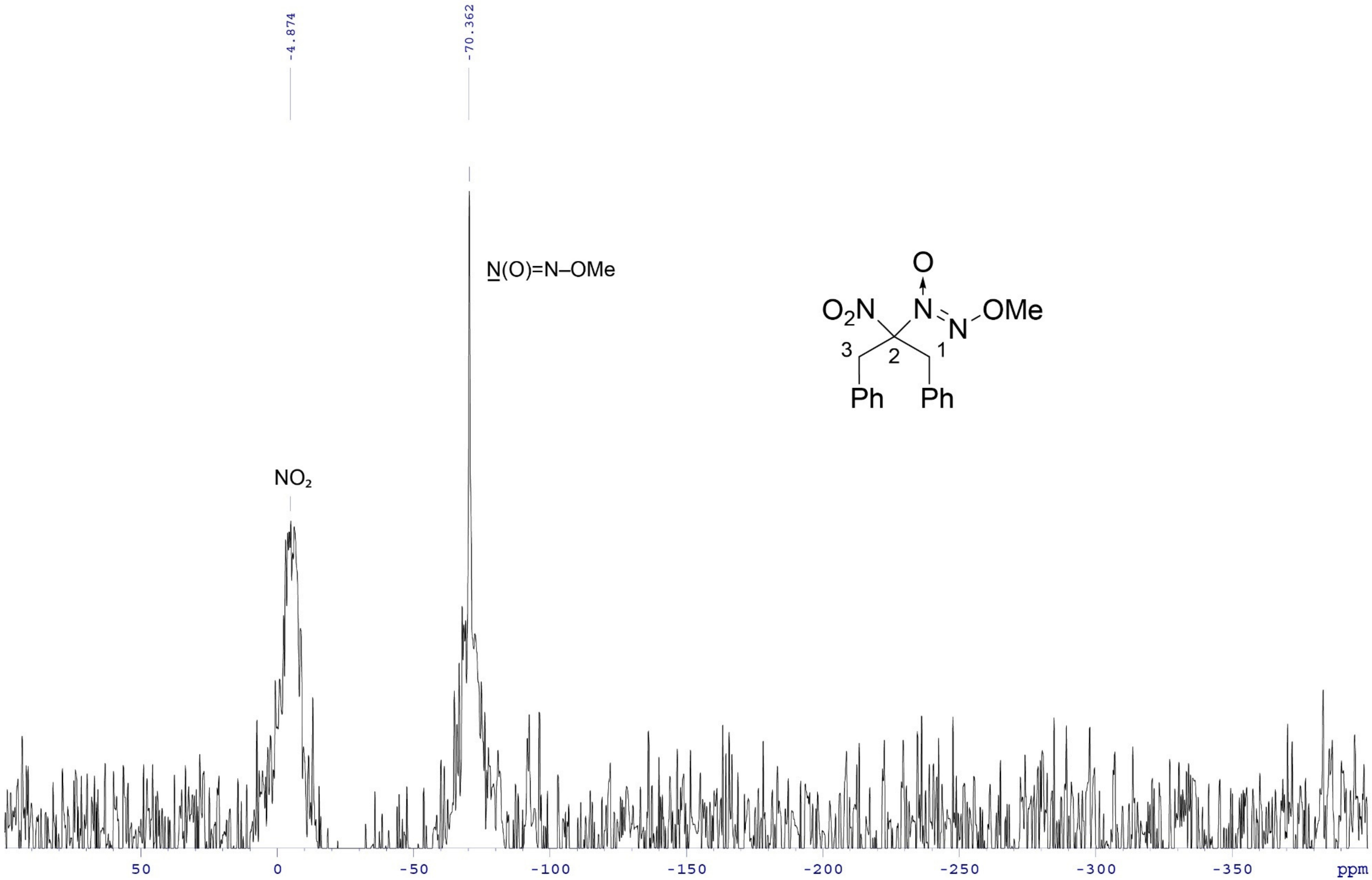

9.16.1 ^{1}H NMR spectrum of compound 2p [500.13 MHz, [D$_6$]acetone]

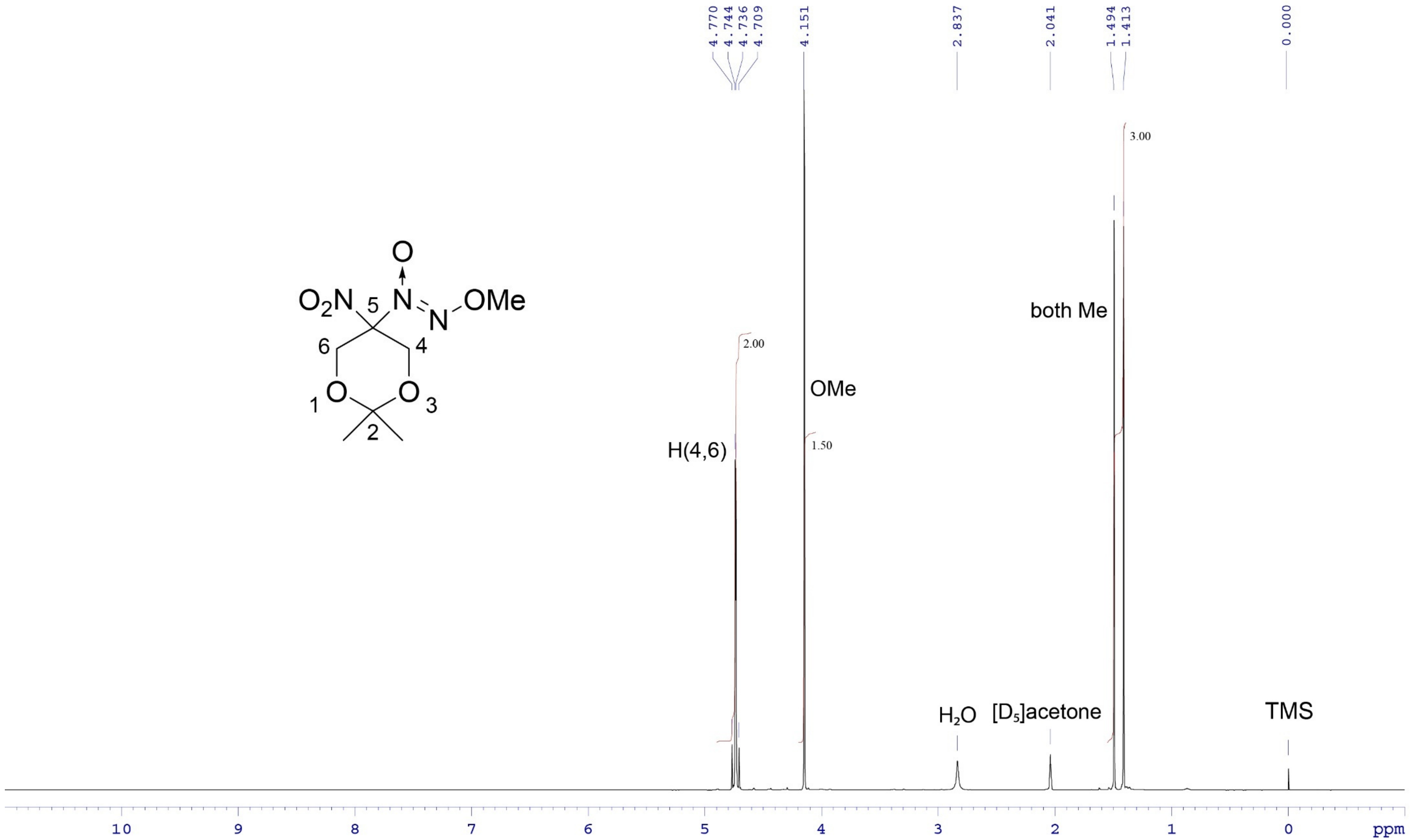

9.16.2 ^{13}C NMR spectrum of compound 2p [125.76 MHz, [D$_6$]acetone]

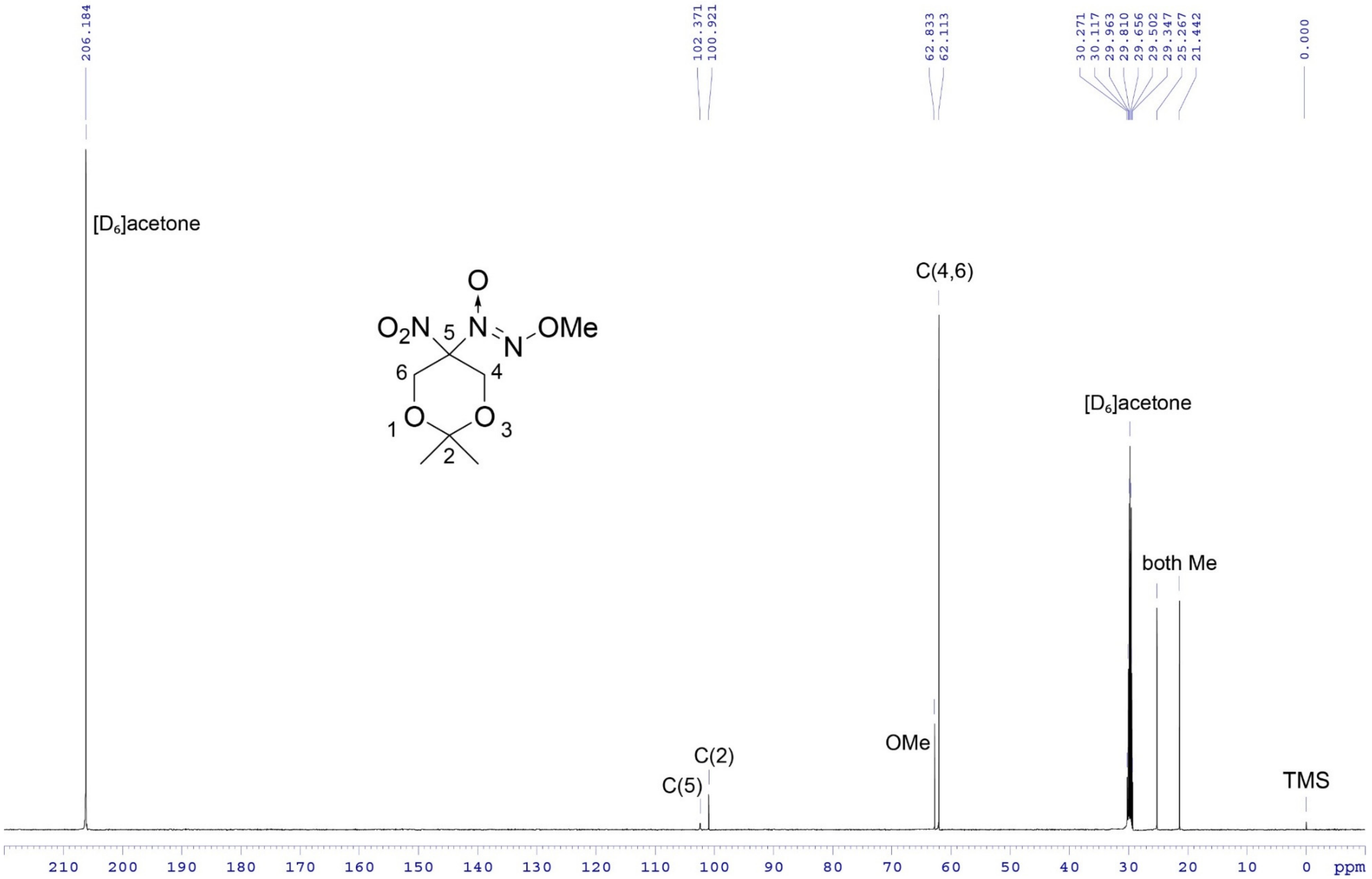

9.16.3 {^1H–^{13}C} HSQC spectrum of compound 2p [500.13 MHz, [D$_6$]acetone]

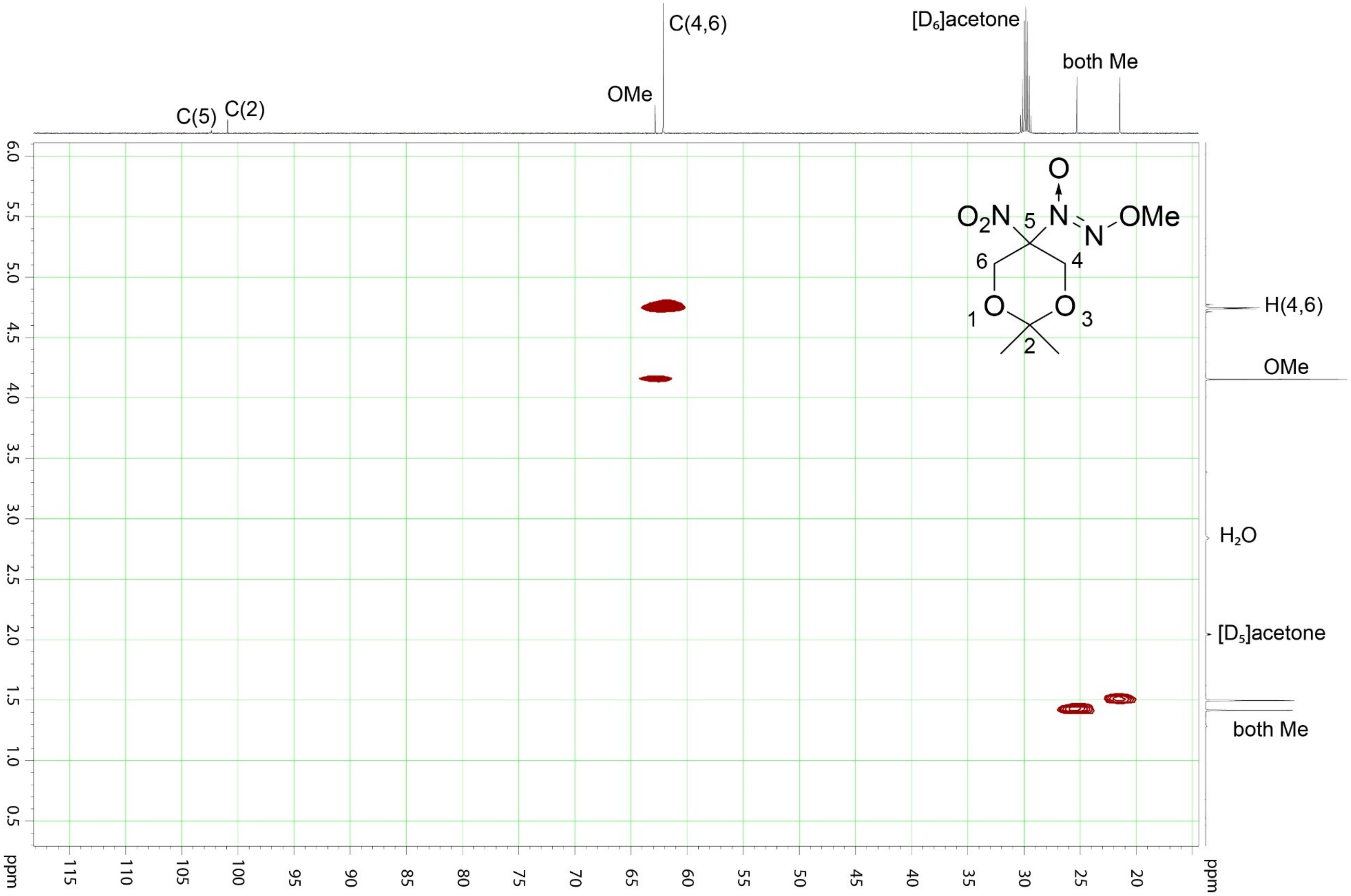

9.16.4 {^{1}H–^{13}C} HMBC spectrum of compound 2p [500.13 MHz, [D$_6$]acetone]

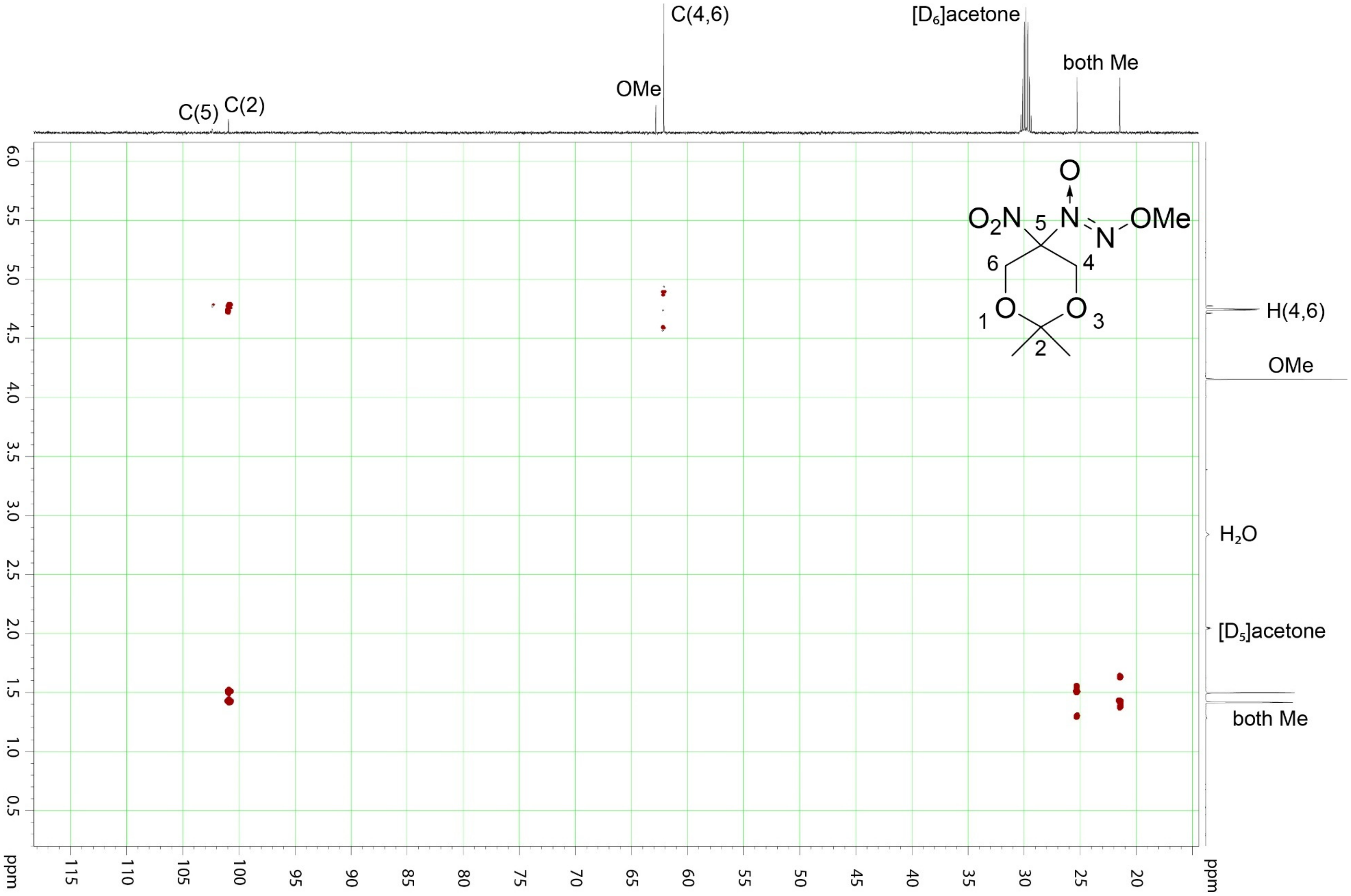

9.16.5 ^{14}N NMR spectrum of compound 2p [36.14 MHz, [D₆]acetone]

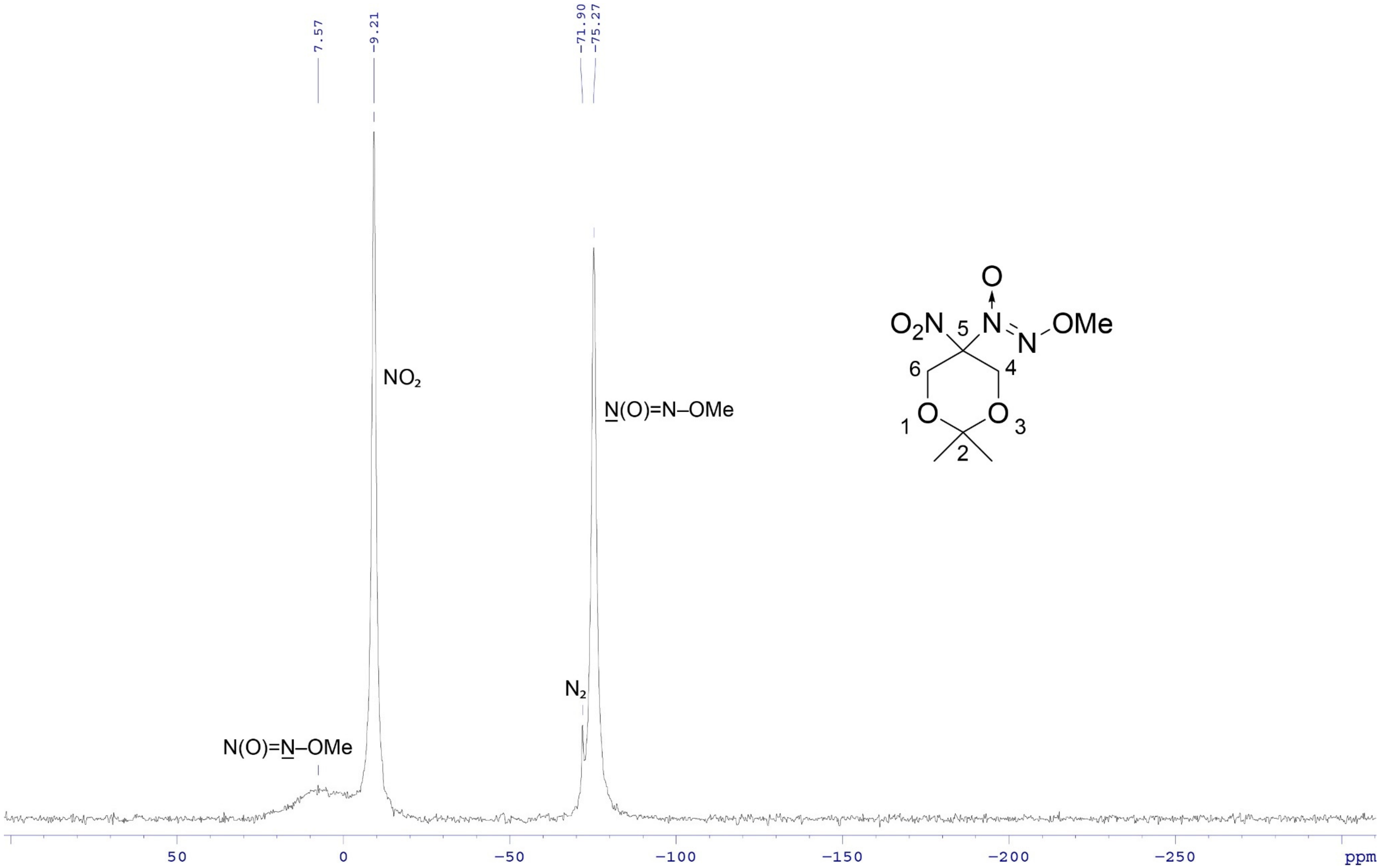

9.17.1 ^{1}H NMR spectrum of compound 2q [500.13 MHz, $CDCl_3$]

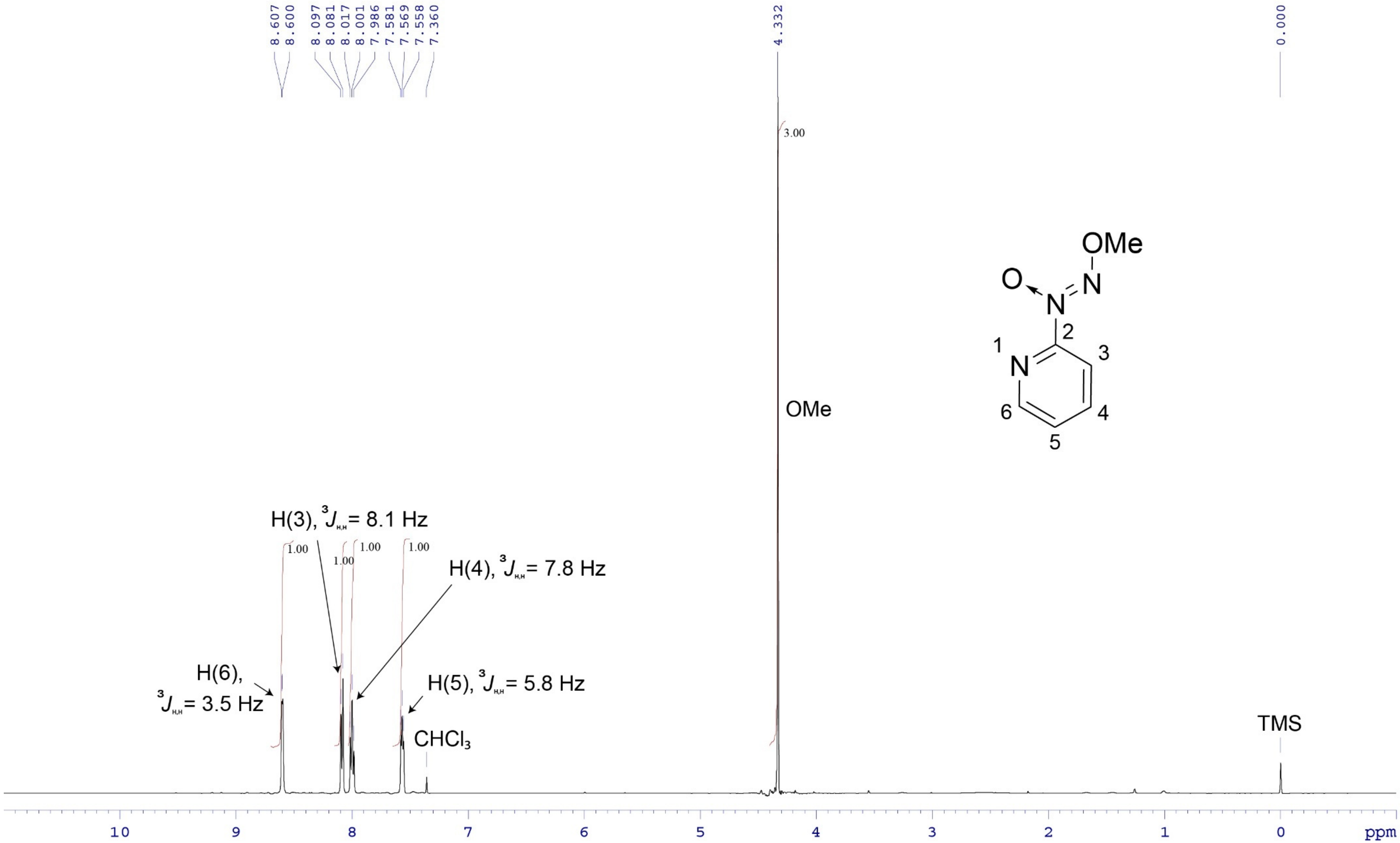

9.17.2 ^{13}C NMR spectrum of compound 2q [125.76 MHz, CDCl$_3$]

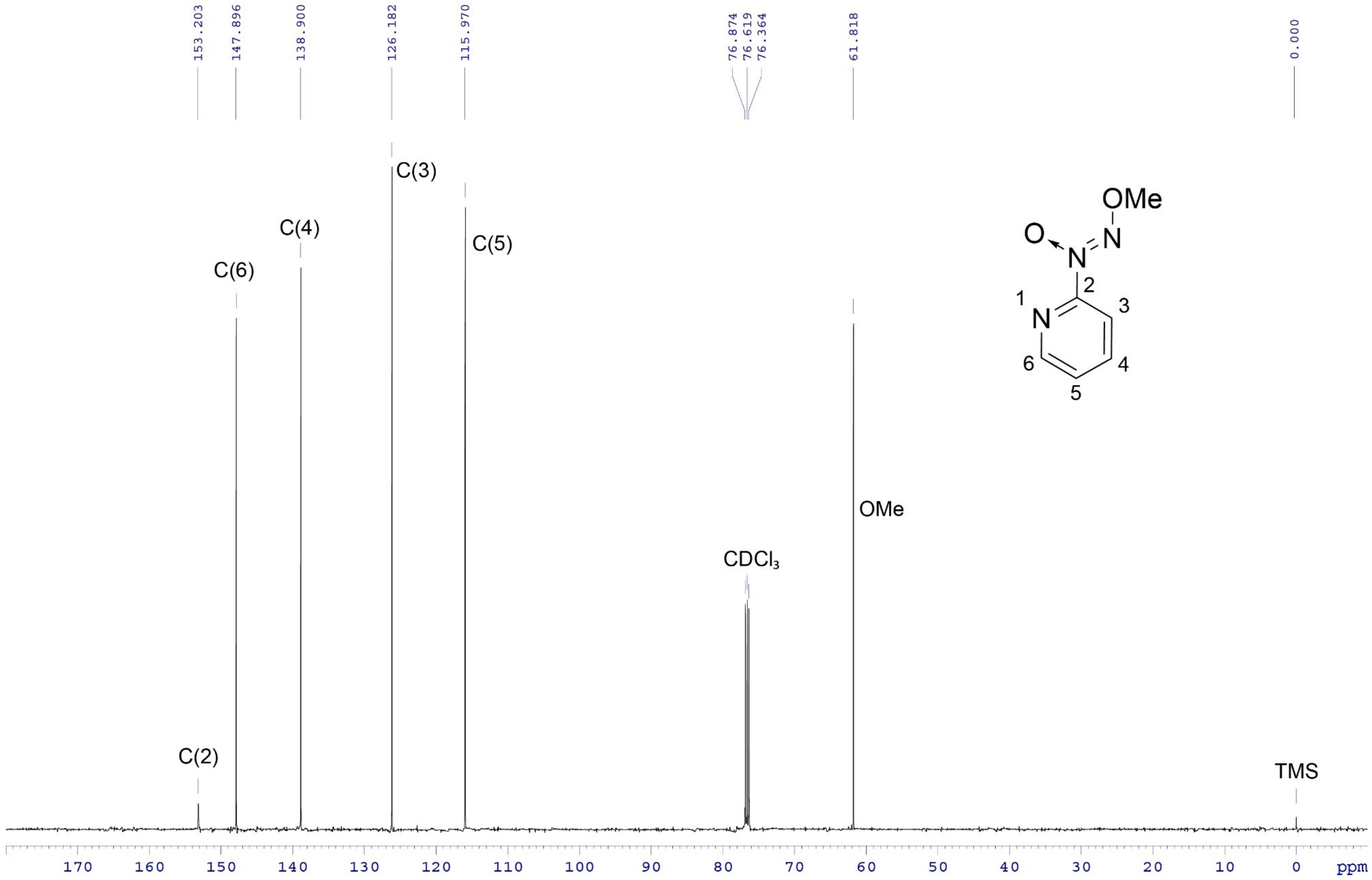

9.17.3 {^{1}H–^{13}C} HSQC spectrum of compound 2q [500.13 MHz, $CDCl_3$]

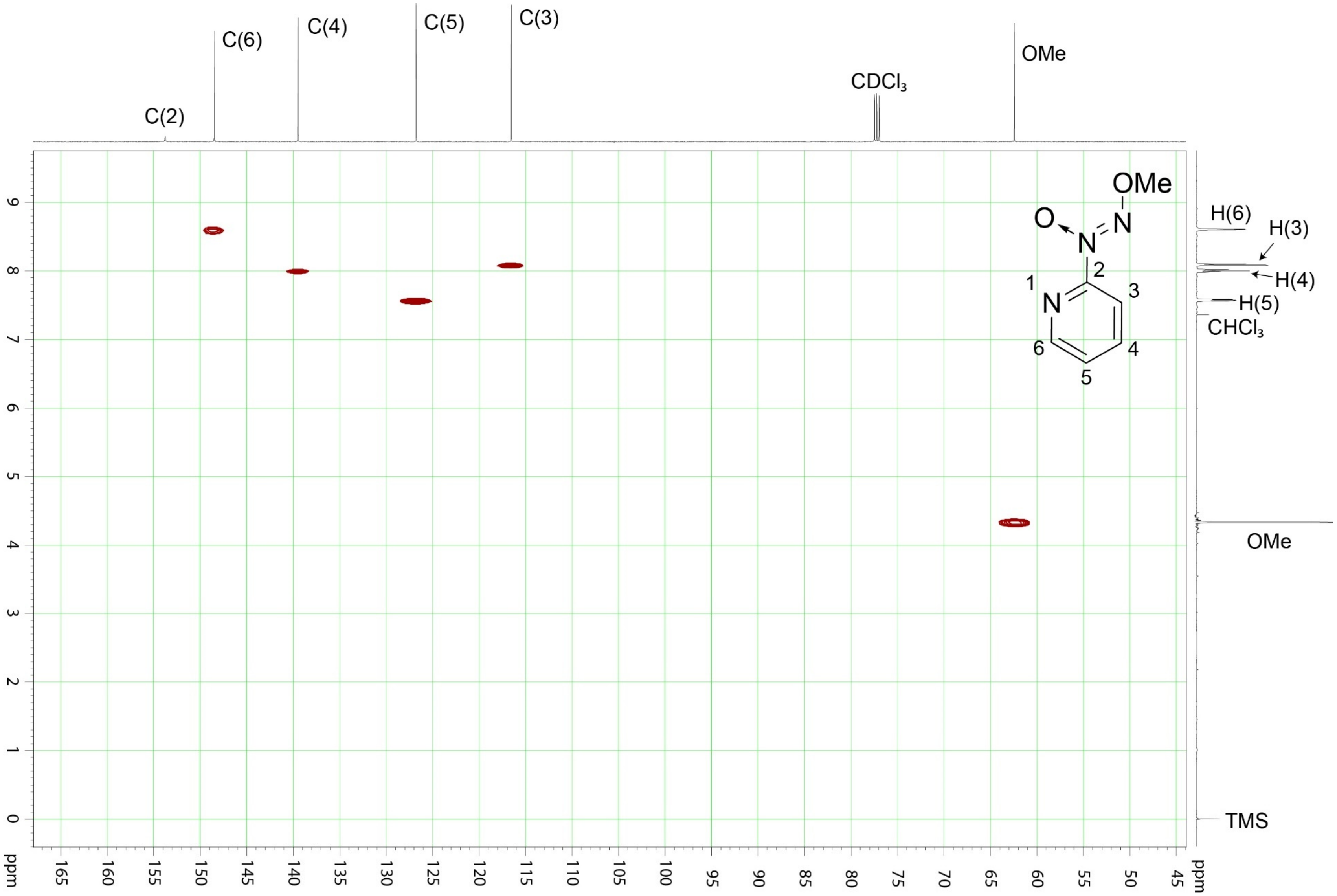

9.17.4 {^{1}H–^{13}C} HMBC spectrum of compound 2q [500.13 MHz, $CDCl_3$]

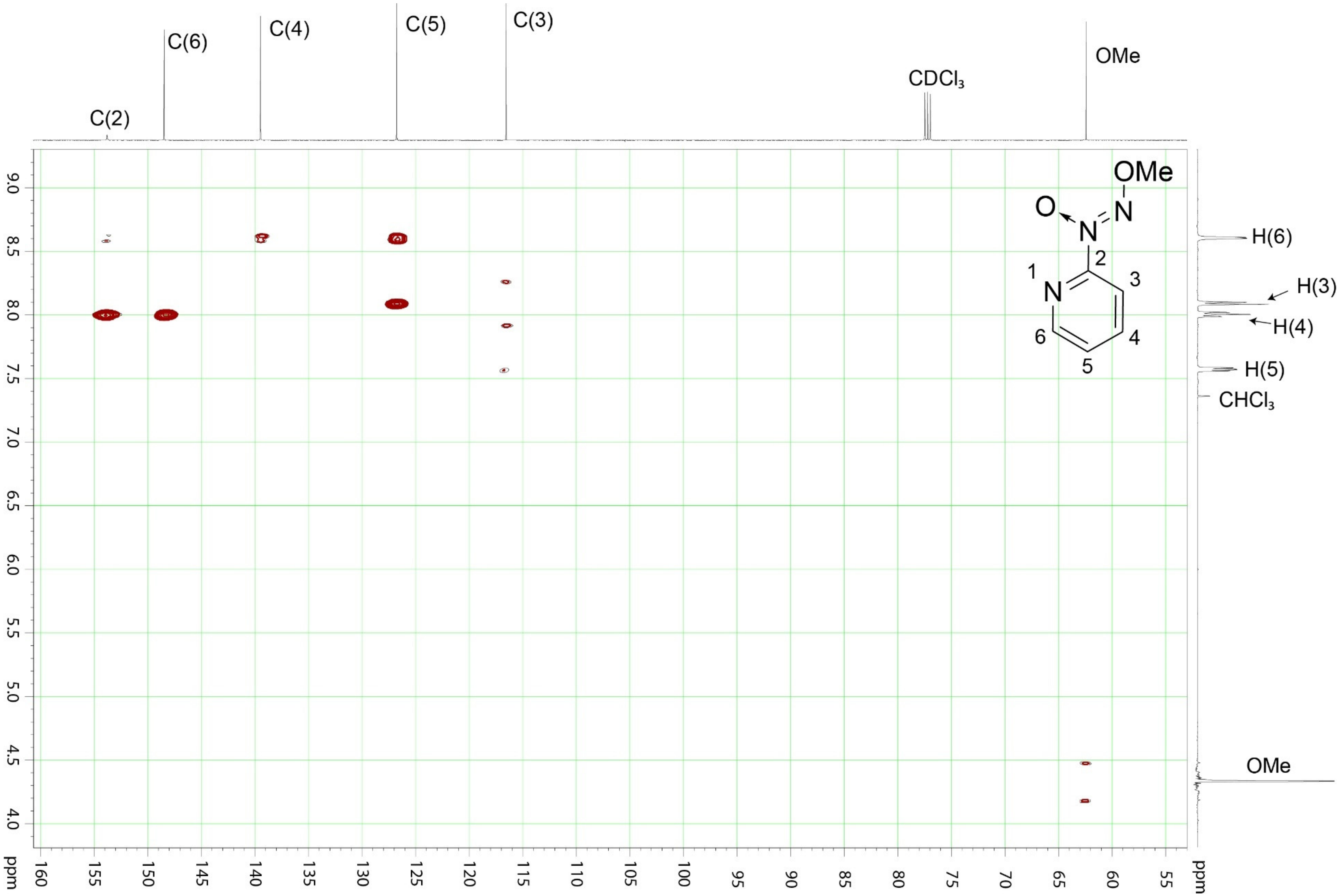

9.17.5 ^{14}N NMR spectrum of compound 2q [36.14 MHz, $CDCl_3$]

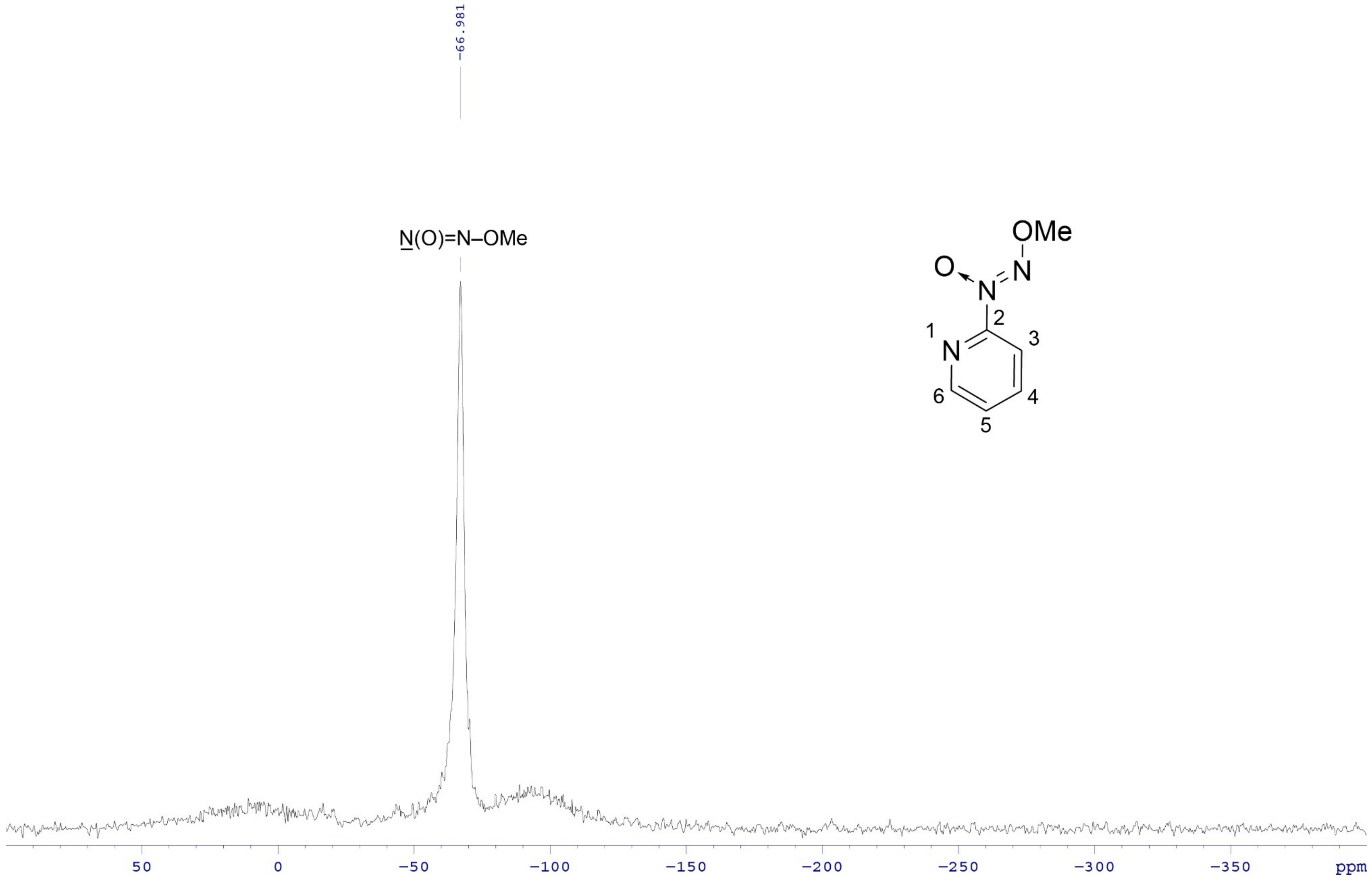

9.18.1 ^{1}H NMR spectrum of compound 2r [500.13 MHz, [D$_6$]acetone]

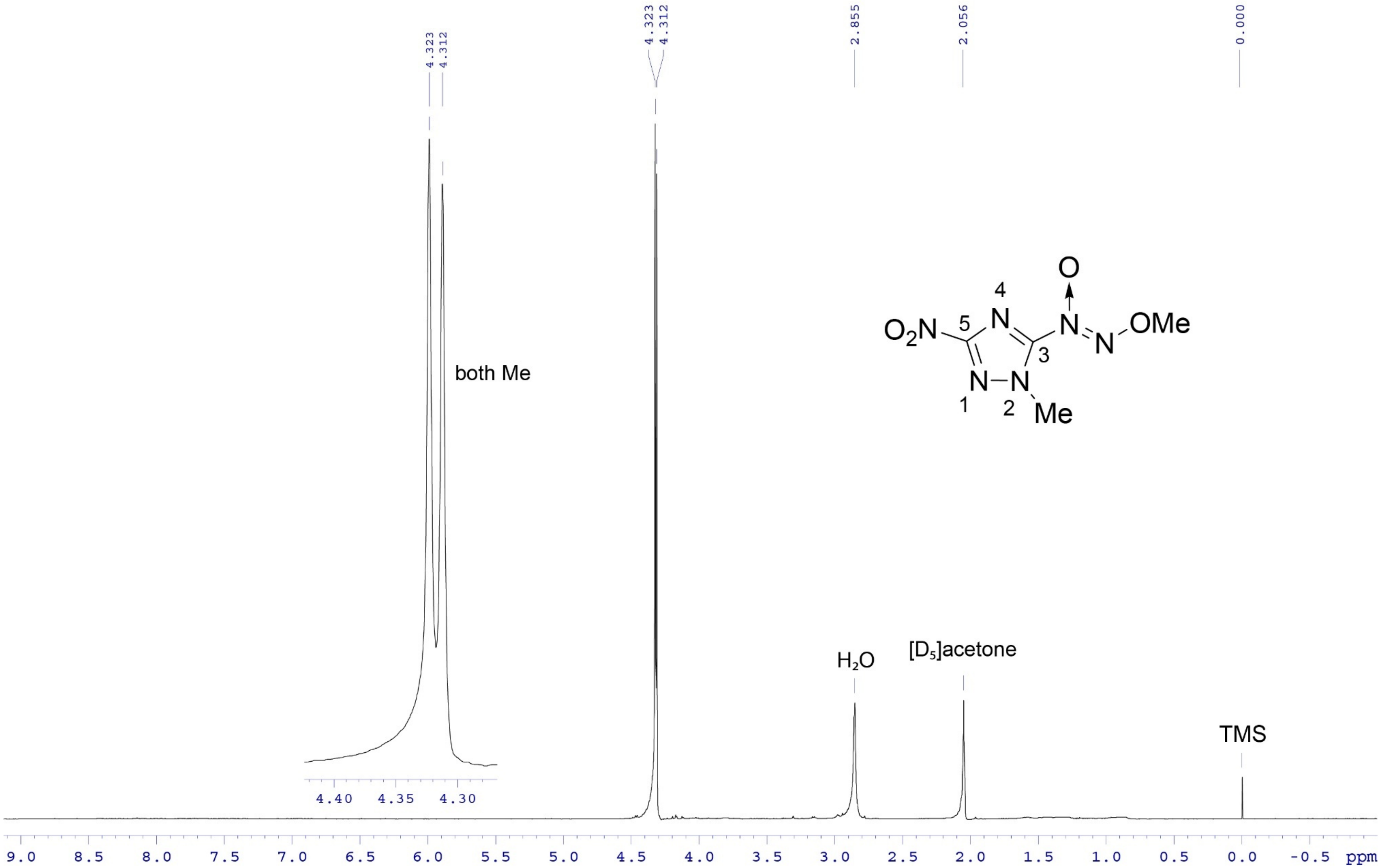

9.18.2 ^{13}C NMR spectrum of compound 2r [125.76 MHz, [D6]acetone]

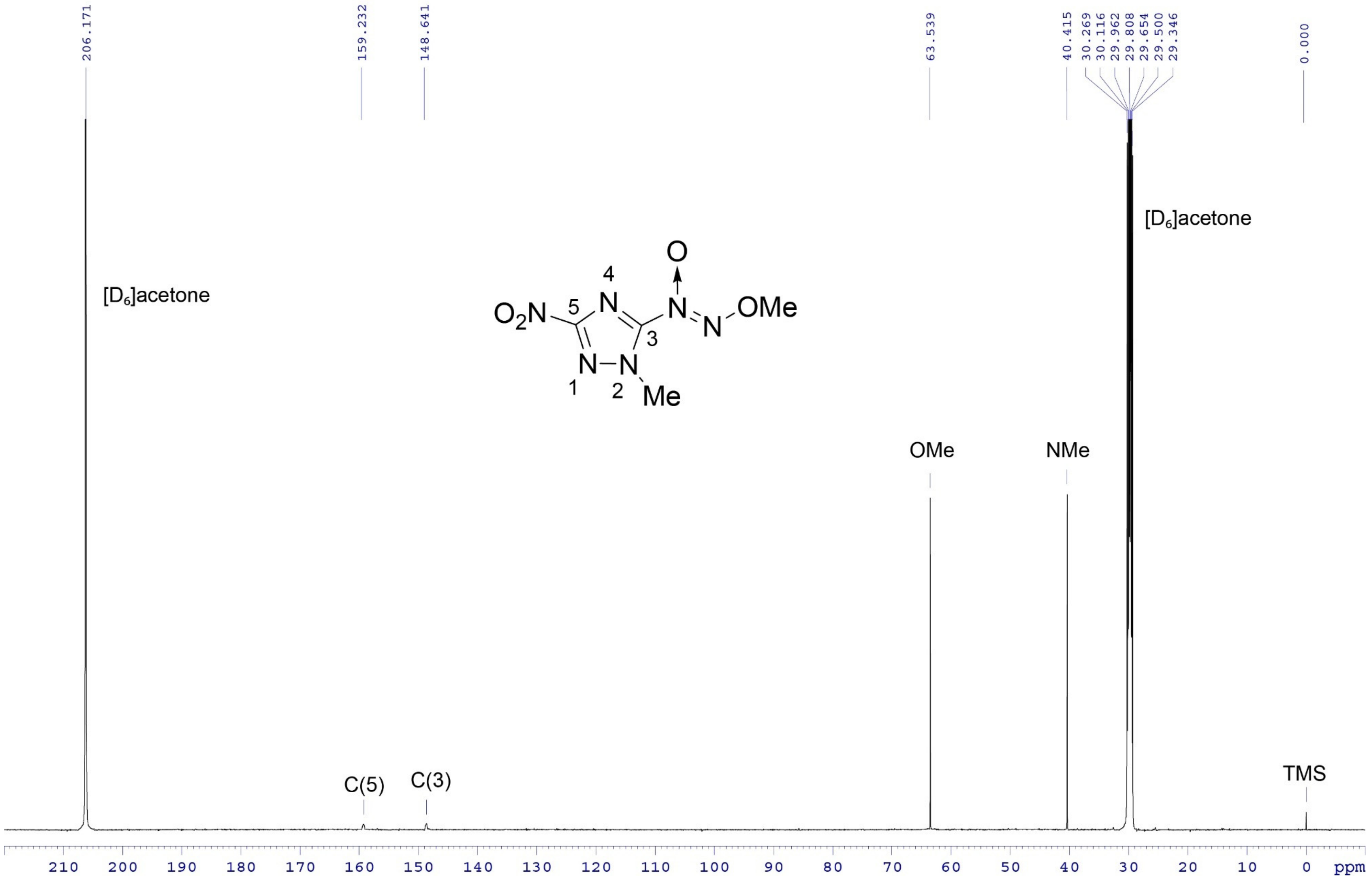

9.18.3 {^{1}H–^{13}C} HSQC spectrum of compound 2r [500.13 MHz, [D$_6$]acetone]

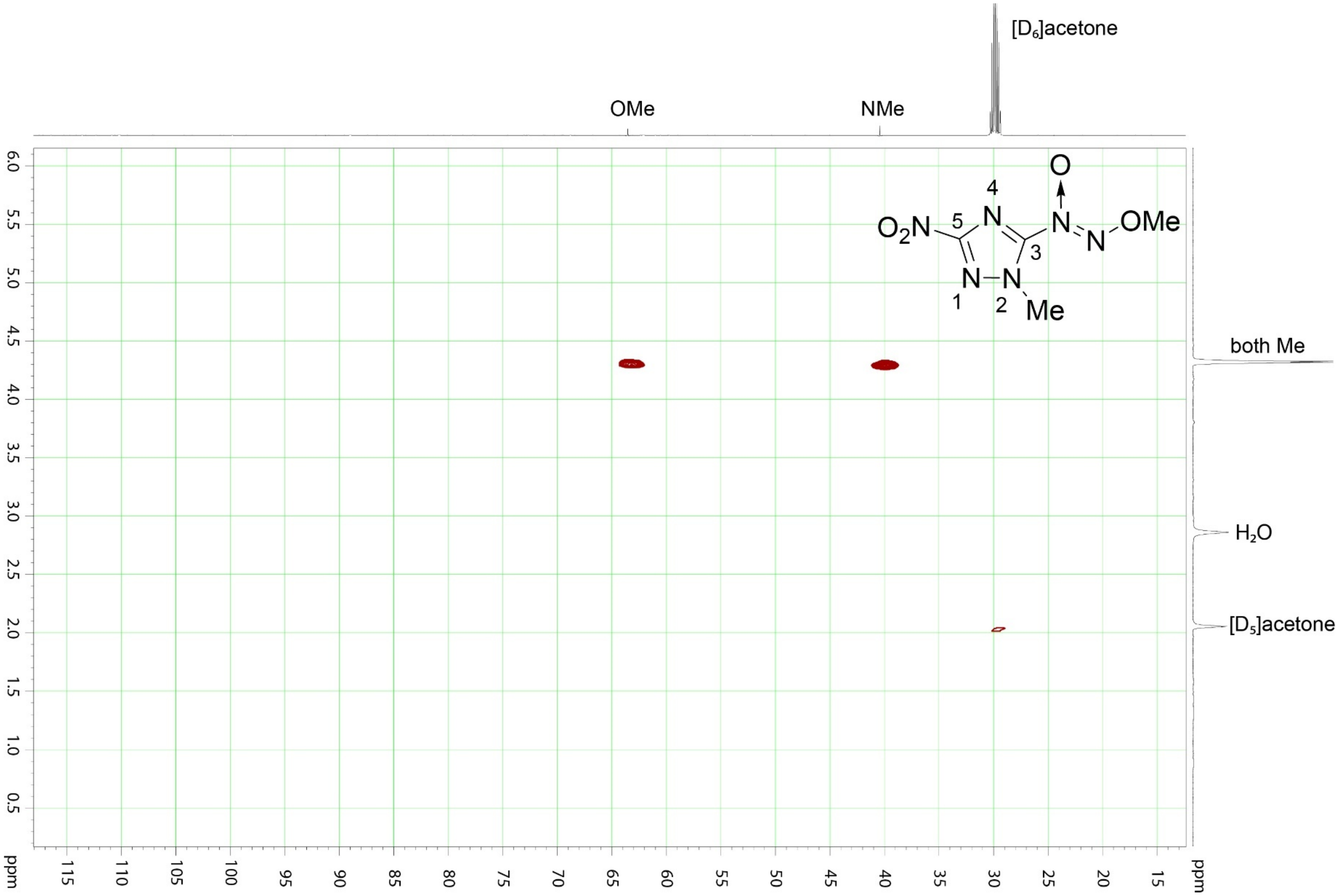

9.18.4 {^{1}H–^{13}C} HMBC spectrum of compound 2r [500.13 MHz, [D$_6$]acetone]

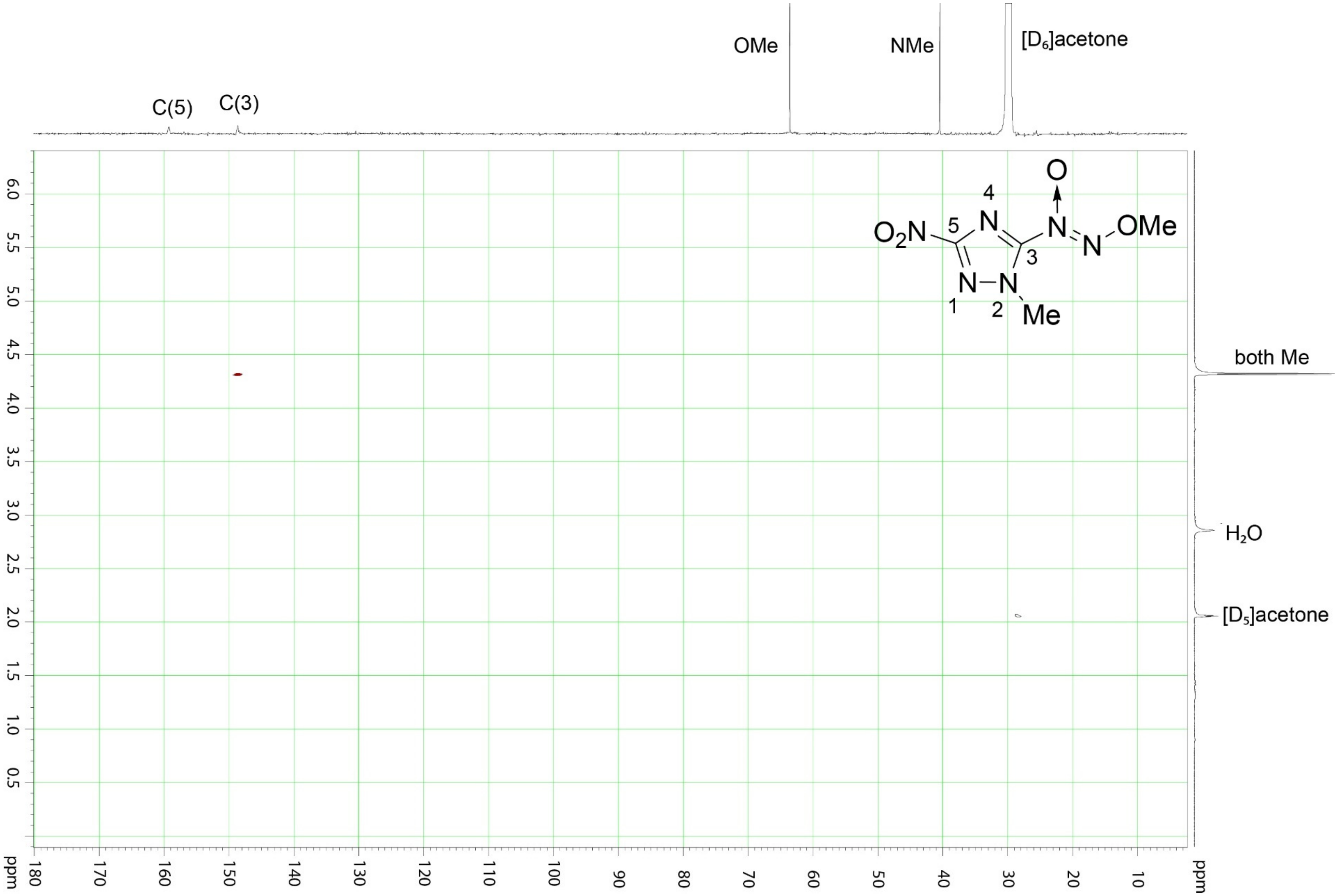

9.18.5 ^{14}N NMR spectrum of compound 2r [36.14 MHz, [D$_6$]acetone]

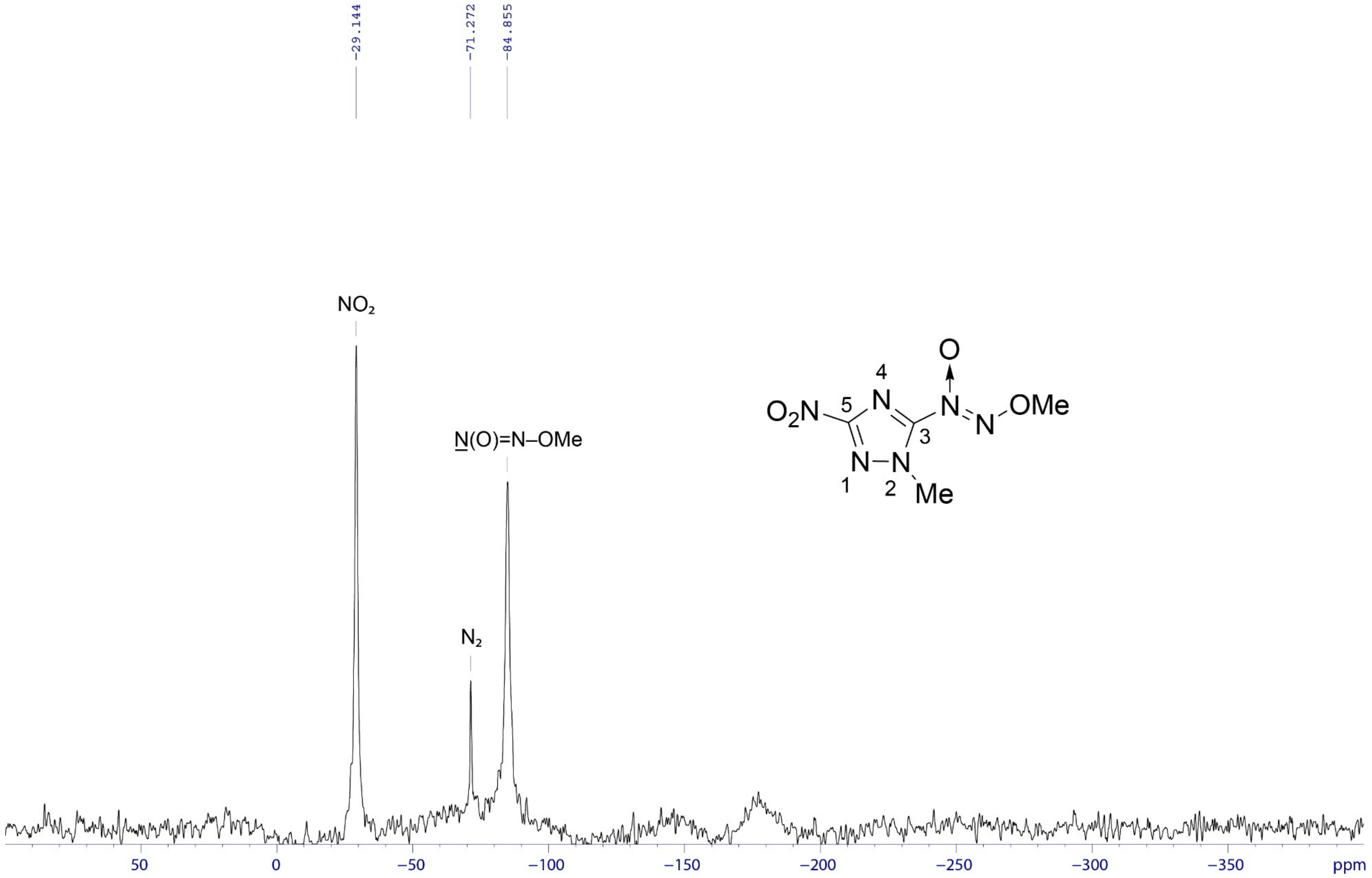

9.19.1 ^{1}H NMR spectrum of compound 2s [600.13 MHz, [D$_6$]acetone]

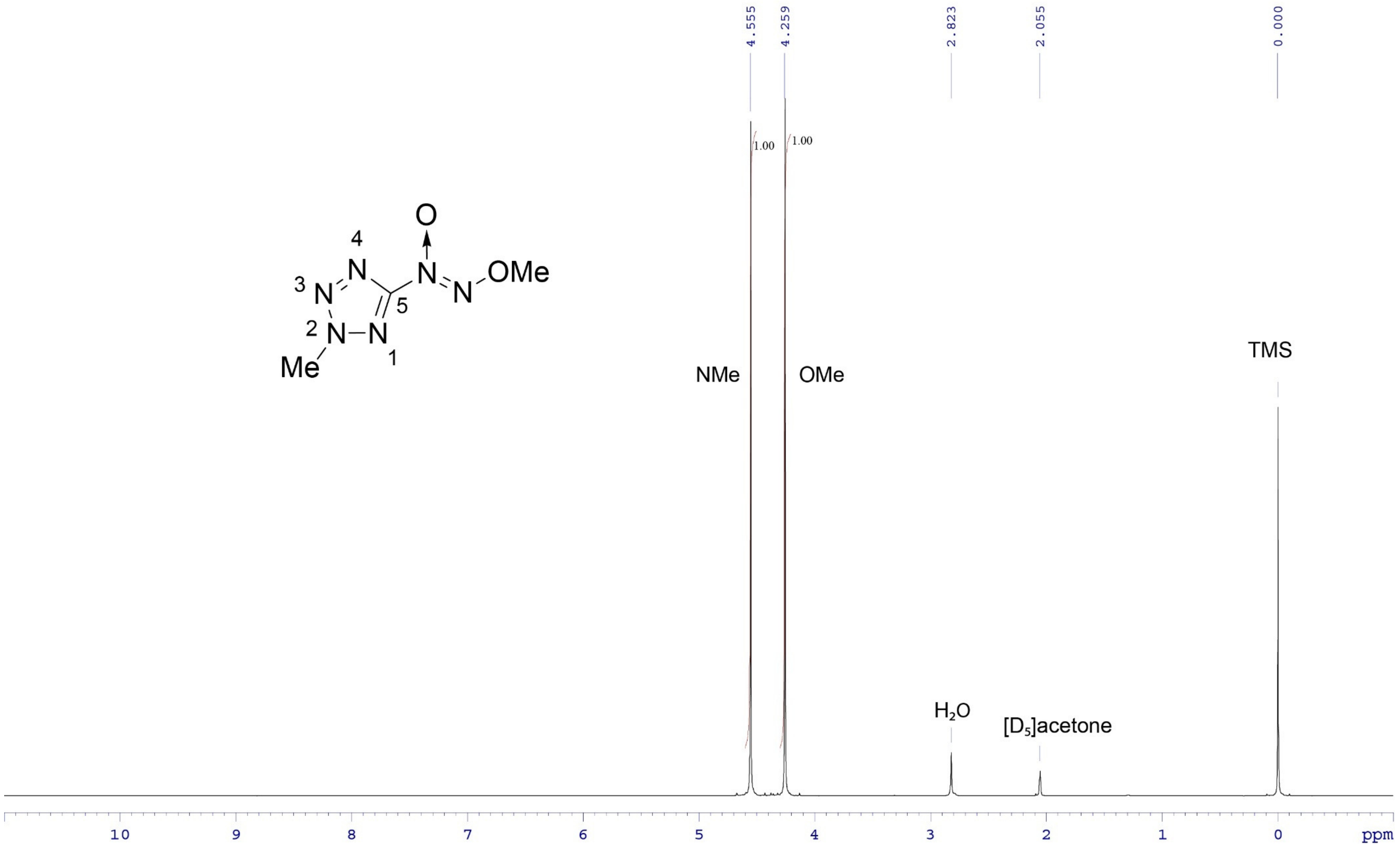

9.19.2 ^{13}C NMR spectrum of compound 2s [150.90 MHz, [D$_6$]acetone]

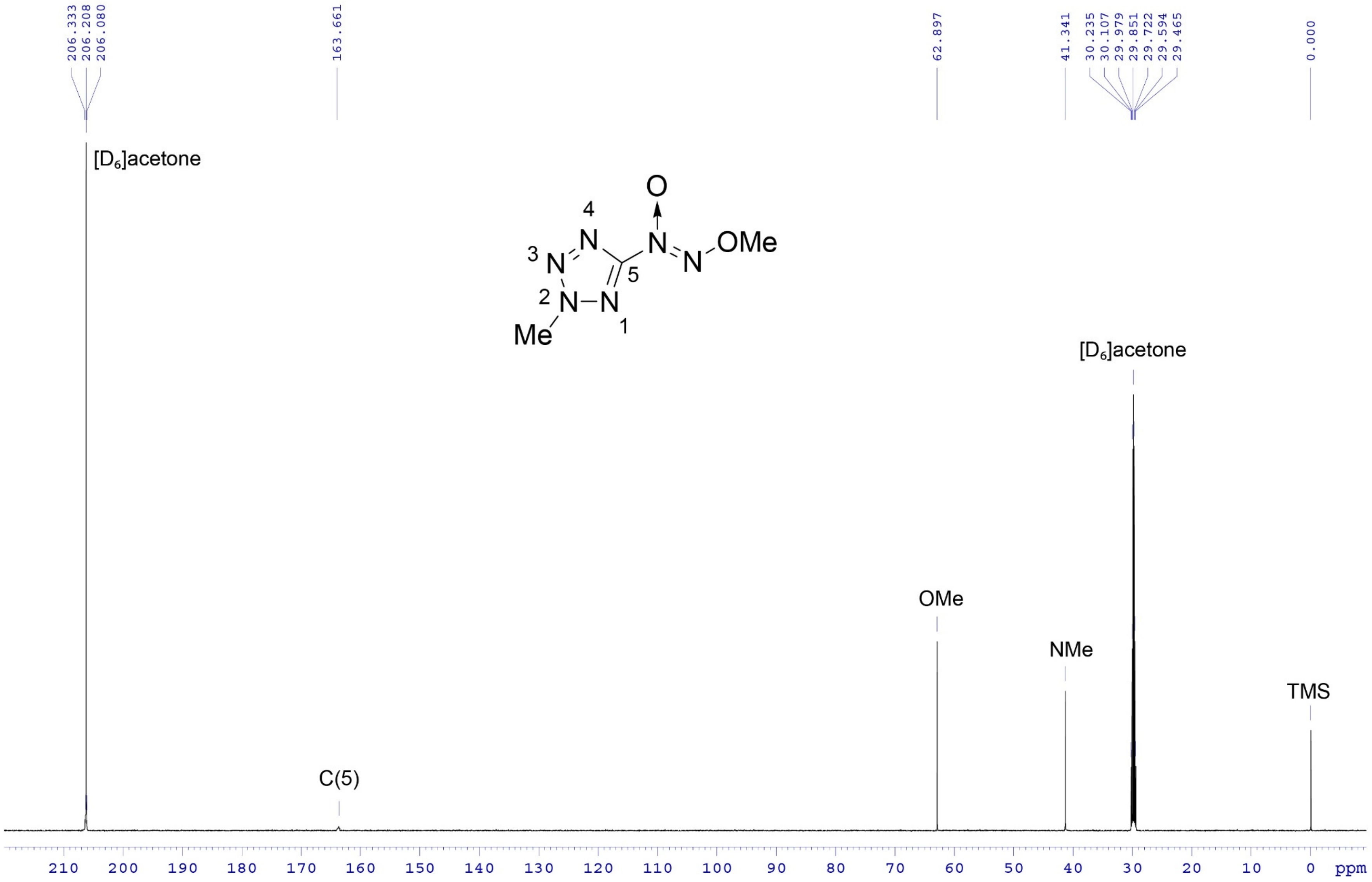

9.19.3 {^1H–^{13}C} HSQC spectrum of compound 2s [600.13 MHz, [D$_6$]acetone]

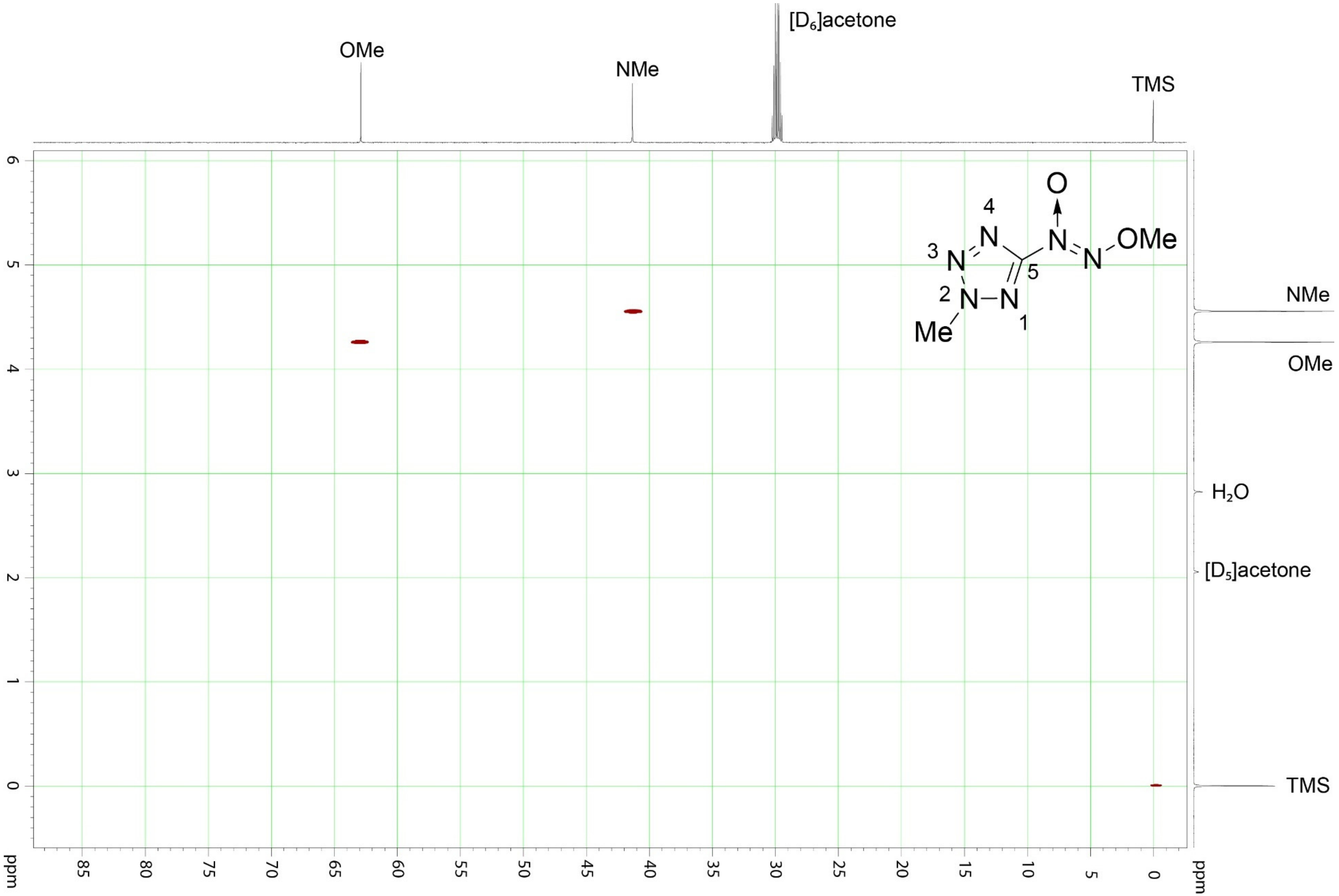

9.19.5 ^{14}N NMR spectrum of compound 2s [43.37 MHz, [D$_6$]acetone]

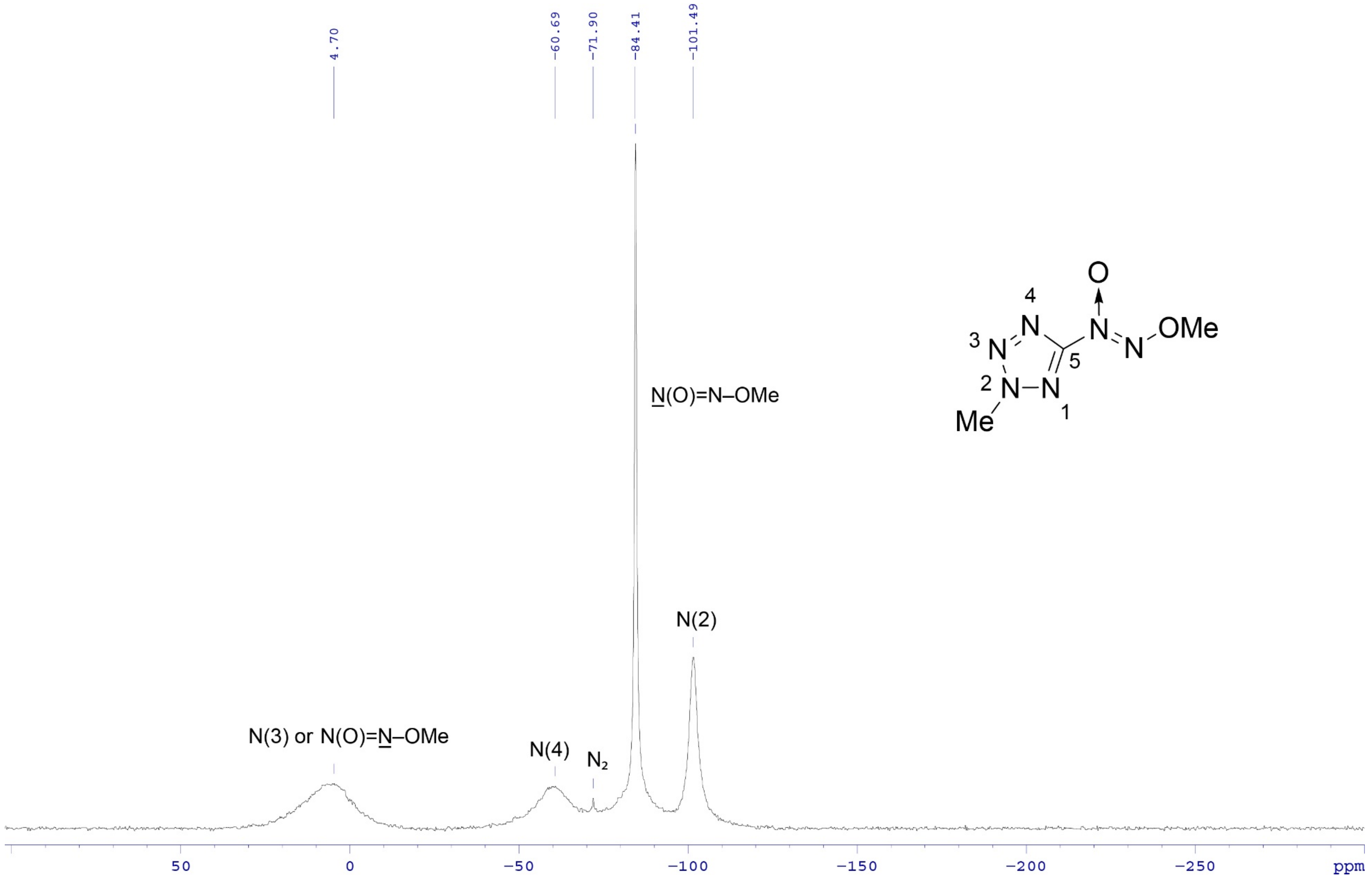

9.20.1 ^{1}H NMR spectrum of compound 4a [600.13 MHz, $CDCl_3$]

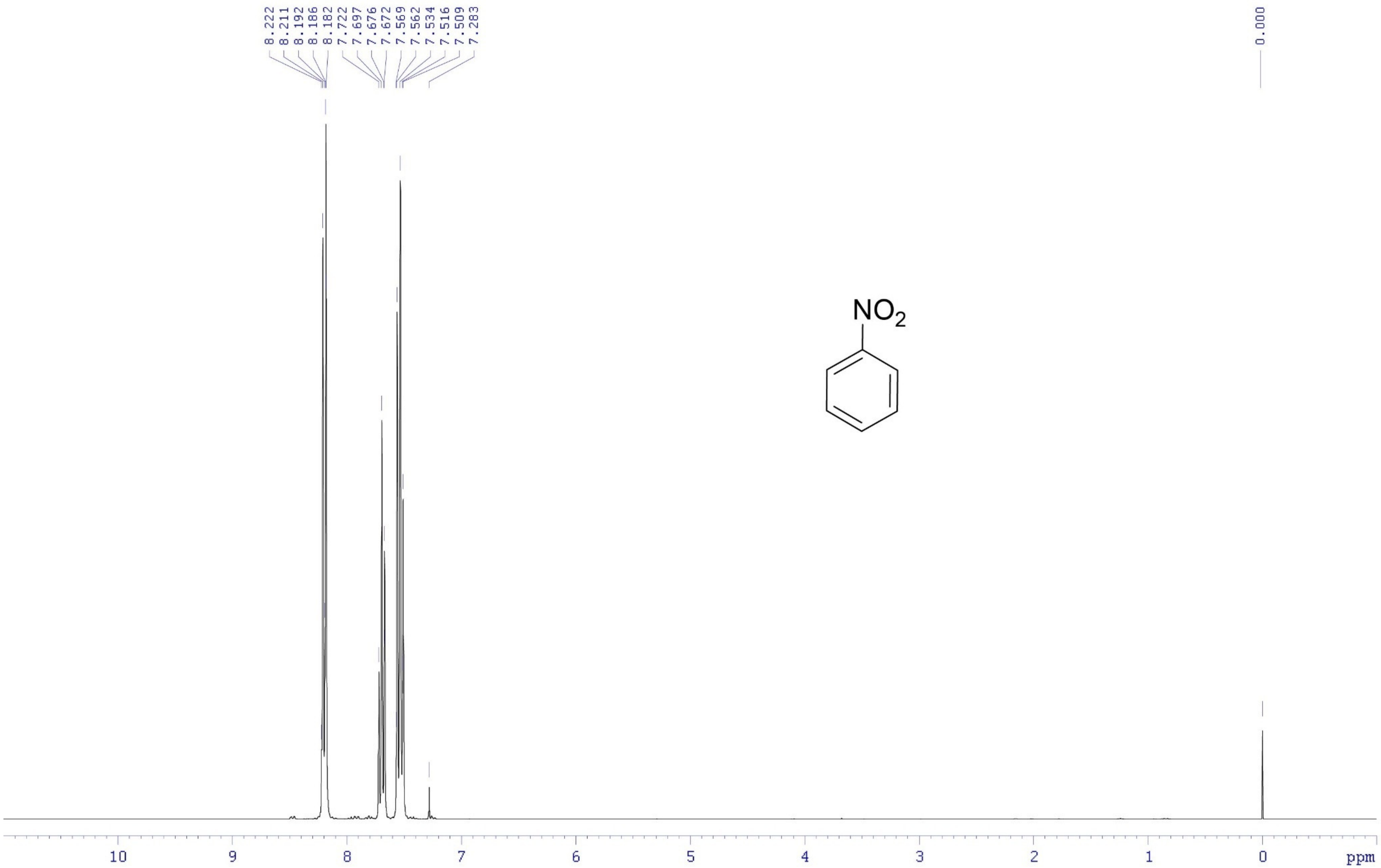

9.20.2 ^{13}C NMR spectrum of compound 4a [150.90 MHz, $CDCl_3$]

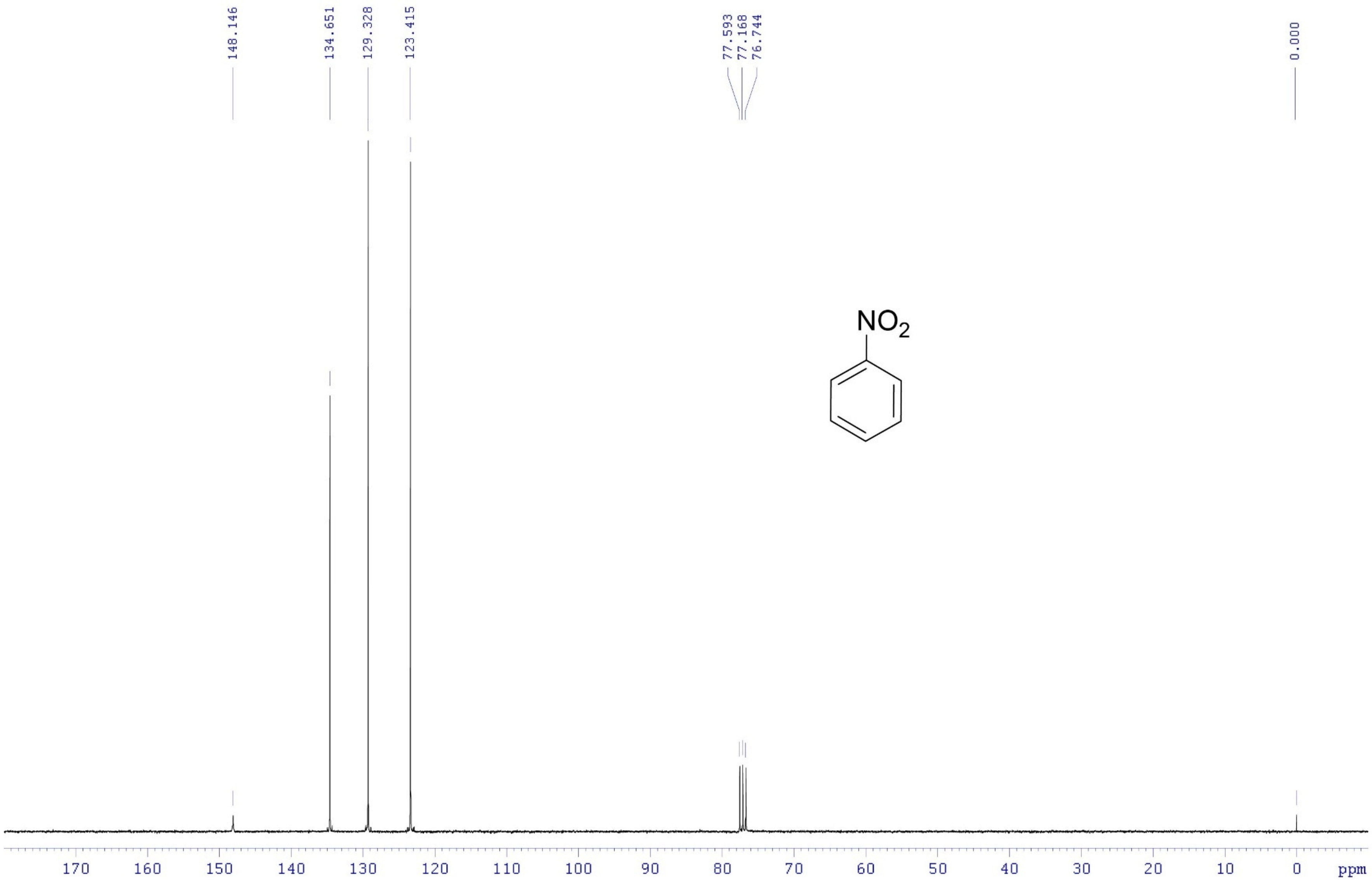

9.20.3 ^{14}N NMR spectrum of compound 4a [43.37 MHz, $CDCl_3$]

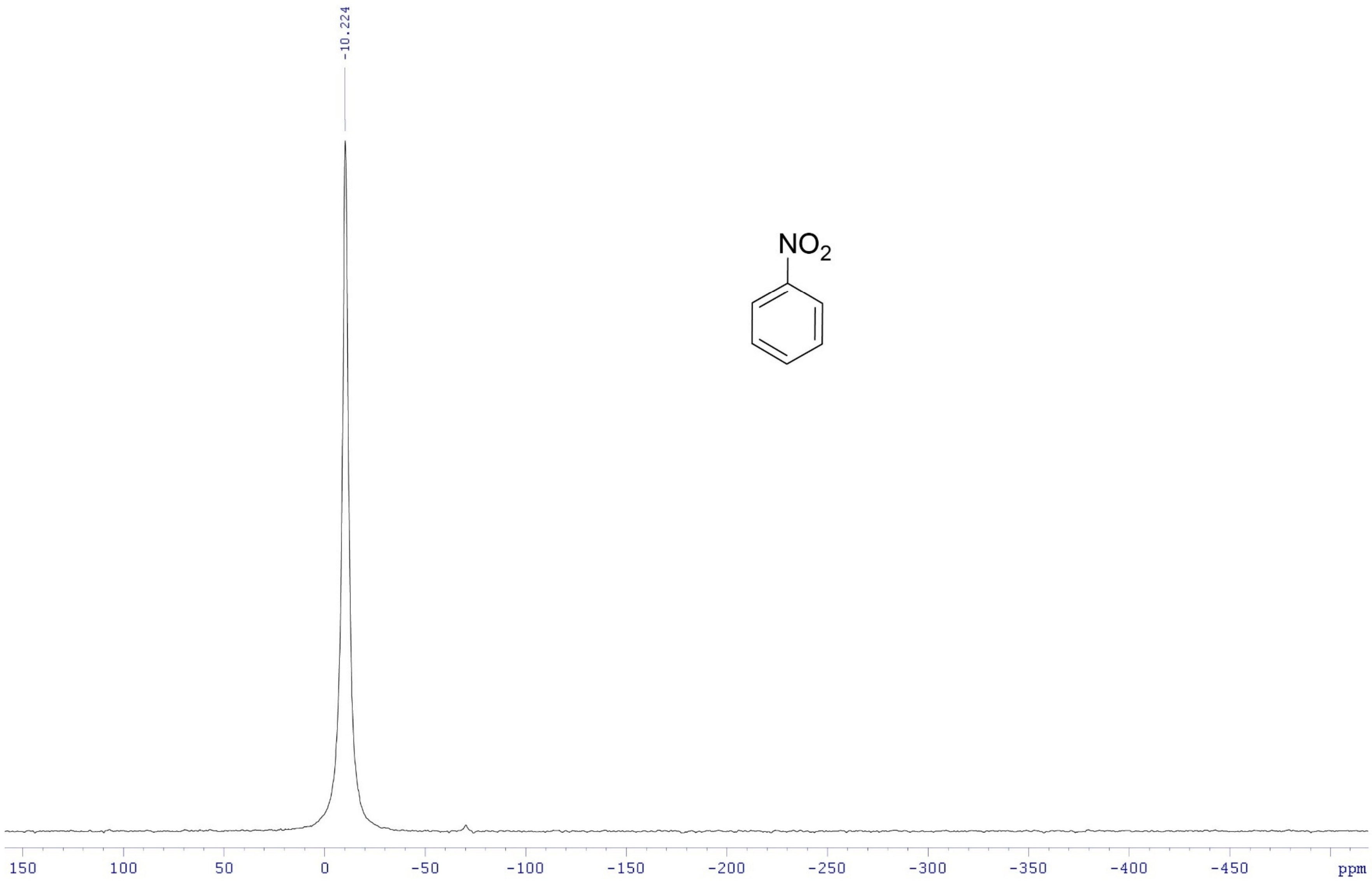

Supplement: Supplementary file 1 [file molecules-30-04723-s001.zip › molecules-4005536-supplementary.pdf]
